# Effects of High-Dose Ionizing Radiation in Human Gene Expression: A Meta-Analysis

**DOI:** 10.3390/ijms21061938

**Published:** 2020-03-12

**Authors:** Dimitrios S. Kanakoglou, Theodora-Dafni Michalettou, Christina Vasileiou, Evangelos Gioukakis, Dorothea Maneta, Konstantinos V. Kyriakidis, Alexandros G. Georgakilas, Ioannis Michalopoulos

**Affiliations:** 1 Centre of Systems Biology, Biomedical Research Foundation, Academy of Athens, 115 27 Athens, Greece; kanakogloud@biol.uoa.gr (D.S.K.); daphnelettos@biol.uoa.gr (T.-D.M.); christinevasil@mail.ntua.gr (C.V.); ge13010@central.ntua.gr (E.G.); ge13097@central.ntua.gr (D.M.); kyriakidk@pharm.auth.gr (K.V.K.); 2 Section of Cell Biology and Biophysics, Department of Biology, School of Sciences, National and Kapodistrian University of Athens, 157 01 Athens, Greece; 3 DNA Damage Laboratory, Physics Department, School of Applied Mathematical and Physical Sciences, National Technical University of Athens, 157 80 Athens, Greece; alexg@mail.ntua.gr; 4 Laboratory of Pharmacology, School of Pharmacy, Aristotle University of Thessaloniki, 541 24 Thessaloniki, Greece

**Keywords:** high-dose ionizing radiation, RNA-Seq, differential gene expression, DNA damage response

## Abstract

The use of high-dose Ionizing Radiation (IR) is currently one of the most common modalities in treatment of many types of cancer. The objective of this work was to investigate the effects of high-dose ionizing radiation on healthy human tissue, utilizing quantitative analysis of gene expression. To this end, publicly available transcriptomics datasets from human samples irradiated with a high dose of radiation and non-irradiated (control) ones were selected, and gene expression was determined using RNA-Seq data analysis. Raw data from these studies were subjected to quality control and trimming. Mapping of RNA-Seq reads was performed by the partial selective alignment method, and differential gene expression analysis was conducted. Subsequently, a meta-analysis was performed to select differentially expressed genes across datasets. Based on the differentially expressed genes discovered by meta-analysis, we constructed a protein-to-protein interaction network, and we identified biological pathways and processes related to high-dose IR effects. Our findings suggest that cell cycle arrest is activated, supported by our top down-regulated genes associated with cell cycle activation. DNA repair genes are down-regulated in their majority. However, several genes implicated in the nucleotide excision repair pathway are upregulated. Nevertheless, apoptotic mechanisms seem to be activated probably due to severe high-dose-induced complex DNA damage. The significant upregulation of CDKN1A, as a downstream gene of TP53, further validates programmed cell death. Finally, down-regulation of TIMELESS, signifies a correlation between IR response and circadian rhythm. Nonetheless, high-dose IR exposure effects regarding normal tissue (radiation toxicity) and its possible long-term outcomes should be studied to a greater extend.

## 1. Introduction

Regarding human exposure to Ionizing Radiation (IR), doses below 0.1Gy are classified as “low” [1], while doses normally used in medical procedures, such as Radiation Therapy (RT) (2-3Gy) are classified as high [2,3]. As such, apart from understanding the biological consequences of rare very high-dose exposures like in the case of nuclear accidents, a major field of radiobiological and clinical interest is the optimization of RT which usually involves moderate to high fraction doses.

The main therapeutic modality employed in RT are photon beams in the form of low Linear Energy Transfer (LET) radiation (X-rays, Gamma-rays), although high LET radiation (protons, alpha particles, and other heavy ions) are sometimes incorporated due to their precise dose localization. Radiation particles deposit more energy on the targeted tumor areas (a phenomenon known as the “Bragg peak” [4]) and have a higher Relative Biological Effectiveness (RBE) [5,6,7], while photon beams deposit a relatively small quantity of energy that disperses further to the surrounding healthy tissue due to scattering phenomena. In general, the idea behind RT is that the rapidly proliferating cancer cells are usually more sensitive to radiation than normal cells, while normal cells can usually repair themselves at a faster rate and retain their normal function. Therefore, the goal is to inhibit cancer cell multiplication potential, eventually leading to cell death, while minimizing dosage absorption in normal tissue, to prevent toxicity [5,8]. Nonetheless, IR exposure effects regarding healthy tissue (radiation toxicity) and its possible long-term outcomes should be studied to a greater extend.

Cancer and healthy cells are targeted alike, either directly through damage on their cellular molecules and especially on DNA strands or indirectly via the formation of free radicals, a phenomenon referred to as oxidative stress [9]. In essence, oxidative stress is a procedure of water radiolysis and involves the formation of intermediate, partially reduced oxygen species, collectively termed as Reactive Oxygen Species (ROS), that give rise to the formation of hydroxyl radicals that produce a number of adverse biological reactions by attacking structural and functional molecules [10], thus resulting in generalized cellular stress. Hydroxyl radicals can sometimes indirectly produce Single-Strand Breaks (SSBs) and a plethora of base and sugar lesions in DNA molecules, which can be cytotoxic or mutagenic [11], as well as crosslinks between two complementary DNA strands [12]. On the other hand, direct DNA damage primarily involves the induction of Double-Strand Breaks (DSBs) that represent the most lethal types of DNA damage, leading to cell death or genomic instability if left unrepaired. Finally, closely spaced DNA lesions (referred to as complex or clustered DNA damage) that may occur after IR exposure have been suggested to be highly repair-resistant or non-repairable. Therefore, they are considered highly significant biological lesions [13]. This continuously challenging process may lead to genomic instability and cancer [14], concurrently fueling DNA Damage Response (DDR) activation [15] which constitutes the main component of IR effects on a cellular level.

In general, DDR can be defined as the synthesis of functions (sensors, transducers, effectors) that orchestrate DNA damage sensing and signal transduction, triggering either DNA repair, cell survival, or cell death (apoptosis). Furthermore, pathways of cell cycle checkpoint control are also essential components of DDR [16,17]. Main pathways of DNA repair include Base Excision Repair (BER) and Nucleotide Excision Repair (NER), which repair DNA base damages, and Mismatch Repair (MMR), which corrects base mispairs and small loops that are often found in repetitive sequence DNA. In addition, Homology-dependent Recombination (HR) and Non-Homologous End Joining (NHEJ) act alone or together to repair DSBs and complex events such as inter-strand crosslinks [18,19]. Dysregulation of DDR mechanisms can cause several human disorders that are associated with cancer susceptibility, accelerated aging, and developmental abnormalities [20]. Moreover, like other types of stress, radiation exposure affects the development of the immune system through radiation-induced apoptosis, differentiation, and induction of inflammatory environment via different components of DDR [21,22].

The complete pattern of biological responses to different doses and radiation types is unclear and currently one of the most important questions in radiation biology. The general consensus is that results of IR exposure in any living organism involve a topical and/or systemic stress. A variety of responses is induced, including—but not limited to—oxidative stress in the irradiated area or in the whole body (through systemic non-targeted effects), DDR, DNA repair, and pro-inflammatory pathway initiation [23]. From a systems biology perspective, the aforementioned cellular mechanisms, as well as other related ones, can be examined through altered gene expression. Thus, in this work, we performed a Differential Gene Expression Analysis (DGEA), in human tissues exposed to high-dose IR, taking advantage of the wealth of publicly available RNA-Sequencing (RNA-Seq) data, as previously suggested [24,25,26]. Το this end, we carefully selected five datasets of healthy human cell samples, and in each of them, we identified Differentially Expressed Genes (DEGs) between irradiated and non-irradiated cells. Finally, we performed a meta-analysis, highlighting the common ground of high-dose effects.

## 2. Results

### 2.1. Data Collection, Filtering, Pre-Processing and Mapping

ENA queries identified 71 projects that fulfilled our search criteria. After manual curation, the Bioproject [27] accession numbers of the selected datasets (Table 1) were PRJNA494581 [28], PRJNA450083 [29], PRJNA421022 [30], PRJNA436999 [31], and PRJNA396832. We downloaded RNA-Seq-related FASTQ files of those Bioprojects. After the initial quality control, we performed soft trimming on raw RNA-Seq data, choosing PHRED score Q = 20 [32,33], removing on average ~2.4% of the nucleotide reads with more than 1% probability of an incorrect base call. PRJNA396832 trimming rate was 7.7% with soft trimming, due to adapter contamination and poor quality of reads.

Mapping of the trimmed reads to the reference genome and transcriptome (gentrome) was estimated at a rate of ~83%. There were distinct outliers during the quality control, trimming, and alignment processes, and this served as the first indicator that PRJNA396832 and PRJNA450083 were not of the desired quality for conducting our analysis. Without the outliers, mapping rate was at ~87% across the remaining 3 datasets (PRJNA421022, PRJNA436999, and PRJNA494581).

### 2.2. Differential Gene Expression

**Figure 1:**
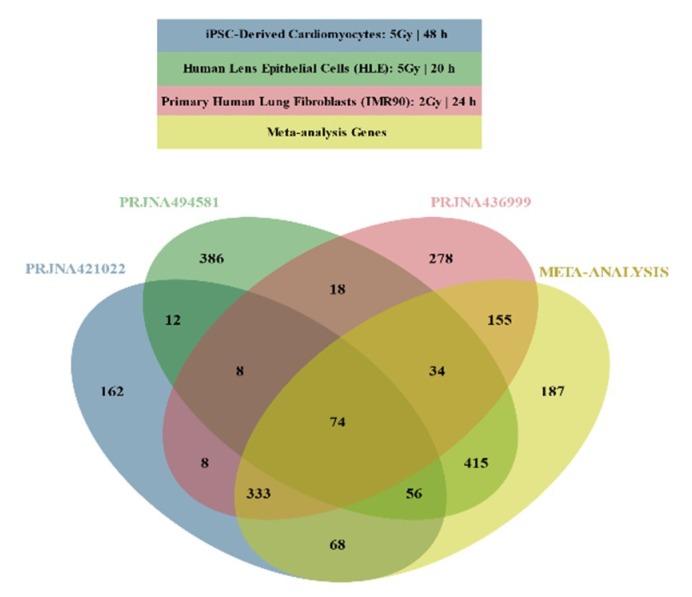
Venn diagram illustrating overlapping Differentially Expressed Genes (DEGs) across selected DEG lists and meta-analysis results.

It was suggested that the minimal number of biological replicates required for RNA-Seq based DGEA is 6 [34]. PRJNA396832 and PRJNA450083 had a single biological replicate for each condition, and were therefore excluded for subsequent analysis, as statistical significance could not be estimated. Studies that contained combinations of experimental conditions (PRJNA436999 2 Gy for 6 and 24 h and PRJNA494581 2 Gy and 5 Gy for 20 h), were split into distinct studies (Table 2) for DGEA. DESeq2 was used to identify DEGs in PRJNA421022 (Supplementary Materials, Table S1) in one of the distinct studies of PRJNA436999 (Supplementary Materials, Table S2) and in one of the distinct studies of PRJNA494581 (Supplementary Materials, Table S3). From 2006 genes that were considered DEGs in at least one dataset, 542 overlapped (Figure 1).

Meta-analysis was conducted using the three lists that derived from the DESeq2 analysis, yielding 1322 DEGs (Supplementary Materials, Table S4): 872 DEGs were found in at least one DESeq2 analysis but not in the meta-analysis; 187 genes that were not characterized as DEGs in any DESeq2 analyses were considered DEGs by meta-analysis (Figure 1).

Some of the genes that were identified as DEGs in the original RNA-Seq analyses were PCR-validated by their own groups. For PRJNA494581 dataset [28], ddPCR validated CDKN1A, AREG, H2BC13 (HIST1H2BL), GDF15, H3C11 (HIST1H31), H1-4 (HIST1H1E), H2BC10 (HIST1H2BF), and TP53INP1 as DEGs and B2M and RPL13A as non-differentially expressed genes. For PRJNA421022 dataset [30], qPCR validated PLK1, BIRC5, AURKB, KIF20A, TOP2A, and CCNA2 as down-regulated and CDKN1A and FDXR as up-regulated genes. Moreover, qPCR did not validate ANGPTL4 and SOGA3 as up-regulated genes, even if those genes were estimated as DEGs in their RNA-Seq data analysis. The DEG list of our meta-analysis agrees with all PCR validations.

### 2.3. Functional Enrichment Results

**Figure 2:**
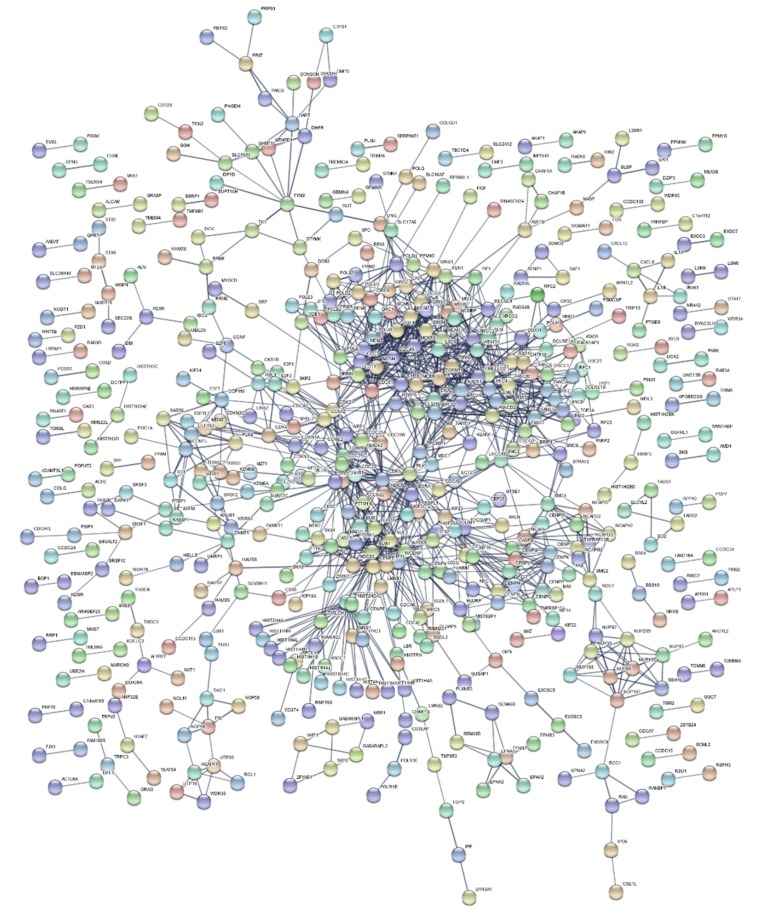
Protein–protein interaction network of DEGs from meta-analysis. Formulated protein clusters in the center of the network are associated with cell cycle processes and multiple DNA repair pathways.

Functional enrichment analysis of up- and down-regulated genes resulting from meta-analysis produced lists of statistically significant Gene Ontology (GO) [35] biological processes, KEGG [36] biological pathways (Table 3), and gene-targeting transcription factors (Table 4). A Protein–Protein Interaction (PPI) network of DEGs was also constructed (Figure 2).

## 3. Discussion

RNA-Seq is gradually becoming the predominant technique for transcriptome analysis, superseding microarrays. RNA-Seq technology is more sensitive in detecting genes with low expression levels, it lacks the associated background noise of hybridization-based techniques, and it is more reproducible [24]. Furthermore, a crucial limitation of microarrays is their inability to study the expression of genes for which no probe is available on the chip. So far, there is no gold-standard methodology for analyzing the transcriptome using RNA-Seq technology. Poorly designed pipelines for differential gene expression can have detrimental effects, compromising the experimental results. Suboptimal pre-processing of raw data, directly affects the mapping process, resulting in poor mapping rates (<60%) [37].

An extensive debate [38,39,40] on the effects of the pre-processing step of trimming, suggests that reads should be carefully trimmed. Illumina Next-Generation Sequencing (NGS) platforms produce sequences of between 25–250 nucleotides, the colorimetric signals of which are translated by an internal Illumina software (CASAVA) to base calls and are represented in FASTQ [41] file format. Minimal trimming (Q < 10) keeps low quality base calls in NGS analyses, adding unreliable and random sequences to the final dataset [38]. However, hard trimming (Q > 30) of reads can have a particularly strong negative impact on RNA-Seq-based gene expression estimates, as it introduces unpredictable and unwanted biases [40]. Hence, we used soft trimming (Q = 20) as a balance, preserving biological information that was not ideally recorded, while discarding non-sense information.

The expected mapping rate of RNA-Seq reads is between 70% and 90% when mapped against the human genome and slightly less when mapped against the transcriptome [37]. In conventional RNA-Seq mapping, multiple reads are mapped across splice junctions [42]. The inability of conventional algorithms, like BWA [43], to handle spliced transcripts renders them obsolete. Splice-aware aligners [44], such as Hisat2 [45], handle mapping in a more efficient manner. However, these traditional approaches require significant computational resources amidst an explosively growing storage-hungry environment [46]. Alignment-independent methods, such as Salmon [47], bypass the mapping step and proceed to quantify directly transcript abundance, boasting a more lightweight and significantly faster novel approach. Our mapping rate, minus the outliers, was calculated at a satisfactory level of ~87% using Salmon’s selective alignment method.

The resulting datasets combined with DESeq2 which utilizes estimates of dispersions and logarithmic fold changes by incorporating data-driven prior distributions [48], yielded substantial statistically significant results. Instead of using transcript levels, as in a classical meta-analysis, we chose Mosteller–Bush, a method which is based on the combination of weighted *z*-values for the independent studies, which are calculated from the *p*-values produced by DESeq2. PCR-based validations showed that our meta-analysis outperformed original RNA-Seq data analyses.

Although a fraction of differentially expressed genes overlap among the 3 DESeq2 studies, a meta-analysis was conducted to enhance the validity of our DGEA (Figure 1). In all three DESeq2 DEG lists (Supplementary Materials, Table S1–S3) and in the meta-analysis DEG list (Supplementary Materials, Table S4), the number of statistically significant under-expressed genes considerably surpasses the number of over-expressed genes. Such a result may indicate that cell cycle arrest has been activated, which is supported by the top down-regulated genes (i.e., MKI67, CCNA2, CDK1, PLK1, and CDCA3) identified by the meta-analysis and associated with cell cycle activation [49]. This difference between up- and down-regulated genes, as well as cell cycle arrest activation, coincides with the primary results for PRJNA421022 dataset [30]. At the same time, observed up-regulation of CDKN1A, responsible for cell cycle G1 phase arrest, in response to a variety of stress stimuli further upholds this suggestion. p21 protein coded by this gene (known to be interdependent with tumor suppressor protein TP53) is also responsible for inhibiting cellular proliferation in response to DNA damage [50]. In addition, it is highly correlated with DNA repair, while also being instrumental in the execution of apoptosis. Although p21 is assumed to play a key role as “genome guardian”, it can alternatively act as the mediator of genomic instability, cellular senescence, and carcinogenesis under certain circumstances, like IR exposure and TP53 deficiency [51]. Another substantial result is the significant up-regulation of GDF15, associated with the response to oxidative stress and induction of inflammatory environment, thus coinciding with DDR and IR response in general [49,52].

TP53 is essential in DDR mechanisms through its downstream responses, which include cell cycle arrest, DNA repair, and apoptosis. The accurate transition from G1 phase of the cell cycle to S phase is crucial for a controlled cell proliferation, and its misregulation promotes oncogenesis [53]. G1 arrest provides the cell adequate time to repair the DNA damage. Should repair be unsuccessful, TP53 levels drop and CDK-cyclin protein kinase activity resumes, leading to entry into S phase and possible apoptosis triggering. [54]. In our results, for samples selected over 20 h post irradiation, TP53 indeed shows no altered expression, while MDM2, as a TP53 downstream gene transcriptionally activated by it [55], is over-expressed.

Regarding enrichment results for down-regulated genes (Table 3), cell cycle checkpoint as well as various DNA repair mechanisms are over-represented. These results may indicate that DNA repair genes (i.e., MSH2, MSH6, XRCC3, and POLA2) are suppressed, due to programmed cell death. However, NER-associated genes DDB1, DDB2, and XPC where found up-regulated. DNA repair in general, due to its complexity, requires balanced expression of its genes in order to avoid erroneous repairs [56]. Over-represented gene-targeting factors E2F1, E2F2, RB1, TFDP1, and TFDP2 (Table 4) are connected through involvement in cell cycle G1/S phase transition, TP53 regulation, and cellular senescence [49]. Moreover, under-expression of DNA repair genes, mediated by the RB/E2F pathway, may play a causal role in senescence induction [57]. In the case of up-regulated genes, p53 signaling pathway, DDR, and apoptotic mechanisms seem to be activated. In addition, platinum drug resistance (Table 3) could arise from increased DNA repair, decreased mismatch repair, defective apoptosis, and altered oncogene expression [58].

Regarding the PPI network (Figure 2), edges corresponding to protein interactions, were constructed with sizeable restrictions regarding the validity of their sources and their assigned score. Distinct clusters were formulated, coinciding with the main components of high-dose IR response. Simultaneously, through these clusters, the network validates the biological processes and pathways derived from the enrichment results. Two major, densely packed clusters are formed, representing multiple cell cycle processes. These clusters are indicative of a collection of DNA repair pathways (GO:0006281), such as DSBs repair (RAD51, BLM, DNA2), Mismatch Repair (MSH6, MSH2), Homologous Recombination (XRCC3), and NER (DDB1, DDB2, XPC, POLE). Furthermore, TIMELESS, a gene found in the center of the network due to its contribution to DSB repair, also acts as a circadian rhythm pathway regulator [49]. Additional circadian rhythm-related results are the under-expression of TYMS and the over-expression of CRY2.

Circadian genes are known to regulate a variety of cellular processes, including cell cycle, apoptosis, and DNA damage repair [59]. Both oxidative defense mechanisms and repair of X-ray induced DSBs in DNA are synchronized by circadian rhythms; thus, RT timing needs to be coordinated as we enter personalized medicine [60]. Furthermore, disruption in circadian gene expression is associated with increased incidence of cancers and gliomas [61]. Finally, the circadian clock system also controls various parameters of the immune system and its biological defense functions [62]. This correlation between circadian clock dysregulation and IR response may reveal possible underlying mechanisms of chronic inflammatory disease development. In addition, understanding the interplay between circadian rhythm, cell cycle, cell proliferation, and DNA repair will deliver benefits in RT by reducing its side effects on healthy tissues.

Biological response to IR (high doses), especially at the organism level, is complicated and partially unknown. RNA-Seq as an -omics methodology provides information on gene expression for several thousand proteins. This opens a unique opportunity to approach this difficult task of delineating the mechanisms triggered after radiation-induced stress, at a systems biology level. Therefore, in this study, after critical screening of several RNA-Seq datasets and applying state-of-the-art bioinformatics and meta-analysis, we were able to identify: (A) 1322 DEGs (371 up-regulated, 951 down-regulated, Supplementary Materials, Table S4); (B) cell cycle checkpoint activation, apoptosis, and various down-regulated repair genes. The last probably relates to late post-irradiation time points (20–48 h), where repair is expected to be completed and the cell is sent to apoptosis. Another suggestion is that transcriptional up-regulation of DNA repair genes by genotoxic stress (p53 activation, Table 3 and Table 4) is counteracted by possible DNA damage that blocks transcription [56]; (C) indication of cellular senescence; (D) association of IR response with the circadian clock.

## 4. Materials and Methods

**Figure 3:**
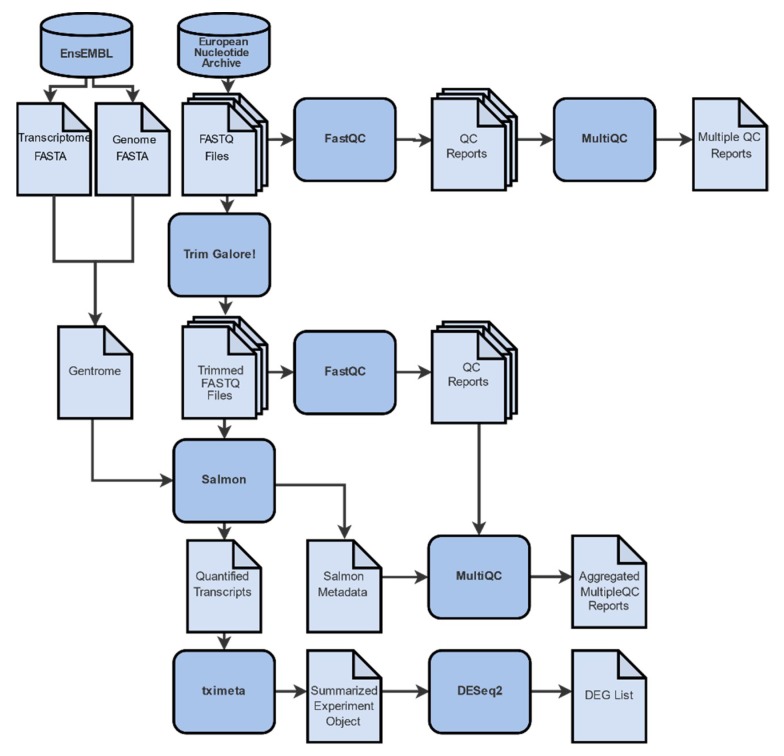
Differential gene expression analysis workflow is comprised of 4 distinct steps: (A) Data collection from the online repositories ENA and EnsEMBL. (B) Quality control and trimming. A conventional analysis pipeline of RNA-Seq data starts with the pre-processing of the raw reads with FastQC, MultiQC, and Trim Galore! (C) Gene abundance quantification with Salmon and tximeta by mapping of the reads to a reference genome and/or transcriptome. (D) Differential gene expression analysis with DESeq2, where gene expression levels of all mapped transcripts are quantified and normalized in order to define differentially expressed genes.

Our RNA-Seq analysis involves a pipeline (Figure 3) of in silico processes, each with its own specific parameters (Appendix A, Table A1), where files undergo a series of transformations. In each step, data are manipulated in a way that information is retained and expanded by additional meta-data.

### 4.1. Datasets

We searched for datasets available in public repositories to identify the studies that performed RNA-Seq in normal and ionized tissues. The appropriate datasets were identified using the European Nucleotide Archive (ENA) advanced search engine [63]. We narrowed our search down to human Illumina RNA-Seq studies which involved ionizing radiation. More specifically, in the read domain of ENA advanced search, our query was: *“instrument_platform = “ILLUMINA” AND library_strategy = “RNA-Seq” AND tax_eq (9606)”*, and in the study domain, our query was: “(study_name = “*ionizing*” OR study_title = “*ionizing*” OR study_description = “*ionizing*” OR study_name = “*alpha particle*” OR study_title = “*alpha particle*” OR study_description = “*alpha particle*” OR study_name = “*irradiation*” OR study_title = “*irradiation*” OR study_description = “*irradiation*” OR study_name = “*X-ray*” OR study_title = “*X-ray*” OR study_description = “*X-ray*” OR study_name = “*X ray*” OR study_title = “*X ray*” OR study_description = “*X ray*” OR study_name = “*gamma ray*” OR study_title = “*gamma ray*” OR study_description = “*gamma ray*” OR study_name = “*positron*” OR study_title = “*positron*” OR study_description = “*positron*” OR study_name = “*radiotherapy*” OR study_title = “*radiotherapy*” OR study_description = “*radiotherapy*” OR study_name = “*ionising*” OR study_title = “*ionising*” OR study_description = “*ionising*” OR study_name = “*IR-induced*” OR study_title = “*IR-induced*” OR study_description = “*IR-induced*”) AND tax_tree(9606)”. The first query yielded 10,541 studies and the second query yielded 504 studies. Afterwards, we selected the overlapping study names. The studies were then manually curated in order to exclude non-irradiated, UV-irradiated, and tumor samples.

### 4.2. Raw Read Evaluation

Extensive quality control was performed on RNA-Seq data of each sample using FastQC (*version 0.11.8*) [64], and summaries were produced by MultiQC (*version 1.8*) [65] to evaluate the integrity of RNA-Seq experiments. Quality control reports mainly involved the analysis of sequence accuracy, presence of PCR artifacts, and adaptor sequences that were not automatically cleaned by Illumina platforms, GC content, k-mer levels, etc. Surgical elimination of low-quality regions, known as “trimming”, was performed—when necessary—by Trim Galore! (*version 0.6.4*) [66], a wrapper package around Cutadapt (*version 2.8*) [67], and FastQC. Consequently, results where re-evaluated with FastQC and MultiQC to verify that the quality of raw data had improved after the trimming process.

### 4.3. Sequence Alignment

Mapping aligns trimmed sequence reads against a known genome and transcriptome. Its efficiency mainly depends on the bioinformatics tools used and the quality of the sequences. Reads were directly mapped into *Homo sapiens* reference genome and transcriptome FASTA-formatted sequences. To this end, we used the latest release of Salmon (*version 1.1.0)* [47] which adopts a selective-alignment algorithm in order to overcome the shortcomings of lightweight approaches, without the additional computational burden of traditional alignment [68]. We produced the transcriptome index for Salmon via the partial selective alignment method, mapping the transcriptome to the genome, extracting the relevant portion out of the genome, and, finally, indexing it along with the transcriptome.

### 4.4. Transcript Quantification

The entirety of the statistical analysis was performed using packages provided by Bioconductor (*BiocManager version 3.10*) [69,70], a suite for analyzing high-throughput genomic data in R (*version 3.6.2*) [71] statistical programming language. All R code was executed through RStudio server (*version 1.2.5033*) [72]. Transcript-level quantification was estimated using tximeta (*version 1.4.3*) [73], an expansion of tximport (*version 1.14.0*) [74].

### 4.5. Differential Gene Expression Analysis

Transcript-level quantification data were processed for DGEA, using DESeq2 (*version 1.26.0*) [48]. Studies with more than two distinct conditions were split in order to analyze genes that were differentially expressed between cells exposed to a specific dose for a specific time point and their corresponding control samples. Exported lists containing statistically significant differentially expressed genes include metrics such as Log_2_ Fold Change (Log_2_FC), *p*-values, and False Discovery Rate (FDR)-adjusted [75] *p*-values for each gene. The lists were further annotated using org.Hs.eg.db (*version 3.8.2*) [76] to include HGNC [77] gene symbols and gene names. The threshold for statistical significance was set at the adjusted *p*-value < 0.05, as non-adjusted *p*-values are not to be considered [75].

### 4.6. Meta-Analysis

**Figure 4:**
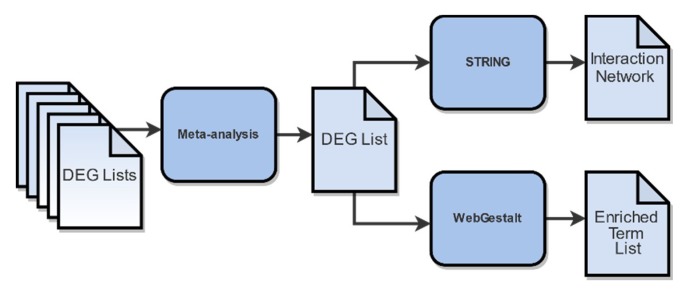
Functional enrichment analysis workflow. Differential Gene Expression Analysis (DGEA)-derived genes, subdued to meta-analysis, yielded the final DEGs of utmost statistical significance. Inputting these DEGs into WebGestalt and STRING generated the resulting enriched terms and PPI network, respectively.

DEG lists were further combined in a meta-analysis (Figure 4) to identify genes of differential expression across studies. In order to achieve optimal results, only DEG lists derived from similar condition groups regarding dose and time of post-irradiation collection were considered. To this end, we excluded the DEG list derived from the PRJNA436999 samples that were collected 6 h post-irradiation, leaving only the DEG list from the samples that were collected after more than 20 h. Finally, the DEG list acquired from the PRJNA494581 samples after 2 Gy irradiation was also excluded, as it was considered an outlier, due to its low DEG number.

Our meta-analysis combined unadjusted *p*-values of each study for every gene, using a weighted version of Stouffer meta-analysis [78], proposed by Mosteller and Bush [79]. For each gene and study, its two-tail unadjusted *p*-value was converted into an one-tail *p*-value, based on the sign of the corresponding Log_2_FC. For each one-tailed *p*-value, the corresponding *z*-score was calculated using the inverse normal distribution function (Φ^−1^). Meta-analysis *p*-value for each gene was calculated from the weighted *z*-score sum, using the normal distribution function (Φ):

$$p=\Phi \left(\frac{\sum_{i=1}^{k}n_{i}\Phi^{-1}\left(p_{i}\right)}{\sqrt{\sum_{i=1}^{k}{n_{i}}^{2}}}\right)$$

where *p_i_* is the DESeq2-derived *p*-value, and *n_i_* is the number of samples of study *i* and *k* the number of studies. Finally, *p*-values underwent FDR adjustment, and 0.05 was selected as threshold for statistical significance.

To evaluate the efficiency of our method, we compared the experimentally validated DEGs derived from the studies the RNA-Seq data of which we chose for analysis with our own estimations.

### 4.7. Functional Enrichment Analysis and Gene Network Construction

To highlight the biological background of DEGs, a functional enrichment analysis (Figure 4) was performed using the WEB-based Gene Set Analysis Toolkit (WebGestalt) [80]. We selected Over-Representation Analysis (ORA) [81] method which performs a statistical evaluation of the fraction of genes in a particular pathway found among the set of genes showing changes in expression. Our terms of interest include biological process GO terms [35], KEGG biological pathways [58], and gene-targeting transcription factors. The statistical significance of each over-representation of biological terms was estimated using hyper-geometric distribution. *p*-values were FDR-adjusted, and terms with adjusted *p*-values < 0.05 were considered statistically significant.

In order to investigate the interactome of the DEGs and identify possible underlying cell mechanisms, we constructed their Protein–Protein Interaction (PPI) network, using STRING (*version 11.0*) [82]. Edges of the network, corresponding to protein interactions, were determined solely based on text mining sources with high confidence (Table A1).

## Figures and Tables

**Table 1:** Information of experiment accessions and sample description.

Bioproject	Geo	IR Type	Tissue	Condition	Sample Count
**PRJNA494581**	GSE120805	X-rays	Human Lens Epithelial Cells	Control	5
				2 Gy | 20 h	5
				5 Gy | 20 h	5
**PRJNA421022**	GSE107685	X-rays	iPSC-Derived Cardiomyocytes	Control	3
				5 Gy| 48 h	3
**PRJNA436999**	GSE111437	X-rays	Primary Human Lung Fibroblasts (IMR90)	Control | 6 h	3
				Control | 24 h	3
				2 Gy | 6 h	3
				2 Gy | 24 h	3
**PRJNA396832**	GSE102145	X-rays	Skin Fibroblasts (WS1)	Control	1
				5 Gy | 24 h	1
**PRJNA450083**	GSE113125	Gamma-rays	Skin Fibroblasts	Control	1
				5 Gy | 1 h	1
			iPSC-Fibroblasts	Control	1
				5 Gy | 1 h	1
			iPSC-Neural Progenitor Cells	Control	1
				5 Gy | 1 h	1

**Table 2:** Statistically significant gene counts derived from the differential gene expression analysis for each comparison within each dataset. The table includes information about experimental sample parameters, dataset accession numbers, and total mapped gene counts for each dataset.

Bioproject Accession	PRJNA421022	PRJNA436999		PRJNA494581	
**GEO Accession**	GSE107685	GSE111437		GSE120805	
**IR Type**	X-rays	X-rays		X-rays	
**Cell Type**	iPSC-Derived Cardiomyocytes	Primary Human Lung Fibroblasts (IMR90)		Human Lens Epithelial Cells (HLE)	
**Dose**	5 Gy	2 Gy	2 Gy	2 Gy	5 Gy
**Time Point**	48 h	6 h	24 h	20 h	20 h
**DEG Counts**	721	353	908	59	1003

**Table 3:** Enriched biological processes (Gene Ontology—GO) and enriched biological pathways (KEGG) for up- and down-regulated genes after meta-analysis.

Gene Set	Description	Source	FDR
**Up-Regulated Genes** 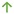			
GO:0072331	Signal transduction by p53 class mediator	GO	0.0022959
hsa04115	p53 signaling pathway	KEGG	2.24 × 10^−9^
GO:0042770	Signal transduction in response to DNA damage	GO	0.009620632
GO:0097193	Intrinsic apoptotic signaling pathway	GO	0.010992521
GO:0071496	Cellular response to external stimulus	GO	0.01930771
GO:0104004	Cellular response to environmental stimulus	GO	0.032524732
GO:0008643	Carbohydrate transport	GO	0.037630683
hsa01524	Platinum drug resistance	KEGG	0.032524732
**Down-Regulated Genes** 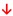			
GO:0000075	Cell cycle checkpoint	GO	0
hsa04110	Cell cycle	KEGG	0
GO:0051321	Meiotic cell cycle	GO	0
GO:0044772	Mitotic cell cycle phase transition	GO	0
GO:0045930	Negative regulation of mitotic cell cycle	GO	0
GO:1902850	Microtubule cytoskeleton organization involved in mitosis	GO	0
GO:0044839	Cell cycle G2/M phase transition	GO	0
GO:0044843	Cell cycle G1/S phase transition	GO	0
GO:1901987	Regulation of cell cycle phase transition	GO	0
GO:0010948	Negative regulation of cell cycle process	GO	0
GO:0045787	Positive regulation of cell cycle	GO	0
GO:0007050	Cell cycle arrest	GO	7.77 × 10^−4^
hsa04115	p53 signaling pathway	KEGG	0.002014026
GO:0006260	DNA replication	GO	0
hsa03030	DNA replication	KEGG	0
hsa00240	Pyrimidine metabolism	KEGG	5.67 × 10^−10^
hsa00230	Purine metabolism	KEGG	4.39 × 10^−5^
GO:0042769	DNA damage response, detection of DNA damage	GO	1.88 × 10^−6^
GO:0006310	DNA recombination	GO	0
GO:0006302	Double-strand break repair	GO	0
GO:0036297	Inter-strand cross-link repair	GO	1.35 × 10^−12^
hsa03430	Mismatch repair	KEGG	3.11 × 10^−11^
hsa03440	Homologous recombination	KEGG	3.11 × 10^−11^
GO:0006284	Base excision repair	GO	6.54 × 10^−6^
hsa03410	Base excision repair	KEGG	1.91 × 10^−7^
GO:0006298	Mismatch repair	GO	4.02 × 10^−4^
GO:0006289	Nucleotide excision repair	GO	5.10 × 10^−4^
hsa03420	Nucleotide excision repair	KEGG	2.91 × 10^−6^
GO:0006333	Chromatin assembly or disassembly	GO	0
GO:0007051	Spindle organization	GO	0
GO:0071103	DNA conformation change	GO	0
GO:0007059	Chromosome segregation	GO	0
GO:0061641	CENP-A containing chromatin organization	GO	0
GO:0048285	Organelle fission	GO	0
GO:0051052	Regulation of DNA metabolic process	GO	0
GO:0071824	Protein-DNA complex subunit organization	GO	0
hsa03013	RNA transport	KEGG	5.66 × 10^−5^
hsa03008	Ribosome biogenesis in eukaryotes	KEGG	4.13 × 10^−5^
hsa04114	Oocyte meiosis	KEGG	0.001082137
hsa04914	Progesterone-mediated oocyte maturation	KEGG	0.008441906
hsa05322	Systemic lupus erythematosus	KEGG	0
hsa05203	Viral carcinogenesis	KEGG	1.46 × 10^−11^
hsa05206	MicroRNAs in cancer	KEGG	0.008110817
hsa03460	Fanconi anemia pathway	KEGG	7.71 × 10^−11^
hsa05166	Human T-cell leukemia virus 1 infection	KEGG	5.47 × 10^−4^
hsa04217	Necroptosis	KEGG	1.19 × 10^−4^
hsa04218	Cellular senescence	KEGG	0.0014615

**Table 4:** Enriched gene-targeting transcription factors for down-regulated genes after meta-analysis.

Gene Set	Gene Symbol	Gene Name	FDR
**Down-Regulated Genes** 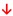			
E2F_Q3_01;E2F_Q4_01; E2F_Q6_01;E2F1_Q4_01	TFDP1	Transcription factor Dp-1	0
E2F1_Q3;E2F1_Q6; E2F1_Q6_01	E2F1	E2F transcription factor 1	0
E2F1DP1_01	E2F1;TFDP1	E2F transcription factor 1; transcription factor Dp-1	0
E2F1DP1RB_01	E2F1;TFDP1; RB1	E2F transcription factor 1; transcription factor Dp-1; RB transcriptional corepressor 1	0
E2F1DP2_01	TFDP2	Transcription factor Dp-2	0
E2F4DP1_01	E2F4;TFDP1	E2F transcription factor 4; transcription factor Dp-1	0
E2F4DP2_01	E2F4;TFDP2	E2F transcription factor 4; transcription factor Dp-2	0
E2F1_Q4;	E2F1	E2F transcription factor 1	9.80 × 10^−12^
E2F1_Q3_01	E2F1	E2F transcription factor 1	4.39 × 10^−5^

## Supplementary Materials

Supplementary materials can be found at https://www.mdpi.com/1422-0067/21/6/1938/s1. Table S1: Statistically significant DEGs (Adj.*p*-value<0.05) derived from DGEA of RNA-Seq data from human iPSC-Derived Cardiomyocytes after X-ray irradiation, using DESeq2. The experiment consists of 3 control samples and 3 irradiated with 5 Gy X-ray radiation for 48 hours [Bioproject: PRJNA421022]; Table S2: Statistically significant DEGs (Adj.*p*-value<0.05) derived from DGEA of RNA-Seq data from Primary Human Lung Fibroblasts (IMR90) after X-ray irradiation, using DESeq2. The experiment consists of 3 control samples and 3 irradiated with 2 Gy X-ray radiation for 24 hours [Bioproject: PRJNA436999]; Table S3: Statistically significant DEGs (Adj.*p*-value<0.05) derived from DGEA of RNA-Seq data from Human Lens Epithelial Cells after X-ray irradiation, using DESeq2. The experiment consists of 5 control samples and 5 irradiated with 5 Gy X-ray radiation for 20 hours [Bioproject: PRJNA494581]; Table S4: Statistically significant DEGs derived from a meta-analysis comparing DESeq2 outputs from irradiated and control samples of three datasets [PRJNA421022, PRJNA436999, PRJNA494581].

## Abbreviations

BER	Base Excision Repair
DDR	DNA Damage Response
DEGs	Differentially Expressed Genes
DGE	Differential Gene Expression
DGEA	Differential Gene Expression Analysis
DSBs	Double Strand Breaks
ENA	European Nucleotide Archive
FDR	False Discovery Rate
GSEA	Gene Set Enrichment Analysis
GO	Gene Ontology
HR	Homology-dependent Recombination
IR	Ionizing Radiation
LET	Linear Energy Transfer
Log_2_FC	Log_2_ Fold Change
MMR	Mismatch Repair
NER	Nucleotide Excision Repair
NGS	Next-Generation Sequencing
NHEJ	Non-Homologous End Joining
ORA	Over-Representation Analysis
PPI	Protein to Protein Interaction
Q	PHRED Score
RBE	Relative Biological Effectiveness
RNA-Seq	RNA-Sequencing
RNS	Reactive Nitrogen Species
ROS	Reactive Oxygen Species
RT	Radiation Therapy
SSBs	Single-Strand Breaks
WebGestalt	WEB-based Gene Set Analysis Toolkit

## Appendix A

**Table A1:** Specific arguments used during the analysis. Unless explicitly specified otherwise, default parameters were used.

**Trim Galore!**	trim_galore --cores 4 --illumina -q 20 --phred33 –paired --fastqc <fastq files>
**Salmon**	salmon quant -i salmon_index --libType A -1 <forward_1.fq.gz> -2 <reverse_1.fq.gz> --gcBias --validateMappings -o <transcripts_directory>
**WebGestalt**	**Basic Parameters:**
	Organism of Interest: Homo sapiens Method of Interest: ORA Functional Database: geneontology + (Biological Process: no redundant), pathway + (KEGG), Network + (Transcription Factor target) Gene List: Select Gene ID Type: EnsEMBL Gene IDReference Gene List: Upload: Mappings per study: EnsEMBL Gene ID
	**Advanced Parameters**
	minimum number of genes for category: 2 Multiple Test Adjustment: Benjamini-Hochberg Significance level: FDR (0.05) Number of categories visualized in the report: 100
**STRING**	**Basic Settings:**
	meaning of network edges: confidence active interaction sources: textmining minimum required interaction score: high confidence (0.7)
	**Advanced Settings:**
	hide disconnected nodes in the network disable structure previous inside network bubbles

## References

[1] UNSCEAR (United Nations Scientific Committee on the Effects of Atomic Radiation) (2012). Biological Mechanisms of Radiation Actions at Low Doses.

[2] Leuraud K., Richardson D.B., Cardis E., Daniels R.D., Gillies M., O’Hagan J.A., Hamra G.B., Haylock R., Laurier D., Moissonnier M. (2015). Ionising radiation and risk of death from leukaemia and lymphoma in radiation-monitored workers (INWORKS): An international cohort study. Lancet. Haematol..

[3] Ray M., Yunis R., Chen X., Rocke D.M. (2012). Comparison of low and high dose ionising radiation using topological analysis of gene coexpression networks. Bmc Genom..

[4] Bragg W.H., Kleeman R. (1904). LXXIV. On the ionization curves of radium. Lond. Edinb. Dublin Philos. Mag. J. Sci..

[5] Baskar R., Dai J., Wenlong N., Yeo R., Yeoh K.W. (2014). Biological response of cancer cells to radiation treatment. Front. Mol. Biosci..

[6] Mehta S.R., Suhag V., Semwal M., Sharma N. (2010). Radiotherapy: Basic Concepts and Recent Advances. Med. J. Armed. Forces India.

[7] Kjellberg R.N., Hanamura T., Davis K.R., Lyons S.L., Adams R.D. (1983). Bragg-Peak Proton-Beam Therapy for Arteriovenous Malformations of the Brain. N. Engl. J. Med..

[8] Bernier J., Hall E.J., Giaccia A. (2004). Radiation oncology: A century of achievements. Nat. Rev. Cancer.

[9] Georgakilas A.G. (2015). Bystander and non-targeted effects: A unifying model from ionizing radiation to cancer. Cancer Lett..

[10] Riley P.A. (1994). Free radicals in biology: Oxidative stress and the effects of ionizing radiation. Int. J. Radiat. Biol..

[11] Wallace S.S. (1998). Enzymatic processing of radiation-induced free radical damage in DNA. Radiat. Res..

[12] Mavragani I.V., Nikitaki Z., Souli M.P., Aziz A., Nowsheen S., Aziz K., Rogakou E., Georgakilas A.G. (2017). Complex DNA Damage: A Route to Radiation-Induced Genomic Instability and Carcinogenesis. Cancers.

[13] Georgakilas A.G. (2008). Processing of DNA damage clusters in human cells: Current status of knowledge. Mol. Biosyst..

[14] Nikitaki Z., Hellweg C.E., Georgakilas A.G., Ravanat J.L. (2015). Stress-induced DNA damage biomarkers: Applications and limitations. Front. Chem..

[15] Ogrunc M., Di Micco R., Liontos M., Bombardelli L., Mione M., Fumagalli M., Gorgoulis V.G., d’Adda di Fagagna F. (2014). Oncogene-induced reactive oxygen species fuel hyperproliferation and DNA damage response activation. Cell Death Differ..

[16] Saini D., Shelke S., Mani Vannan A., Toprani S., Jain V., Das B., Seshadri M. (2012). Transcription profile of DNA damage response genes at G(0) lymphocytes exposed to gamma radiation. Mol. Cell. Biochem..

[17] Nikitaki Z., Pavlopoulou A., Hola M., Dona M., Michalopoulos I., Balestrazzi A., Angelis K.J., Georgakilas A.G. (2017). Bridging Plant and Human Radiation Response and DNA Repair through an In Silico Approach. Cancers.

[18] Knijnenburg T.A., Wang L., Zimmermann M.T., Chambwe N., Gao G.F., Cherniack A.D., Fan H., Shen H., Way G.P., Greene C.S. (2018). Genomic and Molecular Landscape of DNA Damage Repair Deficiency across The Cancer Genome Atlas. Cell Rep..

[19] Friedberg E.C. (2016). A history of the DNA repair and mutagenesis field: The discovery of base excision repair. Dna Repair.

[20] Pan M.R., Li K., Lin S.Y., Hung W.C. (2016). Connecting the Dots: From DNA Damage and Repair to Aging. Int. J. Mol. Sci..

[21] Georgakilas A.G., Pavlopoulou A., Louka M., Nikitaki Z., Vorgias C.E., Bagos P.G., Michalopoulos I. (2015). Emerging molecular networks common in ionizing radiation, immune and inflammatory responses by employing bioinformatics approaches. Cancer Lett..

[22] Nakad R., Schumacher B. (2016). DNA Damage Response and Immune Defense: Links and Mechanisms. Front. Genet..

[23] Hatzi V.I., Laskaratou D.A., Mavragani I.V., Nikitaki Z., Mangelis A., Panayiotidis M.I., Pantelias G.E., Terzoudi G.I., Georgakilas A.G. (2015). Non-targeted radiation effects in vivo: A critical glance of the future in radiobiology. Cancer Lett..

[24] Zhao S., Fung-Leung W.P., Bittner A., Ngo K., Liu X. (2014). Comparison of RNA-Seq and microarray in transcriptome profiling of activated T cells. Plos ONE.

[25] Hrdlickova R., Toloue M., Tian B. (2017). RNA-Seq methods for transcriptome analysis. Wiley Interdiscip Rev. Rna..

[26] Romero J.P., Ortiz-Estevez M., Muniategui A., Carrancio S., de Miguel F.J., Carazo F., Montuenga L.M., Loos R., Pio R., Trotter M.W.B. (2018). Comparison of RNA-seq and microarray platforms for splice event detection using a cross-platform algorithm. Bmc Genom..

[27] Barrett T., Wilhite S.E., Ledoux P., Evangelista C., Kim I.F., Tomashevsky M., Marshall K.A., Phillippy K.H., Sherman P.M., Holko M. (2013). NCBI GEO: Archive for functional genomics data sets--update. Nucleic Acids Res..

[28] Chauhan V., Rowan-Carroll A., Gagne R., Kuo B., Williams A., Yauk C.L. (2019). The use of in vitro transcriptional data to identify thresholds of effects in a human lens epithelial cell-line exposed to ionizing radiation. Int. J. Radiat. Biol..

[29] Shimada M., Tsukada K., Kagawa N., Matsumoto Y. (2019). Reprogramming and differentiation-dependent transcriptional alteration of DNA damage response and apoptosis genes in human induced pluripotent stem cells. J. Radiat. Res..

[30] Becker B.V., Majewski M., Abend M., Palnek A., Nestler K., Port M., Ullmann R. (2018). Gene expression changes in human iPSC-derived cardiomyocytes after X-ray irradiation. Int. J. Radiat. Biol..

[31] Becker B.V., Kaatsch L., Obermair R., Schrock G., Port M., Ullmann R. (2019). X-ray irradiation induces subtle changes in the genome-wide distribution of DNA hydroxymethylation with opposing trends in genic and intergenic regions. Epigenetics.

[32] Ewing B., Hillier L., Wendl M.C., Green P. (1998). Base-calling of automated sequencer traces using phred. I. Accuracy assessment. Genome Res..

[33] Ewing B., Green P. (1998). Base-calling of automated sequencer traces using phred. II. Error probabilities. Genome Res..

[34] Schurch N.J., Schofield P., Gierlinski M., Cole C., Sherstnev A., Singh V., Wrobel N., Gharbi K., Simpson G.G., Owen-Hughes T. (2016). How many biological replicates are needed in an RNA-seq experiment and which differential expression tool should you use?. RNA.

[35] (2015). Gene Ontology Consortium. Gene Ontology Consortium: Going forward. Nucleic Acids Res..

[36] Kanehisa M., Goto S. (2000). KEGG: Kyoto encyclopedia of genes and genomes. Nucleic Acids Res..

[37] Conesa A., Madrigal P., Tarazona S., Gomez-Cabrero D., Cervera A., McPherson A., Szczesniak M.W., Gaffney D.J., Elo L.L., Zhang X. (2016). A survey of best practices for RNA-seq data analysis. Genome Biol..

[38] Del Fabbro C., Scalabrin S., Morgante M., Giorgi F.M. (2013). An extensive evaluation of read trimming effects on Illumina NGS data analysis. PLoS ONE.

[39] Liao Y., Shi W. (2019). Read trimming is not required for mapping and quantification of RNA-seq reads. bioRxiv.

[40] Williams C.R., Baccarella A., Parrish J.Z., Kim C.C. (2016). Trimming of sequence reads alters RNA-Seq gene expression estimates. Bmc Bioinform..

[41] Cock P.J., Fields C.J., Goto N., Heuer M.L., Rice P.M. (2010). The Sanger FASTQ file format for sequences with quality scores, and the Solexa/Illumina FASTQ variants. Nucleic Acids Res..

[42] Kukurba K.R., Montgomery S.B. (2015). RNA Sequencing and Analysis. Cold Spring Harb Protoc.

[43] Li H., Durbin R. (2010). Fast and accurate long-read alignment with Burrows-Wheeler transform. Bioinformatics.

[44] Krizanovic K., Echchiki A., Roux J., Sikic M. (2018). Evaluation of tools for long read RNA-seq splice-aware alignment. Bioinformatics.

[45] Kim D., Paggi J.M., Park C., Bennett C., Salzberg S.L. (2019). Graph-based genome alignment and genotyping with HISAT2 and HISAT-genotype. Nat. Biotechnol..

[46] Kodama Y., Shumway M., Leinonen R., International Nucleotide Sequence Database C. (2012). The Sequence Read Archive: Explosive growth of sequencing data. Nucleic Acids Res..

[47] Patro R., Duggal G., Love M.I., Irizarry R.A., Kingsford C. (2017). Salmon provides fast and bias-aware quantification of transcript expression. Nat Methods.

[48] Love M.I., Huber W., Anders S. (2014). Moderated estimation of fold change and dispersion for RNA-seq data with DESeq2. Genome Biol..

[49] Belinky F., Nativ N., Stelzer G., Zimmerman S., Iny Stein T., Safran M., Lancet D. (2015). PathCards: Multi-source consolidation of human biological pathways. J. Biol. Databases Curation.

[50] Harris S.L., Levine A.J. (2005). The p53 pathway: Positive and negative feedback loops. Oncogene.

[51] Georgakilas A.G., Martin O.A., Bonner W.M. (2017). p21: A Two-Faced Genome Guardian. Trends Mol. Med..

[52] Pateras I.S., Havaki S., Nikitopoulou X., Vougas K., Townsend P.A., Panayiotidis M.I., Georgakilas A.G., Gorgoulis V.G. (2015). The DNA damage response and immune signaling alliance: Is it good or bad? Nature decides when and where. Pharmacol. Ther..

[53] Bertoli C., Skotheim J.M., de Bruin R.A. (2013). Control of cell cycle transcription during G1 and S phases. Nat. Rev. Mol. Cell Biol..

[54] Shu K.X., Li B., Wu L.X. (2007). The p53 network: p53 and its downstream genes. Colloids Surf. Biointerfaces.

[55] O’Leary N.A., Wright M.W., Brister J.R., Ciufo S., Haddad D., McVeigh R., Rajput B., Robbertse B., Smith-White B., Ako-Adjei D. (2016). Reference sequence (RefSeq) database at NCBI: Current status, taxonomic expansion, and functional annotation. Nucleic Acids Res..

[56] Christmann M., Kaina B. (2013). Transcriptional regulation of human DNA repair genes following genotoxic stress: Trigger mechanisms, inducible responses and genotoxic adaptation. Nucleic Acids Res..

[57] Collin G., Huna A., Warnier M., Flaman J.M., Bernard D. (2018). Transcriptional repression of DNA repair genes is a hallmark and a cause of cellular senescence. Cell Death Dis..

[58] Kanehisa M., Sato Y., Furumichi M., Morishima K., Tanabe M. (2019). New approach for understanding genome variations in KEGG. Nucleic Acids Res..

[59] Greene M.W. (2012). Circadian rhythms and tumor growth. Cancer Lett..

[60] Forssell-Aronsson E., Quinlan R.A. (2017). The Impact of Circadian Rhythms on Medical Imaging and Radiotherapy Regimes for the Paediatric Patient. Radiat. Prot. Dosim..

[61] Jim H.S., Lin H.Y., Tyrer J.P., Lawrenson K., Dennis J., Chornokur G., Chen Z., Chen A.Y., Permuth-Wey J., Aben K.K. (2015). Common Genetic Variation in Circadian Rhythm Genes and Risk of Epithelial Ovarian Cancer (EOC). J. Genet. Genome Res..

[62] Kizaki T., Sato S., Shirato K., Sakurai T., Ogasawara J., Izawa T., Ohira Y., Suzuki K., Ohno H. (2015). Effect of Circadian Rhythm on Clinical and Pathophysiological Conditions and Inflammation. Crit. Rev. Immunol..

[63] Silvester N., Alako B., Amid C., Cerdeno-Tarraga A., Clarke L., Cleland I., Harrison P.W., Jayathilaka S., Kay S., Keane T. (2018). The European Nucleotide Archive in 2017. Nucleic Acids Res..

[64] Andrews S. (2010). FastQC: A Quality Control Tool for High Throughput Sequence Data. https://www.bioinformatics.babraham.ac.uk/projects/fastqc/.

[65] Ewels P., Magnusson M., Lundin S., Kaller M. (2016). MultiQC: Summarize analysis results for multiple tools and samples in a single report. Bioinformatics.

[66] Krueger F. (2015). Trim Galore!: A Wrapper Tool around Cutadapt and Cutadapt and FastQC to Consistently Apply Quality and Adapter Trimming to FastQ Files, with Some Extra Functionality for MspI-Digested RRBS-Type (Reduced Representation Bisufite-Seq) Libraries. https://www.bioinformatics.babraham.ac.uk/projects/trim_galore/.

[67] Martin M. (2011). Cutadapt removes adapter sequences from high-throughput sequencing reads. Embnet. J..

[68] Srivastava A., Malik L., Sarkar H., Zakeri M., Almodaresi F., Soneson C., Love M.I., Kingsford C., Patro R. (2019). Alignment and mapping methodology influence transcript abundance estimation. bioRxiv.

[69] Huber W., Carey V.J., Gentleman R., Anders S., Carlson M., Carvalho B.S., Bravo H.C., Davis S., Gatto L., Girke T. (2015). Orchestrating high-throughput genomic analysis with Bioconductor. Nat. Methods..

[70] Gentleman R.C., Carey V.J., Bates D.M., Bolstad B., Dettling M., Dudoit S., Ellis B., Gautier L., Ge Y., Gentry J. (2004). Bioconductor: Open software development for computational biology and bioinformatics. Genome Biol..

[71] R Core Team (2019). R: A Language and Environment for Statistical Computing. R Found. Stat. Comput..

[72] RStudio Team (2019). RStudio: Integrated Development for R. RstudioInc..

[73] Love M.I., Soneson C., Charlotte H., Johnson L., Pierce N., Shepherd L., Morgan M., Patro R. (2019). Tximeta: Reference sequence checksums for provenance identification in RNA-seq. PLOS Comput. Biol..

[74] Soneson C., Love M.I., Robinson M.D. (2015). Differential analyses for RNA-seq: Transcript-level estimates improve gene-level inferences. F1000Res.

[75] Benjamini Y., Hochberg Y. (1995). Controlling the False Discovery Rate: A Practical and Powerful Approach to Multiple Testing. J. R. Stat. Soc. Ser. B (Methodol.).

[76] Carlson M. (2019). org.Hs.eg.db: Genome Wide Annotation for Human. https://bioconductor.org/packages/release/data/annotation/html/org.Hs.eg.db.html.

[77] Yates B., Braschi B., Gray K.A., Seal R.L., Tweedie S., Bruford E.A. (2017). Genenames.org: The HGNC and VGNC resources in 2017. Nucleic Acids Res..

[78] Stouffer S.A., Suchman E.A., DeVinney L.C., Star S.A., Williams R.M. (1949). The American Soldier: Adjustment During Army Life.

[79] Mosteller F., Bush R.R., Lindzey G. (1954). Selected quantitative techniques. Handbook of social psychology: Vol. 1. Theory and Method.

[80] Liao Y., Wang J., Jaehnig E.J., Shi Z., Zhang B. (2019). WebGestalt 2019: Gene set analysis toolkit with revamped UIs and APIs. Nucleic Acids Res..

[81] Khatri P., Sirota M., Butte A.J. (2012). Ten years of pathway analysis: Current approaches and outstanding challenges. Plos Comput. Biol..

[82] Szklarczyk D., Gable A.L., Lyon D., Junge A., Wyder S., Huerta-Cepas J., Simonovic M., Doncheva N.T., Morris J.H., Bork P. (2019). STRING v11: Protein-protein association networks with increased coverage, supporting functional discovery in genome-wide experimental datasets. Nucleic Acids Res..

