# Supplementary material for: Effects of High-Dose Ionizing Radiation in Human Gene Expression: A Meta-Analysis
Source: Int J Mol Sci. 2020 Mar 12;21(6):1938. doi: 10.3390/ijms21061938 (PMC7139561; doi:10.3390/ijms21061938)
Supplement: Supplementary file 1 [file ijms-21-01938-s001.pdf]

**Supplementary Table S1.** Statistically significant DEGs (Adj.*p*-value<0.05) derived from DGEA of RNA-Seq data from human iPSC-Derived Cardiomyocytes after X-ray irradiation, using DESeq2. The experiment consists of 3 control samples and 3 irradiated with 5 Gy X-ray radiation for 48 hours [Bioproject: PRJNA421022].

| Ensembl ID | Gene Symbol | Gene Description | Adj. *p*-Value |
|---|---|---|---|
| | | **Up-Regulated Genes↑** | |
| ENSG00000196826 | AC008758.1 | Novel zinc finger protein | 1.48E-13 |
| ENSG00000163071 | SPATA18 | Spermatogenesis associated 18 | 2.89E-13 |
| ENSG00000173535 | TNFRSF10C | TNF receptor superfamily member 10c | 2.50E-11 |
| ENSG00000124762 | CDKN1A | Cyclin dependent kinase inhibitor 1A | 3.30E-09 |
| ENSG00000128271 | ADORA2A | Adenosine A2a receptor | 7.41E-09 |
| ENSG00000161513 | FDXR | Ferredoxin reductase | 3.25E-07 |
| ENSG00000198211 | AC092143.1 | Novel protein (MC1R-TUBB3 readthrough) | 1.03E-06 |
| ENSG00000270181 | BIVM-ERCC5 | BIVM-ERCC5 readthrough | 4.30E-06 |
| ENSG00000177106 | EPS8L2 | EPS8 like 2 | 6.65E-06 |
| ENSG00000080546 | SESN1 | Sestrin 1 | 7.62E-06 |
| ENSG00000256664 | AC025423.2 | Ribosomal L24 domain containing 1 (RSL24D1) pseudogene | 9.78E-06 |
| ENSG00000102935 | ZNF423 | Zinc finger protein 423 | 1.66E-05 |
| ENSG00000135679 | MDM2 | MDM2 proto-oncogene | 2.63E-05 |
| ENSG00000174307 | PHLDA3 | Pleckstrin homology like domain family A member 3 | 2.92E-05 |
| ENSG00000225968 | ELFN1 | Extracellular leucine rich repeat and fibronectin type III domain containing 1 | 3.06E-05 |
| ENSG00000244694 | PTCHD4 | Patched domain containing 4 | 8.94E-05 |
| ENSG00000131398 | KCNC3 | Potassium voltage-gated channel subfamily C member 3 | 9.03E-05 |
| ENSG00000181026 | AEN | Apoptosis enhancing nuclease | 0.000143 |
| ENSG00000069188 | SDK2 | Sidekick cell adhesion molecule 2 | 0.000209 |
| ENSG00000185088 | RPS27L | Ribosomal protein S27 like | 0.000357 |
| ENSG00000166923 | GREM1 | Gremlin 1, DAN family BMP antagonist | 0.000363 |
| ENSG00000196562 | SULF2 | Sulfatase 2 | 0.000365 |
| ENSG00000130766 | SESN2 | Sestrin 2 | 0.000467 |
| ENSG00000131080 | EDA2R | Ectodysplasin A2 receptor | 0.000562 |
| ENSG00000130517 | PGPEP1 | Pyroglutamyl-peptidase I | 0.000565 |
| ENSG00000185559 | DLK1 | Delta like non-canonical Notch ligand 1 | 0.000604 |
| ENSG00000110900 | TSPAN11 | Tetraspanin 11 | 0.001129 |
| ENSG00000118971 | CCND2 | Cyclin D2 | 0.001327 |
| ENSG00000064201 | TSPAN32 | Tetraspanin 32 | 0.001755 |
| ENSG00000172667 | ZMAT3 | Zinc finger matrin-type 3 | 0.001871 |
| ENSG00000135709 | KIAA0513 | Kiaa0513 | 0.001996 |
| ENSG00000198753 | PLXNB3 | Plexin B3 | 0.002337 |
| ENSG00000026103 | FAS | Fas cell surface death receptor | 0.00256 |
| ENSG00000112249 | ASCC3 | Activating signal cointegrator 1 complex subunit 3 | 0.002767 |
| ENSG00000177076 | ACER2 | Alkaline ceramidase 2 | 0.002961 |
| ENSG00000130513 | GDF15 | Growth differentiation factor 15 | 0.003049 |
| ENSG00000078237 | TIGAR | TP53 induced glycolysis regulatory phosphatase | 0.003505 |
| ENSG00000156097 | GPR61 | G protein-coupled receptor 61 | 0.004582 |
| ENSG00000251357 | AP000350.4 | Novel protein | 0.00497 |
| ENSG00000196557 | CACNA1H | Calcium voltage-gated channel subunit alpha1 H | 0.005111 |
| ENSG00000105290 | APLP1 | Amyloid beta precursor like protein 1 | 0.006093 |

| | | | |
|---|---|---|---|
| ENSG00000244509 | APOBEC3C | Apolipoprotein B mrna editing enzyme catalytic subunit 3C | 0.006844 |
| ENSG00000137103 | TMEM8B | Transmembrane protein 8B | 0.006913 |
| ENSG00000110665 | C11orf21 | Chromosome 11 open reading frame 21 | 0.008327 |
| ENSG00000167971 | CASKIN1 | CASK interacting protein 1 | 0.008441 |
| ENSG00000105327 | BBC3 | BCL2 binding component 3 | 0.010488 |
| ENSG00000087088 | BAX | BCL2 associated X, apoptosis regulator | 0.011202 |
| ENSG00000170271 | FAXDC2 | Fatty acid hydroxylase domain containing 2 | 0.012676 |
| ENSG00000125746 | EML2 | EMAP like 2 | 0.013186 |
| ENSG00000160685 | ZBTB7B | Zinc finger and BTB domain containing 7B | 0.014514 |
| ENSG00000188322 | SBK1 | SH3 domain binding kinase 1 | 0.014946 |
| ENSG00000103227 | LMF1 | Lipase maturation factor 1 | 0.014951 |
| ENSG00000285188 | AC008397.2 | Camp-specific 3',5'-cyclic phosphodiesterase 4C | 0.018796 |
| ENSG00000178947 | SMIM10L2A | Small integral membrane protein 10 like 2A | 0.019552 |
| ENSG00000238098 | ABCA17P | ATP binding cassette subfamily A member 17, pseudogene | 0.020843 |
| ENSG00000262481 | TMEM256-PLSCR3 | TMEM256-PLSCR3 readthrough (NMD candidate) | 0.021436 |
| ENSG00000162643 | WDR63 | WD repeat domain 63 | 0.021956 |
| ENSG00000136378 | ADAMTS7 | ADAM metallopeptidase with thrombospondin type 1 motif 7 | 0.023072 |
| ENSG00000158113 | LRRC43 | Leucine rich repeat containing 43 | 0.024473 |
| ENSG00000170835 | CEL | Carboxyl ester lipase | 0.025568 |
| ENSG00000138792 | ENPEP | Glutamyl aminopeptidase | 0.025659 |
| ENSG00000142046 | TMEM91 | Transmembrane protein 91 | 0.028467 |
| ENSG00000185650 | ZFP36L1 | ZFP36 ring finger protein like 1 | 0.030315 |
| ENSG00000172831 | CES2 | Carboxylesterase 2 | 0.031055 |
| ENSG00000117016 | RIMS3 | Regulating synaptic membrane exocytosis 3 | 0.031222 |
| ENSG00000154767 | XPC | XPC complex subunit, DNA damage recognition and repair factor | 0.031234 |
| ENSG00000100100 | PIK3IP1 | Phosphoinositide-3-kinase interacting protein 1 | 0.033966 |
| ENSG00000102032 | RENBP | Renin binding protein | 0.034619 |
| ENSG00000013364 | MVP | Major vault protein | 0.037031 |
| ENSG00000029534 | ANK1 | Ankyrin 1 | 0.04171 |
| ENSG00000184481 | FOXO4 | Forkhead box O4 | 0.046386 |
| ENSG00000042445 | RETSAT | Retinol saturase | 0.046776 |
| ENSG00000174938 | SEZ6L2 | Seizure related 6 homolog like 2 | 0.047378 |
| **Down-Regulated Genes↓** | | | |
| ENSG00000145386 | CCNA2 | Cyclin A2 | 1.01E-161 |
| ENSG00000170312 | CDK1 | Cyclin dependent kinase 1 | 1.30E-148 |
| ENSG00000148773 | MKI67 | Marker of proliferation Ki-67 | 1.92E-144 |
| ENSG00000024526 | DEPDC1 | DEP domain containing 1 | 6.73E-135 |
| ENSG00000203811 | H3C14 | H3 clustered histone 14 | 2.21E-134 |
| ENSG00000166851 | PLK1 | Polo like kinase 1 | 4.21E-134 |
| ENSG00000171848 | RRM2 | Ribonucleotide reductase regulatory subunit M2 | 4.33E-130 |
| ENSG00000126787 | DLGAP5 | DLG associated protein 5 | 2.93E-121 |
| ENSG00000175063 | UBE2C | Ubiquitin conjugating enzyme E2 C | 6.58E-120 |
| ENSG00000112984 | KIF20A | Kinesin family member 20A | 3.08E-119 |
| ENSG00000168078 | PBK | PDZ binding kinase | 5.35E-119 |
| ENSG00000157456 | CCNB2 | Cyclin B2 | 1.21E-112 |
| ENSG00000183856 | IQGAP3 | IQ motif containing gtpase activating protein 3 | 1.84E-111 |

| | | | |
|---|---|---|---|
| ENSG00000090889 | KIF4A | Kinesin family member 4A | 2.97E-111 |
| ENSG00000101447 | FAM83D | Family with sequence similarity 83 member D | 1.82E-110 |
| ENSG00000286522 | H3C2 | H3 clustered histone 2 | 1.55E-109 |
| ENSG00000142945 | KIF2C | Kinesin family member 2C | 7.68E-104 |
| ENSG00000117724 | CENPF | Centromere protein F | 5.74E-100 |
| ENSG00000101057 | MYBL2 | MYB proto-oncogene like 2 | 8.03E-100 |
| ENSG00000169607 | CKAP2L | Cytoskeleton associated protein 2 like | 1.48E-98 |
| ENSG00000094804 | CDC6 | Cell division cycle 6 | 1.90E-98 |
| ENSG00000198826 | ARHGAP11A | Rho gtpase activating protein 11A | 2.70E-98 |
| ENSG00000088325 | TPX2 | TPX2 microtubule nucleation factor | 3.63E-98 |
| ENSG00000131747 | TOP2A | DNA topoisomerase II alpha | 1.29E-97 |
| ENSG00000112742 | TTK | TTK protein kinase | 1.26E-96 |
| ENSG00000121152 | NCAPH | Non-SMC condensin I complex subunit H | 1.44E-95 |
| ENSG00000134690 | CDCA8 | Cell division cycle associated 8 | 8.76E-94 |
| ENSG00000189057 | FAM111B | Family with sequence similarity 111 member B | 1.39E-91 |
| ENSG00000198901 | PRC1 | Protein regulator of cytokinesis 1 | 2.25E-90 |
| ENSG00000066279 | ASPM | Abnormal spindle microtubule assembly | 6.02E-89 |
| ENSG00000117650 | NEK2 | NIMA related kinase 2 | 1.68E-88 |
| ENSG00000118193 | KIF14 | Kinesin family member 14 | 1.79E-86 |
| ENSG00000109805 | NCAPG | Non-SMC condensin I complex subunit G | 2.49E-86 |
| ENSG00000167900 | TK1 | Thymidine kinase 1 | 2.24E-85 |
| ENSG00000138180 | CEP55 | Centrosomal protein 55 | 2.41E-85 |
| ENSG00000164611 | PTTG1 | PTTG1 regulator of sister chromatid separation, securin | 4.61E-85 |
| ENSG00000143476 | DTL | Denticleless E3 ubiquitin protein ligase homolog | 1.95E-84 |
| ENSG00000076382 | SPAG5 | Sperm associated antigen 5 | 2.24E-84 |
| ENSG00000161800 | RACGAP1 | Rac gtpase activating protein 1 | 6.31E-82 |
| ENSG00000134057 | CCNB1 | Cyclin B1 | 6.31E-82 |
| ENSG00000075218 | GTSE1 | G2 and S-phase expressed 1 | 1.75E-79 |
| ENSG00000111665 | CDCA3 | Cell division cycle associated 3 | 3.42E-79 |
| ENSG00000273983 | H3C8 | H3 clustered histone 8 | 1.79E-76 |
| ENSG00000137804 | NUSAP1 | Nucleolar and spindle associated protein 1 | 1.85E-75 |
| ENSG00000122966 | CIT | Citron rho-interacting serine/threonine kinase | 1.02E-72 |
| ENSG00000129195 | PIMREG | PICALM interacting mitotic regulator | 4.17E-70 |
| ENSG00000138160 | KIF11 | Kinesin family member 11 | 2.43E-69 |
| ENSG00000138778 | CENPE | Centromere protein E | 2.80E-69 |
| ENSG00000065328 | MCM10 | Minichromosome maintenance 10 replication initiation factor | 4.13E-67 |
| ENSG00000139354 | GAS2L3 | Growth arrest specific 2 like 3 | 3.90E-65 |
| ENSG00000068489 | PRR11 | Proline rich 11 | 1.18E-64 |
| ENSG00000164109 | MAD2L1 | Mitotic arrest deficient 2 like 1 | 1.18E-64 |
| ENSG00000105011 | ASF1B | Anti-silencing function 1B histone chaperone | 3.04E-64 |
| ENSG00000129810 | SGO1 | Shugoshin 1 | 1.03E-62 |
| ENSG00000143228 | NUF2 | NUF2 component of NDC80 kinetochore complex | 2.28E-62 |
| ENSG00000169679 | BUB1 | BUB1 mitotic checkpoint serine/threonine kinase | 1.23E-61 |
| ENSG00000186871 | ERCC6L | ERCC excision repair 6 like, spindle assembly checkpoint helicase | 2.30E-59 |
| ENSG00000167513 | CDT1 | Chromatin licensing and DNA replication factor 1 | 7.22E-59 |
| ENSG00000171241 | SHCBP1 | SHC binding and spindle associated 1 | 1.72E-58 |
| ENSG00000108106 | UBE2S | Ubiquitin conjugating enzyme E2 S | 6.65E-57 |
| ENSG00000175305 | CCNE2 | Cyclin E2 | 9.61E-57 |
| ENSG00000186193 | SAPCD2 | Suppressor APC domain containing 2 | 2.49E-55 |

| | | | |
|---|---|---|---|
| ENSG00000115163 | CENPA | Centromere protein A | 1.01E-53 |
| ENSG00000092853 | CLSPN | Claspin | 5.80E-53 |
| ENSG00000100297 | MCM5 | Minichromosome maintenance complex component 5 | 1.42E-52 |
| ENSG00000121621 | KIF18A | Kinesin family member 18A | 1.46E-51 |
| ENSG00000122952 | ZWINT | ZW10 interacting kinetochore protein | 3.76E-51 |
| ENSG00000158402 | CDC25C | Cell division cycle 25C | 1.07E-50 |
| ENSG00000111247 | RAD51AP1 | RAD51 associated protein 1 | 1.68E-50 |
| ENSG00000135451 | TROAP | Trophinin associated protein | 1.73E-50 |
| ENSG00000186185 | KIF18B | Kinesin family member 18B | 4.42E-50 |
| ENSG00000137807 | KIF23 | Kinesin family member 23 | 1.07E-49 |
| ENSG00000123485 | HJURP | Holliday junction recognition protein | 9.76E-49 |
| ENSG00000136122 | BORA | BORA aurora kinase A activator | 1.39E-48 |
| ENSG00000165490 | DDIAS | DNA damage induced apoptosis suppressor | 2.80E-48 |
| ENSG00000146670 | CDCA5 | Cell division cycle associated 5 | 2.15E-47 |
| ENSG00000035499 | DEPDC1B | DEP domain containing 1B | 2.60E-47 |
| ENSG00000178999 | AURKB | Aurora kinase B | 1.99E-46 |
| ENSG00000276043 | UHRF1 | Ubiquitin like with PHD and ring finger domains 1 | 2.18E-46 |
| ENSG00000274641 | H2BC17 | H2B clustered histone 17 | 2.61E-46 |
| ENSG00000165304 | MELK | Maternal embryonic leucine zipper kinase | 2.82E-46 |
| ENSG00000161888 | SPC24 | SPC24 component of NDC80 kinetochore complex | 4.48E-45 |
| ENSG00000089685 | BIRC5 | Baculoviral IAP repeat containing 5 | 7.07E-45 |
| ENSG00000174371 | EXO1 | Exonuclease 1 | 1.65E-44 |
| ENSG00000111206 | FOXM1 | Forkhead box M1 | 8.33E-44 |
| ENSG00000274997 | H2AC12 | H2A clustered histone 12 | 1.03E-43 |
| ENSG00000093009 | CDC45 | Cell division cycle 45 | 1.43E-43 |
| ENSG00000184357 | H1-5 | H1.5 linker histone, cluster member | 4.58E-43 |
| ENSG00000119969 | HELLS | Helicase, lymphoid specific | 6.16E-43 |
| ENSG00000123080 | CDKN2C | Cyclin dependent kinase inhibitor 2C | 7.48E-43 |
| ENSG00000156970 | BUB1B | BUB1 mitotic checkpoint serine/threonine kinase B | 9.01E-43 |
| ENSG00000166803 | PCLAF | PCNA clamp associated factor | 1.39E-42 |
| ENSG00000100526 | CDKN3 | Cyclin dependent kinase inhibitor 3 | 1.97E-42 |
| ENSG00000182010 | RTKN2 | Rhotekin 2 | 6.20E-42 |
| ENSG00000014138 | POLA2 | DNA polymerase alpha 2, accessory subunit | 1.36E-41 |
| ENSG00000185480 | PARPBP | PARP1 binding protein | 1.66E-41 |
| ENSG00000137812 | KNL1 | Kinetochore scaffold 1 | 1.00E-40 |
| ENSG00000080986 | NDC80 | NDC80 kinetochore complex component | 5.33E-40 |
| ENSG00000165480 | SKA3 | Spindle and kinetochore associated complex subunit 3 | 5.33E-40 |
| ENSG00000164087 | POC1A | POC1 centriolar protein A | 1.17E-39 |
| ENSG00000276368 | H2AC14 | H2A clustered histone 14 | 1.64E-39 |
| ENSG00000175643 | RMI2 | Recq mediated genome instability 2 | 1.92E-39 |
| ENSG00000259316 | AC087632.2 | Novel protein | 2.51E-39 |
| ENSG00000196787 | H2AC11 | H2A clustered histone 11 | 2.96E-39 |
| ENSG00000146918 | NCAPG2 | Non-SMC condensin II complex subunit G2 | 7.41E-39 |
| ENSG00000138182 | KIF20B | Kinesin family member 20B | 2.10E-38 |
| ENSG00000177602 | HASPIN | Histone H3 associated protein kinase | 2.17E-38 |
| ENSG00000188486 | H2AX | H2A.X variant histone | 4.82E-38 |
| ENSG00000182481 | KPNA2 | Karyopherin subunit alpha 2 | 5.92E-38 |
| ENSG00000109674 | NEIL3 | Nei like DNA glycosylase 3 | 9.07E-38 |
| ENSG00000092470 | WDR76 | WD repeat domain 76 | 4.57E-37 |
| ENSG00000278588 | H2BC10 | H2B clustered histone 10 | 1.81E-36 |
| ENSG00000071539 | TRIP13 | Thyroid hormone receptor interactor 13 | 1.98E-36 |

| | | | |
|---|---|---|---|
| ENSG00000171320 | ESCO2 | Establishment of sister chromatid cohesion N-acetyltransferase 2 | 5.27E-36 |
| ENSG00000077152 | UBE2T | Ubiquitin conjugating enzyme E2 T | 5.99E-36 |
| ENSG00000011426 | ANLN | Anillin actin binding protein | 6.42E-36 |
| ENSG00000137310 | TCF19 | Transcription factor 19 | 3.43E-35 |
| ENSG00000196747 | H2AC13 | H2A clustered histone 13 | 1.90E-34 |
| ENSG00000124635 | H2BC11 | H2B clustered histone 11 | 8.04E-34 |
| ENSG00000007968 | E2F2 | E2F transcription factor 2 | 5.74E-33 |
| ENSG00000142731 | PLK4 | Polo like kinase 4 | 6.32E-33 |
| ENSG00000102384 | CENPI | Centromere protein I | 1.19E-32 |
| ENSG00000104147 | OIP5 | Opa interacting protein 5 | 1.33E-32 |
| ENSG00000139618 | BRCA2 | BRCA2 DNA repair associated | 2.60E-32 |
| ENSG00000121211 | MND1 | Meiotic nuclear divisions 1 | 5.32E-32 |
| ENSG00000197153 | H3C12 | H3 clustered histone 12 | 7.56E-32 |
| ENSG00000170540 | ARL6IP1 | ADP ribosylation factor like gtpase 6 interacting protein 1 | 2.14E-31 |
| ENSG00000100162 | CENPM | Centromere protein M | 9.49E-31 |
| ENSG00000135476 | ESPL1 | Extra spindle pole bodies like 1, separase | 1.52E-30 |
| ENSG00000087586 | AURKA | Aurora kinase A | 5.61E-30 |
| ENSG00000172244 | C5orf34 | Chromosome 5 open reading frame 34 | 8.35E-30 |
| ENSG00000277775 | H3C7 | H3 clustered histone 7 | 7.29E-29 |
| ENSG00000136982 | DSCC1 | DNA replication and sister chromatid cohesion 1 | 1.10E-28 |
| ENSG00000166845 | C18orf54 | Chromosome 18 open reading frame 54 | 1.12E-28 |
| ENSG00000113810 | SMC4 | Structural maintenance of chromosomes 4 | 1.77E-28 |
| ENSG00000112029 | FBXO5 | F-box protein 5 | 2.24E-28 |
| ENSG00000121957 | GPSM2 | G protein signaling modulator 2 | 2.24E-28 |
| ENSG00000144554 | FANCD2 | FA complementation group D2 | 2.37E-28 |
| ENSG00000167670 | CHAF1A | Chromatin assembly factor 1 subunit A | 3.43E-28 |
| ENSG00000282988 | AL031777.2 | Novel protein | 3.70E-28 |
| ENSG00000153044 | CENPH | Centromere protein H | 1.06E-27 |
| ENSG00000163507 | CIP2A | Cell proliferation regulating inhibitor of protein phosphatase 2A | 1.06E-27 |
| ENSG00000278677 | H2AC17 | H2A clustered histone 17 | 3.60E-27 |
| ENSG00000131153 | GINS2 | GINS complex subunit 2 | 8.28E-27 |
| ENSG00000151725 | CENPU | Centromere protein U | 1.60E-26 |
| ENSG00000073111 | MCM2 | Minichromosome maintenance complex component 2 | 4.28E-26 |
| ENSG00000140525 | FANCI | FA complementation group I | 8.75E-26 |
| ENSG00000168496 | FEN1 | Flap structure-specific endonuclease 1 | 8.78E-26 |
| ENSG00000076003 | MCM6 | Minichromosome maintenance complex component 6 | 1.19E-25 |
| ENSG00000072571 | HMMR | Hyaluronan mediated motility receptor | 1.69E-25 |
| ENSG00000173207 | CKS1B | CDC28 protein kinase regulatory subunit 1B | 4.45E-25 |
| ENSG00000101224 | CDC25B | Cell division cycle 25B | 5.02E-25 |
| ENSG00000165244 | ZNF367 | Zinc finger protein 367 | 5.57E-25 |
| ENSG00000117399 | CDC20 | Cell division cycle 20 | 6.80E-25 |
| ENSG00000111602 | TIMELESS | Timeless circadian regulator | 1.52E-24 |
| ENSG00000270882 | H4C14 | H4 clustered histone 14 | 2.06E-24 |
| ENSG00000213186 | TRIM59 | Tripartite motif containing 59 | 3.64E-24 |
| ENSG00000275713 | H2BC9 | H2B clustered histone 9 | 5.65E-24 |
| ENSG00000051180 | RAD51 | RAD51 recombinase | 6.61E-24 |
| ENSG00000188610 | FAM72B | Family with sequence similarity 72 member B | 1.16E-23 |
| ENSG00000101003 | GINS1 | GINS complex subunit 1 | 1.31E-23 |
| ENSG00000127564 | PKMYT1 | Protein kinase, membrane associated tyrosine/threonine 1 | 1.32E-23 |

| | | | |
|---|---|---|---|
| ENSG00000278463 | H2AC4 | H2A clustered histone 4 | 1.87E-23 |
| ENSG00000147536 | GINS4 | GINS complex subunit 4 | 2.49E-23 |
| ENSG00000164045 | CDC25A | Cell division cycle 25A | 6.68E-23 |
| ENSG00000133119 | RFC3 | Replication factor C subunit 3 | 9.48E-23 |
| ENSG00000203760 | CENPW | Centromere protein W | 9.58E-23 |
| ENSG00000113368 | LMNB1 | Lamin B1 | 1.08E-22 |
| ENSG00000284946 | AC068831.8 | Novel protein | 1.25E-22 |
| ENSG00000197472 | ZNF695 | Zinc finger protein 695 | 1.51E-22 |
| ENSG00000274750 | H3C6 | H3 clustered histone 6 | 2.00E-22 |
| ENSG00000085840 | ORC1 | Origin recognition complex subunit 1 | 3.74E-22 |
| ENSG00000154760 | SLFN13 | Schlafen family member 13 | 5.58E-22 |
| ENSG00000164104 | HMGB2 | High mobility group box 2 | 1.76E-21 |
| ENSG00000013810 | TACC3 | Transforming acidic coiled-coil containing protein 3 | 1.94E-21 |
| ENSG00000144354 | CDCA7 | Cell division cycle associated 7 | 2.07E-21 |
| ENSG00000186638 | KIF24 | Kinesin family member 24 | 4.38E-21 |
| ENSG00000164032 | H2AZ1 | H2A.Z variant histone 1 | 4.87E-21 |
| ENSG00000213347 | MXD3 | MAX dimerization protein 3 | 6.81E-21 |
| ENSG00000097046 | CDC7 | Cell division cycle 7 | 9.48E-21 |
| ENSG00000051341 | POLQ | DNA polymerase theta | 9.54E-21 |
| ENSG00000285920 | AC087721.2 | Novel protein | 1.01E-20 |
| ENSG00000166801 | FAM111A | Family with sequence similarity 111 member A | 1.04E-20 |
| ENSG00000005189 | REXO5 | RNA exonuclease 5 | 1.95E-20 |
| ENSG00000215784 | FAM72D | Family with sequence similarity 72 member D | 4.10E-20 |
| ENSG00000176890 | TYMS | Thymidylate synthetase | 4.68E-20 |
| ENSG00000127423 | AUNIP | Aurora kinase A and ninein interacting protein | 9.66E-20 |
| ENSG00000162063 | CCNF | Cyclin F | 1.02E-19 |
| ENSG00000273703 | H2BC14 | H2B clustered histone 14 | 2.25E-19 |
| ENSG00000117632 | STMN1 | Stathmin 1 | 2.80E-19 |
| ENSG00000276903 | H2AC16 | H2A clustered histone 16 | 3.02E-19 |
| ENSG00000163535 | SGO2 | Shugoshin 2 | 3.58E-19 |
| ENSG00000171208 | NETO2 | Neuropilin and tolloid like 2 | 4.32E-19 |
| ENSG00000174442 | ZWILCH | Zwilch kinetochore protein | 6.40E-19 |
| ENSG00000123473 | STIL | STIL centriolar assembly protein | 6.93E-19 |
| ENSG00000274290 | H2BC6 | H2B clustered histone 6 | 7.07E-19 |
| ENSG00000158164 | TMSB15A | Thymosin beta 15a | 8.21E-19 |
| ENSG00000176244 | ACBD7 | Acyl-coa binding domain containing 7 | 1.03E-18 |
| ENSG00000123219 | CENPK | Centromere protein K | 1.35E-18 |
| ENSG00000134222 | PSRC1 | Proline and serine rich coiled-coil 1 | 1.41E-18 |
| ENSG00000006634 | DBF4 | DBF4 zinc finger | 1.94E-18 |
| ENSG00000214357 | NEURL1B | Neuralized E3 ubiquitin protein ligase 1B | 2.09E-18 |
| ENSG00000100479 | POLE2 | DNA polymerase epsilon 2, accessory subunit | 2.40E-18 |
| ENSG00000123975 | CKS2 | CDC28 protein kinase regulatory subunit 2 | 2.64E-18 |
| ENSG00000120802 | TMPO | Thymopoietin | 3.21E-18 |
| ENSG00000076770 | MBNL3 | Muscleblind like splicing regulator 3 | 3.32E-18 |
| ENSG00000138092 | CENPO | Centromere protein O | 3.39E-18 |
| ENSG00000197299 | BLM | BLM recq like helicase | 4.91E-18 |
| ENSG00000123374 | CDK2 | Cyclin dependent kinase 2 | 6.77E-18 |
| ENSG00000106462 | EZH2 | Enhancer of zeste 2 polycomb repressive complex 2 subunit | 1.64E-17 |
| ENSG00000136492 | BRIP1 | BRCA1 interacting protein C-terminal helicase 1 | 2.46E-17 |
| ENSG00000159259 | CHAF1B | Chromatin assembly factor 1 subunit B | 2.46E-17 |

| | | | |
|---|---|---|---|
| ENSG00000275591 | XKR5 | XK related 5 | 2.91E-17 |
| ENSG00000198554 | WDHD1 | WD repeat and HMG-box DNA binding protein 1 | 1.67E-16 |
| ENSG00000183598 | H3C13 | H3 clustered histone 13 | 2.49E-16 |
| ENSG00000163918 | RFC4 | Replication factor C subunit 4 | 5.24E-16 |
| ENSG00000128944 | KNSTRN | Kinetochore localized astrin (SPAG5) binding protein | 6.57E-16 |
| ENSG00000148019 | CEP78 | Centrosomal protein 78 | 2.70E-15 |
| ENSG00000131470 | PSMC3IP | PSMC3 interacting protein | 3.76E-15 |
| ENSG00000197061 | H4C3 | H4 clustered histone 3 | 5.36E-15 |
| ENSG00000163808 | KIF15 | Kinesin family member 15 | 7.93E-15 |
| ENSG00000137563 | GGH | Gamma-glutamyl hydrolase | 8.11E-15 |
| ENSG00000102098 | SCML2 | Scm polycomb group protein like 2 | 8.39E-15 |
| ENSG00000185130 | H2BC13 | H2B clustered histone 13 | 8.82E-15 |
| ENSG00000100629 | CEP128 | Centrosomal protein 128 | 1.96E-14 |
| ENSG00000149503 | INCENP | Inner centromere protein | 2.09E-14 |
| ENSG00000168393 | DTYMK | Deoxythymidylate kinase | 2.16E-14 |
| ENSG00000278637 | H4C1 | H4 clustered histone 1 | 2.19E-14 |
| ENSG00000149636 | DSN1 | DSN1 component of MIS12 kinetochore complex | 2.64E-14 |
| ENSG00000183850 | ZNF730 | Zinc finger protein 730 | 4.00E-14 |
| ENSG00000167325 | RRM1 | Ribonucleotide reductase catalytic subunit M1 | 5.63E-14 |
| ENSG00000137135 | ARHGEF39 | Rho guanine nucleotide exchange factor 39 | 6.14E-14 |
| ENSG00000204899 | MZT1 | Mitotic spindle organizing protein 1 | 6.60E-14 |
| ENSG00000186777 | ZNF732 | Zinc finger protein 732 | 7.09E-14 |
| ENSG00000156876 | SASS6 | SAS-6 centriolar assembly protein | 9.14E-14 |
| ENSG00000112118 | MCM3 | Minichromosome maintenance complex component 3 | 1.01E-13 |
| ENSG00000183763 | TRAIP | TRAF interacting protein | 1.16E-13 |
| ENSG00000149554 | CHEK1 | Checkpoint kinase 1 | 1.17E-13 |
| ENSG00000168411 | RFWD3 | Ring finger and WD repeat domain 3 | 1.34E-13 |
| ENSG00000101412 | E2F1 | E2F transcription factor 1 | 1.55E-13 |
| ENSG00000185697 | MYBL1 | MYB proto-oncogene like 1 | 2.09E-13 |
| ENSG00000196550 | FAM72A | Family with sequence similarity 72 member A | 4.90E-13 |
| ENSG00000079616 | KIF22 | Kinesin family member 22 | 5.11E-13 |
| ENSG00000049541 | RFC2 | Replication factor C subunit 2 | 5.58E-13 |
| ENSG00000283559 | AC139491.6 | Centrosomal protein 192kda (CEP192) pseudogene | 5.66E-13 |
| ENSG00000160957 | RECQL4 | Recq like helicase 4 | 5.91E-13 |
| ENSG00000122483 | CCDC18 | Coiled-coil domain containing 18 | 7.76E-13 |
| ENSG00000187741 | FANCA | FA complementation group A | 8.96E-13 |
| ENSG00000277157 | H4C4 | H4 clustered histone 4 | 1.40E-12 |
| ENSG00000120539 | MASTL | Microtubule associated serine/threonine kinase like | 1.53E-12 |
| ENSG00000156802 | ATAD2 | Atpase family AAA domain containing 2 | 2.15E-12 |
| ENSG00000146143 | PRIM2 | DNA primase subunit 2 | 2.68E-12 |
| ENSG00000129534 | MIS18BP1 | MIS18 binding protein 1 | 3.66E-12 |
| ENSG00000186767 | SPIN4 | Spindlin family member 4 | 4.11E-12 |
| ENSG00000221829 | FANCG | FA complementation group G | 7.18E-12 |
| ENSG00000140534 | TICRR | TOPBP1 interacting checkpoint and replication regulator | 7.36E-12 |
| ENSG00000196584 | XRCC2 | X-ray repair cross complementing 2 | 1.26E-11 |
| ENSG00000100749 | VRK1 | VRK serine/threonine kinase 1 | 1.92E-11 |
| ENSG00000114346 | ECT2 | Epithelial cell transforming 2 | 2.48E-11 |
| ENSG00000119403 | PHF19 | PHD finger protein 19 | 3.06E-11 |
| ENSG00000196866 | H2AC7 | H2A clustered histone 7 | 3.29E-11 |
| ENSG00000156136 | DCK | Deoxycytidine kinase | 3.58E-11 |
| ENSG00000277075 | H2AC8 | H2A clustered histone 8 | 3.90E-11 |

| | | | |
|---|---|---|---|
| ENSG00000124575 | H1-3 | H1.3 linker histone, cluster member | 4.22E-11 |
| ENSG00000275714 | H3C1 | H3 clustered histone 1 | 4.57E-11 |
| ENSG00000146410 | MTFR2 | Mitochondrial fission regulator 2 | 4.73E-11 |
| ENSG00000170779 | CDCA4 | Cell division cycle associated 4 | 4.87E-11 |
| ENSG00000092140 | G2E3 | G2/M-phase specific E3 ubiquitin protein ligase | 6.24E-11 |
| ENSG00000198830 | HMGN2 | High mobility group nucleosomal binding domain 2 | 7.07E-11 |
| ENSG00000125885 | MCM8 | Minichromosome maintenance 8 homologous recombination repair factor | 7.32E-11 |
| ENSG00000166881 | NEMP1 | Nuclear envelope integral membrane protein 1 | 8.09E-11 |
| ENSG00000103995 | CEP152 | Centrosomal protein 152 | 1.01E-10 |
| ENSG00000176208 | ATAD5 | Atpase family AAA domain containing 5 | 1.31E-10 |
| ENSG00000171792 | RHNO1 | RAD9-HUS1-RAD1 interacting nuclear orphan 1 | 1.36E-10 |
| ENSG00000278828 | H3C10 | H3 clustered histone 10 | 1.44E-10 |
| ENSG00000154920 | EME1 | Essential meiotic structure-specific endonuclease 1 | 2.45E-10 |
| ENSG00000101945 | SUV39H1 | Suppressor of variegation 3-9 homolog 1 | 2.92E-10 |
| ENSG00000234289 | H2BS1 | H2B.S histone 1 | 2.97E-10 |
| ENSG00000166508 | MCM7 | Minichromosome maintenance complex component 7 | 3.91E-10 |
| ENSG00000115159 | GPD2 | Glycerol-3-phosphate dehydrogenase 2 | 4.38E-10 |
| ENSG00000132436 | FIGNL1 | Fidgetin like 1 | 4.84E-10 |
| ENSG00000160298 | C21orf58 | Chromosome 21 open reading frame 58 | 5.63E-10 |
| ENSG00000151849 | CENPJ | Centromere protein J | 5.92E-10 |
| ENSG00000112877 | CEP72 | Centrosomal protein 72 | 7.62E-10 |
| ENSG00000198056 | PRIM1 | DNA primase subunit 1 | 8.00E-10 |
| ENSG00000284491 | THSD8 | Thrombospondin type 1 domain containing 8 | 1.00E-09 |
| ENSG00000185361 | TNFAIP8L1 | TNF alpha induced protein 8 like 1 | 1.13E-09 |
| ENSG00000180596 | H2BC4 | H2B clustered histone 4 | 2.85E-09 |
| ENSG00000040275 | SPDL1 | Spindle apparatus coiled-coil protein 1 | 3.08E-09 |
| ENSG00000277224 | H2BC7 | H2B clustered histone 7 | 3.51E-09 |
| ENSG00000146263 | MMS22L | MMS22 like, DNA repair protein | 4.58E-09 |
| ENSG00000112312 | GMNN | Geminin DNA replication inhibitor | 5.01E-09 |
| ENSG00000205208 | C4orf46 | Chromosome 4 open reading frame 46 | 5.63E-09 |
| ENSG00000166451 | CENPN | Centromere protein N | 7.63E-09 |
| ENSG00000172167 | MTBP | MDM2 binding protein | 1.10E-08 |
| ENSG00000163923 | RPL39L | Ribosomal protein L39 like | 1.12E-08 |
| ENSG00000187123 | LYPD6 | LY6/PLAUR domain containing 6 | 1.17E-08 |
| ENSG00000120334 | CENPL | Centromere protein L | 1.55E-08 |
| ENSG00000133101 | CCNA1 | Cyclin A1 | 2.03E-08 |
| ENSG00000115687 | PASK | PAS domain containing serine/threonine kinase | 2.73E-08 |
| ENSG00000197238 | H4C11 | H4 clustered histone 11 | 3.80E-08 |
| ENSG00000080839 | RBL1 | RB transcriptional corepressor like 1 | 4.03E-08 |
| ENSG00000188312 | CENPP | Centromere protein P | 4.20E-08 |
| ENSG00000167747 | C19orf48 | Chromosome 19 open reading frame 48 | 4.54E-08 |
| ENSG00000064199 | SPA17 | Sperm autoantigenic protein 17 | 5.23E-08 |
| ENSG00000198331 | HYLS1 | HYLS1 centriolar and ciliogenesis associated | 6.75E-08 |
| ENSG00000126215 | XRCC3 | X-ray repair cross complementing 3 | 7.28E-08 |
| ENSG00000158406 | H4C8 | H4 clustered histone 8 | 7.29E-08 |
| ENSG00000031691 | CENPQ | Centromere protein Q | 7.30E-08 |
| ENSG00000115392 | FANCL | FA complementation group L | 8.50E-08 |
| ENSG00000189403 | HMGB1 | High mobility group box 1 | 1.10E-07 |
| ENSG00000278705 | H4C2 | H4 clustered histone 2 | 1.15E-07 |
| ENSG00000276180 | H4C9 | H4 clustered histone 9 | 1.17E-07 |

| | | | |
|---|---|---|---|
| ENSG00000123136 | DDX39A | Dexd-box helicase 39A | 1.17E-07 |
| ENSG00000181544 | FANCB | FA complementation group B | 1.21E-07 |
| ENSG00000228716 | DHFR | Dihydrofolate reductase | 1.21E-07 |
| ENSG00000006047 | YBX2 | Y-box binding protein 2 | 1.30E-07 |
| ENSG00000136943 | CTSV | Cathepsin V | 1.32E-07 |
| ENSG00000273802 | H2BC8 | H2B clustered histone 8 | 1.41E-07 |
| ENSG00000237649 | KIFC1 | Kinesin family member C1 | 1.52E-07 |
| ENSG00000160949 | TONSL | Tonsoku like, DNA repair protein | 1.71E-07 |
| ENSG00000175175 | PPM1E | Protein phosphatase, Mg2+/Mn2+ dependent 1E | 1.82E-07 |
| ENSG00000128245 | YWHAH | Tyrosine 3-monooxygenase/tryptophan 5-monooxygenase activation protein eta | 2.30E-07 |
| ENSG00000197275 | RAD54B | RAD54 homolog B | 2.39E-07 |
| ENSG00000154839 | SKA1 | Spindle and kinetochore associated complex subunit 1 | 2.58E-07 |
| ENSG00000133863 | TEX15 | Testis expressed 15, meiosis and synapsis associated | 2.72E-07 |
| ENSG00000127337 | YEATS4 | YEATS domain containing 4 | 2.79E-07 |
| ENSG00000275379 | H3C11 | H3 clustered histone 11 | 2.84E-07 |
| ENSG00000145832 | SLC25A48 | Solute carrier family 25 member 48 | 3.01E-07 |
| ENSG00000138346 | DNA2 | DNA replication helicase/nuclease 2 | 3.14E-07 |
| ENSG00000162607 | USP1 | Ubiquitin specific peptidase 1 | 3.89E-07 |
| ENSG00000285077 | ARHGAP11B | Rho gtpase activating protein 11B | 4.16E-07 |
| ENSG00000144395 | CCDC150 | Coiled-coil domain containing 150 | 4.21E-07 |
| ENSG00000105486 | LIG1 | DNA ligase 1 | 4.25E-07 |
| ENSG00000159055 | MIS18A | MIS18 kinetochore protein A | 4.37E-07 |
| ENSG00000180198 | RCC1 | Regulator of chromosome condensation 1 | 4.72E-07 |
| ENSG00000135045 | C9orf40 | Chromosome 9 open reading frame 40 | 4.81E-07 |
| ENSG00000077264 | PAK3 | P21 (RAC1) activated kinase 3 | 4.90E-07 |
| ENSG00000167553 | TUBA1C | Tubulin alpha 1c | 4.90E-07 |
| ENSG00000143815 | LBR | Lamin B receptor | 4.90E-07 |
| ENSG00000124795 | DEK | DEK proto-oncogene | 7.52E-07 |
| ENSG00000138376 | BARD1 | BRCA1 associated RING domain 1 | 7.86E-07 |
| ENSG00000233822 | H2BC15 | H2B clustered histone 15 | 8.68E-07 |
| ENSG00000152503 | TRIM36 | Tripartite motif containing 36 | 9.65E-07 |
| ENSG00000131773 | KHDRBS3 | KH RNA binding domain containing, signal transduction associated 3 | 9.83E-07 |
| ENSG00000131351 | HAUS8 | HAUS augmin like complex subunit 8 | 1.01E-06 |
| ENSG00000109084 | TMEM97 | Transmembrane protein 97 | 1.01E-06 |
| ENSG00000140451 | PIF1 | PIF1 5'-to-3' DNA helicase | 1.07E-06 |
| ENSG00000197903 | H2BC12 | H2B clustered histone 12 | 1.17E-06 |
| ENSG00000196247 | ZNF107 | Zinc finger protein 107 | 1.18E-06 |
| ENSG00000189159 | JPT1 | Jupiter microtubule associated homolog 1 | 1.24E-06 |
| ENSG00000111445 | RFC5 | Replication factor C subunit 5 | 1.44E-06 |
| ENSG00000178966 | RMI1 | Recq mediated genome instability 1 | 1.46E-06 |
| ENSG00000135407 | AVIL | Advillin | 1.68E-06 |
| ENSG00000163781 | TOPBP1 | DNA topoisomerase II binding protein 1 | 1.69E-06 |
| ENSG00000072864 | NDE1 | Nude neurodevelopment protein 1 | 2.08E-06 |
| ENSG00000154473 | BUB3 | BUB3 mitotic checkpoint protein | 2.09E-06 |
| ENSG00000119397 | CNTRL | Centriolin | 2.21E-06 |
| ENSG00000062822 | POLD1 | DNA polymerase delta 1, catalytic subunit | 2.23E-06 |
| ENSG00000128408 | RIBC2 | RIB43A domain with coiled-coils 2 | 2.31E-06 |
| ENSG00000099901 | RANBP1 | RAN binding protein 1 | 2.73E-06 |
| ENSG00000178878 | APOLD1 | Apolipoprotein L domain containing 1 | 2.74E-06 |

| | | | |
|---|---|---|---|
| ENSG00000132646 | PCNA | Proliferating cell nuclear antigen | 2.77E-06 |
| ENSG00000092621 | PHGDH | Phosphoglycerate dehydrogenase | 3.10E-06 |
| ENSG00000185432 | METTL7A | Methyltransferase like 7A | 3.12E-06 |
| ENSG00000165197 | VEGFD | Vascular endothelial growth factor D | 3.25E-06 |
| ENSG00000111581 | NUP107 | Nucleoporin 107 | 3.39E-06 |
| ENSG00000125319 | HROB | Homologous recombination factor with OB-fold | 3.73E-06 |
| ENSG00000158373 | H2BC5 | H2B clustered histone 5 | 4.25E-06 |
| ENSG00000010292 | NCAPD2 | Non-SMC condensin I complex subunit D2 | 4.63E-06 |
| ENSG00000091651 | ORC6 | Origin recognition complex subunit 6 | 5.20E-06 |
| ENSG00000284906 | ARHGAP11B | Rho gtpase activating protein 11B | 5.29E-06 |
| ENSG00000198176 | TFDP1 | Transcription factor Dp-1 | 5.47E-06 |
| ENSG00000079819 | EPB41L2 | Erythrocyte membrane protein band 4.1 like 2 | 5.69E-06 |
| ENSG00000175455 | CCDC14 | Coiled-coil domain containing 14 | 5.93E-06 |
| ENSG00000278023 | RDM1 | RAD52 motif containing 1 | 6.48E-06 |
| ENSG00000159147 | DONSON | Downstream neighbor of SON | 6.92E-06 |
| ENSG00000094916 | CBX5 | Chromobox 5 | 8.04E-06 |
| ENSG00000106399 | RPA3 | Replication protein A3 | 8.37E-06 |
| ENSG00000095002 | MSH2 | Muts homolog 2 | 8.91E-06 |
| ENSG00000152270 | PDE3B | Phosphodiesterase 3B | 9.13E-06 |
| ENSG00000132967 | HMGB1P5 | High mobility group box 1 pseudogene 5 | 1.00E-05 |
| ENSG00000152240 | HAUS1 | HAUS augmin like complex subunit 1 | 1.01E-05 |
| ENSG00000132749 | TESMIN | Testis expressed metallothionein like protein | 1.03E-05 |
| ENSG00000172687 | ZNF738 | Zinc finger protein 738 | 1.03E-05 |
| ENSG00000136824 | SMC2 | Structural maintenance of chromosomes 2 | 1.16E-05 |
| ENSG00000104738 | MCM4 | Minichromosome maintenance complex component 4 | 1.24E-05 |
| ENSG00000118655 | DCLRE1B | DNA cross-link repair 1B | 1.24E-05 |
| ENSG00000169918 | OTUD7A | OTU deubiquitinase 7A | 1.27E-05 |
| ENSG00000145375 | SPATA5 | Spermatogenesis associated 5 | 1.46E-05 |
| ENSG00000160447 | PKN3 | Protein kinase N3 | 1.48E-05 |
| ENSG00000185710 | SMG1P4 | SMG1 pseudogene 4 | 1.50E-05 |
| ENSG00000165891 | E2F7 | E2F transcription factor 7 | 1.50E-05 |
| ENSG00000168476 | REEP4 | Receptor accessory protein 4 | 1.65E-05 |
| ENSG00000187790 | FANCM | FA complementation group M | 1.74E-05 |
| ENSG00000142149 | HUNK | Hormonally up-regulated Neu-associated kinase | 2.00E-05 |
| ENSG00000256663 | AC112777.1 | Ubiquitin-like with PHD and ring finger domains 1 (UHRF1) pseudogene | 2.06E-05 |
| ENSG00000160229 | ZNF66 | Zinc finger protein 66 | 2.09E-05 |
| ENSG00000155755 | TMEM237 | Transmembrane protein 237 | 2.11E-05 |
| ENSG00000163002 | NUP35 | Nucleoporin 35 | 2.19E-05 |
| ENSG00000165501 | LRR1 | Leucine rich repeat protein 1 | 2.38E-05 |
| ENSG00000182504 | CEP97 | Centrosomal protein 97 | 2.50E-05 |
| ENSG00000103495 | MAZ | MYC associated zinc finger protein | 2.67E-05 |
| ENSG00000231205 | ZNF826P | Zinc finger protein 826, pseudogene | 2.91E-05 |
| ENSG00000258555 | SPECC1L-ADORA2A | SPECC1L-ADORA2A readthrough (NMD candidate) | 3.12E-05 |
| ENSG00000130270 | ATP8B3 | Atpase phospholipid transporting 8B3 | 3.16E-05 |
| ENSG00000177084 | POLE | DNA polymerase epsilon, catalytic subunit | 3.36E-05 |
| ENSG00000267041 | ZNF850 | Zinc finger protein 850 | 3.45E-05 |
| ENSG00000070081 | NUCB2 | Nucleobindin 2 | 3.52E-05 |
| ENSG00000184661 | CDCA2 | Cell division cycle associated 2 | 3.87E-05 |
| ENSG00000174799 | CEP135 | Centrosomal protein 135 | 3.95E-05 |

| | | | |
|---|---|---|---|
| ENSG00000154146 | NRGN | Neurogranin | 4.17E-05 |
| ENSG00000184897 | H1-10 | H1.10 linker histone | 4.58E-05 |
| ENSG00000164985 | PSIP1 | PC4 and SFRS1 interacting protein 1 | 4.78E-05 |
| ENSG00000176619 | LMNB2 | Lamin B2 | 4.84E-05 |
| ENSG00000175445 | LPL | Lipoprotein lipase | 4.91E-05 |
| ENSG00000133739 | LRRCC1 | Leucine rich repeat and coiled-coil centrosomal protein 1 | 5.27E-05 |
| ENSG00000136518 | ACTL6A | Actin like 6A | 5.44E-05 |
| ENSG00000099256 | PRTFDC1 | Phosphoribosyl transferase domain containing 1 | 6.89E-05 |
| ENSG00000173218 | VANGL1 | VANGL planar cell polarity protein 1 | 7.03E-05 |
| ENSG00000176974 | SHMT1 | Serine hydroxymethyltransferase 1 | 7.09E-05 |
| ENSG00000137337 | MDC1 | Mediator of DNA damage checkpoint 1 | 7.45E-05 |
| ENSG00000124207 | CSE1L | Chromosome segregation 1 like | 8.64E-05 |
| ENSG00000116830 | TTF2 | Transcription termination factor 2 | 8.87E-05 |
| ENSG00000081320 | STK17B | Serine/threonine kinase 17b | 9.07E-05 |
| ENSG00000101868 | POLA1 | DNA polymerase alpha 1, catalytic subunit | 9.38E-05 |
| ENSG00000158427 | TMSB15B | Thymosin beta 15B | 9.44E-05 |
| ENSG00000050438 | SLC4A8 | Solute carrier family 4 member 8 | 0.000106 |
| ENSG00000256229 | ZNF486 | Zinc finger protein 486 | 0.000106 |
| ENSG00000123737 | EXOSC9 | Exosome component 9 | 0.00011 |
| ENSG00000106268 | NUDT1 | Nudix hydrolase 1 | 0.000112 |
| ENSG00000109861 | CTSC | Cathepsin C | 0.000117 |
| ENSG00000198189 | HSD17B11 | Hydroxysteroid 17-beta dehydrogenase 11 | 0.000134 |
| ENSG00000183765 | CHEK2 | Checkpoint kinase 2 | 0.000149 |
| ENSG00000213551 | DNAJC9 | Dnaj heat shock protein family (Hsp40) member C9 | 0.000151 |
| ENSG00000070950 | RAD18 | RAD18 E3 ubiquitin protein ligase | 0.000153 |
| ENSG00000115875 | SRSF7 | Serine and arginine rich splicing factor 7 | 0.000161 |
| ENSG00000267500 | ZNF887P | Zinc finger protein 887, pseudogene | 0.000166 |
| ENSG00000136108 | CKAP2 | Cytoskeleton associated protein 2 | 0.000187 |
| ENSG00000095777 | MYO3A | Myosin IIIA | 0.00019 |
| ENSG00000144485 | HES6 | Hes family bhlh transcription factor 6 | 0.000192 |
| ENSG00000274588 | DGKK | Diacylglycerol kinase kappa | 0.000223 |
| ENSG00000100242 | SUN2 | Sad1 and UNC84 domain containing 2 | 0.000239 |
| ENSG00000156509 | FBXO43 | F-box protein 43 | 0.00024 |
| ENSG00000128951 | DUT | Deoxyuridine triphosphatase | 0.000243 |
| ENSG00000187837 | H1-2 | H1.2 linker histone, cluster member | 0.000256 |
| ENSG00000155561 | NUP205 | Nucleoporin 205 | 0.000274 |
| ENSG00000167617 | CDC42EP5 | CDC42 effector protein 5 | 0.000282 |
| ENSG00000149548 | CCDC15 | Coiled-coil domain containing 15 | 0.000317 |
| ENSG00000214826 | DDX12P | DEAD/H-box helicase 12, pseudogene | 0.000325 |
| ENSG00000067955 | CBFB | Core-binding factor subunit beta | 0.000344 |
| ENSG00000162062 | TEDC2 | Tubulin epsilon and delta complex 2 | 0.000358 |
| ENSG00000179841 | AKAP5 | A-kinase anchoring protein 5 | 0.000405 |
| ENSG00000103540 | CCP110 | Centriolar coiled-coil protein 110 | 0.000415 |
| ENSG00000259781 | HMGB1P6 | High mobility group box 1 pseudogene 6 | 0.000423 |
| ENSG00000144645 | OSBPL10 | Oxysterol binding protein like 10 | 0.000429 |
| ENSG00000136811 | ODF2 | Outer dense fiber of sperm tails 2 | 0.000447 |
| ENSG00000081870 | HSPB11 | Heat shock protein family B (small) member 11 | 0.000492 |
| ENSG00000158201 | ABHD3 | Abhydrolase domain containing 3 | 0.000587 |
| ENSG00000178295 | GEN1 | GEN1 Holliday junction 5' flap endonuclease | 0.000634 |
| ENSG00000076248 | UNG | Uracil DNA glycosylase | 0.00069 |

| | | | |
|---|---|---|---|
| ENSG00000106355 | LSM5 | LSM5 homolog, U6 small nuclear RNA and mrna degradation associated | 0.000694 |
| ENSG00000259845 | HERC2P10 | Hect domain and RLD 2 pseudogene 10 | 0.000698 |
| ENSG00000275221 | H2AC15 | H2A clustered histone 15 | 0.000699 |
| ENSG00000242419 | PCDHGC4 | Protocadherin gamma subfamily C, 4 | 0.0007 |
| ENSG00000077514 | POLD3 | DNA polymerase delta 3, accessory subunit | 0.000702 |
| ENSG00000125148 | MT2A | Metallothionein 2A | 0.000725 |
| ENSG00000101773 | RBBP8 | RB binding protein 8, endonuclease | 0.000773 |
| ENSG00000126453 | BCL2L12 | BCL2 like 12 | 0.000785 |
| ENSG00000248710 | AC079594.2 | TRIM59 and ift80 readthrough | 0.000797 |
| ENSG00000197451 | HNRNPAB | Heterogeneous nuclear ribonucleoprotein A/B | 0.000898 |
| ENSG00000080200 | CRYBG3 | Crystallin beta-gamma domain containing 3 | 0.000912 |
| ENSG00000260342 | AC138811.2 | Novel protein | 0.000916 |
| ENSG00000104219 | ZDHHC2 | Zinc finger DHHC-type palmitoyltransferase 2 | 0.000937 |
| ENSG00000272196 | H2AC19 | H2A clustered histone 19 | 0.000939 |
| ENSG00000100578 | KIAA0586 | Kiaa0586 | 0.000966 |
| ENSG00000275126 | H4C13 | H4 clustered histone 13 | 0.000979 |
| ENSG00000183684 | ALYREF | Aly/REF export factor | 0.000984 |
| ENSG00000163006 | CCDC138 | Coiled-coil domain containing 138 | 0.000999 |
| ENSG00000029993 | HMGB3 | High mobility group box 3 | 0.001048 |
| ENSG00000263513 | FAM72C | Family with sequence similarity 72 member C | 0.001078 |
| ENSG00000179750 | APOBEC3B | Apolipoprotein B mrna editing enzyme catalytic subunit 3B | 0.001273 |
| ENSG00000187833 | C2orf78 | Chromosome 2 open reading frame 78 | 0.001295 |
| ENSG00000162174 | ASRGL1 | Asparaginase and isoaspartyl peptidase 1 | 0.001295 |
| ENSG00000012048 | BRCA1 | BRCA1 DNA repair associated | 0.001392 |
| ENSG00000163950 | SLBP | Stem-loop binding protein | 0.001392 |
| ENSG00000116062 | MSH6 | Muts homolog 6 | 0.001401 |
| ENSG00000177917 | ARL6IP6 | ADP ribosylation factor like gtpase 6 interacting protein 6 | 0.001414 |
| ENSG00000217555 | CKLF | Chemokine like factor | 0.001614 |
| ENSG00000184635 | ZNF93 | Zinc finger protein 93 | 0.00165 |
| ENSG00000214796 | AC098934.1 | Tubulin, alpha (TUBA) pseudogene | 0.001755 |
| ENSG00000013573 | DDX11 | DEAD/H-box helicase 11 | 0.001846 |
| ENSG00000162636 | FAM102B | Family with sequence similarity 102 member B | 0.001932 |
| ENSG00000108468 | CBX1 | Chromobox 1 | 0.001949 |
| ENSG00000156398 | SFXN2 | Sideroflexin 2 | 0.001949 |
| ENSG00000152455 | SUV39H2 | Suppressor of variegation 3-9 homolog 2 | 0.002016 |
| ENSG00000164754 | RAD21 | RAD21 cohesin complex component | 0.002147 |
| ENSG00000029153 | ARNTL2 | Aryl hydrocarbon receptor nuclear translocator like 2 | 0.002311 |
| ENSG00000143493 | INTS7 | Integrator complex subunit 7 | 0.002371 |
| ENSG00000147050 | KDM6A | Lysine demethylase 6A | 0.002564 |
| ENSG00000120647 | CCDC77 | Coiled-coil domain containing 77 | 0.002565 |
| ENSG00000178343 | SHISA3 | Shisa family member 3 | 0.002773 |
| ENSG00000162039 | MEIOB | Meiosis specific with OB-fold | 0.002914 |
| ENSG00000167088 | SNRPD1 | Small nuclear ribonucleoprotein D1 polypeptide | 0.002961 |
| ENSG00000122566 | HNRNPA2B1 | Heterogeneous nuclear ribonucleoprotein A2/B1 | 0.003167 |
| ENSG00000134291 | TMEM106C | Transmembrane protein 106C | 0.003506 |
| ENSG00000187134 | AKR1C1 | Aldo-keto reductase family 1 member C1 | 0.003687 |
| ENSG00000152253 | SPC25 | SPC25 component of NDC80 kinetochore complex | 0.003714 |
| ENSG00000196172 | ZNF681 | Zinc finger protein 681 | 0.003918 |
| ENSG00000149929 | HIRIP3 | HIRA interacting protein 3 | 0.004202 |

| | | | |
|---|---|---|---|
| ENSG00000276256 | AC011043.1 | Uncharacterized LOC389831 | 0.004312 |
| ENSG00000178177 | LCORL | Ligand dependent nuclear receptor corepressor like | 0.004477 |
| ENSG00000154040 | CABYR | Calcium binding tyrosine phosphorylation regulated | 0.004515 |
| ENSG00000196081 | ZNF724 | Zinc finger protein 724 | 0.004551 |
| ENSG00000109255 | NMU | Neuromedin U | 0.004588 |
| ENSG00000004777 | ARHGAP33 | Rho gtpase activating protein 33 | 0.004665 |
| ENSG00000120699 | EXOSC8 | Exosome component 8 | 0.004677 |
| ENSG00000160352 | ZNF714 | Zinc finger protein 714 | 0.004715 |
| ENSG00000175322 | ZNF519 | Zinc finger protein 519 | 0.004817 |
| ENSG00000100206 | DMC1 | DNA meiotic recombinase 1 | 0.00495 |
| ENSG00000100401 | RANGAP1 | Ran gtpase activating protein 1 | 0.004974 |
| ENSG00000251188 | AC079140.3 | Novel zinc finger protein pseudogene | 0.005107 |
| ENSG00000214425 | LRRC37A4P | Leucine rich repeat containing 37 member A4, pseudogene | 0.005111 |
| ENSG00000188985 | DHFRP1 | Dihydrofolate reductase pseudogene 1 | 0.005143 |
| ENSG00000000460 | C1orf112 | Chromosome 1 open reading frame 112 | 0.005155 |
| ENSG00000089280 | FUS | FUS RNA binding protein | 0.00523 |
| ENSG00000112081 | SRSF3 | Serine and arginine rich splicing factor 3 | 0.005289 |
| ENSG00000147231 | RADX | RPA1 related single stranded DNA binding protein, X-linked | 0.005719 |
| ENSG00000128059 | PPAT | Phosphoribosyl pyrophosphate amidotransferase | 0.005774 |
| ENSG00000253250 | C8orf88 | Chromosome 8 open reading frame 88 | 0.005837 |
| ENSG00000235748 | SEPTIN14P12 | Septin 14 pseudogene 12 | 0.005948 |
| ENSG00000104626 | ERI1 | Exoribonuclease 1 | 0.006691 |
| ENSG00000105750 | ZNF85 | Zinc finger protein 85 | 0.006724 |
| ENSG00000187514 | PTMA | Prothymosin alpha | 0.006724 |
| ENSG00000184992 | BRI3BP | BRI3 binding protein | 0.006796 |
| ENSG00000198157 | HMGN5 | High mobility group nucleosome binding domain 5 | 0.006989 |
| ENSG00000147274 | RBMX | RNA binding motif protein X-linked | 0.007154 |
| ENSG00000151287 | TEX30 | Testis expressed 30 | 0.007212 |
| ENSG00000162073 | PAQR4 | Progestin and adipoq receptor family member 4 | 0.007582 |
| ENSG00000119333 | WDR34 | WD repeat domain 34 | 0.007634 |
| ENSG00000213024 | NUP62 | Nucleoporin 62 | 0.007664 |
| ENSG00000149136 | SSRP1 | Structure specific recognition protein 1 | 0.007799 |
| ENSG00000105717 | PBX4 | PBX homeobox 4 | 0.008232 |
| ENSG00000132780 | NASP | Nuclear autoantigenic sperm protein | 0.008232 |
| ENSG00000250312 | ZNF718 | Zinc finger protein 718 | 0.008774 |
| ENSG00000198088 | NUP62CL | Nucleoporin 62 C-terminal like | 0.009001 |
| ENSG00000271254 | AC240274.1 | Neuroblastoma breakpoint family member 1 | 0.009001 |
| ENSG00000025770 | NCAPH2 | Non-SMC condensin II complex subunit H2 | 0.009174 |
| ENSG00000151503 | NCAPD3 | Non-SMC condensin II complex subunit D3 | 0.00932 |
| ENSG00000204209 | DAXX | Death domain associated protein | 0.009468 |
| ENSG00000214944 | ARHGEF28 | Rho guanine nucleotide exchange factor 28 | 0.009631 |
| ENSG00000175130 | MARCKSL1 | MARCKS like 1 | 0.009761 |
| ENSG00000171793 | CTPS1 | CTP synthase 1 | 0.009934 |
| ENSG00000079387 | SENP1 | SUMO specific peptidase 1 | 0.009978 |
| ENSG00000197121 | PGAP1 | Post-GPI attachment to proteins inositol deacylase 1 | 0.009987 |
| ENSG00000148835 | TAF5 | TATA-box binding protein associated factor 5 | 0.010421 |
| ENSG00000173848 | NET1 | Neuroepithelial cell transforming 1 | 0.010811 |
| ENSG00000077684 | JADE1 | Jade family PHD finger 1 | 0.01111 |

| | | | |
|---|---|---|---|
| ENSG00000151388 | ADAMTS12 | ADAM metallopeptidase with thrombospondin type 1 motif 12 | 0.011792 |
| ENSG00000136861 | CDK5RAP2 | CDK5 regulatory subunit associated protein 2 | 0.012035 |
| ENSG00000186298 | PPP1CC | Protein phosphatase 1 catalytic subunit gamma | 0.012084 |
| ENSG00000169684 | CHRNA5 | Cholinergic receptor nicotinic alpha 5 subunit | 0.012309 |
| ENSG00000118298 | CA14 | Carbonic anhydrase 14 | 0.012535 |
| ENSG00000244306 | AL589743.1 | Double homeobox A pseudogene 10 | 0.013477 |
| ENSG00000123416 | TUBA1B | Tubulin alpha 1b | 0.013583 |
| ENSG00000196230 | TUBB | Tubulin beta class I | 0.013583 |
| ENSG00000141576 | RNF157 | Ring finger protein 157 | 0.014763 |
| ENSG00000068028 | RASSF1 | Ras association domain family member 1 | 0.014917 |
| ENSG00000198435 | NRARP | NOTCH regulated ankyrin repeat protein | 0.01502 |
| ENSG00000139291 | TMEM19 | Transmembrane protein 19 | 0.015387 |
| ENSG00000139734 | DIAPH3 | Diaphanous related formin 3 | 0.015427 |
| ENSG00000269226 | TMSB15B | Thymosin beta 15B | 0.016019 |
| ENSG00000115355 | CCDC88A | Coiled-coil domain containing 88A | 0.016645 |
| ENSG00000162929 | KIAA1841 | Kiaa1841 | 0.01682 |
| ENSG00000107566 | ERLIN1 | ER lipid raft associated 1 | 0.017038 |
| ENSG00000141401 | IMPA2 | Inositol monophosphatase 2 | 0.017097 |
| ENSG00000198521 | ZNF43 | Zinc finger protein 43 | 0.017097 |
| ENSG00000174600 | CMKLR1 | Chemerin chemokine-like receptor 1 | 0.017237 |
| ENSG00000182628 | SKA2 | Spindle and kinetochore associated complex subunit 2 | 0.017932 |
| ENSG00000174010 | KLHL15 | Kelch like family member 15 | 0.018042 |
| ENSG00000125450 | NUP85 | Nucleoporin 85 | 0.01827 |
| ENSG00000277112 | ANKRD20A21P | Ankyrin repeat domain 20 family member A21, pseudogene | 0.018527 |
| ENSG00000176894 | PXMP2 | Peroxisomal membrane protein 2 | 0.018527 |
| ENSG00000099783 | HNRNPM | Heterogeneous nuclear ribonucleoprotein M | 0.01863 |
| ENSG00000133985 | TTC9 | Tetratricopeptide repeat domain 9 | 0.01902 |
| ENSG00000132016 | C19orf57 | Chromosome 19 open reading frame 57 | 0.019595 |
| ENSG00000143643 | TTC13 | Tetratricopeptide repeat domain 13 | 0.019841 |
| ENSG00000011332 | DPF1 | Double PHD fingers 1 | 0.019976 |
| ENSG00000101911 | PRPS2 | Phosphoribosyl pyrophosphate synthetase 2 | 0.02015 |
| ENSG00000197020 | ZNF100 | Zinc finger protein 100 | 0.020626 |
| ENSG00000138587 | MNS1 | Meiosis specific nuclear structural 1 | 0.020638 |
| ENSG00000151466 | SCLT1 | Sodium channel and clathrin linker 1 | 0.021023 |
| ENSG00000104059 | FAM189A1 | Family with sequence similarity 189 member A1 | 0.02128 |
| ENSG00000102230 | PCYT1B | Phosphate cytidylyltransferase 1, choline, beta | 0.021392 |
| ENSG00000114405 | C3orf14 | Chromosome 3 open reading frame 14 | 0.021959 |
| ENSG00000147874 | HAUS6 | HAUS augmin like complex subunit 6 | 0.022358 |
| ENSG00000183624 | HMCES | 5-hydroxymethylcytosine binding, ES cell specific | 0.022527 |
| ENSG00000179241 | LDLRAD3 | Low density lipoprotein receptor class A domain containing 3 | 0.022638 |
| ENSG00000128708 | HAT1 | Histone acetyltransferase 1 | 0.022648 |
| ENSG00000105968 | H2AZ2 | H2A.Z variant histone 2 | 0.022686 |
| ENSG00000183207 | RUVBL2 | Ruvb like AAA atpase 2 | 0.022768 |
| ENSG00000104267 | CA2 | Carbonic anhydrase 2 | 0.023145 |
| ENSG00000020922 | MRE11 | MRE11 homolog, double strand break repair nuclease | 0.023578 |
| ENSG00000189362 | NEMP2 | Nuclear envelope integral membrane protein 2 | 0.024134 |
| ENSG00000170264 | FAM161A | FAM161 centrosomal protein A | 0.024257 |
| ENSG00000106571 | GLI3 | GLI family zinc finger 3 | 0.024473 |

| | | | |
|---|---|---|---|
| ENSG00000136159 | NUDT15 | Nudix hydrolase 15 | 0.024473 |
| ENSG00000114541 | FRMD4B | FERM domain containing 4B | 0.027076 |
| ENSG00000197771 | MCMBP | Minichromosome maintenance complex binding protein | 0.027145 |
| ENSG00000213390 | ARHGAP19 | Rho gtpase activating protein 19 | 0.027612 |
| ENSG00000198039 | ZNF273 | Zinc finger protein 273 | 0.028146 |
| ENSG00000249115 | HAUS5 | HAUS augmin like complex subunit 5 | 0.029503 |
| ENSG00000107672 | NSMCE4A | NSE4 homolog A, SMC5-SMC6 complex component | 0.032497 |
| ENSG00000136938 | ANP32B | Acidic nuclear phosphoprotein 32 family member B | 0.033534 |
| ENSG00000183148 | ANKRD20A2 | Ankyrin repeat domain 20 family member A2 | 0.033804 |
| ENSG00000186522 | SEPTIN10 | Septin 10 | 0.033966 |
| ENSG00000286132 | AC022415.3 | Novel protein | 0.033993 |
| ENSG00000169508 | GPR183 | G protein-coupled receptor 183 | 0.034006 |
| ENSG00000241634 | AC069499.2 | Ribosomal protein L13 (RPL13) pseudogene | 0.035083 |
| ENSG00000198168 | SVIP | Small VCP interacting protein | 0.035464 |
| ENSG00000107105 | ELAVL2 | ELAV like RNA binding protein 2 | 0.035771 |
| ENSG00000130816 | DNMT1 | DNA methyltransferase 1 | 0.036655 |
| ENSG00000134758 | RNF138 | Ring finger protein 138 | 0.037618 |
| ENSG00000146426 | TIAM2 | TIAM Rac1 associated GEF 2 | 0.038632 |
| ENSG00000121988 | ZRANB3 | Zinc finger RANBP2-type containing 3 | 0.040433 |
| ENSG00000280571 | AC006059.2 | Novel protein | 0.040631 |
| ENSG00000161547 | SRSF2 | Serine and arginine rich splicing factor 2 | 0.041157 |
| ENSG00000126878 | AIF1L | Allograft inflammatory factor 1 like | 0.04171 |
| ENSG00000056050 | HPF1 | Histone parylation factor 1 | 0.041956 |
| ENSG00000104889 | RNASEH2A | Ribonuclease H2 subunit A | 0.043069 |
| ENSG00000184270 | H2AC21 | H2A clustered histone 21 | 0.043394 |
| ENSG00000203814 | H2BC18 | H2B clustered histone 18 | 0.043794 |
| ENSG00000181588 | MEX3D | Mex-3 RNA binding family member D | 0.04415 |
| ENSG00000171960 | PPIH | Peptidylprolyl isomerase H | 0.044159 |
| ENSG00000198155 | ZNF876P | Zinc finger protein 876, pseudogene | 0.044573 |
| ENSG00000170037 | CNTROB | Centrobin, centriole duplication and spindle assembly protein | 0.044584 |
| ENSG00000260802 | SERTM2 | Serine rich and transmembrane domain containing 2 | 0.04481 |
| ENSG00000183798 | EMILIN3 | Elastin microfibril interfacer 3 | 0.044836 |
| ENSG00000104177 | MYEF2 | Myelin expression factor 2 | 0.045202 |
| ENSG00000180998 | GPR137C | G protein-coupled receptor 137C | 0.045294 |
| ENSG00000096654 | ZNF184 | Zinc finger protein 184 | 0.045418 |
| ENSG00000118412 | CASP8AP2 | Caspase 8 associated protein 2 | 0.046588 |
| ENSG00000149639 | SOGA1 | Suppressor of glucose, autophagy associated 1 | 0.047252 |
| ENSG00000118513 | MYB | MYB proto-oncogene, transcription factor | 0.048162 |
| ENSG00000204410 | MSH5 | Muts homolog 5 | 0.048907 |
| ENSG00000160201 | U2AF1 | U2 small nuclear RNA auxiliary factor 1 | 0.049099 |
| ENSG00000144677 | CTDSPL | CTD small phosphatase like | 0.049262 |
| ENSG00000087301 | TXNDC16 | Thioredoxin domain containing 16 | 0.049712 |

**Supplementary Table S2.** Statistically significant DEGs (Adj.*p*-value<0.05) derived from DGEA of RNA-Seq data from Primary Human Lung Fibroblasts (IMR90) after X-ray irradiation, using DESeq2. The experiment consists of 3 control samples and 3 irradiated with 2 Gy X-ray radiation for 24 hours [Bioproject: PRJNA436999].

| **Ensembl ID** | **Gene Symbol** | **Gene Description** | **Adj. *p*-Value** |
|---|---|---|---|
| | | **Up-Regulated Genes↑** | |
| ENSG00000124762 | CDKN1A | Cyclin dependent kinase inhibitor 1A | 1.17E-83 |
| ENSG00000163071 | SPATA18 | Spermatogenesis associated 18 | 3.10E-38 |
| ENSG00000173530 | TNFRSF10D | TNF receptor superfamily member 10d | 5.93E-29 |
| ENSG00000181026 | AEN | Apoptosis enhancing nuclease | 7.02E-28 |
| ENSG00000055163 | CYFIP2 | Cytoplasmic FMR1 interacting protein 2 | 1.83E-27 |
| ENSG00000161513 | FDXR | Ferredoxin reductase | 7.58E-27 |
| ENSG00000130513 | GDF15 | Growth differentiation factor 15 | 3.66E-25 |
| ENSG00000087088 | BAX | BCL2 associated X, apoptosis regulator | 7.75E-25 |
| ENSG00000105327 | BBC3 | BCL2 binding component 3 | 8.07E-22 |
| ENSG00000131080 | EDA2R | Ectodysplasin A2 receptor | 4.88E-21 |
| ENSG00000110900 | TSPAN11 | Tetraspanin 11 | 2.57E-20 |
| ENSG00000120889 | TNFRSF10B | TNF receptor superfamily member 10b | 2.82E-20 |
| ENSG00000134574 | DDB2 | Damage specific DNA binding protein 2 | 5.11E-19 |
| ENSG00000132274 | TRIM22 | Tripartite motif containing 22 | 4.48E-17 |
| ENSG00000080546 | SESN1 | Sestrin 1 | 1.39E-14 |
| ENSG00000180155 | LYNX1 | Ly6/neurotoxin 1 | 3.06E-14 |
| ENSG00000167196 | FBXO22 | F-box protein 22 | 9.81E-14 |
| ENSG00000154767 | XPC | XPC complex subunit, DNA damage recognition and repair factor | 1.90E-13 |
| ENSG00000174307 | PHLDA3 | Pleckstrin homology like domain family A member 3 | 6.02E-13 |
| ENSG00000112249 | ASCC3 | Activating signal cointegrator 1 complex subunit 3 | 3.45E-12 |
| ENSG00000196562 | SULF2 | Sulfatase 2 | 5.29E-12 |
| ENSG00000172667 | ZMAT3 | Zinc finger matrin-type 3 | 3.93E-11 |
| ENSG00000173535 | TNFRSF10C | TNF receptor superfamily member 10c | 7.97E-11 |
| ENSG00000142627 | EPHA2 | EPH receptor A2 | 8.93E-11 |
| ENSG00000026103 | FAS | Fas cell surface death receptor | 5.97E-10 |
| ENSG00000228430 | AL162726.3 | Serine palmitoyltransferase long chain base subunit 1 (SPTLC1) pseudogene | 5.24E-09 |
| ENSG00000148175 | STOM | Stomatin | 9.52E-09 |
| ENSG00000135919 | SERPINE2 | Serpin family E member 2 | 1.07E-08 |
| ENSG00000170855 | TRIAP1 | TP53 regulated inhibitor of apoptosis 1 | 1.51E-08 |
| ENSG00000177106 | EPS8L2 | EPS8 like 2 | 1.97E-08 |
| ENSG00000128422 | KRT17 | Keratin 17 | 2.02E-08 |
| ENSG00000130766 | SESN2 | Sestrin 2 | 4.04E-08 |
| ENSG00000054148 | PHPT1 | Phosphohistidine phosphatase 1 | 7.49E-08 |
| ENSG00000177943 | MAMDC4 | MAM domain containing 4 | 1.02E-07 |
| ENSG00000051108 | HERPUD1 | Homocysteine inducible ER protein with ubiquitin like domain 1 | 1.14E-07 |
| ENSG00000171246 | NPTX1 | Neuronal pentraxin 1 | 1.98E-07 |
| ENSG00000112715 | VEGFA | Vascular endothelial growth factor A | 3.01E-07 |
| ENSG00000159388 | BTG2 | BTG anti-proliferation factor 2 | 3.74E-07 |
| ENSG00000141232 | TOB1 | Transducer of ERBB2, 1 | 4.46E-07 |
| ENSG00000176697 | BDNF | Brain derived neurotrophic factor | 1.77E-06 |
| ENSG00000173846 | PLK3 | Polo like kinase 3 | 1.96E-06 |

| | | | |
|---|---|---|---|
| ENSG00000182247 | UBE2E2 | Ubiquitin conjugating enzyme E2 E2 | 2.10E-06 |
| ENSG00000048392 | RRM2B | Ribonucleotide reductase regulatory TP53 inducible subunit M2B | 4.60E-06 |
| ENSG00000011114 | BTBD7 | BTB domain containing 7 | 5.28E-06 |
| ENSG00000168621 | GDNF | Glial cell derived neurotrophic factor | 5.35E-06 |
| ENSG00000197093 | GAL3ST4 | Galactose-3-O-sulfotransferase 4 | 6.71E-06 |
| ENSG00000165072 | MAMDC2 | MAM domain containing 2 | 6.71E-06 |
| ENSG00000163735 | CXCL5 | C-X-C motif chemokine ligand 5 | 6.71E-06 |
| ENSG00000196975 | ANXA4 | Annexin A4 | 8.08E-06 |
| ENSG00000178607 | ERN1 | Endoplasmic reticulum to nucleus signaling 1 | 1.09E-05 |
| ENSG00000162643 | WDR63 | WD repeat domain 63 | 1.70E-05 |
| ENSG00000113328 | CCNG1 | Cyclin G1 | 1.76E-05 |
| ENSG00000138166 | DUSP5 | Dual specificity phosphatase 5 | 2.22E-05 |
| ENSG00000185088 | RPS27L | Ribosomal protein S27 like | 2.45E-05 |
| ENSG00000182752 | PAPPA | Pappalysin 1 | 2.64E-05 |
| ENSG00000113108 | APBB3 | Amyloid beta precursor protein binding family B member 3 | 3.20E-05 |
| ENSG00000059804 | SLC2A3 | Solute carrier family 2 member 3 | 3.41E-05 |
| ENSG00000244509 | APOBEC3C | Apolipoprotein B mrna editing enzyme catalytic subunit 3C | 5.14E-05 |
| ENSG00000124466 | LYPD3 | LY6/PLAUR domain containing 3 | 6.25E-05 |
| ENSG00000104689 | TNFRSF10A | TNF receptor superfamily member 10a | 6.46E-05 |
| ENSG00000277639 | AC007906.2 | Novel protein | 7.16E-05 |
| ENSG00000116574 | RHOU | Ras homolog family member U | 7.34E-05 |
| ENSG00000169429 | CXCL8 | C-X-C motif chemokine ligand 8 | 7.42E-05 |
| ENSG00000116183 | PAPPA2 | Pappalysin 2 | 7.89E-05 |
| ENSG00000160691 | SHC1 | SHC adaptor protein 1 | 8.01E-05 |
| ENSG00000182580 | EPHB3 | EPH receptor B3 | 9.16E-05 |
| ENSG00000197872 | FAM49A | Family with sequence similarity 49 member A | 0.000157 |
| ENSG00000244694 | PTCHD4 | Patched domain containing 4 | 0.000157 |
| ENSG00000168918 | INPP5D | Inositol polyphosphate-5-phosphatase D | 0.000158 |
| ENSG00000065491 | TBC1D22B | TBC1 domain family member 22B | 0.000162 |
| ENSG00000103257 | SLC7A5 | Solute carrier family 7 member 5 | 0.000169 |
| ENSG00000101928 | MOSPD1 | Motile sperm domain containing 1 | 0.000182 |
| ENSG00000136244 | IL6 | Interleukin 6 | 0.000211 |
| ENSG00000196152 | ZNF79 | Zinc finger protein 79 | 0.000221 |
| ENSG00000105372 | RPS19 | Ribosomal protein S19 | 0.000222 |
| ENSG00000197632 | SERPINB2 | Serpin family B member 2 | 0.000301 |
| ENSG00000163734 | CXCL3 | C-X-C motif chemokine ligand 3 | 0.000325 |
| ENSG00000105649 | RAB3A | RAB3A, member RAS oncogene family | 0.000326 |
| ENSG00000225511 | LINC00475 | Long intergenic non-protein coding RNA 475 | 0.000345 |
| ENSG00000125538 | IL1B | Interleukin 1 beta | 0.000353 |
| ENSG00000163739 | CXCL1 | C-X-C motif chemokine ligand 1 | 0.000369 |
| ENSG00000124098 | FAM210B | Family with sequence similarity 210 member B | 0.000386 |
| ENSG00000198064 | NPIPB13 | Nuclear pore complex interacting protein family, member B13 | 0.000395 |
| ENSG00000081041 | CXCL2 | C-X-C motif chemokine ligand 2 | 0.000443 |
| ENSG00000164938 | TP53INP1 | Tumor protein p53 inducible nuclear protein 1 | 0.000451 |
| ENSG00000116761 | CTH | Cystathionine gamma-lyase | 0.000466 |
| ENSG00000140379 | BCL2A1 | BCL2 related protein A1 | 0.000486 |
| ENSG00000168209 | DDIT4 | DNA damage inducible transcript 4 | 0.000509 |

| | | | |
|---|---|---|---|
| ENSG00000158050 | DUSP2 | Dual specificity phosphatase 2 | 0.000558 |
| ENSG00000132256 | TRIM5 | Tripartite motif containing 5 | 0.000573 |
| ENSG00000135679 | MDM2 | MDM2 proto-oncogene | 0.000593 |
| ENSG00000112773 | TENT5A | Terminal nucleotidyltransferase 5A | 0.000594 |
| ENSG00000144476 | ACKR3 | Atypical chemokine receptor 3 | 0.000595 |
| ENSG00000115008 | IL1A | Interleukin 1 alpha | 0.000596 |
| ENSG00000255112 | CHMP1B | Charged multivesicular body protein 1B | 0.000621 |
| ENSG00000172890 | NADSYN1 | NAD synthetase 1 | 0.000626 |
| ENSG00000116741 | RGS2 | Regulator of G protein signaling 2 | 0.000658 |
| ENSG00000123358 | NR4A1 | Nuclear receptor subfamily 4 group A member 1 | 0.000684 |
| ENSG00000272921 | AC005832.4 | Novel protein | 0.000887 |
| ENSG00000104613 | INTS10 | Integrator complex subunit 10 | 0.00091 |
| ENSG00000068912 | ERLEC1 | Endoplasmic reticulum lectin 1 | 0.000989 |
| ENSG00000013588 | GPRC5A | G protein-coupled receptor class C group 5 member A | 0.001023 |
| ENSG00000185112 | FAM43A | Family with sequence similarity 43 member A | 0.001062 |
| ENSG00000163659 | TIPARP | TCDD inducible poly(ADP-ribose) polymerase | 0.001064 |
| ENSG00000138685 | FGF2 | Fibroblast growth factor 2 | 0.001151 |
| ENSG00000111817 | DSE | Dermatan sulfate epimerase | 0.001176 |
| ENSG00000168497 | CAVIN2 | Caveolae associated protein 2 | 0.00125 |
| ENSG00000108342 | CSF3 | Colony stimulating factor 3 | 0.00125 |
| ENSG00000112697 | TMEM30A | Transmembrane protein 30A | 0.001326 |
| ENSG00000184205 | TSPYL2 | TSPY like 2 | 0.001374 |
| ENSG00000065357 | DGKA | Diacylglycerol kinase alpha | 0.001546 |
| ENSG00000136826 | KLF4 | Kruppel like factor 4 | 0.001598 |
| ENSG00000196576 | PLXNB2 | Plexin B2 | 0.001926 |
| ENSG00000171033 | PKIA | Camp-dependent protein kinase inhibitor alpha | 0.002065 |
| ENSG00000137819 | PAQR5 | Progestin and adipoq receptor family member 5 | 0.002151 |
| ENSG00000095794 | CREM | Camp responsive element modulator | 0.002188 |
| ENSG00000179094 | PER1 | Period circadian regulator 1 | 0.002197 |
| ENSG00000146592 | CREB5 | Camp responsive element binding protein 5 | 0.00231 |
| ENSG00000088826 | SMOX | Spermine oxidase | 0.002351 |
| ENSG00000087589 | CASS4 | Cas scaffold protein family member 4 | 0.002384 |
| ENSG00000106004 | HOXA5 | Homeobox A5 | 0.00253 |
| ENSG00000128590 | DNAJB9 | Dnaj heat shock protein family (Hsp40) member B9 | 0.002562 |
| ENSG00000184588 | PDE4B | Phosphodiesterase 4B | 0.002629 |
| ENSG00000001626 | CFTR | CF transmembrane conductance regulator | 0.002785 |
| ENSG00000176907 | TCIM | Transcriptional and immune response regulator | 0.002798 |
| ENSG00000070540 | WIPI1 | WD repeat domain, phosphoinositide interacting 1 | 0.002885 |
| ENSG00000154319 | FAM167A | Family with sequence similarity 167 member A | 0.003048 |
| ENSG00000073756 | PTGS2 | Prostaglandin-endoperoxide synthase 2 | 0.003157 |
| ENSG00000285976 | AL135905.2 | Novel protein | 0.003404 |
| ENSG00000256206 | AC018523.2 | Novel protein | 0.003457 |
| ENSG00000130066 | SAT1 | Spermidine/spermine N1-acetyltransferase 1 | 0.003582 |
| ENSG00000104970 | KIR3DX1 | Killer cell immunoglobulin like receptor, three Ig domains X1 (pseudogene) | 0.003582 |
| ENSG00000121039 | RDH10 | Retinol dehydrogenase 10 | 0.003664 |
| ENSG00000171219 | CDC42BPG | CDC42 binding protein kinase gamma | 0.003842 |
| ENSG00000134107 | BHLHE40 | Basic helix-loop-helix family member e40 | 0.00399 |
| ENSG00000100739 | BDKRB1 | Bradykinin receptor B1 | 0.003994 |
| ENSG00000143507 | DUSP10 | Dual specificity phosphatase 10 | 0.003994 |
| ENSG00000145040 | UCN2 | Urocortin 2 | 0.004269 |

| | | | |
|---|---|---|---|
| ENSG00000167992 | VWCE | Von Willebrand factor C and EGF domains | 0.004289 |
| ENSG00000100906 | NFKBIA | NFKB inhibitor alpha | 0.004488 |
| ENSG00000169594 | BNC1 | Basonuclin 1 | 0.0052 |
| ENSG00000102804 | TSC22D1 | TSC22 domain family member 1 | 0.00534 |
| ENSG00000124216 | SNAI1 | Snail family transcriptional repressor 1 | 0.005595 |
| ENSG00000122484 | RPAP2 | RNA polymerase II associated protein 2 | 0.005767 |
| ENSG00000164237 | CMBL | Carboxymethylenebutenolidase homolog | 0.005945 |
| ENSG00000128342 | LIF | LIF interleukin 6 family cytokine | 0.00626 |
| ENSG00000115421 | PAPOLG | Poly(A) polymerase gamma | 0.007146 |
| ENSG00000175600 | SUGCT | Succinyl-coa:glutarate-coa transferase | 0.00734 |
| ENSG00000188243 | COMMD6 | COMM domain containing 6 | 0.007405 |
| ENSG00000104321 | TRPA1 | Transient receptor potential cation channel subfamily A member 1 | 0.007434 |
| ENSG00000091592 | NLRP1 | NLR family pyrin domain containing 1 | 0.007483 |
| ENSG00000101255 | TRIB3 | Tribbles pseudokinase 3 | 0.007787 |
| ENSG00000003989 | SLC7A2 | Solute carrier family 7 member 2 | 0.008206 |
| ENSG00000142178 | SIK1 | Salt inducible kinase 1 | 0.008358 |
| ENSG00000170836 | PPM1D | Protein phosphatase, Mg2+/Mn2+ dependent 1D | 0.008422 |
| ENSG00000176531 | PHLDB3 | Pleckstrin homology like domain family B member 3 | 0.008947 |
| ENSG00000075413 | MARK3 | Microtubule affinity regulating kinase 3 | 0.009249 |
| ENSG00000137975 | CLCA2 | Chloride channel accessory 2 | 0.009424 |
| ENSG00000042445 | RETSAT | Retinol saturase | 0.009564 |
| ENSG00000130844 | ZNF331 | Zinc finger protein 331 | 0.00959 |
| ENSG00000088340 | FER1L4 | Fer-1 like family member 4 (pseudogene) | 0.010024 |
| ENSG00000165272 | AQP3 | Aquaporin 3 (Gill blood group) | 0.010913 |
| ENSG00000163638 | ADAMTS9 | ADAM metallopeptidase with thrombospondin type 1 motif 9 | 0.010973 |
| ENSG00000124882 | EREG | Epiregulin | 0.011037 |
| ENSG00000078237 | TIGAR | TP53 induced glycolysis regulatory phosphatase | 0.011078 |
| ENSG00000114270 | COL7A1 | Collagen type VII alpha 1 chain | 0.011081 |
| ENSG00000124875 | CXCL6 | C-X-C motif chemokine ligand 6 | 0.011613 |
| ENSG00000204920 | ZNF155 | Zinc finger protein 155 | 0.011786 |
| ENSG00000134363 | FST | Follistatin | 0.011789 |
| ENSG00000103253 | HAGHL | Hydroxyacylglutathione hydrolase like | 0.011802 |
| ENSG00000145632 | PLK2 | Polo like kinase 2 | 0.011821 |
| ENSG00000148344 | PTGES | Prostaglandin E synthase | 0.011821 |
| ENSG00000144655 | CSRNP1 | Cysteine and serine rich nuclear protein 1 | 0.011836 |
| ENSG00000162496 | DHRS3 | Dehydrogenase/reductase 3 | 0.01195 |
| ENSG00000171223 | JUNB | Junb proto-oncogene, AP-1 transcription factor subunit | 0.012326 |
| ENSG00000255730 | AC011462.1 | Novel protein | 0.012385 |
| ENSG00000153234 | NR4A2 | Nuclear receptor subfamily 4 group A member 2 | 0.012396 |
| ENSG00000104635 | SLC39A14 | Solute carrier family 39 member 14 | 0.012936 |
| ENSG00000152049 | KCNE4 | Potassium voltage-gated channel subfamily E regulatory subunit 4 | 0.013231 |
| ENSG00000188234 | AGAP4 | Arfgap with gtpase domain, ankyrin repeat and PH domain 4 | 0.013316 |
| ENSG00000115009 | CCL20 | C-C motif chemokine ligand 20 | 0.013408 |
| ENSG00000132510 | KDM6B | Lysine demethylase 6B | 0.0135 |
| ENSG00000151065 | DCP1B | Decapping mrna 1B | 0.013627 |
| ENSG00000177595 | PIDD1 | P53-induced death domain protein 1 | 0.013665 |
| ENSG00000165312 | OTUD1 | OTU deubiquitinase 1 | 0.014166 |

| | | | |
|---|---|---|---|
| ENSG00000186866 | POFUT2 | Protein O-fucosyltransferase 2 | 0.014758 |
| ENSG00000099875 | MKNK2 | MAPK interacting serine/threonine kinase 2 | 0.014866 |
| ENSG00000164331 | ANKRA2 | Ankyrin repeat family A member 2 | 0.015052 |
| ENSG00000124067 | SLC12A4 | Solute carrier family 12 member 4 | 0.015183 |
| ENSG00000156976 | EIF4A2 | Eukaryotic translation initiation factor 4A2 | 0.015304 |
| ENSG00000125772 | GPCPD1 | Glycerophosphocholine phosphodiesterase 1 | 0.015445 |
| ENSG00000198001 | AC093012.1 | Interleukin 1 receptor associated kinase 4 | 0.015605 |
| ENSG00000013441 | CLK1 | CDC like kinase 1 | 0.015605 |
| ENSG00000143333 | RGS16 | Regulator of G protein signaling 16 | 0.015635 |
| ENSG00000224877 | NDUFAF8 | NADH:ubiquinone oxidoreductase complex assembly factor 8 | 0.015678 |
| ENSG00000204219 | TCEA3 | Transcription elongation factor A3 | 0.015678 |
| ENSG00000173230 | GOLGB1 | Golgin B1 | 0.01621 |
| ENSG00000123610 | TNFAIP6 | TNF alpha induced protein 6 | 0.016961 |
| ENSG00000121361 | KCNJ8 | Potassium inwardly rectifying channel subfamily J member 8 | 0.017063 |
| ENSG00000056972 | TRAF3IP2 | TRAF3 interacting protein 2 | 0.01728 |
| ENSG00000103742 | IGDCC4 | Immunoglobulin superfamily DCC subclass member 4 | 0.017285 |
| ENSG00000110944 | IL23A | Interleukin 23 subunit alpha | 0.018741 |
| ENSG00000186340 | THBS2 | Thrombospondin 2 | 0.019515 |
| ENSG00000139112 | GABARAPL1 | GABA type A receptor associated protein like 1 | 0.019696 |
| ENSG00000139636 | LMBR1L | Limb development membrane protein 1 like | 0.020848 |
| ENSG00000259529 | AL136295.5 | Novel transcript | 0.02178 |
| ENSG00000128052 | KDR | Kinase insert domain receptor | 0.022347 |
| ENSG00000002745 | WNT16 | Wnt family member 16 | 0.022521 |
| ENSG00000279483 | AC090498.1 | Ribosomal protein L41 (RPL41) pseudogene | 0.022677 |
| ENSG00000164125 | GASK1B | Golgi associated kinase 1B | 0.022743 |
| ENSG00000140285 | FGF7 | Fibroblast growth factor 7 | 0.023727 |
| ENSG00000137500 | CCDC90B | Coiled-coil domain containing 90B | 0.023889 |
| ENSG00000120129 | DUSP1 | Dual specificity phosphatase 1 | 0.024747 |
| ENSG00000160746 | ANO10 | Anoctamin 10 | 0.024981 |
| ENSG00000114841 | DNAH1 | Dynein axonemal heavy chain 1 | 0.026204 |
| ENSG00000172216 | CEBPB | CCAAT enhancer binding protein beta | 0.02658 |
| ENSG00000119729 | RHOQ | Ras homolog family member Q | 0.026876 |
| ENSG00000141337 | ARSG | Arylsulfatase G | 0.027492 |
| ENSG00000099625 | CBARP | CACN subunit beta associated regulatory protein | 0.027492 |
| ENSG00000099860 | GADD45B | Growth arrest and DNA damage inducible beta | 0.028408 |
| ENSG00000184319 | RPL23AP82 | Ribosomal protein L23a pseudogene 82 | 0.028908 |
| ENSG00000182220 | ATP6AP2 | Atpase H+ transporting accessory protein 2 | 0.029037 |
| ENSG00000111275 | ALDH2 | Aldehyde dehydrogenase 2 family member | 0.029527 |
| ENSG00000162772 | ATF3 | Activating transcription factor 3 | 0.029783 |
| ENSG00000164181 | ELOVL7 | ELOVL fatty acid elongase 7 | 0.029969 |
| ENSG00000204815 | TTC25 | Tetratricopeptide repeat domain 25 | 0.030457 |
| ENSG00000152229 | PSTPIP2 | Proline-serine-threonine phosphatase interacting protein 2 | 0.030546 |
| ENSG00000172738 | TMEM217 | Transmembrane protein 217 | 0.030546 |
| ENSG00000078142 | PIK3C3 | Phosphatidylinositol 3-kinase catalytic subunit type 3 | 0.030698 |
| ENSG00000135452 | TSPAN31 | Tetraspanin 31 | 0.030926 |
| ENSG00000140945 | CDH13 | Cadherin 13 | 0.031045 |
| ENSG00000243970 | PPIEL | Peptidylprolyl isomerase E like pseudogene | 0.031437 |
| ENSG00000228463 | AP006222.1 | Ribosomal protein L23a (RPL23A) pseudogene | 0.032863 |

| | | | |
|---|---|---|---|
| ENSG00000153714 | LURAP1L | Leucine rich adaptor protein 1 like | 0.033138 |
| ENSG00000187479 | C11orf96 | Chromosome 11 open reading frame 96 | 0.033138 |
| ENSG00000105928 | GSDME | Gasdermin E | 0.033901 |
| ENSG00000184557 | SOCS3 | Suppressor of cytokine signaling 3 | 0.034506 |
| ENSG00000119899 | SLC17A5 | Solute carrier family 17 member 5 | 0.035611 |
| ENSG00000149968 | MMP3 | Matrix metallopeptidase 3 | 0.036731 |
| ENSG00000115107 | STEAP3 | STEAP3 metalloreductase | 0.037274 |
| ENSG00000196072 | BLOC1S2 | Biogenesis of lysosomal organelles complex 1 subunit 2 | 0.037385 |
| ENSG00000162004 | CCDC78 | Coiled-coil domain containing 78 | 0.038281 |
| ENSG00000083097 | DOP1A | DOP1 leucine zipper like protein A | 0.039387 |
| ENSG00000120669 | SOHLH2 | Spermatogenesis and oogenesis specific basic helix-loop-helix 2 | 0.039778 |
| ENSG00000044574 | HSPA5 | Heat shock protein family A (Hsp70) member 5 | 0.040279 |
| ENSG00000100647 | SUSD6 | Sushi domain containing 6 | 0.041242 |
| ENSG00000138449 | SLC40A1 | Solute carrier family 40 member 1 | 0.041272 |
| ENSG00000126785 | RHOJ | Ras homolog family member J | 0.041803 |
| ENSG00000132793 | LPIN3 | Lipin 3 | 0.042514 |
| ENSG00000139874 | SSTR1 | Somatostatin receptor 1 | 0.042888 |
| ENSG00000134070 | IRAK2 | Interleukin 1 receptor associated kinase 2 | 0.043333 |
| ENSG00000206561 | COLQ | Collagen like tail subunit of asymmetric acetylcholinesterase | 0.045497 |
| ENSG00000150681 | RGS18 | Regulator of G protein signaling 18 | 0.046018 |
| ENSG00000149428 | HYOU1 | Hypoxia up-regulated 1 | 0.047357 |
| ENSG00000109323 | MANBA | Mannosidase beta | 0.047357 |
| ENSG00000091129 | NRCAM | Neuronal cell adhesion molecule | 0.047357 |
| ENSG00000100298 | APOBEC3H | Apolipoprotein B mrna editing enzyme catalytic subunit 3H | 0.047536 |
| ENSG00000198722 | UNC13B | Unc-13 homolog B | 0.047741 |
| ENSG00000113504 | SLC12A7 | Solute carrier family 12 member 7 | 0.048192 |
| **Down-Regulated Genes↓** | | | |
| ENSG00000111665 | CDCA3 | Cell division cycle associated 3 | 2.18E-103 |
| ENSG00000148773 | MKI67 | Marker of proliferation Ki-67 | 2.13E-87 |
| ENSG00000164104 | HMGB2 | High mobility group box 2 | 1.47E-69 |
| ENSG00000123485 | HJURP | Holliday junction recognition protein | 4.95E-68 |
| ENSG00000178999 | AURKB | Aurora kinase B | 1.18E-65 |
| ENSG00000117399 | CDC20 | Cell division cycle 20 | 4.35E-65 |
| ENSG00000101057 | MYBL2 | MYB proto-oncogene like 2 | 3.61E-63 |
| ENSG00000105011 | ASF1B | Anti-silencing function 1B histone chaperone | 3.87E-63 |
| ENSG00000127564 | PKMYT1 | Protein kinase, membrane associated tyrosine/threonine 1 | 7.64E-62 |
| ENSG00000013810 | TACC3 | Transforming acidic coiled-coil containing protein 3 | 7.96E-62 |
| ENSG00000134690 | CDCA8 | Cell division cycle associated 8 | 2.18E-61 |
| ENSG00000145386 | CCNA2 | Cyclin A2 | 3.15E-61 |
| ENSG00000171848 | RRM2 | Ribonucleotide reductase regulatory subunit M2 | 2.90E-59 |
| ENSG00000075218 | GTSE1 | G2 and S-phase expressed 1 | 2.96E-59 |
| ENSG00000113368 | LMNB1 | Lamin B1 | 7.27E-58 |
| ENSG00000146670 | CDCA5 | Cell division cycle associated 5 | 1.05E-57 |
| ENSG00000166851 | PLK1 | Polo like kinase 1 | 1.53E-54 |
| ENSG00000237649 | KIFC1 | Kinesin family member C1 | 2.60E-54 |
| ENSG00000011426 | ANLN | Anillin actin binding protein | 2.81E-54 |
| ENSG00000162063 | CCNF | Cyclin F | 1.05E-53 |
| ENSG00000175063 | UBE2C | Ubiquitin conjugating enzyme E2 C | 3.42E-53 |

| | | | |
|---|---|---|---|
| ENSG00000111206 | FOXM1 | Forkhead box M1 | 1.39E-51 |
| ENSG00000101447 | FAM83D | Family with sequence similarity 83 member D | 1.88E-51 |
| ENSG00000161888 | SPC24 | SPC24 component of NDC80 kinetochore complex | 5.98E-50 |
| ENSG00000088325 | TPX2 | TPX2 microtubule nucleation factor | 1.48E-49 |
| ENSG00000137804 | NUSAP1 | Nucleolar and spindle associated protein 1 | 6.72E-49 |
| ENSG00000186185 | KIF18B | Kinesin family member 18B | 1.75E-48 |
| ENSG00000109805 | NCAPG | Non-SMC condensin I complex subunit G | 4.92E-48 |
| ENSG00000156970 | BUB1B | BUB1 mitotic checkpoint serine/threonine kinase B | 7.72E-48 |
| ENSG00000188486 | H2AX | H2A.X variant histone | 9.64E-48 |
| ENSG00000162073 | PAQR4 | Progestin and adipoq receptor family member 4 | 1.48E-47 |
| ENSG00000121152 | NCAPH | Non-SMC condensin I complex subunit H | 4.12E-47 |
| ENSG00000138180 | CEP55 | Centrosomal protein 55 | 6.03E-46 |
| ENSG00000170312 | CDK1 | Cyclin dependent kinase 1 | 1.50E-45 |
| ENSG00000126787 | DLGAP5 | DLG associated protein 5 | 1.77E-44 |
| ENSG00000165480 | SKA3 | Spindle and kinetochore associated complex subunit 3 | 4.20E-43 |
| ENSG00000137807 | KIF23 | Kinesin family member 23 | 5.31E-43 |
| ENSG00000122952 | ZWINT | ZW10 interacting kinetochore protein | 8.01E-43 |
| ENSG00000138160 | KIF11 | Kinesin family member 11 | 3.03E-42 |
| ENSG00000142945 | KIF2C | Kinesin family member 2C | 3.39E-42 |
| ENSG00000174371 | EXO1 | Exonuclease 1 | 1.54E-41 |
| ENSG00000073111 | MCM2 | Minichromosome maintenance complex component 2 | 1.93E-39 |
| ENSG00000198901 | PRC1 | Protein regulator of cytokinesis 1 | 3.11E-39 |
| ENSG00000213347 | MXD3 | MAX dimerization protein 3 | 1.97E-38 |
| ENSG00000276043 | UHRF1 | Ubiquitin like with PHD and ring finger domains 1 | 1.02E-37 |
| ENSG00000112029 | FBXO5 | F-box protein 5 | 1.05E-37 |
| ENSG00000165304 | MELK | Maternal embryonic leucine zipper kinase | 1.06E-37 |
| ENSG00000112984 | KIF20A | Kinesin family member 20A | 3.88E-37 |
| ENSG00000166508 | MCM7 | Minichromosome maintenance complex component 7 | 4.74E-37 |
| ENSG00000161800 | RACGAP1 | Rac gtpase activating protein 1 | 6.29E-37 |
| ENSG00000101412 | E2F1 | E2F transcription factor 1 | 1.48E-36 |
| ENSG00000168496 | FEN1 | Flap structure-specific endonuclease 1 | 4.66E-36 |
| ENSG00000080986 | NDC80 | NDC80 kinetochore complex component | 4.96E-36 |
| ENSG00000076382 | SPAG5 | Sperm associated antigen 5 | 6.41E-36 |
| ENSG00000183856 | IQGAP3 | IQ motif containing gtpase activating protein 3 | 1.37E-35 |
| ENSG00000169679 | BUB1 | BUB1 mitotic checkpoint serine/threonine kinase | 2.81E-35 |
| ENSG00000090889 | KIF4A | Kinesin family member 4A | 3.57E-35 |
| ENSG00000140534 | TICRR | TOPBP1 interacting checkpoint and replication regulator | 2.17E-34 |
| ENSG00000179750 | APOBEC3B | Apolipoprotein B mrna editing enzyme catalytic subunit 3B | 5.36E-34 |
| ENSG00000085840 | ORC1 | Origin recognition complex subunit 1 | 2.93E-33 |
| ENSG00000093009 | CDC45 | Cell division cycle 45 | 4.26E-33 |
| ENSG00000131747 | TOP2A | DNA topoisomerase II alpha | 1.01E-32 |
| ENSG00000186193 | SAPCD2 | Suppressor APC domain containing 2 | 1.25E-32 |
| ENSG00000120802 | TMPO | Thymopoietin | 1.31E-32 |
| ENSG00000228716 | DHFR | Dihydrofolate reductase | 3.65E-32 |
| ENSG00000158402 | CDC25C | Cell division cycle 25C | 9.50E-32 |
| ENSG00000189057 | FAM111B | Family with sequence similarity 111 member B | 1.94E-31 |
| ENSG00000135451 | TROAP | Trophinin associated protein | 2.11E-31 |
| ENSG00000129195 | PIMREG | PICALM interacting mitotic regulator | 3.73E-31 |
| ENSG00000131153 | GINS2 | GINS complex subunit 2 | 8.11E-31 |
| ENSG00000066279 | ASPM | Abnormal spindle microtubule assembly | 1.28E-30 |

| | | | |
|---|---|---|---|
| ENSG00000065328 | MCM10 | Minichromosome maintenance 10 replication initiation factor | 3.54E-30 |
| ENSG00000112118 | MCM3 | Minichromosome maintenance complex component 3 | 2.90E-29 |
| ENSG00000149503 | INCENP | Inner centromere protein | 6.53E-29 |
| ENSG00000089685 | BIRC5 | Baculoviral IAP repeat containing 5 | 1.61E-28 |
| ENSG00000071539 | TRIP13 | Thyroid hormone receptor interactor 13 | 1.71E-28 |
| ENSG00000143228 | NUF2 | NUF2 component of NDC80 kinetochore complex | 4.27E-28 |
| ENSG00000007968 | E2F2 | E2F transcription factor 2 | 8.22E-28 |
| ENSG00000129173 | E2F8 | E2F transcription factor 8 | 8.54E-28 |
| ENSG00000156802 | ATAD2 | Atpase family AAA domain containing 2 | 1.44E-27 |
| ENSG00000079616 | KIF22 | Kinesin family member 22 | 2.80E-27 |
| ENSG00000184661 | CDCA2 | Cell division cycle associated 2 | 5.96E-27 |
| ENSG00000115163 | CENPA | Centromere protein A | 7.58E-27 |
| ENSG00000151725 | CENPU | Centromere protein U | 7.72E-27 |
| ENSG00000092853 | CLSPN | Claspin | 2.14E-26 |
| ENSG00000101003 | GINS1 | GINS complex subunit 1 | 2.64E-26 |
| ENSG00000167670 | CHAF1A | Chromatin assembly factor 1 subunit A | 3.85E-26 |
| ENSG00000182481 | KPNA2 | Karyopherin subunit alpha 2 | 6.32E-26 |
| ENSG00000075702 | WDR62 | WD repeat domain 62 | 6.59E-26 |
| ENSG00000100162 | CENPM | Centromere protein M | 1.12E-25 |
| ENSG00000167513 | CDT1 | Chromatin licensing and DNA replication factor 1 | 3.94E-25 |
| ENSG00000170779 | CDCA4 | Cell division cycle associated 4 | 4.26E-25 |
| ENSG00000117724 | CENPF | Centromere protein F | 5.46E-25 |
| ENSG00000138778 | CENPE | Centromere protein E | 5.88E-25 |
| ENSG00000160957 | RECQL4 | Recq like helicase 4 | 1.38E-24 |
| ENSG00000165244 | ZNF367 | Zinc finger protein 367 | 1.45E-24 |
| ENSG00000187741 | FANCA | FA complementation group A | 5.08E-24 |
| ENSG00000117650 | NEK2 | NIMA related kinase 2 | 7.86E-24 |
| ENSG00000135476 | ESPL1 | Extra spindle pole bodies like 1, separase | 7.93E-24 |
| ENSG00000085999 | RAD54L | RAD54 like | 8.65E-24 |
| ENSG00000166803 | PCLAF | PCNA clamp associated factor | 8.74E-24 |
| ENSG00000144554 | FANCD2 | FA complementation group D2 | 1.32E-23 |
| ENSG00000112742 | TTK | TTK protein kinase | 1.37E-23 |
| ENSG00000035499 | DEPDC1B | DEP domain containing 1B | 2.45E-23 |
| ENSG00000094804 | CDC6 | Cell division cycle 6 | 5.15E-23 |
| ENSG00000122966 | CIT | Citron rho-interacting serine/threonine kinase | 1.10E-22 |
| ENSG00000123219 | CENPK | Centromere protein K | 1.35E-22 |
| ENSG00000169607 | CKAP2L | Cytoskeleton associated protein 2 like | 1.70E-22 |
| ENSG00000111247 | RAD51AP1 | RAD51 associated protein 1 | 2.14E-22 |
| ENSG00000167325 | RRM1 | Ribonucleotide reductase catalytic subunit M1 | 2.71E-22 |
| ENSG00000157456 | CCNB2 | Cyclin B2 | 4.45E-22 |
| ENSG00000168078 | PBK | PDZ binding kinase | 4.75E-22 |
| ENSG00000171241 | SHCBP1 | SHC binding and spindle associated 1 | 6.42E-22 |
| ENSG00000184445 | KNTC1 | Kinetochore associated 1 | 9.61E-22 |
| ENSG00000175305 | CCNE2 | Cyclin E2 | 1.74E-21 |
| ENSG00000092470 | WDR76 | WD repeat domain 76 | 3.30E-21 |
| ENSG00000137135 | ARHGEF39 | Rho guanine nucleotide exchange factor 39 | 6.74E-21 |
| ENSG00000143476 | DTL | Denticleless E3 ubiquitin protein ligase homolog | 1.14E-20 |
| ENSG00000134222 | PSRC1 | Proline and serine rich coiled-coil 1 | 1.20E-20 |
| ENSG00000051180 | RAD51 | RAD51 recombinase | 1.22E-20 |

| | | | |
|---|---|---|---|
| ENSG00000186871 | ERCC6L | ERCC excision repair 6 like, spindle assembly checkpoint helicase | 1.30E-20 |
| ENSG00000213551 | DNAJC9 | Dnaj heat shock protein family (Hsp40) member C9 | 1.42E-20 |
| ENSG00000133119 | RFC3 | Replication factor C subunit 3 | 1.53E-20 |
| ENSG00000106462 | EZH2 | Enhancer of zeste 2 polycomb repressive complex 2 subunit | 1.53E-20 |
| ENSG00000176619 | LMNB2 | Lamin B2 | 2.35E-20 |
| ENSG00000111602 | TIMELESS | Timeless circadian regulator | 2.53E-20 |
| ENSG00000149636 | DSN1 | DSN1 component of MIS12 kinetochore complex | 2.90E-20 |
| ENSG00000176890 | TYMS | Thymidylate synthetase | 3.70E-20 |
| ENSG00000131470 | PSMC3IP | PSMC3 interacting protein | 1.16E-19 |
| ENSG00000136108 | CKAP2 | Cytoskeleton associated protein 2 | 1.78E-19 |
| ENSG00000151503 | NCAPD3 | Non-SMC condensin II complex subunit D3 | 2.30E-19 |
| ENSG00000163808 | KIF15 | Kinesin family member 15 | 2.96E-19 |
| ENSG00000101945 | SUV39H1 | Suppressor of variegation 3-9 homolog 1 | 3.70E-19 |
| ENSG00000104889 | RNASEH2A | Ribonuclease H2 subunit A | 4.62E-19 |
| ENSG00000077152 | UBE2T | Ubiquitin conjugating enzyme E2 T | 6.14E-19 |
| ENSG00000159259 | CHAF1B | Chromatin assembly factor 1 subunit B | 6.46E-19 |
| ENSG00000029993 | HMGB3 | High mobility group box 3 | 1.71E-18 |
| ENSG00000068489 | PRR11 | Proline rich 11 | 2.43E-18 |
| ENSG00000198056 | PRIM1 | DNA primase subunit 1 | 2.85E-18 |
| ENSG00000177602 | HASPIN | Histone H3 associated protein kinase | 5.38E-18 |
| ENSG00000186638 | KIF24 | Kinesin family member 24 | 6.01E-18 |
| ENSG00000171320 | ESCO2 | Establishment of sister chromatid cohesion N-acetyltransferase 2 | 1.02E-17 |
| ENSG00000160949 | TONSL | Tonsoku like, DNA repair protein | 1.73E-17 |
| ENSG00000138182 | KIF20B | Kinesin family member 20B | 1.92E-17 |
| ENSG00000113810 | SMC4 | Structural maintenance of chromosomes 4 | 1.94E-17 |
| ENSG00000146918 | NCAPG2 | Non-SMC condensin II complex subunit G2 | 2.51E-17 |
| ENSG00000160298 | C21orf58 | Chromosome 21 open reading frame 58 | 3.49E-17 |
| ENSG00000198826 | ARHGAP11A | Rho gtpase activating protein 11A | 5.47E-17 |
| ENSG00000164087 | POC1A | POC1 centriolar protein A | 5.98E-17 |
| ENSG00000100297 | MCM5 | Minichromosome maintenance complex component 5 | 9.98E-17 |
| ENSG00000091651 | ORC6 | Origin recognition complex subunit 6 | 1.15E-16 |
| ENSG00000144354 | CDCA7 | Cell division cycle associated 7 | 1.18E-16 |
| ENSG00000127586 | CHTF18 | Chromosome transmission fidelity factor 18 | 1.41E-16 |
| ENSG00000123080 | CDKN2C | Cyclin dependent kinase inhibitor 2C | 1.60E-16 |
| ENSG00000138092 | CENPO | Centromere protein O | 2.72E-16 |
| ENSG00000176974 | SHMT1 | Serine hydroxymethyltransferase 1 | 3.69E-16 |
| ENSG00000131351 | HAUS8 | HAUS augmin like complex subunit 8 | 3.89E-16 |
| ENSG00000170540 | ARL6IP1 | ADP ribosylation factor like gtpase 6 interacting protein 1 | 4.24E-16 |
| ENSG00000134057 | CCNB1 | Cyclin B1 | 4.34E-16 |
| ENSG00000024526 | DEPDC1 | DEP domain containing 1 | 4.38E-16 |
| ENSG00000117632 | STMN1 | Stathmin 1 | 7.15E-16 |
| ENSG00000167900 | TK1 | Thymidine kinase 1 | 8.56E-16 |
| ENSG00000072571 | HMMR | Hyaluronan mediated motility receptor | 1.24E-15 |
| ENSG00000137812 | KNL1 | Kinetochore scaffold 1 | 1.48E-15 |
| ENSG00000284946 | AC068831.8 | Novel protein | 1.53E-15 |
| ENSG00000119333 | WDR34 | WD repeat domain 34 | 4.88E-15 |
| ENSG00000154839 | SKA1 | Spindle and kinetochore associated complex subunit 1 | 4.93E-15 |
| ENSG00000112312 | GMNN | Geminin DNA replication inhibitor | 6.13E-15 |

| | | | |
|---|---|---|---|
| ENSG00000198554 | WDHD1 | WD repeat and HMG-box DNA binding protein 1 | 6.42E-15 |
| ENSG00000104147 | OIP5 | Opa interacting protein 5 | 6.63E-15 |
| ENSG00000154146 | NRGN | Neurogranin | 1.05E-14 |
| ENSG00000164611 | PTTG1 | PTTG1 regulator of sister chromatid separation, securin | 1.45E-14 |
| ENSG00000149554 | CHEK1 | Checkpoint kinase 1 | 1.64E-14 |
| ENSG00000163507 | CIP2A | Cell proliferation regulating inhibitor of protein phosphatase 2A | 1.70E-14 |
| ENSG00000120539 | MASTL | Microtubule associated serine/threonine kinase like | 3.12E-14 |
| ENSG00000177084 | POLE | DNA polymerase epsilon, catalytic subunit | 5.11E-14 |
| ENSG00000196584 | XRCC2 | X-ray repair cross complementing 2 | 7.72E-14 |
| ENSG00000123473 | STIL | STIL centriolar assembly protein | 8.12E-14 |
| ENSG00000013573 | DDX11 | DEAD/H-box helicase 11 | 2.73E-13 |
| ENSG00000163918 | RFC4 | Replication factor C subunit 4 | 6.02E-13 |
| ENSG00000130816 | DNMT1 | DNA methyltransferase 1 | 6.33E-13 |
| ENSG00000109674 | NEIL3 | Nei like DNA glycosylase 3 | 7.28E-13 |
| ENSG00000185347 | TEDC1 | Tubulin epsilon and delta complex 1 | 7.42E-13 |
| ENSG00000101868 | POLA1 | DNA polymerase alpha 1, catalytic subunit | 1.14E-12 |
| ENSG00000214826 | DDX12P | DEAD/H-box helicase 12, pseudogene | 1.22E-12 |
| ENSG00000160447 | PKN3 | Protein kinase N3 | 1.34E-12 |
| ENSG00000136492 | BRIP1 | BRCA1 interacting protein C-terminal helicase 1 | 2.39E-12 |
| ENSG00000100526 | CDKN3 | Cyclin dependent kinase inhibitor 3 | 2.55E-12 |
| ENSG00000076003 | MCM6 | Minichromosome maintenance complex component 6 | 4.06E-12 |
| ENSG00000105486 | LIG1 | DNA ligase 1 | 4.21E-12 |
| ENSG00000119403 | PHF19 | PHD finger protein 19 | 4.27E-12 |
| ENSG00000125885 | MCM8 | Minichromosome maintenance 8 homologous recombination repair factor | 5.13E-12 |
| ENSG00000129810 | SGO1 | Shugoshin 1 | 6.31E-12 |
| ENSG00000100714 | MTHFD1 | Methylenetetrahydrofolate dehydrogenase, cyclohydrolase and formyltetrahydrofolate synthetase 1 | 6.58E-12 |
| ENSG00000145861 | C1QTNF2 | C1q and TNF related 2 | 9.64E-12 |
| ENSG00000164032 | H2AZ1 | H2A.Z variant histone 1 | 1.16E-11 |
| ENSG00000101224 | CDC25B | Cell division cycle 25B | 1.24E-11 |
| ENSG00000249115 | HAUS5 | HAUS augmin like complex subunit 5 | 1.26E-11 |
| ENSG00000128973 | CLN6 | CLN6 transmembrane ER protein | 1.31E-11 |
| ENSG00000162607 | USP1 | Ubiquitin specific peptidase 1 | 1.61E-11 |
| ENSG00000143799 | PARP1 | Poly(ADP-ribose) polymerase 1 | 1.72E-11 |
| ENSG00000118193 | KIF14 | Kinesin family member 14 | 2.19E-11 |
| ENSG00000111445 | RFC5 | Replication factor C subunit 5 | 3.42E-11 |
| ENSG00000121211 | MND1 | Meiotic nuclear divisions 1 | 3.43E-11 |
| ENSG00000104738 | MCM4 | Minichromosome maintenance complex component 4 | 3.71E-11 |
| ENSG00000097046 | CDC7 | Cell division cycle 7 | 3.92E-11 |
| ENSG00000168411 | RFWD3 | Ring finger and WD repeat domain 3 | 4.19E-11 |
| ENSG00000168393 | DTYMK | Deoxythymidylate kinase | 5.37E-11 |
| ENSG00000142731 | PLK4 | Polo like kinase 4 | 6.21E-11 |
| ENSG00000164045 | CDC25A | Cell division cycle 25A | 6.26E-11 |
| ENSG00000124795 | DEK | DEK proto-oncogene | 7.32E-11 |
| ENSG00000139734 | DIAPH3 | Diaphanous related formin 3 | 1.10E-10 |
| ENSG00000154920 | EME1 | Essential meiotic structure-specific endonuclease 1 | 1.25E-10 |
| ENSG00000075624 | ACTB | Actin beta | 1.56E-10 |
| ENSG00000153044 | CENPH | Centromere protein H | 1.69E-10 |
| ENSG00000126215 | XRCC3 | X-ray repair cross complementing 3 | 1.80E-10 |

| | | | |
|---|---|---|---|
| ENSG00000080839 | RBL1 | RB transcriptional corepressor like 1 | 1.86E-10 |
| ENSG00000138376 | BARD1 | BRCA1 associated RING domain 1 | 1.87E-10 |
| ENSG00000214357 | NEURL1B | Neuralized E3 ubiquitin protein ligase 1B | 2.13E-10 |
| ENSG00000004777 | ARHGAP33 | Rho gtpase activating protein 33 | 2.16E-10 |
| ENSG00000051341 | POLQ | DNA polymerase theta | 2.34E-10 |
| ENSG00000130695 | CEP85 | Centrosomal protein 85 | 2.71E-10 |
| ENSG00000123975 | CKS2 | CDC28 protein kinase regulatory subunit 2 | 2.81E-10 |
| ENSG00000062822 | POLD1 | DNA polymerase delta 1, catalytic subunit | 2.93E-10 |
| ENSG00000213186 | TRIM59 | Tripartite motif containing 59 | 3.34E-10 |
| ENSG00000119969 | HELLS | Helicase, lymphoid specific | 5.58E-10 |
| ENSG00000163950 | SLBP | Stem-loop binding protein | 5.84E-10 |
| ENSG00000132780 | NASP | Nuclear autoantigenic sperm protein | 6.00E-10 |
| ENSG00000183763 | TRAIP | TRAF interacting protein | 6.57E-10 |
| ENSG00000100629 | CEP128 | Centrosomal protein 128 | 6.79E-10 |
| ENSG00000118655 | DCLRE1B | DNA cross-link repair 1B | 6.80E-10 |
| ENSG00000164109 | MAD2L1 | Mitotic arrest deficient 2 like 1 | 7.09E-10 |
| ENSG00000040275 | SPDL1 | Spindle apparatus coiled-coil protein 1 | 7.34E-10 |
| ENSG00000185361 | TNFAIP8L1 | TNF alpha induced protein 8 like 1 | 8.82E-10 |
| ENSG00000159055 | MIS18A | MIS18 kinetochore protein A | 1.21E-09 |
| ENSG00000180198 | RCC1 | Regulator of chromosome condensation 1 | 1.32E-09 |
| ENSG00000126453 | BCL2L12 | BCL2 like 12 | 1.76E-09 |
| ENSG00000203760 | CENPW | Centromere protein W | 1.77E-09 |
| ENSG00000173207 | CKS1B | CDC28 protein kinase regulatory subunit 1B | 1.88E-09 |
| ENSG00000140451 | PIF1 | PIF1 5'-to-3' DNA helicase | 2.10E-09 |
| ENSG00000166451 | CENPN | Centromere protein N | 2.63E-09 |
| ENSG00000161692 | DBF4B | DBF4 zinc finger B | 3.59E-09 |
| ENSG00000136824 | SMC2 | Structural maintenance of chromosomes 2 | 4.40E-09 |
| ENSG00000139354 | GAS2L3 | Growth arrest specific 2 like 3 | 4.76E-09 |
| ENSG00000161547 | SRSF2 | Serine and arginine rich splicing factor 2 | 6.59E-09 |
| ENSG00000120334 | CENPL | Centromere protein L | 7.90E-09 |
| ENSG00000173894 | CBX2 | Chromobox 2 | 8.93E-09 |
| ENSG00000182963 | GJC1 | Gap junction protein gamma 1 | 9.52E-09 |
| ENSG00000123136 | DDX39A | Dexd-box helicase 39A | 9.61E-09 |
| ENSG00000134291 | TMEM106C | Transmembrane protein 106C | 9.93E-09 |
| ENSG00000143401 | ANP32E | Acidic nuclear phosphoprotein 32 family member E | 1.07E-08 |
| ENSG00000049541 | RFC2 | Replication factor C subunit 2 | 1.45E-08 |
| ENSG00000058804 | NDC1 | NDC1 transmembrane nucleoporin | 1.83E-08 |
| ENSG00000111331 | OAS3 | 2'-5'-oligoadenylate synthetase 3 | 2.07E-08 |
| ENSG00000096060 | FKBP5 | FKBP prolyl isomerase 5 | 2.38E-08 |
| ENSG00000162062 | TEDC2 | Tubulin epsilon and delta complex 2 | 2.56E-08 |
| ENSG00000095002 | MSH2 | Muts homolog 2 | 2.65E-08 |
| ENSG00000158164 | TMSB15A | Thymosin beta 15a | 3.11E-08 |
| ENSG00000188229 | TUBB4B | Tubulin beta 4B class ivb | 3.39E-08 |
| ENSG00000109881 | CCDC34 | Coiled-coil domain containing 34 | 3.43E-08 |
| ENSG00000176887 | SOX11 | SRY-box transcription factor 11 | 3.64E-08 |
| ENSG00000014138 | POLA2 | DNA polymerase alpha 2, accessory subunit | 5.71E-08 |
| ENSG00000102384 | CENPI | Centromere protein I | 6.00E-08 |
| ENSG00000197299 | BLM | BLM recq like helicase | 6.72E-08 |
| ENSG00000100350 | FOXRED2 | FAD dependent oxidoreductase domain containing 2 | 7.02E-08 |
| ENSG00000012963 | UBR7 | Ubiquitin protein ligase E3 component n-recognin 7 (putative) | 9.52E-08 |

| | | | |
|---|---|---|---|
| ENSG00000166845 | C18orf54 | Chromosome 18 open reading frame 54 | 1.01E-07 |
| ENSG00000128245 | YWHAH | Tyrosine 3-monooxygenase/tryptophan 5-monooxygenase activation protein eta | 1.26E-07 |
| ENSG00000136699 | SMPD4 | Sphingomyelin phosphodiesterase 4 | 1.40E-07 |
| ENSG00000000460 | C1orf112 | Chromosome 1 open reading frame 112 | 1.42E-07 |
| ENSG00000109084 | TMEM97 | Transmembrane protein 97 | 1.69E-07 |
| ENSG00000127423 | AUNIP | Aurora kinase A and ninein interacting protein | 1.95E-07 |
| ENSG00000213390 | ARHGAP19 | Rho gtpase activating protein 19 | 2.55E-07 |
| ENSG00000163535 | SGO2 | Shugoshin 2 | 2.82E-07 |
| ENSG00000083720 | OXCT1 | 3-oxoacid coa-transferase 1 | 3.01E-07 |
| ENSG00000168077 | SCARA3 | Scavenger receptor class A member 3 | 3.60E-07 |
| ENSG00000140525 | FANCI | FA complementation group I | 3.66E-07 |
| ENSG00000198176 | TFDP1 | Transcription factor Dp-1 | 4.02E-07 |
| ENSG00000167747 | C19orf48 | Chromosome 19 open reading frame 48 | 4.17E-07 |
| ENSG00000128944 | KNSTRN | Kinetochore localized astrin (SPAG5) binding protein | 4.53E-07 |
| ENSG00000125319 | HROB | Homologous recombination factor with OB-fold | 5.11E-07 |
| ENSG00000108106 | UBE2S | Ubiquitin conjugating enzyme E2 S | 5.58E-07 |
| ENSG00000188807 | TMEM201 | Transmembrane protein 201 | 5.83E-07 |
| ENSG00000164985 | PSIP1 | PC4 and SFRS1 interacting protein 1 | 6.21E-07 |
| ENSG00000259781 | HMGB1P6 | High mobility group box 1 pseudogene 6 | 6.29E-07 |
| ENSG00000010292 | NCAPD2 | Non-SMC condensin I complex subunit D2 | 6.46E-07 |
| ENSG00000285920 | AC087721.2 | Novel protein | 6.72E-07 |
| ENSG00000114346 | ECT2 | Epithelial cell transforming 2 | 7.29E-07 |
| ENSG00000165490 | DDIAS | DNA damage induced apoptosis suppressor | 8.02E-07 |
| ENSG00000149929 | HIRIP3 | HIRA interacting protein 3 | 8.24E-07 |
| ENSG00000221829 | FANCG | FA complementation group G | 8.41E-07 |
| ENSG00000174442 | ZWILCH | Zwilch kinetochore protein | 8.99E-07 |
| ENSG00000196230 | TUBB | Tubulin beta class I | 1.22E-06 |
| ENSG00000146410 | MTFR2 | Mitochondrial fission regulator 2 | 1.26E-06 |
| ENSG00000130402 | ACTN4 | Actinin alpha 4 | 1.30E-06 |
| ENSG00000198830 | HMGN2 | High mobility group nucleosomal binding domain 2 | 1.44E-06 |
| ENSG00000025770 | NCAPH2 | Non-SMC condensin II complex subunit H2 | 1.70E-06 |
| ENSG00000136982 | DSCC1 | DNA replication and sister chromatid cohesion 1 | 1.94E-06 |
| ENSG00000184216 | IRAK1 | Interleukin 1 receptor associated kinase 1 | 2.16E-06 |
| ENSG00000106144 | CASP2 | Caspase 2 | 2.40E-06 |
| ENSG00000011304 | PTBP1 | Polypyrimidine tract binding protein 1 | 3.14E-06 |
| ENSG00000153048 | CARHSP1 | Calcium regulated heat stable protein 1 | 3.23E-06 |
| ENSG00000182010 | RTKN2 | Rhotekin 2 | 4.48E-06 |
| ENSG00000077514 | POLD3 | DNA polymerase delta 3, accessory subunit | 4.73E-06 |
| ENSG00000128951 | DUT | Deoxyuridine triphosphatase | 4.73E-06 |
| ENSG00000189403 | HMGB1 | High mobility group box 1 | 5.17E-06 |
| ENSG00000187514 | PTMA | Prothymosin alpha | 5.42E-06 |
| ENSG00000129534 | MIS18BP1 | MIS18 binding protein 1 | 5.47E-06 |
| ENSG00000213853 | EMP2 | Epithelial membrane protein 2 | 5.72E-06 |
| ENSG00000087586 | AURKA | Aurora kinase A | 5.96E-06 |
| ENSG00000012048 | BRCA1 | BRCA1 DNA repair associated | 5.99E-06 |
| ENSG00000076248 | UNG | Uracil DNA glycosylase | 6.63E-06 |
| ENSG00000183048 | SLC25A10 | Solute carrier family 25 member 10 | 7.67E-06 |
| ENSG00000158246 | TENT5B | Terminal nucleotidyltransferase 5B | 9.14E-06 |
| ENSG00000096433 | ITPR3 | Inositol 1,4,5-trisphosphate receptor type 3 | 9.55E-06 |
| ENSG00000143554 | SLC27A3 | Solute carrier family 27 member 3 | 9.75E-06 |

| | | | |
|---|---|---|---|
| ENSG00000061337 | LZTS1 | Leucine zipper tumor suppressor 1 | 1.01E-05 |
| ENSG00000092820 | EZR | Ezrin | 1.02E-05 |
| ENSG00000166881 | NEMP1 | Nuclear envelope integral membrane protein 1 | 1.09E-05 |
| ENSG00000132436 | FIGNL1 | Fidgetin like 1 | 1.17E-05 |
| ENSG00000146263 | MMS22L | MMS22 like, DNA repair protein | 1.37E-05 |
| ENSG00000121957 | GPSM2 | G protein signaling modulator 2 | 1.37E-05 |
| ENSG00000144395 | CCDC150 | Coiled-coil domain containing 150 | 1.54E-05 |
| ENSG00000117748 | RPA2 | Replication protein A2 | 1.56E-05 |
| ENSG00000205208 | C4orf46 | Chromosome 4 open reading frame 46 | 1.76E-05 |
| ENSG00000184635 | ZNF93 | Zinc finger protein 93 | 1.90E-05 |
| ENSG00000166483 | WEE1 | WEE1 G2 checkpoint kinase | 1.91E-05 |
| ENSG00000099901 | RANBP1 | RAN binding protein 1 | 2.03E-05 |
| ENSG00000184162 | NR2C2AP | Nuclear receptor 2C2 associated protein | 2.32E-05 |
| ENSG00000169258 | GPRIN1 | G protein regulated inducer of neurite outgrowth 1 | 2.53E-05 |
| ENSG00000123416 | TUBA1B | Tubulin alpha 1b | 3.18E-05 |
| ENSG00000100749 | VRK1 | VRK serine/threonine kinase 1 | 3.46E-05 |
| ENSG00000165891 | E2F7 | E2F transcription factor 7 | 3.99E-05 |
| ENSG00000099783 | HNRNPM | Heterogeneous nuclear ribonucleoprotein M | 4.28E-05 |
| ENSG00000141560 | FN3KRP | Fructosamine 3 kinase related protein | 4.29E-05 |
| ENSG00000181938 | GINS3 | GINS complex subunit 3 | 4.73E-05 |
| ENSG00000123737 | EXOSC9 | Exosome component 9 | 4.74E-05 |
| ENSG00000137563 | GGH | Gamma-glutamyl hydrolase | 4.79E-05 |
| ENSG00000167553 | TUBA1C | Tubulin alpha 1c | 5.13E-05 |
| ENSG00000164649 | CDCA7L | Cell division cycle associated 7 like | 5.15E-05 |
| ENSG00000123374 | CDK2 | Cyclin dependent kinase 2 | 5.77E-05 |
| ENSG00000164105 | SAP30 | Sin3A associated protein 30 | 5.91E-05 |
| ENSG00000133026 | MYH10 | Myosin heavy chain 10 | 6.11E-05 |
| ENSG00000213024 | NUP62 | Nucleoporin 62 | 6.22E-05 |
| ENSG00000124207 | CSE1L | Chromosome segregation 1 like | 6.71E-05 |
| ENSG00000079462 | PAFAH1B3 | Platelet activating factor acetylhydrolase 1b catalytic subunit 3 | 7.23E-05 |
| ENSG00000105968 | H2AZ2 | H2A.Z variant histone 2 | 7.56E-05 |
| ENSG00000035928 | RFC1 | Replication factor C subunit 1 | 7.74E-05 |
| ENSG00000121774 | KHDRBS1 | KH RNA binding domain containing, signal transduction associated 1 | 8.00E-05 |
| ENSG00000121621 | KIF18A | Kinesin family member 18A | 9.17E-05 |
| ENSG00000175592 | FOSL1 | FOS like 1, AP-1 transcription factor subunit | 0.000108 |
| ENSG00000148019 | CEP78 | Centrosomal protein 78 | 0.000111 |
| ENSG00000186767 | SPIN4 | Spindlin family member 4 | 0.000127 |
| ENSG00000053438 | NNAT | Neuronatin | 0.000133 |
| ENSG00000161618 | ALDH16A1 | Aldehyde dehydrogenase 16 family member A1 | 0.000133 |
| ENSG00000111581 | NUP107 | Nucleoporin 107 | 0.000136 |
| ENSG00000115875 | SRSF7 | Serine and arginine rich splicing factor 7 | 0.000136 |
| ENSG00000197275 | RAD54B | RAD54 homolog B | 0.000155 |
| ENSG00000185480 | PARPBP | PARP1 binding protein | 0.000171 |
| ENSG00000086475 | SEPHS1 | Selenophosphate synthetase 1 | 0.000175 |
| ENSG00000135045 | C9orf40 | Chromosome 9 open reading frame 40 | 0.000185 |
| ENSG00000242114 | MTFP1 | Mitochondrial fission process 1 | 0.000197 |
| ENSG00000108055 | SMC3 | Structural maintenance of chromosomes 3 | 0.000198 |
| ENSG00000185262 | UBALD2 | UBA like domain containing 2 | 0.00022 |
| ENSG00000163781 | TOPBP1 | DNA topoisomerase II binding protein 1 | 0.000231 |

| | | | |
|---|---|---|---|
| ENSG00000113569 | NUP155 | Nucleoporin 155 | 0.000241 |
| ENSG00000183963 | SMTN | Smoothelin | 0.000247 |
| ENSG00000116237 | ICMT | Isoprenylcysteine carboxyl methyltransferase | 0.000278 |
| ENSG00000275591 | XKR5 | XK related 5 | 0.00029 |
| ENSG00000089280 | FUS | FUS RNA binding protein | 0.00029 |
| ENSG00000112759 | SLC29A1 | Solute carrier family 29 member 1 (Augustine blood group) | 0.000291 |
| ENSG00000197771 | MCMBP | Minichromosome maintenance complex binding protein | 0.000301 |
| ENSG00000006634 | DBF4 | DBF4 zinc finger | 0.000306 |
| ENSG00000159147 | DONSON | Downstream neighbor of SON | 0.000309 |
| ENSG00000075131 | TIPIN | TIMELESS interacting protein | 0.000311 |
| ENSG00000173456 | RNF26 | Ring finger protein 26 | 0.000325 |
| ENSG00000005189 | REXO5 | RNA exonuclease 5 | 0.000353 |
| ENSG00000137814 | HAUS2 | HAUS augmin like complex subunit 2 | 0.000358 |
| ENSG00000156136 | DCK | Deoxycytidine kinase | 0.000368 |
| ENSG00000151287 | TEX30 | Testis expressed 30 | 0.000375 |
| ENSG00000184992 | BRI3BP | BRI3 binding protein | 0.000399 |
| ENSG00000206053 | JPT2 | Jupiter microtubule associated homolog 2 | 0.000403 |
| ENSG00000108179 | PPIF | Peptidylprolyl isomerase F | 0.000415 |
| ENSG00000156876 | SASS6 | SAS-6 centriolar assembly protein | 0.000451 |
| ENSG00000116133 | DHCR24 | 24-dehydrocholesterol reductase | 0.000461 |
| ENSG00000184897 | H1-10 | H1.10 linker histone | 0.000499 |
| ENSG00000138658 | ZGRF1 | Zinc finger GRF-type containing 1 | 0.000545 |
| ENSG00000149136 | SSRP1 | Structure specific recognition protein 1 | 0.000582 |
| ENSG00000277443 | MARCKS | Myristoylated alanine rich protein kinase C substrate | 0.000584 |
| ENSG00000092201 | SUPT16H | SPT16 homolog, facilitates chromatin remodeling subunit | 0.000593 |
| ENSG00000031691 | CENPQ | Centromere protein Q | 0.000598 |
| ENSG00000136861 | CDK5RAP2 | CDK5 regulatory subunit associated protein 2 | 0.000733 |
| ENSG00000115687 | PASK | PAS domain containing serine/threonine kinase | 0.000756 |
| ENSG00000168476 | REEP4 | Receptor accessory protein 4 | 0.000757 |
| ENSG00000141556 | TBCD | Tubulin folding cofactor D | 0.000779 |
| ENSG00000131462 | TUBG1 | Tubulin gamma 1 | 0.000839 |
| ENSG00000176208 | ATAD5 | Atpase family AAA domain containing 5 | 0.000881 |
| ENSG00000125450 | NUP85 | Nucleoporin 85 | 0.000948 |
| ENSG00000178966 | RMI1 | Recq mediated genome instability 1 | 0.000983 |
| ENSG00000142856 | ITGB3BP | Integrin subunit beta 3 binding protein | 0.001004 |
| ENSG00000132341 | RAN | RAN, member RAS oncogene family | 0.001072 |
| ENSG00000100242 | SUN2 | Sad1 and UNC84 domain containing 2 | 0.001084 |
| ENSG00000068028 | RASSF1 | Ras association domain family member 1 | 0.001096 |
| ENSG00000125347 | IRF1 | Interferon regulatory factor 1 | 0.001204 |
| ENSG00000134802 | SLC43A3 | Solute carrier family 43 member 3 | 0.00125 |
| ENSG00000141499 | WRAP53 | WD repeat containing antisense to TP53 | 0.001402 |
| ENSG00000110108 | TMEM109 | Transmembrane protein 109 | 0.001608 |
| ENSG00000253304 | TMEM200B | Transmembrane protein 200B | 0.00168 |
| ENSG00000138668 | HNRNPD | Heterogeneous nuclear ribonucleoprotein D | 0.001742 |
| ENSG00000132383 | RPA1 | Replication protein A1 | 0.001825 |
| ENSG00000054967 | RELT | RELT TNF receptor | 0.001825 |
| ENSG00000104081 | BMF | Bcl2 modifying factor | 0.001825 |
| ENSG00000129484 | PARP2 | Poly(ADP-ribose) polymerase 2 | 0.001969 |
| ENSG00000160392 | C19orf47 | Chromosome 19 open reading frame 47 | 0.001986 |
| ENSG00000169813 | HNRNPF | Heterogeneous nuclear ribonucleoprotein F | 0.001986 |

| | | | |
|---|---|---|---|
| ENSG00000005022 | SLC25A5 | Solute carrier family 25 member 5 | 0.002021 |
| ENSG00000136122 | BORA | BORA aurora kinase A activator | 0.002034 |
| ENSG00000151849 | CENPJ | Centromere protein J | 0.002065 |
| ENSG00000173848 | NET1 | Neuroepithelial cell transforming 1 | 0.002078 |
| ENSG00000101773 | RBBP8 | RB binding protein 8, endonuclease | 0.00212 |
| ENSG00000147536 | GINS4 | GINS complex subunit 4 | 0.002139 |
| ENSG00000160352 | ZNF714 | Zinc finger protein 714 | 0.00216 |
| ENSG00000070814 | TCOF1 | Treacle ribosome biogenesis factor 1 | 0.002176 |
| ENSG00000179409 | GEMIN4 | Gem nuclear organelle associated protein 4 | 0.002248 |
| ENSG00000136527 | TRA2B | Transformer 2 beta homolog | 0.002288 |
| ENSG00000176894 | PXMP2 | Peroxisomal membrane protein 2 | 0.002301 |
| ENSG00000172009 | THOP1 | Thimet oligopeptidase 1 | 0.002351 |
| ENSG00000112039 | FANCE | FA complementation group E | 0.002427 |
| ENSG00000105173 | CCNE1 | Cyclin E1 | 0.00243 |
| ENSG00000109685 | NSD2 | Nuclear receptor binding SET domain protein 2 | 0.002641 |
| ENSG00000100479 | POLE2 | DNA polymerase epsilon 2, accessory subunit | 0.002647 |
| ENSG00000215784 | FAM72D | Family with sequence similarity 72 member D | 0.002685 |
| ENSG00000136518 | ACTL6A | Actin like 6A | 0.002756 |
| ENSG00000103995 | CEP152 | Centrosomal protein 152 | 0.00278 |
| ENSG00000125898 | FAM110A | Family with sequence similarity 110 member A | 0.002874 |
| ENSG00000151702 | FLI1 | Fli-1 proto-oncogene, ETS transcription factor | 0.003093 |
| ENSG00000126822 | PLEKHG3 | Pleckstrin homology and rhogef domain containing G3 | 0.003149 |
| ENSG00000154473 | BUB3 | BUB3 mitotic checkpoint protein | 0.003235 |
| ENSG00000140365 | COMMD4 | COMM domain containing 4 | 0.003242 |
| ENSG00000126216 | TUBGCP3 | Tubulin gamma complex associated protein 3 | 0.003404 |
| ENSG00000187790 | FANCM | FA complementation group M | 0.003445 |
| ENSG00000156509 | FBXO43 | F-box protein 43 | 0.00345 |
| ENSG00000156381 | ANKRD9 | Ankyrin repeat domain 9 | 0.003664 |
| ENSG00000104626 | ERI1 | Exoribonuclease 1 | 0.00371 |
| ENSG00000175216 | CKAP5 | Cytoskeleton associated protein 5 | 0.003755 |
| ENSG00000117877 | CD3EAP | CD3e molecule associated protein | 0.003817 |
| ENSG00000102007 | PLP2 | Proteolipid protein 2 | 0.003947 |
| ENSG00000114999 | TTL | Tubulin tyrosine ligase | 0.003956 |
| ENSG00000155561 | NUP205 | Nucleoporin 205 | 0.003987 |
| ENSG00000149548 | CCDC15 | Coiled-coil domain containing 15 | 0.004037 |
| ENSG00000182054 | IDH2 | Isocitrate dehydrogenase (NADP(+)) 2 | 0.004037 |
| ENSG00000051596 | THOC3 | THO complex 3 | 0.004277 |
| ENSG00000161996 | WDR90 | WD repeat domain 90 | 0.004285 |
| ENSG00000198885 | ITPRIPL1 | ITPRIP like 1 | 0.0044 |
| ENSG00000142149 | HUNK | Hormonally up-regulated Neu-associated kinase | 0.00441 |
| ENSG00000025772 | TOMM34 | Translocase of outer mitochondrial membrane 34 | 0.004497 |
| ENSG00000248710 | AC079594.2 | TRIM59 and ift80 readthrough | 0.004565 |
| ENSG00000119326 | CTNNAL1 | Catenin alpha like 1 | 0.004758 |
| ENSG00000102900 | NUP93 | Nucleoporin 93 | 0.004808 |
| ENSG00000159231 | CBR3 | Carbonyl reductase 3 | 0.004817 |
| ENSG00000149596 | JPH2 | Junctophilin 2 | 0.004928 |
| ENSG00000103121 | CMC2 | C-X9-C motif containing 2 | 0.005154 |
| ENSG00000102974 | CTCF | CCCTC-binding factor | 0.005154 |
| ENSG00000142733 | MAP3K6 | Mitogen-activated protein kinase kinase kinase 6 | 0.005354 |
| ENSG00000136450 | SRSF1 | Serine and arginine rich splicing factor 1 | 0.005354 |
| ENSG00000143815 | LBR | Lamin B receptor | 0.005399 |

| | | | |
|---|---|---|---|
| ENSG00000064666 | CNN2 | Calponin 2 | 0.005641 |
| ENSG00000138796 | HADH | Hydroxyacyl-coa dehydrogenase | 0.005689 |
| ENSG00000142230 | SAE1 | SUMO1 activating enzyme subunit 1 | 0.005742 |
| ENSG00000120437 | ACAT2 | Acetyl-coa acetyltransferase 2 | 0.005944 |
| ENSG00000284491 | THSD8 | Thrombospondin type 1 domain containing 8 | 0.006122 |
| ENSG00000136811 | ODF2 | Outer dense fiber of sperm tails 2 | 0.006324 |
| ENSG00000151466 | SCLT1 | Sodium channel and clathrin linker 1 | 0.006372 |
| ENSG00000106628 | POLD2 | DNA polymerase delta 2, accessory subunit | 0.006372 |
| ENSG00000239306 | RBM14 | RNA binding motif protein 14 | 0.006383 |
| ENSG00000005059 | MCUB | Mitochondrial calcium uniporter dominant negative beta subunit | 0.006408 |
| ENSG00000100401 | RANGAP1 | Ran gtpase activating protein 1 | 0.006465 |
| ENSG00000184009 | ACTG1 | Actin gamma 1 | 0.006504 |
| ENSG00000147155 | EBP | EBP cholestenol delta-isomerase | 0.006573 |
| ENSG00000171388 | APLN | Apelin | 0.006642 |
| ENSG00000213160 | KLHL23 | Kelch like family member 23 | 0.006672 |
| ENSG00000171649 | ZIK1 | Zinc finger protein interacting with K protein 1 | 0.006678 |
| ENSG00000122483 | CCDC18 | Coiled-coil domain containing 18 | 0.006996 |
| ENSG00000171793 | CTPS1 | CTP synthase 1 | 0.007255 |
| ENSG00000165501 | LRR1 | Leucine rich repeat protein 1 | 0.007324 |
| ENSG00000132017 | DCAF15 | DDB1 and CUL4 associated factor 15 | 0.00734 |
| ENSG00000108468 | CBX1 | Chromobox 1 | 0.007358 |
| ENSG00000136159 | NUDT15 | Nudix hydrolase 15 | 0.007576 |
| ENSG00000136715 | SAP130 | Sin3A associated protein 130 | 0.007802 |
| ENSG00000169683 | LRRC45 | Leucine rich repeat containing 45 | 0.007919 |
| ENSG00000174013 | FBXO45 | F-box protein 45 | 0.00816 |
| ENSG00000075618 | FSCN1 | Fascin actin-bundling protein 1 | 0.008264 |
| ENSG00000163002 | NUP35 | Nucleoporin 35 | 0.008337 |
| ENSG00000205476 | CCDC85C | Coiled-coil domain containing 85C | 0.008337 |
| ENSG00000131504 | DIAPH1 | Diaphanous related formin 1 | 0.008364 |
| ENSG00000100304 | TTLL12 | Tubulin tyrosine ligase like 12 | 0.008527 |
| ENSG00000166833 | NAV2 | Neuron navigator 2 | 0.008651 |
| ENSG00000011332 | DPF1 | Double PHD fingers 1 | 0.008712 |
| ENSG00000095319 | NUP188 | Nucleoporin 188 | 0.008725 |
| ENSG00000197451 | HNRNPAB | Heterogeneous nuclear ribonucleoprotein A/B | 0.00947 |
| ENSG00000116670 | MAD2L2 | Mitotic arrest deficient 2 like 2 | 0.009539 |
| ENSG00000180998 | GPR137C | G protein-coupled receptor 137C | 0.009912 |
| ENSG00000172687 | ZNF738 | Zinc finger protein 738 | 0.009912 |
| ENSG00000088247 | KHSRP | KH-type splicing regulatory protein | 0.009973 |
| ENSG00000101407 | TTI1 | TELO2 interacting protein 1 | 0.010043 |
| ENSG00000166801 | FAM111A | Family with sequence similarity 111 member A | 0.010294 |
| ENSG00000129355 | CDKN2D | Cyclin dependent kinase inhibitor 2D | 0.010324 |
| ENSG00000128408 | RIBC2 | RIB43A domain with coiled-coils 2 | 0.010623 |
| ENSG00000136938 | ANP32B | Acidic nuclear phosphoprotein 32 family member B | 0.010943 |
| ENSG00000163923 | RPL39L | Ribosomal protein L39 like | 0.011078 |
| ENSG00000111788 | AC009533.1 | DEAD/H (Asp-Glu-Ala-Asp/His) box polypeptide like pseudogene | 0.011081 |
| ENSG00000183814 | LIN9 | Lin-9 DREAM muvb core complex component | 0.011502 |
| ENSG00000105323 | HNRNPUL1 | Heterogeneous nuclear ribonucleoprotein U like 1 | 0.011502 |
| ENSG00000170379 | TCAF2 | TRPM8 channel associated factor 2 | 0.011704 |
| ENSG00000267041 | ZNF850 | Zinc finger protein 850 | 0.01177 |

| | | | |
|---|---|---|---|
| ENSG00000198331 | HYLS1 | HYLS1 centriolar and ciliogenesis associated | 0.011836 |
| ENSG00000164002 | EXO5 | Exonuclease 5 | 0.01195 |
| ENSG00000117593 | DARS2 | Aspartyl-trna synthetase 2, mitochondrial | 0.011999 |
| ENSG00000138587 | MNS1 | Meiosis specific nuclear structural 1 | 0.012054 |
| ENSG00000133392 | MYH11 | Myosin heavy chain 11 | 0.012167 |
| ENSG00000044459 | CNTLN | Centlein | 0.012396 |
| ENSG00000196363 | WDR5 | WD repeat domain 5 | 0.012396 |
| ENSG00000101004 | NINL | Ninein like | 0.012575 |
| ENSG00000138780 | GSTCD | Glutathione S-transferase C-terminal domain containing | 0.012814 |
| ENSG00000164742 | ADCY1 | Adenylate cyclase 1 | 0.012936 |
| ENSG00000061918 | GUCY1B1 | Guanylate cyclase 1 soluble subunit beta 1 | 0.012936 |
| ENSG00000164053 | ATRIP | ATR interacting protein | 0.013591 |
| ENSG00000135801 | TAF5L | TATA-box binding protein associated factor 5 like | 0.013811 |
| ENSG00000112081 | SRSF3 | Serine and arginine rich splicing factor 3 | 0.013855 |
| ENSG00000106459 | NRF1 | Nuclear respiratory factor 1 | 0.01391 |
| ENSG00000183688 | RFLNB | Refilin B | 0.014333 |
| ENSG00000170144 | HNRNPA3 | Heterogeneous nuclear ribonucleoprotein A3 | 0.014552 |
| ENSG00000101361 | NOP56 | NOP56 ribonucleoprotein | 0.015183 |
| ENSG00000135744 | AGT | Angiotensinogen | 0.015251 |
| ENSG00000143321 | HDGF | Heparin binding growth factor | 0.015251 |
| ENSG00000167088 | SNRPD1 | Small nuclear ribonucleoprotein D1 polypeptide | 0.015445 |
| ENSG00000285035 | AC091057.6 | Serine/threonine-protein kinase ULK4 (ULK4), pseudogene | 0.015448 |
| ENSG00000217555 | CKLF | Chemokine like factor | 0.015563 |
| ENSG00000177613 | CSTF2T | Cleavage stimulation factor subunit 2 tau variant | 0.015683 |
| ENSG00000183955 | KMT5A | Lysine methyltransferase 5A | 0.016031 |
| ENSG00000187676 | B3GLCT | Beta 3-glucosyltransferase | 0.016838 |
| ENSG00000166889 | PATL1 | PAT1 homolog 1, processing body mrna decay factor | 0.01728 |
| ENSG00000152455 | SUV39H2 | Suppressor of variegation 3-9 homolog 2 | 0.017679 |
| ENSG00000176225 | RTTN | Rotatin | 0.018559 |
| ENSG00000138346 | DNA2 | DNA replication helicase/nuclease 2 | 0.018897 |
| ENSG00000099849 | RASSF7 | Ras association domain family member 7 | 0.019598 |
| ENSG00000182575 | NXPH3 | Neurexophilin 3 | 0.020821 |
| ENSG00000198055 | GRK6 | G protein-coupled receptor kinase 6 | 0.021082 |
| ENSG00000183496 | MEX3B | Mex-3 RNA binding family member B | 0.021203 |
| ENSG00000171960 | PPIH | Peptidylprolyl isomerase H | 0.021378 |
| ENSG00000116350 | SRSF4 | Serine and arginine rich splicing factor 4 | 0.021864 |
| ENSG00000110958 | PTGES3 | Prostaglandin E synthase 3 | 0.021904 |
| ENSG00000121775 | TMEM39B | Transmembrane protein 39B | 0.021924 |
| ENSG00000169710 | FASN | Fatty acid synthase | 0.022366 |
| ENSG00000204899 | MZT1 | Mitotic spindle organizing protein 1 | 0.022521 |
| ENSG00000008838 | MED24 | Mediator complex subunit 24 | 0.022521 |
| ENSG00000180385 | EMC3-AS1 | EMC3 antisense RNA 1 | 0.022533 |
| ENSG00000116161 | CACYBP | Calcyclin binding protein | 0.022743 |
| ENSG00000130881 | LRP3 | LDL receptor related protein 3 | 0.022743 |
| ENSG00000171097 | KYAT1 | Kynurenine aminotransferase 1 | 0.023335 |
| ENSG00000275895 | U2AF1L5 | U2 small nuclear RNA auxiliary factor 1 like 5 | 0.023804 |
| ENSG00000183684 | ALYREF | Aly/REF export factor | 0.023953 |
| ENSG00000130520 | LSM4 | LSM4 homolog, U6 small nuclear RNA and mrna degradation associated | 0.024258 |
| ENSG00000198700 | IPO9 | Importin 9 | 0.025198 |

| | | | |
|---|---|---|---|
| ENSG00000006625 | GGCT | Gamma-glutamylcyclotransferase | 0.025742 |
| ENSG00000175643 | RMI2 | Recq mediated genome instability 2 | 0.025825 |
| ENSG00000167306 | MYO5B | Myosin VB | 0.025836 |
| ENSG00000254726 | MEX3A | Mex-3 RNA binding family member A | 0.025836 |
| ENSG00000205659 | LIN52 | Lin-52 DREAM muvb core complex component | 0.026108 |
| ENSG00000078900 | TP73 | Tumor protein p73 | 0.026449 |
| ENSG00000188312 | CENPP | Centromere protein P | 0.026939 |
| ENSG00000074696 | HACD3 | 3-hydroxyacyl-coa dehydratase 3 | 0.027313 |
| ENSG00000172336 | POP7 | POP7 homolog, ribonuclease P/MRP subunit | 0.02736 |
| ENSG00000128578 | STRIP2 | Striatin interacting protein 2 | 0.027816 |
| ENSG00000170989 | S1PR1 | Sphingosine-1-phosphate receptor 1 | 0.02853 |
| ENSG00000075461 | CACNG4 | Calcium voltage-gated channel auxiliary subunit gamma 4 | 0.028547 |
| ENSG00000197977 | ELOVL2 | ELOVL fatty acid elongase 2 | 0.028879 |
| ENSG00000103495 | MAZ | MYC associated zinc finger protein | 0.028879 |
| ENSG00000189159 | JPT1 | Jupiter microtubule associated homolog 1 | 0.028893 |
| ENSG00000256663 | AC112777.1 | Ubiquitin-like with PHD and ring finger domains 1 (UHRF1) pseudogene | 0.029783 |
| ENSG00000069974 | RAB27A | RAB27A, member RAS oncogene family | 0.029807 |
| ENSG00000112658 | SRF | Serum response factor | 0.030132 |
| ENSG00000094916 | CBX5 | Chromobox 5 | 0.03022 |
| ENSG00000198168 | SVIP | Small VCP interacting protein | 0.030297 |
| ENSG00000127337 | YEATS4 | YEATS domain containing 4 | 0.030373 |
| ENSG00000181588 | MEX3D | Mex-3 RNA binding family member D | 0.030405 |
| ENSG00000122779 | TRIM24 | Tripartite motif containing 24 | 0.030405 |
| ENSG00000137656 | BUD13 | BUD13 homolog | 0.030405 |
| ENSG00000119397 | CNTRL | Centriolin | 0.030478 |
| ENSG00000147955 | SIGMAR1 | Sigma non-opioid intracellular receptor 1 | 0.030847 |
| ENSG00000128708 | HAT1 | Histone acetyltransferase 1 | 0.031237 |
| ENSG00000162613 | FUBP1 | Far upstream element binding protein 1 | 0.031577 |
| ENSG00000177917 | ARL6IP6 | ADP ribosylation factor like gtpase 6 interacting protein 6 | 0.031885 |
| ENSG00000130826 | DKC1 | Dyskerin pseudouridine synthase 1 | 0.031885 |
| ENSG00000155755 | TMEM237 | Transmembrane protein 237 | 0.031929 |
| ENSG00000169689 | CENPX | Centromere protein X | 0.032383 |
| ENSG00000066044 | ELAVL1 | ELAV like RNA binding protein 1 | 0.032383 |
| ENSG00000139998 | RAB15 | RAB15, member RAS oncogene family | 0.032659 |
| ENSG00000183386 | FHL3 | Four and a half LIM domains 3 | 0.034072 |
| ENSG00000229676 | ZNF492 | Zinc finger protein 492 | 0.034179 |
| ENSG00000128050 | PAICS | Phosphoribosylaminoimidazole carboxylase and phosphoribosylaminoimidazolesuccinocarboxamide synthase | 0.034399 |
| ENSG00000130935 | NOL11 | Nucleolar protein 11 | 0.034588 |
| ENSG00000130208 | APOC1 | Apolipoprotein C1 | 0.035042 |
| ENSG00000130726 | TRIM28 | Tripartite motif containing 28 | 0.035584 |
| ENSG00000106268 | NUDT1 | Nudix hydrolase 1 | 0.035611 |
| ENSG00000164754 | RAD21 | RAD21 cohesin complex component | 0.035736 |
| ENSG00000124160 | NCOA5 | Nuclear receptor coactivator 5 | 0.036026 |
| ENSG00000117676 | RPS6KA1 | Ribosomal protein S6 kinase A1 | 0.037086 |
| ENSG00000051825 | MPHOSPH9 | M-phase phosphoprotein 9 | 0.037086 |
| ENSG00000135052 | GOLM1 | Golgi membrane protein 1 | 0.037261 |
| ENSG00000137124 | ALDH1B1 | Aldehyde dehydrogenase 1 family member B1 | 0.037274 |

| | | | |
|---|---|---|---|
| ENSG00000103966 | EHD4 | EH domain containing 4 | 0.037302 |
| ENSG00000090273 | NUDC | Nuclear distribution C, dynein complex regulator | 0.038214 |
| ENSG00000149782 | PLCB3 | Phospholipase C beta 3 | 0.038218 |
| ENSG00000188566 | NDOR1 | NADPH dependent diflavin oxidoreductase 1 | 0.039752 |
| ENSG00000178531 | CTXN1 | Cortexin 1 | 0.040737 |
| ENSG00000120699 | EXOSC8 | Exosome component 8 | 0.041059 |
| ENSG00000165527 | ARF6 | ADP ribosylation factor 6 | 0.041662 |
| ENSG00000256229 | ZNF486 | Zinc finger protein 486 | 0.042014 |
| ENSG00000104885 | DOT1L | DOT1 like histone lysine methyltransferase | 0.042079 |
| ENSG00000180979 | LRRC57 | Leucine rich repeat containing 57 | 0.042184 |
| ENSG00000130669 | PAK4 | P21 (RAC1) activated kinase 4 | 0.042878 |
| ENSG00000205531 | NAP1L4 | Nucleosome assembly protein 1 like 4 | 0.04297 |
| ENSG00000177733 | HNRNPA0 | Heterogeneous nuclear ribonucleoprotein A0 | 0.04367 |
| ENSG00000092036 | HAUS4 | HAUS augmin like complex subunit 4 | 0.043836 |
| ENSG00000078246 | TULP3 | TUB like protein 3 | 0.044114 |
| ENSG00000132661 | NXT1 | Nuclear transport factor 2 like export factor 1 | 0.045091 |
| ENSG00000070950 | RAD18 | RAD18 E3 ubiquitin protein ligase | 0.045478 |
| ENSG00000116062 | MSH6 | Muts homolog 6 | 0.045478 |
| ENSG00000127191 | TRAF2 | TNF receptor associated factor 2 | 0.045478 |
| ENSG00000162066 | AMDHD2 | Amidohydrolase domain containing 2 | 0.045478 |
| ENSG00000105202 | FBL | Fibrillarin | 0.045478 |
| ENSG00000167173 | C15orf39 | Chromosome 15 open reading frame 39 | 0.045478 |
| ENSG00000257921 | AC025165.3 | Novel protein | 0.045983 |
| ENSG00000050820 | BCAR1 | BCAR1 scaffold protein, Cas family member | 0.046295 |
| ENSG00000102034 | ELF4 | E74 like ETS transcription factor 4 | 0.046427 |
| ENSG00000172731 | LRRC20 | Leucine rich repeat containing 20 | 0.04662 |
| ENSG00000143493 | INTS7 | Integrator complex subunit 7 | 0.046652 |
| ENSG00000152284 | TCF7L1 | Transcription factor 7 like 1 | 0.046684 |
| ENSG00000267060 | PTGES3L | Prostaglandin E synthase 3 like | 0.046763 |
| ENSG00000002822 | MAD1L1 | Mitotic arrest deficient 1 like 1 | 0.047023 |
| ENSG00000167699 | GLOD4 | Glyoxalase domain containing 4 | 0.047357 |
| ENSG00000140682 | TGFB1I1 | Transforming growth factor beta 1 induced transcript 1 | 0.047357 |
| ENSG00000171791 | BCL2 | BCL2 apoptosis regulator | 0.047694 |
| ENSG00000172244 | C5orf34 | Chromosome 5 open reading frame 34 | 0.047741 |
| ENSG00000084710 | EFR3B | EFR3 homolog B | 0.047741 |
| ENSG00000178202 | POGLUT3 | Protein O-glucosyltransferase 3 | 0.047763 |
| ENSG00000137834 | SMAD6 | SMAD family member 6 | 0.048255 |
| ENSG00000156787 | TBC1D31 | TBC1 domain family member 31 | 0.04935 |

**Supplementary Table S3.** Statistically significant DEGs (Adj.*p*-value<0.05) derived from DGEA of RNA-Seq data from Human Lens Epithelial Cells after X-ray irradiation, using DESeq2. The experiment consists of 5 control samples and 5 irradiated with 5 Gy X-ray radiation for 20 hours [Bioproject: PRJNA494581].

| Ensembl ID | Gene Symbol | Gene Description | Adj. *p*-Value |
|---|---|---|---|
| | | **Up-Regulated Genes↑** | |
| ENSG00000124762 | CDKN1A | Cyclin dependent kinase inhibitor 1A | 3.70E-39 |
| ENSG00000109321 | AREG | Amphiregulin | 1.75E-27 |
| ENSG00000164938 | TP53INP1 | Tumor protein p53 inducible nuclear protein 1 | 5.09E-24 |
| ENSG00000130513 | GDF15 | Growth differentiation factor 15 | 7.44E-16 |
| ENSG00000076382 | SPAG5 | Sperm associated antigen 5 | 1.34E-15 |
| ENSG00000256664 | AC025423.2 | Ribosomal L24 domain containing 1 (RSL24D1) pseudogene | 1.35E-14 |
| ENSG00000175063 | UBE2C | Ubiquitin conjugating enzyme E2 C | 1.24E-13 |
| ENSG00000090889 | KIF4A | Kinesin family member 4A | 2.64E-13 |
| ENSG00000132563 | REEP2 | Receptor accessory protein 2 | 3.57E-13 |
| ENSG00000176244 | ACBD7 | Acyl-coa binding domain containing 7 | 1.92E-12 |
| ENSG00000119397 | CNTRL | Centriolin | 2.15E-12 |
| ENSG00000157456 | CCNB2 | Cyclin B2 | 2.34E-12 |
| ENSG00000143217 | NECTIN4 | Nectin cell adhesion molecule 4 | 3.08E-12 |
| ENSG00000177459 | ERICH5 | Glutamate rich 5 | 5.58E-12 |
| ENSG00000135679 | MDM2 | MDM2 proto-oncogene | 8.64E-12 |
| ENSG00000135423 | GLS2 | Glutaminase 2 | 1.49E-11 |
| ENSG00000120889 | TNFRSF10B | TNF receptor superfamily member 10b | 3.97E-11 |
| ENSG00000087586 | AURKA | Aurora kinase A | 1.58E-10 |
| ENSG00000162490 | DRAXIN | Dorsal inhibitory axon guidance protein | 1.69E-10 |
| ENSG00000100526 | CDKN3 | Cyclin dependent kinase inhibitor 3 | 1.03E-09 |
| ENSG00000159388 | BTG2 | BTG anti-proliferation factor 2 | 1.47E-09 |
| ENSG00000123933 | MXD4 | MAX dimerization protein 4 | 6.31E-09 |
| ENSG00000167107 | ACSF2 | Acyl-coa synthetase family member 2 | 7.26E-09 |
| ENSG00000117724 | CENPF | Centromere protein F | 8.50E-09 |
| ENSG00000112984 | KIF20A | Kinesin family member 20A | 1.18E-08 |
| ENSG00000185361 | TNFAIP8L1 | TNF alpha induced protein 8 like 1 | 3.11E-08 |
| ENSG00000115163 | CENPA | Centromere protein A | 4.32E-08 |
| ENSG00000066279 | ASPM | Abnormal spindle microtubule assembly | 5.00E-08 |
| ENSG00000166592 | RRAD | RRAD, Ras related glycolysis inhibitor and calcium channel regulator | 5.37E-08 |
| ENSG00000198919 | DZIP3 | DAZ interacting zinc finger protein 3 | 5.98E-08 |
| ENSG00000170540 | ARL6IP1 | ADP ribosylation factor like gtpase 6 interacting protein 1 | 1.03E-07 |
| ENSG00000104870 | FCGRT | Fc fragment of igg receptor and transporter | 1.08E-07 |
| ENSG00000068745 | IP6K2 | Inositol hexakisphosphate kinase 2 | 1.14E-07 |
| ENSG00000161513 | FDXR | Ferredoxin reductase | 1.72E-07 |
| ENSG00000139354 | GAS2L3 | Growth arrest specific 2 like 3 | 2.20E-07 |
| ENSG00000153208 | MERTK | MER proto-oncogene, tyrosine kinase | 4.61E-07 |
| ENSG00000142945 | KIF2C | Kinesin family member 2C | 4.85E-07 |
| ENSG00000214357 | NEURL1B | Neuralized E3 ubiquitin protein ligase 1B | 8.49E-07 |
| ENSG00000177076 | ACER2 | Alkaline ceramidase 2 | 1.07E-06 |
| ENSG00000010030 | ETV7 | ETS variant transcription factor 7 | 2.33E-06 |
| ENSG00000239713 | APOBEC3G | Apolipoprotein B mrna editing enzyme catalytic subunit 3G | 2.89E-06 |
| ENSG00000118193 | KIF14 | Kinesin family member 14 | 3.00E-06 |

| | | | |
|---|---|---|---|
| ENSG00000111665 | CDCA3 | Cell division cycle associated 3 | 3.45E-06 |
| ENSG00000127483 | HP1BP3 | Heterochromatin protein 1 binding protein 3 | 3.72E-06 |
| ENSG00000021826 | CPS1 | Carbamoyl-phosphate synthase 1 | 3.86E-06 |
| ENSG00000168918 | INPP5D | Inositol polyphosphate-5-phosphatase D | 4.12E-06 |
| ENSG00000055163 | CYFIP2 | Cytoplasmic FMR1 interacting protein 2 | 4.56E-06 |
| ENSG00000138271 | GPR87 | G protein-coupled receptor 87 | 4.69E-06 |
| ENSG00000026103 | FAS | Fas cell surface death receptor | 6.14E-06 |
| ENSG00000276180 | H4C9 | H4 clustered histone 9 | 7.10E-06 |
| ENSG00000164463 | CREBRF | CREB3 regulatory factor | 7.20E-06 |
| ENSG00000158402 | CDC25C | Cell division cycle 25C | 8.12E-06 |
| ENSG00000137975 | CLCA2 | Chloride channel accessory 2 | 9.39E-06 |
| ENSG00000154040 | CABYR | Calcium binding tyrosine phosphorylation regulated | 1.17E-05 |
| ENSG00000169760 | NLGN1 | Neuroligin 1 | 1.33E-05 |
| ENSG00000153029 | MR1 | Major histocompatibility complex, class I-related | 1.33E-05 |
| ENSG00000072571 | HMMR | Hyaluronan mediated motility receptor | 1.67E-05 |
| ENSG00000117461 | PIK3R3 | Phosphoinositide-3-kinase regulatory subunit 3 | 1.68E-05 |
| ENSG00000131747 | TOP2A | DNA topoisomerase II alpha | 1.78E-05 |
| ENSG00000075218 | GTSE1 | G2 and S-phase expressed 1 | 1.93E-05 |
| ENSG00000133101 | CCNA1 | Cyclin A1 | 2.12E-05 |
| ENSG00000173156 | RHOD | Ras homolog family member D | 2.83E-05 |
| ENSG00000105650 | PDE4C | Phosphodiesterase 4C | 3.37E-05 |
| ENSG00000132824 | SERINC3 | Serine incorporator 3 | 4.40E-05 |
| ENSG00000196814 | MVB12B | Multivesicular body subunit 12B | 5.87E-05 |
| ENSG00000047346 | FAM214A | Family with sequence similarity 214 member A | 6.26E-05 |
| ENSG00000139291 | TMEM19 | Transmembrane protein 19 | 8.39E-05 |
| ENSG00000189195 | BTBD8 | BTB domain containing 8 | 0.000107 |
| ENSG00000129195 | PIMREG | PICALM interacting mitotic regulator | 0.000127 |
| ENSG00000073331 | ALPK1 | Alpha kinase 1 | 0.000133 |
| ENSG00000063854 | HAGH | Hydroxyacylglutathione hydrolase | 0.000149 |
| ENSG00000164611 | PTTG1 | PTTG1 regulator of sister chromatid separation, securin | 0.000192 |
| ENSG00000117650 | NEK2 | NIMA related kinase 2 | 0.000198 |
| ENSG00000161896 | IP6K3 | Inositol hexakisphosphate kinase 3 | 0.000204 |
| ENSG00000137414 | FAM8A1 | Family with sequence similarity 8 member A1 | 0.000216 |
| ENSG00000166313 | APBB1 | Amyloid beta precursor protein binding family B member 1 | 0.000227 |
| ENSG00000233369 | GTF2IP4 | General transcription factor iii pseudogene 4 | 0.00023 |
| ENSG00000198909 | MAP3K3 | Mitogen-activated protein kinase kinase kinase 3 | 0.000241 |
| ENSG00000173230 | GOLGB1 | Golgin B1 | 0.000243 |
| ENSG00000148660 | CAMK2G | Calcium/calmodulin dependent protein kinase II gamma | 0.000244 |
| ENSG00000175938 | ORAI3 | ORAI calcium release-activated calcium modulator 3 | 0.000264 |
| ENSG00000105325 | FZR1 | Fizzy and cell division cycle 20 related 1 | 0.000299 |
| ENSG00000130517 | PGPEP1 | Pyroglutamyl-peptidase I | 0.000335 |
| ENSG00000128944 | KNSTRN | Kinetochore localized astrin (SPAG5) binding protein | 0.00034 |
| ENSG00000178401 | DNAJC22 | Dnaj heat shock protein family (Hsp40) member C22 | 0.000362 |
| ENSG00000144452 | ABCA12 | ATP binding cassette subfamily A member 12 | 0.000394 |
| ENSG00000179163 | FUCA1 | Alpha-L-fucosidase 1 | 0.000403 |
| ENSG00000024526 | DEPDC1 | DEP domain containing 1 | 0.000498 |
| ENSG00000175793 | SFN | Stratifin | 0.000587 |
| ENSG00000158055 | GRHL3 | Grainyhead like transcription factor 3 | 0.000589 |
| ENSG00000197892 | KIF13B | Kinesin family member 13B | 0.000602 |
| ENSG00000108830 | RND2 | Rho family gtpase 2 | 0.000646 |

| | | | |
|---|---|---|---|
| ENSG00000139508 | SLC46A3 | Solute carrier family 46 member 3 | 0.000674 |
| ENSG00000092140 | G2E3 | G2/M-phase specific E3 ubiquitin protein ligase | 0.000686 |
| ENSG00000136859 | ANGPTL2 | Angiopoietin like 2 | 0.000698 |
| ENSG00000157150 | TIMP4 | TIMP metallopeptidase inhibitor 4 | 0.000706 |
| ENSG00000151117 | TMEM86A | Transmembrane protein 86A | 0.000728 |
| ENSG00000126709 | IFI6 | Interferon alpha inducible protein 6 | 0.000759 |
| ENSG00000160539 | PLPP7 | Phospholipid phosphatase 7 (inactive) | 0.000832 |
| ENSG00000163346 | PBXIP1 | PBX homeobox interacting protein 1 | 0.000946 |
| ENSG00000119630 | PGF | Placental growth factor | 0.001018 |
| ENSG00000197496 | SLC2A10 | Solute carrier family 2 member 10 | 0.00119 |
| ENSG00000163683 | SMIM14 | Small integral membrane protein 14 | 0.001282 |
| ENSG00000136108 | CKAP2 | Cytoskeleton associated protein 2 | 0.001294 |
| ENSG00000105483 | CARD8 | Caspase recruitment domain family member 8 | 0.001435 |
| ENSG00000174684 | B4GAT1 | Beta-1,4-glucuronyltransferase 1 | 0.001489 |
| ENSG00000132793 | LPIN3 | Lipin 3 | 0.001489 |
| ENSG00000188921 | HACD4 | 3-hydroxyacyl-coa dehydratase 4 | 0.001678 |
| ENSG00000166233 | ARIH1 | Ariadne RBR E3 ubiquitin protein ligase 1 | 0.00175 |
| ENSG00000182481 | KPNA2 | Karyopherin subunit alpha 2 | 0.00175 |
| ENSG00000011243 | AKAP8L | A-kinase anchoring protein 8 like | 0.001787 |
| ENSG00000119139 | TJP2 | Tight junction protein 2 | 0.001898 |
| ENSG00000175216 | CKAP5 | Cytoskeleton associated protein 5 | 0.001912 |
| ENSG00000160469 | BRSK1 | BR serine/threonine kinase 1 | 0.001928 |
| ENSG00000001461 | NIPAL3 | NIPA like domain containing 3 | 0.001944 |
| ENSG00000184661 | CDCA2 | Cell division cycle associated 2 | 0.002024 |
| ENSG00000116299 | KIAA1324 | Kiaa1324 | 0.002318 |
| ENSG00000163596 | ICA1L | Islet cell autoantigen 1 like | 0.002417 |
| ENSG00000089041 | P2RX7 | Purinergic receptor P2X 7 | 0.002478 |
| ENSG00000131015 | ULBP2 | UL16 binding protein 2 | 0.002618 |
| ENSG00000185215 | TNFAIP2 | TNF alpha induced protein 2 | 0.002838 |
| ENSG00000096872 | IFT74 | Intraflagellar transport 74 | 0.00284 |
| ENSG00000166311 | SMPD1 | Sphingomyelin phosphodiesterase 1 | 0.002855 |
| ENSG00000105443 | CYTH2 | Cytohesin 2 | 0.002957 |
| ENSG00000161618 | ALDH16A1 | Aldehyde dehydrogenase 16 family member A1 | 0.003124 |
| ENSG00000138606 | SHF | Src homology 2 domain containing F | 0.003198 |
| ENSG00000136378 | ADAMTS7 | ADAM metallopeptidase with thrombospondin type 1 motif 7 | 0.003198 |
| ENSG00000108187 | PBLD | Phenazine biosynthesis like protein domain containing | 0.003218 |
| ENSG00000142459 | EVI5L | Ecotropic viral integration site 5 like | 0.003222 |
| ENSG00000188002 | AC026412.1 | Programmed cell death 6 (PDCD6) pseudogene | 0.003222 |
| ENSG00000213463 | SYNJ2BP | Synaptojanin 2 binding protein | 0.003299 |
| ENSG00000118898 | PPL | Periplakin | 0.0033 |
| ENSG00000156973 | PDE6D | Phosphodiesterase 6D | 0.003349 |
| ENSG00000241839 | PLEKHO2 | Pleckstrin homology domain containing O2 | 0.003407 |
| ENSG00000204219 | TCEA3 | Transcription elongation factor A3 | 0.003501 |
| ENSG00000120693 | SMAD9 | SMAD family member 9 | 0.003514 |
| ENSG00000143390 | RFX5 | Regulatory factor X5 | 0.003522 |
| ENSG00000176438 | SYNE3 | Spectrin repeat containing nuclear envelope family member 3 | 0.003522 |
| ENSG00000179277 | MEIS3P1 | Meis homeobox 3 pseudogene 1 | 0.00357 |
| ENSG00000244694 | PTCHD4 | Patched domain containing 4 | 0.003593 |
| ENSG00000035499 | DEPDC1B | DEP domain containing 1B | 0.003685 |

| | | | |
|---|---|---|---|
| ENSG00000168874 | ATOH8 | Atonal bhlh transcription factor 8 | 0.003702 |
| ENSG00000149809 | TM7SF2 | Transmembrane 7 superfamily member 2 | 0.003729 |
| ENSG00000169679 | BUB1 | BUB1 mitotic checkpoint serine/threonine kinase | 0.003775 |
| ENSG00000117399 | CDC20 | Cell division cycle 20 | 0.003917 |
| ENSG00000175893 | ZDHHC21 | Zinc finger DHHC-type containing 21 | 0.003964 |
| ENSG00000104177 | MYEF2 | Myelin expression factor 2 | 0.004088 |
| ENSG00000237094 | AL732372.2 | Pseudogene similar to part of septin 14 SEPT14 | 0.004106 |
| ENSG00000132623 | ANKEF1 | Ankyrin repeat and EF-hand domain containing 1 | 0.004148 |
| ENSG00000130703 | OSBPL2 | Oxysterol binding protein like 2 | 0.004328 |
| ENSG00000133460 | SLC2A11 | Solute carrier family 2 member 11 | 0.004328 |
| ENSG00000196187 | TMEM63A | Transmembrane protein 63A | 0.004328 |
| ENSG00000167861 | HID1 | HID1 domain containing | 0.004328 |
| ENSG00000142619 | PADI3 | Peptidyl arginine deiminase 3 | 0.004328 |
| ENSG00000231007 | CDC20P1 | Cell division cycle 20 pseudogene 1 | 0.004468 |
| ENSG00000185432 | METTL7A | Methyltransferase like 7A | 0.004498 |
| ENSG00000139112 | GABARAPL1 | GABA type A receptor associated protein like 1 | 0.004541 |
| ENSG00000165861 | ZFYVE1 | Zinc finger FYVE-type containing 1 | 0.004612 |
| ENSG00000102981 | PARD6A | Par-6 family cell polarity regulator alpha | 0.004721 |
| ENSG00000196550 | FAM72A | Family with sequence similarity 72 member A | 0.004743 |
| ENSG00000034713 | GABARAPL2 | GABA type A receptor associated protein like 2 | 0.004771 |
| ENSG00000076685 | NT5C2 | 5'-nucleotidase, cytosolic II | 0.004943 |
| ENSG00000197429 | IPP | Intracisternal A particle-promoted polypeptide | 0.00519 |
| ENSG00000184602 | SNN | Stannin | 0.005326 |
| ENSG00000114107 | CEP70 | Centrosomal protein 70 | 0.005499 |
| ENSG00000165219 | GAPVD1 | Gtpase activating protein and VPS9 domains 1 | 0.005565 |
| ENSG00000188554 | NBR1 | NBR1 autophagy cargo receptor | 0.005581 |
| ENSG00000119403 | PHF19 | PHD finger protein 19 | 0.005763 |
| ENSG00000064199 | SPA17 | Sperm autoantigenic protein 17 | 0.006228 |
| ENSG00000139597 | N4BP2L1 | NEDD4 binding protein 2 like 1 | 0.006267 |
| ENSG00000164088 | PPM1M | Protein phosphatase, Mg2+/Mn2+ dependent 1M | 0.006334 |
| ENSG00000132849 | PATJ | PATJ crumbs cell polarity complex component | 0.006337 |
| ENSG00000156671 | SAMD8 | Sterile alpha motif domain containing 8 | 0.006347 |
| ENSG00000138495 | COX17 | Cytochrome c oxidase copper chaperone COX17 | 0.00656 |
| ENSG00000169621 | APLF | Aprataxin and PNKP like factor | 0.00661 |
| ENSG00000135452 | TSPAN31 | Tetraspanin 31 | 0.00664 |
| ENSG00000135451 | TROAP | Trophinin associated protein | 0.00664 |
| ENSG00000085998 | POMGNT1 | Protein O-linked mannose N-acetylglucosaminyltransferase 1 (beta 1,2-) | 0.006658 |
| ENSG00000080986 | NDC80 | NDC80 kinetochore complex component | 0.006658 |
| ENSG00000126787 | DLGAP5 | DLG associated protein 5 | 0.006732 |
| ENSG00000185339 | TCN2 | Transcobalamin 2 | 0.006737 |
| ENSG00000112983 | BRD8 | Bromodomain containing 8 | 0.006845 |
| ENSG00000103485 | QPRT | Quinolinate phosphoribosyltransferase | 0.00685 |
| ENSG00000125746 | EML2 | EMAP like 2 | 0.00685 |
| ENSG00000169902 | TPST1 | Tyrosylprotein sulfotransferase 1 | 0.007033 |
| ENSG00000158467 | AHCYL2 | Adenosylhomocysteinase like 2 | 0.0073 |
| ENSG00000101447 | FAM83D | Family with sequence similarity 83 member D | 0.007334 |
| ENSG00000141504 | SAT2 | Spermidine/spermine N1-acetyltransferase family member 2 | 0.007526 |
| ENSG00000149212 | SESN3 | Sestrin 3 | 0.007815 |
| ENSG00000138798 | EGF | Epidermal growth factor | 0.007859 |

| | | | |
|---|---|---|---|
| ENSG00000127914 | AKAP9 | A-kinase anchoring protein 9 | 0.007878 |
| ENSG00000132570 | PCBD2 | Pterin-4 alpha-carbinolamine dehydratase 2 | 0.007986 |
| ENSG00000168734 | PKIG | Camp-dependent protein kinase inhibitor gamma | 0.008273 |
| ENSG00000158457 | TSPAN33 | Tetraspanin 33 | 0.008298 |
| ENSG00000064687 | ABCA7 | ATP binding cassette subfamily A member 7 | 0.008324 |
| ENSG00000111269 | CREBL2 | Camp responsive element binding protein like 2 | 0.008329 |
| ENSG00000146232 | NFKBIE | NFKB inhibitor epsilon | 0.008329 |
| ENSG00000161835 | GRASP | General receptor for phosphoinositides 1 associated scaffold protein | 0.008421 |
| ENSG00000163449 | TMEM169 | Transmembrane protein 169 | 0.008489 |
| ENSG00000158106 | RHPN1 | Rhophilin Rho gtpase binding protein 1 | 0.008495 |
| ENSG00000188766 | SPRED3 | Sprouty related EVH1 domain containing 3 | 0.008537 |
| ENSG00000174007 | CEP19 | Centrosomal protein 19 | 0.008557 |
| ENSG00000105851 | PIK3CG | Phosphatidylinositol-4,5-bisphosphate 3-kinase catalytic subunit gamma | 0.00866 |
| ENSG00000121310 | ECHDC2 | Enoyl-coa hydratase domain containing 2 | 0.009255 |
| ENSG00000137880 | GCHFR | GTP cyclohydrolase I feedback regulator | 0.009255 |
| ENSG00000154240 | CEP112 | Centrosomal protein 112 | 0.009681 |
| ENSG00000012822 | CALCOCO1 | Calcium binding and coiled-coil domain 1 | 0.009774 |
| ENSG00000173064 | HECTD4 | HECT domain E3 ubiquitin protein ligase 4 | 0.009934 |
| ENSG00000239382 | ALKBH6 | Alkb homolog 6 | 0.010383 |
| ENSG00000181513 | ACBD4 | Acyl-coa binding domain containing 4 | 0.010426 |
| ENSG00000092758 | COL9A3 | Collagen type IX alpha 3 chain | 0.010511 |
| ENSG00000155111 | CDK19 | Cyclin dependent kinase 19 | 0.010678 |
| ENSG00000110713 | NUP98 | Nucleoporin 98 | 0.010749 |
| ENSG00000089685 | BIRC5 | Baculoviral IAP repeat containing 5 | 0.010906 |
| ENSG00000055732 | MCOLN3 | Mucolipin 3 | 0.011107 |
| ENSG00000196502 | SULT1A1 | Sulfotransferase family 1A member 1 | 0.011272 |
| ENSG00000173214 | MFSD4B | Major facilitator superfamily domain containing 4B | 0.011392 |
| ENSG00000135119 | RNFT2 | Ring finger protein, transmembrane 2 | 0.011459 |
| ENSG00000169583 | CLIC3 | Chloride intracellular channel 3 | 0.011513 |
| ENSG00000144834 | TAGLN3 | Transgelin 3 | 0.011947 |
| ENSG00000087884 | AAMDC | Adipogenesis associated Mth938 domain containing | 0.011965 |
| ENSG00000056558 | TRAF1 | TNF receptor associated factor 1 | 0.012012 |
| ENSG00000178038 | ALS2CL | ALS2 C-terminal like | 0.012283 |
| ENSG00000108106 | UBE2S | Ubiquitin conjugating enzyme E2 S | 0.012575 |
| ENSG00000137804 | NUSAP1 | Nucleolar and spindle associated protein 1 | 0.012956 |
| ENSG00000181754 | AMIGO1 | Adhesion molecule with Ig like domain 1 | 0.013273 |
| ENSG00000134057 | CCNB1 | Cyclin B1 | 0.0133 |
| ENSG00000219481 | NBPF1 | NBPF member 1 | 0.013365 |
| ENSG00000140386 | SCAPER | S-phase cyclin A associated protein in the ER | 0.013486 |
| ENSG00000123609 | NMI | N-myc and STAT interactor | 0.013486 |
| ENSG00000120709 | FAM53C | Family with sequence similarity 53 member C | 0.013752 |
| ENSG00000100401 | RANGAP1 | Ran gtpase activating protein 1 | 0.013752 |
| ENSG00000146411 | SLC2A12 | Solute carrier family 2 member 12 | 0.013844 |
| ENSG00000100290 | BIK | BCL2 interacting killer | 0.013844 |
| ENSG00000181045 | SLC26A11 | Solute carrier family 26 member 11 | 0.013926 |
| ENSG00000243749 | TMEM35B | Transmembrane protein 35B | 0.014452 |
| ENSG00000163535 | SGO2 | Shugoshin 2 | 0.014452 |
| ENSG00000136874 | STX17 | Syntaxin 17 | 0.014537 |
| ENSG00000141232 | TOB1 | Transducer of ERBB2, 1 | 0.014837 |

| | | | |
|---|---|---|---|
| ENSG00000198331 | HYLS1 | HYLS1 centriolar and ciliogenesis associated | 0.015192 |
| ENSG00000160460 | AC020929.1 | Spectrin beta, non-erythrocytic 4 | 0.015222 |
| ENSG00000157184 | CPT2 | Carnitine palmitoyltransferase 2 | 0.015481 |
| ENSG00000066926 | FECH | Ferrochelatase | 0.0155 |
| ENSG00000134070 | IRAK2 | Interleukin 1 receptor associated kinase 2 | 0.0155 |
| ENSG00000197302 | ZNF720 | Zinc finger protein 720 | 0.0155 |
| ENSG00000060656 | PTPRU | Protein tyrosine phosphatase receptor type U | 0.0155 |
| ENSG00000065320 | NTN1 | Netrin 1 | 0.0155 |
| ENSG00000067369 | TP53BP1 | Tumor protein p53 binding protein 1 | 0.015728 |
| ENSG00000178184 | PARD6G | Par-6 family cell polarity regulator gamma | 0.015736 |
| ENSG00000138653 | NDST4 | N-deacetylase and N-sulfotransferase 4 | 0.015948 |
| ENSG00000170667 | RASA4B | RAS p21 protein activator 4B | 0.015948 |
| ENSG00000166145 | SPINT1 | Serine peptidase inhibitor, Kunitz type 1 | 0.015996 |
| ENSG00000213918 | DNASE1 | Deoxyribonuclease 1 | 0.015998 |
| ENSG00000064961 | HMG20B | High mobility group 20B | 0.016158 |
| ENSG00000031003 | FAM13B | Family with sequence similarity 13 member B | 0.016299 |
| ENSG00000112981 | NME5 | NME/NM23 family member 5 | 0.016486 |
| ENSG00000285827 | AP001267.5 | Novel protein | 0.017593 |
| ENSG00000128917 | DLL4 | Delta like canonical Notch ligand 4 | 0.017956 |
| ENSG00000183763 | TRAIP | TRAF interacting protein | 0.018133 |
| ENSG00000138182 | KIF20B | Kinesin family member 20B | 0.018192 |
| ENSG00000187688 | TRPV2 | Transient receptor potential cation channel subfamily V member 2 | 0.018419 |
| ENSG00000163701 | IL17RE | Interleukin 17 receptor E | 0.018529 |
| ENSG00000137207 | YIPF3 | Yip1 domain family member 3 | 0.018805 |
| ENSG00000165280 | VCP | Valosin containing protein | 0.019236 |
| ENSG00000138641 | HERC3 | HECT and RLD domain containing E3 ubiquitin protein ligase 3 | 0.019692 |
| ENSG00000111058 | ACSS3 | Acyl-coa synthetase short chain family member 3 | 0.019948 |
| ENSG00000175854 | SWI5 | SWI5 homologous recombination repair protein | 0.020255 |
| ENSG00000141380 | SS18 | SS18 subunit of BAF chromatin remodeling complex | 0.020397 |
| ENSG00000166816 | LDHD | Lactate dehydrogenase D | 0.020625 |
| ENSG00000175352 | NRIP3 | Nuclear receptor interacting protein 3 | 0.020865 |
| ENSG00000156970 | BUB1B | BUB1 mitotic checkpoint serine/threonine kinase B | 0.020959 |
| ENSG00000027869 | SH2D2A | SH2 domain containing 2A | 0.020959 |
| ENSG00000165209 | STRBP | Spermatid perinuclear RNA binding protein | 0.021159 |
| ENSG00000153237 | CCDC148 | Coiled-coil domain containing 148 | 0.021195 |
| ENSG00000165113 | GKAP1 | G kinase anchoring protein 1 | 0.022053 |
| ENSG00000240771 | ARHGEF25 | Rho guanine nucleotide exchange factor 25 | 0.022312 |
| ENSG00000161800 | RACGAP1 | Rac gtpase activating protein 1 | 0.022425 |
| ENSG00000132879 | FBXO44 | F-box protein 44 | 0.022425 |
| ENSG00000171236 | LRG1 | Leucine rich alpha-2-glycoprotein 1 | 0.022425 |
| ENSG00000171680 | PLEKHG5 | Pleckstrin homology and rhogef domain containing G5 | 0.022753 |
| ENSG00000141376 | BCAS3 | BCAS3 microtubule associated cell migration factor | 0.023124 |
| ENSG00000166887 | VPS39 | VPS39 subunit of HOPS complex | 0.023249 |
| ENSG00000115226 | FNDC4 | Fibronectin type III domain containing 4 | 0.023754 |
| ENSG00000135506 | OS9 | OS9 endoplasmic reticulum lectin | 0.024111 |
| ENSG00000043143 | JADE2 | Jade family PHD finger 2 | 0.024111 |
| ENSG00000163521 | GLB1L | Galactosidase beta 1 like | 0.024677 |
| ENSG00000104756 | KCTD9 | Potassium channel tetramerization domain containing 9 | 0.02474 |
| ENSG00000101298 | SNPH | Syntaphilin | 0.02474 |

| | | | |
|---|---|---|---|
| ENSG00000185745 | IFIT1 | Interferon induced protein with tetratricopeptide repeats 1 | 0.024853 |
| ENSG00000112561 | TFEB | Transcription factor EB | 0.024856 |
| ENSG00000138166 | DUSP5 | Dual specificity phosphatase 5 | 0.024897 |
| ENSG00000122483 | CCDC18 | Coiled-coil domain containing 18 | 0.024897 |
| ENSG00000135828 | RNASEL | Ribonuclease L | 0.024901 |
| ENSG00000175155 | YPEL2 | Yippee like 2 | 0.024995 |
| ENSG00000129003 | VPS13C | Vacuolar protein sorting 13 homolog C | 0.025037 |
| ENSG00000138036 | DYNC2LI1 | Dynein cytoplasmic 2 light intermediate chain 1 | 0.02518 |
| ENSG00000174307 | PHLDA3 | Pleckstrin homology like domain family A member 3 | 0.02518 |
| ENSG00000157954 | WIPI2 | WD repeat domain, phosphoinositide interacting 2 | 0.025197 |
| ENSG00000165475 | CRYL1 | Crystallin lambda 1 | 0.025231 |
| ENSG00000172738 | TMEM217 | Transmembrane protein 217 | 0.0253 |
| ENSG00000125347 | IRF1 | Interferon regulatory factor 1 | 0.025727 |
| ENSG00000128604 | IRF5 | Interferon regulatory factor 5 | 0.025768 |
| ENSG00000066084 | DIP2B | Disco interacting protein 2 homolog B | 0.025984 |
| ENSG00000182134 | TDRKH | Tudor and KH domain containing | 0.025984 |
| ENSG00000273045 | C2orf15 | Chromosome 2 open reading frame 15 | 0.026342 |
| ENSG00000159592 | GPBP1L1 | GC-rich promoter binding protein 1 like 1 | 0.026379 |
| ENSG00000185669 | SNAI3 | Snail family transcriptional repressor 3 | 0.026379 |
| ENSG00000171130 | ATP6V0E2 | Atpase H+ transporting V0 subunit e2 | 0.027007 |
| ENSG00000102038 | SMARCA1 | SWI/SNF related, matrix associated, actin dependent regulator of chromatin, subfamily a, member 1 | 0.027438 |
| ENSG00000211584 | SLC48A1 | Solute carrier family 48 member 1 | 0.028058 |
| ENSG00000078304 | PPP2R5C | Protein phosphatase 2 regulatory subunit b'gamma | 0.028337 |
| ENSG00000189241 | TSPYL1 | TSPY like 1 | 0.028611 |
| ENSG00000081377 | CDC14B | Cell division cycle 14B | 0.028971 |
| ENSG00000178078 | STAP2 | Signal transducing adaptor family member 2 | 0.028971 |
| ENSG00000138160 | KIF11 | Kinesin family member 11 | 0.029221 |
| ENSG00000100577 | GSTZ1 | Glutathione S-transferase zeta 1 | 0.029284 |
| ENSG00000109654 | TRIM2 | Tripartite motif containing 2 | 0.029637 |
| ENSG00000172508 | CARNS1 | Carnosine synthase 1 | 0.03065 |
| ENSG00000283050 | GTF2IP12 | General transcription factor iii pseudogene 12 | 0.031757 |
| ENSG00000054267 | ARID4B | AT-rich interaction domain 4B | 0.031907 |
| ENSG00000156042 | CFAP70 | Cilia and flagella associated protein 70 | 0.032416 |
| ENSG00000198842 | DUSP27 | Dual specificity phosphatase 27, atypical | 0.032807 |
| ENSG00000188610 | FAM72B | Family with sequence similarity 72 member B | 0.032955 |
| ENSG00000242294 | STAG3L5P | Stromal antigen 3-like 5 pseudogene | 0.033107 |
| ENSG00000186193 | SAPCD2 | Suppressor APC domain containing 2 | 0.033107 |
| ENSG00000197056 | ZMYM1 | Zinc finger MYM-type containing 1 | 0.034281 |
| ENSG00000072954 | TMEM38A | Transmembrane protein 38A | 0.034347 |
| ENSG00000054219 | LY75 | Lymphocyte antigen 75 | 0.035345 |
| ENSG00000278023 | RDM1 | RAD52 motif containing 1 | 0.035475 |
| ENSG00000137807 | KIF23 | Kinesin family member 23 | 0.036362 |
| ENSG00000120733 | KDM3B | Lysine demethylase 3B | 0.036419 |
| ENSG00000173992 | CCS | Copper chaperone for superoxide dismutase | 0.036822 |
| ENSG00000116793 | PHTF1 | Putative homeodomain transcription factor 1 | 0.037191 |
| ENSG00000148950 | IMMP1L | Inner mitochondrial membrane peptidase subunit 1 | 0.037191 |
| ENSG00000176907 | TCIM | Transcriptional and immune response regulator | 0.037191 |
| ENSG00000183682 | BMP8A | Bone morphogenetic protein 8a | 0.037551 |
| ENSG00000183624 | HMCES | 5-hydroxymethylcytosine binding, ES cell specific | 0.037593 |

| | | | |
|---|---|---|---|
| ENSG00000105327 | BBC3 | BCL2 binding component 3 | 0.037696 |
| ENSG00000174125 | TLR1 | Toll like receptor 1 | 0.037722 |
| ENSG00000260300 | | | 0.037731 |
| ENSG00000130477 | UNC13A | Unc-13 homolog A | 0.037771 |
| ENSG00000172831 | CES2 | Carboxylesterase 2 | 0.037908 |
| ENSG00000162643 | WDR63 | WD repeat domain 63 | 0.038078 |
| ENSG00000140451 | PIF1 | PIF1 5'-to-3' DNA helicase | 0.038158 |
| ENSG00000205403 | CFI | Complement factor I | 0.03888 |
| ENSG00000136158 | SPRY2 | Sprouty RTK signaling antagonist 2 | 0.038978 |
| ENSG00000123975 | CKS2 | CDC28 protein kinase regulatory subunit 2 | 0.039119 |
| ENSG00000112294 | ALDH5A1 | Aldehyde dehydrogenase 5 family member A1 | 0.040097 |
| ENSG00000170634 | ACYP2 | Acylphosphatase 2 | 0.040295 |
| ENSG00000084764 | MAPRE3 | Microtubule associated protein RP/EB family member 3 | 0.040436 |
| ENSG00000134202 | GSTM3 | Glutathione S-transferase mu 3 | 0.040533 |
| ENSG00000141574 | SECTM1 | Secreted and transmembrane 1 | 0.040735 |
| ENSG00000170734 | POLH | DNA polymerase eta | 0.040897 |
| ENSG00000171812 | COL8A2 | Collagen type VIII alpha 2 chain | 0.040897 |
| ENSG00000205089 | CCNI2 | Cyclin I family member 2 | 0.040897 |
| ENSG00000150977 | RILPL2 | Rab interacting lysosomal protein like 2 | 0.041042 |
| ENSG00000170542 | SERPINB9 | Serpin family B member 9 | 0.041584 |
| ENSG00000115129 | TP53I3 | Tumor protein p53 inducible protein 3 | 0.042293 |
| ENSG00000101347 | SAMHD1 | SAM and HD domain containing deoxynucleoside triphosphate triphosphohydrolase 1 | 0.042377 |
| ENSG00000100897 | DCAF11 | DDB1 and CUL4 associated factor 11 | 0.042607 |
| ENSG00000213186 | TRIM59 | Tripartite motif containing 59 | 0.042807 |
| ENSG00000125744 | RTN2 | Reticulon 2 | 0.04398 |
| ENSG00000103490 | PYCARD | PYD and CARD domain containing | 0.04398 |
| ENSG00000135525 | MAP7 | Microtubule associated protein 7 | 0.04406 |
| ENSG00000130363 | RSPH3 | Radial spoke head 3 | 0.044069 |
| ENSG00000128394 | APOBEC3F | Apolipoprotein B mrna editing enzyme catalytic subunit 3F | 0.045478 |
| ENSG00000213930 | GALT | Galactose-1-phosphate uridylyltransferase | 0.046342 |
| ENSG00000231551 | AC245100.4 | Phosphodiesterase 4D interacting protein (PDE4DIP) pseudogene | 0.046342 |
| ENSG00000118292 | C1orf54 | Chromosome 1 open reading frame 54 | 0.04641 |
| ENSG00000136861 | CDK5RAP2 | CDK5 regulatory subunit associated protein 2 | 0.046519 |
| ENSG00000182511 | FES | FES proto-oncogene, tyrosine kinase | 0.046523 |
| ENSG00000197165 | SULT1A2 | Sulfotransferase family 1A member 2 | 0.04733 |
| ENSG00000006283 | CACNA1G | Calcium voltage-gated channel subunit alpha1 G | 0.047333 |
| ENSG00000236609 | ZNF853 | Zinc finger protein 853 | 0.047432 |
| ENSG00000148057 | IDNK | IDNK gluconokinase | 0.047852 |
| ENSG00000137135 | ARHGEF39 | Rho guanine nucleotide exchange factor 39 | 0.047866 |
| ENSG00000160014 | CALM3 | Calmodulin 3 | 0.047954 |
| ENSG00000108771 | DHX58 | Dexh-box helicase 58 | 0.048092 |
| ENSG00000100027 | YPEL1 | Yippee like 1 | 0.048719 |
| ENSG00000173193 | PARP14 | Poly(ADP-ribose) polymerase family member 14 | 0.049401 |
| ENSG00000119508 | NR4A3 | Nuclear receptor subfamily 4 group A member 3 | 0.049457 |
| ENSG00000255150 | EID3 | EP300 interacting inhibitor of differentiation 3 | 0.049717 |
| ENSG00000078487 | ZCWPW1 | Zinc finger CW-type and PWWP domain containing 1 | 0.049821 |
| **Down-Regulated Genes↓** | | | |
| ENSG00000107562 | CXCL12 | C-X-C motif chemokine ligand 12 | 4.22E-27 |

| | | | |
|---|---|---|---|
| ENSG00000168298 | H1-4 | H1.4 linker histone, cluster member | 3.66E-25 |
| ENSG00000146674 | IGFBP3 | Insulin like growth factor binding protein 3 | 1.84E-24 |
| ENSG00000184357 | H1-5 | H1.5 linker histone, cluster member | 1.35E-22 |
| ENSG00000277075 | H2AC8 | H2A clustered histone 8 | 3.10E-22 |
| ENSG00000197153 | H3C12 | H3 clustered histone 12 | 2.98E-20 |
| ENSG00000184260 | H2AC20 | H2A clustered histone 20 | 1.11E-18 |
| ENSG00000184270 | H2AC21 | H2A clustered histone 21 | 2.48E-18 |
| ENSG00000183688 | RFLNB | Refilin B | 6.46E-18 |
| ENSG00000276903 | H2AC16 | H2A clustered histone 16 | 1.21E-17 |
| ENSG00000183598 | H3C13 | H3 clustered histone 13 | 2.42E-17 |
| ENSG00000278463 | H2AC4 | H2A clustered histone 4 | 3.72E-17 |
| ENSG00000187837 | H1-2 | H1.2 linker histone, cluster member | 1.41E-16 |
| ENSG00000154678 | PDE1C | Phosphodiesterase 1C | 7.97E-16 |
| ENSG00000092969 | TGFB2 | Transforming growth factor beta 2 | 1.00E-14 |
| ENSG00000127863 | TNFRSF19 | TNF receptor superfamily member 19 | 6.99E-14 |
| ENSG00000286522 | H3C2 | H3 clustered histone 2 | 1.17E-13 |
| ENSG00000196747 | H2AC13 | H2A clustered histone 13 | 1.86E-13 |
| ENSG00000124575 | H1-3 | H1.3 linker histone, cluster member | 1.57E-12 |
| ENSG00000278677 | H2AC17 | H2A clustered histone 17 | 1.92E-12 |
| ENSG00000273802 | H2BC8 | H2B clustered histone 8 | 2.22E-12 |
| ENSG00000100504 | PYGL | Glycogen phosphorylase L | 3.50E-12 |
| ENSG00000203814 | H2BC18 | H2B clustered histone 18 | 8.56E-12 |
| ENSG00000274997 | H2AC12 | H2A clustered histone 12 | 1.07E-11 |
| ENSG00000275379 | H3C11 | H3 clustered histone 11 | 1.32E-11 |
| ENSG00000101938 | CHRDL1 | Chordin like 1 | 2.08E-11 |
| ENSG00000180596 | H2BC4 | H2B clustered histone 4 | 2.73E-11 |
| ENSG00000164125 | GASK1B | Golgi associated kinase 1B | 2.95E-11 |
| ENSG00000185130 | H2BC13 | H2B clustered histone 13 | 5.61E-11 |
| ENSG00000277224 | H2BC7 | H2B clustered histone 7 | 6.63E-11 |
| ENSG00000277157 | H4C4 | H4 clustered histone 4 | 1.58E-10 |
| ENSG00000272196 | H2AC19 | H2A clustered histone 19 | 2.65E-10 |
| ENSG00000145681 | HAPLN1 | Hyaluronan and proteoglycan link protein 1 | 4.93E-10 |
| ENSG00000196787 | H2AC11 | H2A clustered histone 11 | 5.71E-10 |
| ENSG00000128422 | KRT17 | Keratin 17 | 5.71E-10 |
| ENSG00000162909 | CAPN2 | Calpain 2 | 5.71E-10 |
| ENSG00000282988 | AL031777.2 | Novel protein | 7.51E-10 |
| ENSG00000099194 | SCD | Stearoyl-coa desaturase | 1.16E-09 |
| ENSG00000275714 | H3C1 | H3 clustered histone 1 | 1.52E-09 |
| ENSG00000203811 | H3C14 | H3 clustered histone 14 | 1.67E-09 |
| ENSG00000145604 | SKP2 | S-phase kinase associated protein 2 | 2.17E-09 |
| ENSG00000180730 | SHISA2 | Shisa family member 2 | 2.93E-09 |
| ENSG00000197903 | H2BC12 | H2B clustered histone 12 | 2.99E-09 |
| ENSG00000156466 | GDF6 | Growth differentiation factor 6 | 3.77E-09 |
| ENSG00000105974 | CAV1 | Caveolin 1 | 3.90E-09 |
| ENSG00000196866 | H2AC7 | H2A clustered histone 7 | 3.97E-09 |
| ENSG00000044524 | EPHA3 | EPH receptor A3 | 4.04E-09 |
| ENSG00000183287 | CCBE1 | Collagen and calcium binding EGF domains 1 | 4.37E-09 |
| ENSG00000072041 | SLC6A15 | Solute carrier family 6 member 15 | 5.21E-09 |
| ENSG00000124610 | H1-1 | H1.1 linker histone, cluster member | 5.75E-09 |
| ENSG00000158373 | H2BC5 | H2B clustered histone 5 | 7.51E-09 |
| ENSG00000180914 | OXTR | Oxytocin receptor | 1.06E-08 |

| | | | |
|---|---|---|---|
| ENSG00000179431 | FJX1 | Four-jointed box kinase 1 | 1.44E-08 |
| ENSG00000276368 | H2AC14 | H2A clustered histone 14 | 2.23E-08 |
| ENSG00000270882 | H4C14 | H4 clustered histone 14 | 2.31E-08 |
| ENSG00000116774 | OLFML3 | Olfactomedin like 3 | 2.58E-08 |
| ENSG00000147202 | DIAPH2 | Diaphanous related formin 2 | 3.30E-08 |
| ENSG00000120158 | RCL1 | RNA terminal phosphate cyclase like 1 | 4.10E-08 |
| ENSG00000162849 | KIF26B | Kinesin family member 26B | 4.90E-08 |
| ENSG00000117597 | UTP25 | UTP25 small subunit processor component | 5.12E-08 |
| ENSG00000074800 | ENO1 | Enolase 1 | 6.29E-08 |
| ENSG00000137124 | ALDH1B1 | Aldehyde dehydrogenase 1 family member B1 | 7.68E-08 |
| ENSG00000134986 | NREP | Neuronal regeneration related protein | 8.06E-08 |
| ENSG00000120217 | CD274 | CD274 molecule | 8.42E-08 |
| ENSG00000107796 | ACTA2 | Actin alpha 2, smooth muscle | 1.23E-07 |
| ENSG00000141052 | MYOCD | Myocardin | 1.40E-07 |
| ENSG00000168672 | LRATD2 | LRAT domain containing 2 | 1.94E-07 |
| ENSG00000115380 | EFEMP1 | EGF containing fibulin extracellular matrix protein 1 | 1.96E-07 |
| ENSG00000125384 | PTGER2 | Prostaglandin E receptor 2 | 2.10E-07 |
| ENSG00000135763 | URB2 | URB2 ribosome biogenesis homolog | 2.53E-07 |
| ENSG00000125485 | DDX31 | DEAD-box helicase 31 | 2.65E-07 |
| ENSG00000274641 | H2BC17 | H2B clustered histone 17 | 4.05E-07 |
| ENSG00000174371 | EXO1 | Exonuclease 1 | 4.11E-07 |
| ENSG00000123213 | NLN | Neurolysin | 5.05E-07 |
| ENSG00000170153 | RNF150 | Ring finger protein 150 | 5.76E-07 |
| ENSG00000278828 | H3C10 | H3 clustered histone 10 | 9.34E-07 |
| ENSG00000113140 | SPARC | Secreted protein acidic and cysteine rich | 9.34E-07 |
| ENSG00000234289 | H2BS1 | H2B.S histone 1 | 1.01E-06 |
| ENSG00000019549 | SNAI2 | Snail family transcriptional repressor 2 | 1.12E-06 |
| ENSG00000146477 | SLC22A3 | Solute carrier family 22 member 3 | 1.16E-06 |
| ENSG00000120658 | ENOX1 | Ecto-NOX disulfide-thiol exchanger 1 | 1.17E-06 |
| ENSG00000139278 | GLIPR1 | GLI pathogenesis related 1 | 1.17E-06 |
| ENSG00000129038 | LOXL1 | Lysyl oxidase like 1 | 1.17E-06 |
| ENSG00000144891 | AGTR1 | Angiotensin II receptor type 1 | 1.27E-06 |
| ENSG00000134775 | FHOD3 | Formin homology 2 domain containing 3 | 1.27E-06 |
| ENSG00000076248 | UNG | Uracil DNA glycosylase | 1.27E-06 |
| ENSG00000275221 | H2AC15 | H2A clustered histone 15 | 1.32E-06 |
| ENSG00000118596 | SLC16A7 | Solute carrier family 16 member 7 | 1.59E-06 |
| ENSG00000133019 | CHRM3 | Cholinergic receptor muscarinic 3 | 1.89E-06 |
| ENSG00000135549 | PKIB | Camp-dependent protein kinase inhibitor beta | 2.03E-06 |
| ENSG00000135362 | PRR5L | Proline rich 5 like | 2.50E-06 |
| ENSG00000153993 | SEMA3D | Semaphorin 3D | 3.13E-06 |
| ENSG00000189057 | FAM111B | Family with sequence similarity 111 member B | 3.23E-06 |
| ENSG00000144681 | STAC | SH3 and cysteine rich domain | 3.27E-06 |
| ENSG00000082512 | TRAF5 | TNF receptor associated factor 5 | 4.26E-06 |
| ENSG00000170017 | ALCAM | Activated leukocyte cell adhesion molecule | 4.42E-06 |
| ENSG00000243955 | GSTA1 | Glutathione S-transferase alpha 1 | 4.56E-06 |
| ENSG00000196456 | ZNF775 | Zinc finger protein 775 | 4.56E-06 |
| ENSG00000154734 | ADAMTS1 | ADAM metallopeptidase with thrombospondin type 1 motif 1 | 4.90E-06 |
| ENSG00000178409 | BEND3 | BEN domain containing 3 | 4.90E-06 |
| ENSG00000101868 | POLA1 | DNA polymerase alpha 1, catalytic subunit | 5.53E-06 |
| ENSG00000197238 | H4C11 | H4 clustered histone 11 | 5.88E-06 |

| | | | |
|---|---|---|---|
| ENSG00000115648 | MLPH | Melanophilin | 6.14E-06 |
| ENSG00000003989 | SLC7A2 | Solute carrier family 7 member 2 | 6.14E-06 |
| ENSG00000133107 | TRPC4 | Transient receptor potential cation channel subfamily C member 4 | 6.14E-06 |
| ENSG00000134285 | FKBP11 | FKBP prolyl isomerase 11 | 6.17E-06 |
| ENSG00000157168 | NRG1 | Neuregulin 1 | 6.50E-06 |
| ENSG00000119285 | HEATR1 | HEAT repeat containing 1 | 6.72E-06 |
| ENSG00000101361 | NOP56 | NOP56 ribonucleoprotein | 7.16E-06 |
| ENSG00000150687 | PRSS23 | Serine protease 23 | 7.20E-06 |
| ENSG00000185760 | KCNQ5 | Potassium voltage-gated channel subfamily Q member 5 | 7.31E-06 |
| ENSG00000151376 | ME3 | Malic enzyme 3 | 7.95E-06 |
| ENSG00000164619 | BMPER | BMP binding endothelial regulator | 9.52E-06 |
| ENSG00000115461 | IGFBP5 | Insulin like growth factor binding protein 5 | 9.62E-06 |
| ENSG00000091986 | CCDC80 | Coiled-coil domain containing 80 | 9.62E-06 |
| ENSG00000178695 | KCTD12 | Potassium channel tetramerization domain containing 12 | 9.62E-06 |
| ENSG00000182240 | BACE2 | Beta-secretase 2 | 9.62E-06 |
| ENSG00000172197 | MBOAT1 | Membrane bound O-acyltransferase domain containing 1 | 1.01E-05 |
| ENSG00000158406 | H4C8 | H4 clustered histone 8 | 1.07E-05 |
| ENSG00000143179 | UCK2 | Uridine-cytidine kinase 2 | 1.28E-05 |
| ENSG00000104356 | POP1 | POP1 homolog, ribonuclease P/MRP subunit | 1.36E-05 |
| ENSG00000196950 | SLC39A10 | Solute carrier family 39 member 10 | 1.68E-05 |
| ENSG00000180573 | H2AC6 | H2A clustered histone 6 | 1.76E-05 |
| ENSG00000182197 | EXT1 | Exostosin glycosyltransferase 1 | 1.86E-05 |
| ENSG00000233822 | H2BC15 | H2B clustered histone 15 | 1.91E-05 |
| ENSG00000080823 | MOK | MOK protein kinase | 2.18E-05 |
| ENSG00000136111 | TBC1D4 | TBC1 domain family member 4 | 2.48E-05 |
| ENSG00000234571 | H2BP2 | H2B histone pseudogene 2 | 2.54E-05 |
| ENSG00000184678 | H2BC21 | H2B clustered histone 21 | 2.65E-05 |
| ENSG00000116679 | IVNS1ABP | Influenza virus NS1A binding protein | 2.65E-05 |
| ENSG00000120254 | MTHFD1L | Methylenetetrahydrofolate dehydrogenase (NADP+ dependent) 1 like | 2.83E-05 |
| ENSG00000120708 | TGFBI | Transforming growth factor beta induced | 2.83E-05 |
| ENSG00000145934 | TENM2 | Teneurin transmembrane protein 2 | 2.94E-05 |
| ENSG00000154646 | TMPRSS15 | Transmembrane serine protease 15 | 3.00E-05 |
| ENSG00000135919 | SERPINE2 | Serpin family E member 2 | 3.03E-05 |
| ENSG00000128510 | CPA4 | Carboxypeptidase A4 | 3.04E-05 |
| ENSG00000122870 | BICC1 | Bicc family RNA binding protein 1 | 3.37E-05 |
| ENSG00000144354 | CDCA7 | Cell division cycle associated 7 | 4.30E-05 |
| ENSG00000120594 | PLXDC2 | Plexin domain containing 2 | 4.44E-05 |
| ENSG00000060982 | BCAT1 | Branched chain amino acid transaminase 1 | 4.53E-05 |
| ENSG00000070404 | FSTL3 | Follistatin like 3 | 4.53E-05 |
| ENSG00000172927 | MYEOV | Myeloma overexpressed | 4.61E-05 |
| ENSG00000111057 | KRT18 | Keratin 18 | 4.70E-05 |
| ENSG00000183010 | PYCR1 | Pyrroline-5-carboxylate reductase 1 | 5.04E-05 |
| ENSG00000168003 | SLC3A2 | Solute carrier family 3 member 2 | 5.06E-05 |
| ENSG00000164176 | EDIL3 | EGF like repeats and discoidin domains 3 | 5.33E-05 |
| ENSG00000139211 | AMIGO2 | Adhesion molecule with Ig like domain 2 | 5.45E-05 |
| ENSG00000159131 | GART | Phosphoribosylglycinamide formyltransferase, phosphoribosylglycinamide synthetase, phosphoribosylaminoimidazole synthetase | 5.66E-05 |
| ENSG00000180535 | BHLHA15 | Basic helix-loop-helix family member a15 | 5.69E-05 |

| | | | |
|---|---|---|---|
| ENSG00000119537 | KDSR | 3-ketodihydrosphingosine reductase | 6.27E-05 |
| ENSG00000187840 | EIF4EBP1 | Eukaryotic translation initiation factor 4E binding protein 1 | 6.85E-05 |
| ENSG00000124635 | H2BC11 | H2B clustered histone 11 | 7.05E-05 |
| ENSG00000145675 | PIK3R1 | Phosphoinositide-3-kinase regulatory subunit 1 | 8.01E-05 |
| ENSG00000115232 | ITGA4 | Integrin subunit alpha 4 | 8.35E-05 |
| ENSG00000204291 | COL15A1 | Collagen type XV alpha 1 chain | 9.03E-05 |
| ENSG00000181751 | C5orf30 | Chromosome 5 open reading frame 30 | 9.28E-05 |
| ENSG00000113739 | STC2 | Stanniocalcin 2 | 9.45E-05 |
| ENSG00000136261 | BZW2 | Basic leucine zipper and W2 domains 2 | 9.62E-05 |
| ENSG00000265107 | GJA5 | Gap junction protein alpha 5 | 0.000101 |
| ENSG00000117394 | SLC2A1 | Solute carrier family 2 member 1 | 0.000107 |
| ENSG00000161647 | MPP3 | Membrane palmitoylated protein 3 | 0.000113 |
| ENSG00000157483 | MYO1E | Myosin IE | 0.000134 |
| ENSG00000102271 | KLHL4 | Kelch like family member 4 | 0.000142 |
| ENSG00000128050 | PAICS | Phosphoribosylaminoimidazole carboxylase and phosphoribosylaminoimidazolesuccinocarboxamide synthase | 0.000142 |
| ENSG00000132669 | RIN2 | Ras and Rab interactor 2 | 0.000147 |
| ENSG00000153823 | PID1 | Phosphotyrosine interaction domain containing 1 | 0.000148 |
| ENSG00000170854 | RIOX2 | Ribosomal oxygenase 2 | 0.000154 |
| ENSG00000141569 | TRIM65 | Tripartite motif containing 65 | 0.000154 |
| ENSG00000122420 | PTGFR | Prostaglandin F receptor | 0.000155 |
| ENSG00000109861 | CTSC | Cathepsin C | 0.000173 |
| ENSG00000197837 | H4-16 | H4 histone 16 | 0.000184 |
| ENSG00000112276 | BVES | Blood vessel epicardial substance | 0.000192 |
| ENSG00000198796 | ALPK2 | Alpha kinase 2 | 0.000194 |
| ENSG00000111885 | MAN1A1 | Mannosidase alpha class 1A member 1 | 0.000195 |
| ENSG00000183722 | LHFPL6 | LHFPL tetraspan subfamily member 6 | 0.000195 |
| ENSG00000054277 | OPN3 | Opsin 3 | 0.000198 |
| ENSG00000113013 | HSPA9 | Heat shock protein family A (Hsp70) member 9 | 0.000203 |
| ENSG00000186854 | TRABD2A | Trab domain containing 2A | 0.000209 |
| ENSG00000156265 | MAP3K7CL | MAP3K7 C-terminal like | 0.000209 |
| ENSG00000145416 | MARCHF1 | Membrane associated ring-CH-type finger 1 | 0.000212 |
| ENSG00000176697 | BDNF | Brain derived neurotrophic factor | 0.000225 |
| ENSG00000275126 | H4C13 | H4 clustered histone 13 | 0.000229 |
| ENSG00000178776 | C5orf46 | Chromosome 5 open reading frame 46 | 0.000243 |
| ENSG00000050344 | NFE2L3 | Nuclear factor, erythroid 2 like 3 | 0.000254 |
| ENSG00000243444 | | | 0.000257 |
| ENSG00000181418 | DDN | Dendrin | 0.000262 |
| ENSG00000186480 | INSIG1 | Insulin induced gene 1 | 0.000279 |
| ENSG00000156398 | SFXN2 | Sideroflexin 2 | 0.000292 |
| ENSG00000140416 | TPM1 | Tropomyosin 1 | 0.000298 |
| ENSG00000152661 | GJA1 | Gap junction protein alpha 1 | 0.000303 |
| ENSG00000179104 | TMTC2 | Transmembrane O-mannosyltransferase targeting cadherins 2 | 0.00032 |
| ENSG00000187193 | MT1X | Metallothionein 1X | 0.000331 |
| ENSG00000111186 | WNT5B | Wnt family member 5B | 0.00034 |
| ENSG00000106105 | GARS1 | Glycyl-trna synthetase 1 | 0.000342 |
| ENSG00000197061 | H4C3 | H4 clustered histone 3 | 0.00037 |
| ENSG00000117877 | CD3EAP | CD3e molecule associated protein | 0.000394 |

| | | | |
|---|---|---|---|
| ENSG00000132768 | DPH2 | Diphthamide biosynthesis 2 | 0.000403 |
| ENSG00000206190 | ATP10A | Atpase phospholipid transporting 10A (putative) | 0.000403 |
| ENSG00000166333 | ILK | Integrin linked kinase | 0.000405 |
| ENSG00000189212 | DPY19L2P1 | DPY19L2 pseudogene 1 | 0.000415 |
| ENSG00000150051 | MKX | Mohawk homeobox | 0.00042 |
| ENSG00000071575 | TRIB2 | Tribbles pseudokinase 2 | 0.00042 |
| ENSG00000166002 | SMCO4 | Single-pass membrane protein with coiled-coil domains 4 | 0.000429 |
| ENSG00000159176 | CSRP1 | Cysteine and glycine rich protein 1 | 0.00044 |
| ENSG00000157064 | NMNAT2 | Nicotinamide nucleotide adenylyltransferase 2 | 0.000459 |
| ENSG00000244067 | GSTA2 | Glutathione S-transferase alpha 2 | 0.00046 |
| ENSG00000082516 | GEMIN5 | Gem nuclear organelle associated protein 5 | 0.000482 |
| ENSG00000141384 | TAF4B | TATA-box binding protein associated factor 4b | 0.000487 |
| ENSG00000198947 | DMD | Dystrophin | 0.000543 |
| ENSG00000143314 | MRPL24 | Mitochondrial ribosomal protein L24 | 0.000546 |
| ENSG00000148484 | RSU1 | Ras suppressor protein 1 | 0.000547 |
| ENSG00000278705 | H4C2 | H4 clustered histone 2 | 0.000554 |
| ENSG00000026559 | KCNG1 | Potassium voltage-gated channel modifier subfamily G member 1 | 0.000554 |
| ENSG00000072274 | TFRC | Transferrin receptor | 0.000581 |
| ENSG00000125257 | ABCC4 | ATP binding cassette subfamily C member 4 | 0.000589 |
| ENSG00000101546 | RBFA | Ribosome binding factor A | 0.000589 |
| ENSG00000170515 | PA2G4 | Proliferation-associated 2G4 | 0.00059 |
| ENSG00000105810 | CDK6 | Cyclin dependent kinase 6 | 0.000593 |
| ENSG00000106366 | SERPINE1 | Serpin family E member 1 | 0.000603 |
| ENSG00000120800 | UTP20 | UTP20 small subunit processome component | 0.000614 |
| ENSG00000091527 | CDV3 | CDV3 homolog | 0.000625 |
| ENSG00000163083 | INHBB | Inhibin subunit beta B | 0.000634 |
| ENSG00000115107 | STEAP3 | STEAP3 metalloreductase | 0.000634 |
| ENSG00000238243 | OR2W3 | Olfactory receptor family 2 subfamily W member 3 | 0.000634 |
| ENSG00000189007 | ADAT2 | Adenosine deaminase trna specific 2 | 0.000662 |
| ENSG00000162419 | GMEB1 | Glucocorticoid modulatory element binding protein 1 | 0.000662 |
| ENSG00000143157 | POGK | Pogo transposable element derived with KRAB domain | 0.000797 |
| ENSG00000053372 | MRTO4 | MRT4 homolog, ribosome maturation factor | 0.000798 |
| ENSG00000121057 | AKAP1 | A-kinase anchoring protein 1 | 0.000807 |
| ENSG00000125458 | NT5C | 5', 3'-nucleotidase, cytosolic | 0.000819 |
| ENSG00000196730 | DAPK1 | Death associated protein kinase 1 | 0.000839 |
| ENSG00000164649 | CDCA7L | Cell division cycle associated 7 like | 0.000853 |
| ENSG00000140945 | CDH13 | Cadherin 13 | 0.000869 |
| ENSG00000106628 | POLD2 | DNA polymerase delta 2, accessory subunit | 0.000872 |
| ENSG00000170876 | TMEM43 | Transmembrane protein 43 | 0.000873 |
| ENSG00000152256 | PDK1 | Pyruvate dehydrogenase kinase 1 | 0.000879 |
| ENSG00000149485 | FADS1 | Fatty acid desaturase 1 | 0.000887 |
| ENSG00000255561 | FDXACB1 | Ferredoxin-fold anticodon binding domain containing 1 | 0.000887 |
| ENSG00000072682 | P4HA2 | Prolyl 4-hydroxylase subunit alpha 2 | 0.000892 |
| ENSG00000106799 | TGFBR1 | Transforming growth factor beta receptor 1 | 0.000944 |
| ENSG00000219607 | PPP1R3G | Protein phosphatase 1 regulatory subunit 3G | 0.000946 |
| ENSG00000164687 | FABP5 | Fatty acid binding protein 5 | 0.00098 |
| ENSG00000144824 | PHLDB2 | Pleckstrin homology like domain family B member 2 | 0.000988 |
| ENSG00000168405 | CMAHP | Cytidine monophospho-N-acetylneuraminic acid hydroxylase, pseudogene | 0.00103 |
| ENSG00000134987 | WDR36 | WD repeat domain 36 | 0.001108 |

| | | | |
|---|---|---|---|
| ENSG00000197646 | PDCD1LG2 | Programmed cell death 1 ligand 2 | 0.001122 |
| ENSG00000183496 | MEX3B | Mex-3 RNA binding family member B | 0.001154 |
| ENSG00000276966 | H4C5 | H4 clustered histone 5 | 0.001243 |
| ENSG00000168542 | COL3A1 | Collagen type III alpha 1 chain | 0.001294 |
| ENSG00000251493 | FOXD1 | Forkhead box D1 | 0.0013 |
| ENSG00000113657 | DPYSL3 | Dihydropyrimidinase like 3 | 0.00132 |
| ENSG00000135318 | NT5E | 5'-nucleotidase ecto | 0.001363 |
| ENSG00000247626 | MARS2 | Methionyl-trna synthetase 2, mitochondrial | 0.001384 |
| ENSG00000137054 | POLR1E | RNA polymerase I subunit E | 0.001412 |
| ENSG00000181201 | H2BU2P | H2B.U histone 2, pseudogene | 0.001435 |
| ENSG00000135312 | HTR1B | 5-hydroxytryptamine receptor 1B | 0.001451 |
| ENSG00000085276 | MECOM | MDS1 and EVI1 complex locus | 0.00147 |
| ENSG00000113578 | FGF1 | Fibroblast growth factor 1 | 0.001568 |
| ENSG00000087116 | ADAMTS2 | ADAM metallopeptidase with thrombospondin type 1 motif 2 | 0.001648 |
| ENSG00000137819 | PAQR5 | Progestin and adipoq receptor family member 5 | 0.001648 |
| ENSG00000178860 | MSC | Musculin | 0.001696 |
| ENSG00000136933 | RABEPK | Rab9 effector protein with kelch motifs | 0.001798 |
| ENSG00000165175 | MID1IP1 | MID1 interacting protein 1 | 0.001861 |
| ENSG00000138356 | AOX1 | Aldehyde oxidase 1 | 0.001898 |
| ENSG00000091136 | LAMB1 | Laminin subunit beta 1 | 0.001906 |
| ENSG00000180611 | MB21D2 | Mab-21 domain containing 2 | 0.001936 |
| ENSG00000196352 | CD55 | CD55 molecule (Cromer blood group) | 0.001936 |
| ENSG00000177191 | B3GNT8 | UDP-glcnac:betagal beta-1,3-N-acetylglucosaminyltransferase 8 | 0.001944 |
| ENSG00000102452 | NALCN | Sodium leak channel, non-selective | 0.001944 |
| ENSG00000278637 | H4C1 | H4 clustered histone 1 | 0.00195 |
| ENSG00000173457 | PPP1R14B | Protein phosphatase 1 regulatory inhibitor subunit 14B | 0.00195 |
| ENSG00000196890 | H2BU1 | H2B.U histone 1 | 0.002024 |
| ENSG00000273703 | H2BC14 | H2B clustered histone 14 | 0.002187 |
| ENSG00000065150 | IPO5 | Importin 5 | 0.002231 |
| ENSG00000116132 | PRRX1 | Paired related homeobox 1 | 0.002263 |
| ENSG00000039139 | DNAH5 | Dynein axonemal heavy chain 5 | 0.002444 |
| ENSG00000039560 | RAI14 | Retinoic acid induced 14 | 0.002464 |
| ENSG00000079156 | OSBPL6 | Oxysterol binding protein like 6 | 0.002528 |
| ENSG00000141668 | CBLN2 | Cerebellin 2 precursor | 0.002552 |
| ENSG00000158716 | DUSP23 | Dual specificity phosphatase 23 | 0.002639 |
| ENSG00000158483 | FAM86C1 | Family with sequence similarity 86 member C1 | 0.002657 |
| ENSG00000144867 | SRPRB | SRP receptor subunit beta | 0.00275 |
| ENSG00000186340 | THBS2 | Thrombospondin 2 | 0.002815 |
| ENSG00000112773 | TENT5A | Terminal nucleotidyltransferase 5A | 0.002853 |
| ENSG00000135245 | HILPDA | Hypoxia inducible lipid droplet associated | 0.002957 |
| ENSG00000198081 | ZBTB14 | Zinc finger and BTB domain containing 14 | 0.003085 |
| ENSG00000138074 | SLC5A6 | Solute carrier family 5 member 6 | 0.003124 |
| ENSG00000169432 | SCN9A | Sodium voltage-gated channel alpha subunit 9 | 0.003312 |
| ENSG00000156471 | PTDSS1 | Phosphatidylserine synthase 1 | 0.00336 |
| ENSG00000171492 | LRRC8D | Leucine rich repeat containing 8 VRAC subunit D | 0.00348 |
| ENSG00000153885 | KCTD15 | Potassium channel tetramerization domain containing 15 | 0.003522 |
| ENSG00000144749 | LRIG1 | Leucine rich repeats and immunoglobulin like domains 1 | 0.00357 |
| ENSG00000137801 | THBS1 | Thrombospondin 1 | 0.003584 |
| ENSG00000019186 | CYP24A1 | Cytochrome P450 family 24 subfamily A member 1 | 0.003591 |

| | | | |
|---|---|---|---|
| ENSG00000176171 | BNIP3 | BCL2 interacting protein 3 | 0.00361 |
| ENSG00000139329 | LUM | Lumican | 0.00362 |
| ENSG00000151892 | GFRA1 | GDNF family receptor alpha 1 | 0.003685 |
| ENSG00000120256 | LRP11 | LDL receptor related protein 11 | 0.003702 |
| ENSG00000159399 | HK2 | Hexokinase 2 | 0.003751 |
| ENSG00000139263 | LRIG3 | Leucine rich repeats and immunoglobulin like domains 3 | 0.003752 |
| ENSG00000111371 | SLC38A1 | Solute carrier family 38 member 1 | 0.003774 |
| ENSG00000162433 | AK4 | Adenylate kinase 4 | 0.003837 |
| ENSG00000135446 | CDK4 | Cyclin dependent kinase 4 | 0.003837 |
| ENSG00000168502 | MTCL1 | Microtubule crosslinking factor 1 | 0.003917 |
| ENSG00000130299 | GTPBP3 | GTP binding protein 3, mitochondrial | 0.003955 |
| ENSG00000099250 | NRP1 | Neuropilin 1 | 0.004047 |
| ENSG00000102743 | SLC25A15 | Solute carrier family 25 member 15 | 0.004106 |
| ENSG00000144642 | RBMS3 | RNA binding motif single stranded interacting protein 3 | 0.004204 |
| ENSG00000117152 | RGS4 | Regulator of G protein signaling 4 | 0.004258 |
| ENSG00000164284 | GRPEL2 | Grpe like 2, mitochondrial | 0.004259 |
| ENSG00000184675 | AMER1 | APC membrane recruitment protein 1 | 0.004295 |
| ENSG00000181982 | CCDC149 | Coiled-coil domain containing 149 | 0.004328 |
| ENSG00000100234 | TIMP3 | TIMP metallopeptidase inhibitor 3 | 0.004328 |
| ENSG00000147883 | CDKN2B | Cyclin dependent kinase inhibitor 2B | 0.004328 |
| ENSG00000075142 | SRI | Sorcin | 0.004328 |
| ENSG00000105855 | ITGB8 | Integrin subunit beta 8 | 0.004477 |
| ENSG00000125863 | MKKS | Mckusick-Kaufman syndrome | 0.004498 |
| ENSG00000165030 | NFIL3 | Nuclear factor, interleukin 3 regulated | 0.00456 |
| ENSG00000198121 | LPAR1 | Lysophosphatidic acid receptor 1 | 0.00459 |
| ENSG00000174827 | PDZK1 | PDZ domain containing 1 | 0.004596 |
| ENSG00000174136 | RGMB | Repulsive guidance molecule BMP co-receptor b | 0.004608 |
| ENSG00000176055 | MBLAC2 | Metallo-beta-lactamase domain containing 2 | 0.004633 |
| ENSG00000239264 | TXNDC5 | Thioredoxin domain containing 5 | 0.004707 |
| ENSG00000145242 | EPHA5 | EPH receptor A5 | 0.004763 |
| ENSG00000203805 | PLPP4 | Phospholipid phosphatase 4 | 0.004798 |
| ENSG00000123096 | SSPN | Sarcospan | 0.004887 |
| ENSG00000113721 | PDGFRB | Platelet derived growth factor receptor beta | 0.005066 |
| ENSG00000141858 | SAMD1 | Sterile alpha motif domain containing 1 | 0.005111 |
| ENSG00000186523 | FAM86B1 | Family with sequence similarity 86 member B1 | 0.005182 |
| ENSG00000148848 | ADAM12 | ADAM metallopeptidase domain 12 | 0.005341 |
| ENSG00000154978 | VOPP1 | VOPP1 WW domain binding protein | 0.005411 |
| ENSG00000160193 | WDR4 | WD repeat domain 4 | 0.005522 |
| ENSG00000242114 | MTFP1 | Mitochondrial fission process 1 | 0.005545 |
| ENSG00000155858 | LSM11 | LSM11, U7 small nuclear RNA associated | 0.005565 |
| ENSG00000150938 | CRIM1 | Cysteine rich transmembrane BMP regulator 1 | 0.005736 |
| ENSG00000196517 | SLC6A9 | Solute carrier family 6 member 9 | 0.005763 |
| ENSG00000103196 | CRISPLD2 | Cysteine rich secretory protein LCCL domain containing 2 | 0.005843 |
| ENSG00000115657 | ABCB6 | ATP binding cassette subfamily B member 6 (Langereis blood group) | 0.006087 |
| ENSG00000244026 | FAM86DP | Family with sequence similarity 86 member D, pseudogene | 0.006251 |
| ENSG00000185070 | FLRT2 | Fibronectin leucine rich transmembrane protein 2 | 0.006328 |
| ENSG00000145990 | GFOD1 | Glucose-fructose oxidoreductase domain containing 1 | 0.006334 |
| ENSG00000180448 | AC004151.1 | Rho gtpase activating protein 45 | 0.006347 |

| | | | |
|---|---|---|---|
| ENSG00000186352 | ANKRD37 | Ankyrin repeat domain 37 | 0.006347 |
| ENSG00000164107 | HAND2 | Heart and neural crest derivatives expressed 2 | 0.006383 |
| ENSG00000112902 | SEMA5A | Semaphorin 5A | 0.006533 |
| ENSG00000021645 | NRXN3 | Neurexin 3 | 0.006619 |
| ENSG00000241697 | TMEFF1 | Transmembrane protein with EGF like and two follistatin like domains 1 | 0.00664 |
| ENSG00000181588 | MEX3D | Mex-3 RNA binding family member D | 0.006658 |
| ENSG00000184009 | ACTG1 | Actin gamma 1 | 0.00675 |
| ENSG00000167778 | SPRYD3 | SPRY domain containing 3 | 0.006961 |
| ENSG00000073536 | NLE1 | Notchless homolog 1 | 0.007115 |
| ENSG00000203668 | CHML | CHM like Rab escort protein | 0.007195 |
| ENSG00000112365 | ZBTB24 | Zinc finger and BTB domain containing 24 | 0.0073 |
| ENSG00000103522 | IL21R | Interleukin 21 receptor | 0.007366 |
| ENSG00000183023 | SLC8A1 | Solute carrier family 8 member A1 | 0.007393 |
| ENSG00000150556 | LYPD6B | LY6/PLAUR domain containing 6B | 0.007412 |
| ENSG00000182173 | TSEN54 | Trna splicing endonuclease subunit 54 | 0.007471 |
| ENSG00000123843 | C4BPB | Complement component 4 binding protein beta | 0.007475 |
| ENSG00000167460 | TPM4 | Tropomyosin 4 | 0.007588 |
| ENSG00000117519 | CNN3 | Calponin 3 | 0.007662 |
| ENSG00000148680 | HTR7 | 5-hydroxytryptamine receptor 7 | 0.007768 |
| ENSG00000074410 | CA12 | Carbonic anhydrase 12 | 0.007768 |
| ENSG00000070961 | ATP2B1 | Atpase plasma membrane Ca2+ transporting 1 | 0.007768 |
| ENSG00000106484 | MEST | Mesoderm specific transcript | 0.007815 |
| ENSG00000132661 | NXT1 | Nuclear transport factor 2 like export factor 1 | 0.007839 |
| ENSG00000049323 | LTBP1 | Latent transforming growth factor beta binding protein 1 | 0.007859 |
| ENSG00000137547 | MRPL15 | Mitochondrial ribosomal protein L15 | 0.008181 |
| ENSG00000166741 | NNMT | Nicotinamide N-methyltransferase | 0.008199 |
| ENSG00000146281 | PM20D2 | Peptidase M20 domain containing 2 | 0.008298 |
| ENSG00000134668 | SPOCD1 | SPOC domain containing 1 | 0.008329 |
| ENSG00000125630 | POLR1B | RNA polymerase I subunit B | 0.008495 |
| ENSG00000128973 | CLN6 | CLN6 transmembrane ER protein | 0.008566 |
| ENSG00000038295 | TLL1 | Tolloid like 1 | 0.00859 |
| ENSG00000106772 | PRUNE2 | Prune homolog 2 with BCH domain | 0.008606 |
| ENSG00000174206 | C12orf66 | Chromosome 12 open reading frame 66 | 0.00866 |
| ENSG00000171791 | BCL2 | BCL2 apoptosis regulator | 0.008669 |
| ENSG00000101255 | TRIB3 | Tribbles pseudokinase 3 | 0.009087 |
| ENSG00000133816 | MICAL2 | Microtubule associated monooxygenase, calponin and LIM domain containing 2 | 0.009167 |
| ENSG00000187742 | SECISBP2 | SECIS binding protein 2 | 0.009381 |
| ENSG00000112658 | SRF | Serum response factor | 0.009519 |
| ENSG00000125247 | TMTC4 | Transmembrane O-mannosyltransferase targeting cadherins 4 | 0.009804 |
| ENSG00000119938 | PPP1R3C | Protein phosphatase 1 regulatory subunit 3C | 0.009804 |
| ENSG00000188290 | HES4 | Hes family bhlh transcription factor 4 | 0.009834 |
| ENSG00000166289 | PLEKHF1 | Pleckstrin homology and FYVE domain containing 1 | 0.010088 |
| ENSG00000137273 | FOXF2 | Forkhead box F2 | 0.010511 |
| ENSG00000147485 | PXDNL | Peroxidasin like | 0.010573 |
| ENSG00000139370 | SLC15A4 | Solute carrier family 15 member 4 | 0.010678 |
| ENSG00000171488 | LRRC8C | Leucine rich repeat containing 8 VRAC subunit C | 0.010744 |
| ENSG00000261236 | BOP1 | BOP1 ribosomal biogenesis factor | 0.011134 |
| ENSG00000138944 | SHISAL1 | Shisa like 1 | 0.011167 |

| | | | |
|---|---|---|---|
| ENSG00000131019 | ULBP3 | UL16 binding protein 3 | 0.011403 |
| ENSG00000163751 | CPA3 | Carboxypeptidase A3 | 0.011513 |
| ENSG00000042832 | TG | Thyroglobulin | 0.011686 |
| ENSG00000072310 | SREBF1 | Sterol regulatory element binding transcription factor 1 | 0.011686 |
| ENSG00000099725 | PRKY | Protein kinase Y-linked (pseudogene) | 0.011876 |
| ENSG00000130810 | PPAN | Peter pan homolog | 0.011955 |
| ENSG00000205542 | TMSB4X | Thymosin beta 4 X-linked | 0.012568 |
| ENSG00000204856 | FAM216A | Family with sequence similarity 216 member A | 0.012575 |
| ENSG00000107104 | KANK1 | KN motif and ankyrin repeat domains 1 | 0.012714 |
| ENSG00000125430 | HS3ST3B1 | Heparan sulfate-glucosamine 3-sulfotransferase 3B1 | 0.012714 |
| ENSG00000145632 | PLK2 | Polo like kinase 2 | 0.012735 |
| ENSG00000117479 | SLC19A2 | Solute carrier family 19 member 2 | 0.012735 |
| ENSG00000112893 | MAN2A1 | Mannosidase alpha class 2A member 1 | 0.012751 |
| ENSG00000134294 | SLC38A2 | Solute carrier family 38 member 2 | 0.012751 |
| ENSG00000049192 | ADAMTS6 | ADAM metallopeptidase with thrombospondin type 1 motif 6 | 0.013066 |
| ENSG00000180801 | ARSJ | Arylsulfatase family member J | 0.013066 |
| ENSG00000118894 | EEF2KMT | Eukaryotic elongation factor 2 lysine methyltransferase | 0.013108 |
| ENSG00000161547 | SRSF2 | Serine and arginine rich splicing factor 2 | 0.013136 |
| ENSG00000161091 | MFSD12 | Major facilitator superfamily domain containing 12 | 0.0133 |
| ENSG00000080493 | SLC4A4 | Solute carrier family 4 member 4 | 0.013348 |
| ENSG00000180198 | RCC1 | Regulator of chromosome condensation 1 | 0.013365 |
| ENSG00000105971 | CAV2 | Caveolin 2 | 0.013365 |
| ENSG00000154127 | UBASH3B | Ubiquitin associated and SH3 domain containing B | 0.013533 |
| ENSG00000177192 | PUS1 | Pseudouridine synthase 1 | 0.013606 |
| ENSG00000102967 | DHODH | Dihydroorotate dehydrogenase (quinone) | 0.013606 |
| ENSG00000077092 | RARB | Retinoic acid receptor beta | 0.013752 |
| ENSG00000277775 | H3C7 | H3 clustered histone 7 | 0.013771 |
| ENSG00000176749 | CDK5R1 | Cyclin dependent kinase 5 regulatory subunit 1 | 0.013844 |
| ENSG00000104907 | TRMT1 | Trna methyltransferase 1 | 0.013844 |
| ENSG00000153976 | HS3ST3A1 | Heparan sulfate-glucosamine 3-sulfotransferase 3A1 | 0.013874 |
| ENSG00000111450 | STX2 | Syntaxin 2 | 0.014621 |
| ENSG00000185238 | PRMT3 | Protein arginine methyltransferase 3 | 0.014754 |
| ENSG00000254093 | PINX1 | PIN2 (TERF1) interacting telomerase inhibitor 1 | 0.014819 |
| ENSG00000148229 | POLE3 | DNA polymerase epsilon 3, accessory subunit | 0.01538 |
| ENSG00000171208 | NETO2 | Neuropilin and tolloid like 2 | 0.015481 |
| ENSG00000213853 | EMP2 | Epithelial membrane protein 2 | 0.015481 |
| ENSG00000138448 | ITGAV | Integrin subunit alpha V | 0.015481 |
| ENSG00000160208 | RRP1B | Ribosomal RNA processing 1B | 0.015481 |
| ENSG00000124787 | RPP40 | Ribonuclease P/MRP subunit p40 | 0.0155 |
| ENSG00000184465 | WDR27 | WD repeat domain 27 | 0.0155 |
| ENSG00000125266 | EFNB2 | Ephrin B2 | 0.0155 |
| ENSG00000244306 | AL589743.1 | Double homeobox A pseudogene 10 | 0.0155 |
| ENSG00000157193 | LRP8 | LDL receptor related protein 8 | 0.015728 |
| ENSG00000165732 | DDX21 | Dexd-box helicase 21 | 0.015728 |
| ENSG00000154553 | PDLIM3 | PDZ and LIM domain 3 | 0.015742 |
| ENSG00000186654 | PRR5 | Proline rich 5 | 0.015905 |
| ENSG00000085415 | SEH1L | SEH1 like nucleoporin | 0.015905 |
| ENSG00000158125 | XDH | Xanthine dehydrogenase | 0.015948 |
| ENSG00000075223 | SEMA3C | Semaphorin 3C | 0.015948 |
| ENSG00000122861 | PLAU | Plasminogen activator, urokinase | 0.016158 |

| | | | |
|---|---|---|---|
| ENSG00000128294 | TPST2 | Tyrosylprotein sulfotransferase 2 | 0.016256 |
| ENSG00000136999 | CCN3 | Cellular communication network factor 3 | 0.016269 |
| ENSG00000118508 | RAB32 | RAB32, member RAS oncogene family | 0.016269 |
| ENSG00000117411 | B4GALT2 | Beta-1,4-galactosyltransferase 2 | 0.01643 |
| ENSG00000120942 | UBIAD1 | Ubia prenyltransferase domain containing 1 | 0.016683 |
| ENSG00000116667 | C1orf21 | Chromosome 1 open reading frame 21 | 0.017091 |
| ENSG00000234602 | MCIDAS | Multiciliate differentiation and DNA synthesis associated cell cycle protein | 0.017213 |
| ENSG00000168785 | TSPAN5 | Tetraspanin 5 | 0.017365 |
| ENSG00000093217 | XYLB | Xylulokinase | 0.017815 |
| ENSG00000128272 | ATF4 | Activating transcription factor 4 | 0.017815 |
| ENSG00000138741 | TRPC3 | Transient receptor potential cation channel subfamily C member 3 | 0.017853 |
| ENSG00000136720 | HS6ST1 | Heparan sulfate 6-O-sulfotransferase 1 | 0.01787 |
| ENSG00000131652 | THOC6 | THO complex 6 | 0.017996 |
| ENSG00000124784 | RIOK1 | RIO kinase 1 | 0.017996 |
| ENSG00000145287 | PLAC8 | Placenta associated 8 | 0.018116 |
| ENSG00000155846 | PPARGC1B | PPARG coactivator 1 beta | 0.018192 |
| ENSG00000183605 | SFXN4 | Sideroflexin 4 | 0.018192 |
| ENSG00000100029 | PES1 | Pescadillo ribosomal biogenesis factor 1 | 0.018192 |
| ENSG00000100314 | CABP7 | Calcium binding protein 7 | 0.018223 |
| ENSG00000239672 | NME1 | NME/NM23 nucleoside diphosphate kinase 1 | 0.018223 |
| ENSG00000048162 | NOP16 | NOP16 nucleolar protein | 0.018301 |
| ENSG00000127329 | PTPRB | Protein tyrosine phosphatase receptor type B | 0.018322 |
| ENSG00000284620 | AF228730.5 | RNA binding protein, fox-1 homolog (C. Elegans) 1 (RBFOX1) pseudogene | 0.018561 |
| ENSG00000145545 | SRD5A1 | Steroid 5 alpha-reductase 1 | 0.018586 |
| ENSG00000050405 | LIMA1 | LIM domain and actin binding 1 | 0.018652 |
| ENSG00000082482 | KCNK2 | Potassium two pore domain channel subfamily K member 2 | 0.018805 |
| ENSG00000114686 | MRPL3 | Mitochondrial ribosomal protein L3 | 0.019114 |
| ENSG00000186847 | KRT14 | Keratin 14 | 0.019236 |
| ENSG00000165359 | INTS6L | Integrator complex subunit 6 like | 0.019344 |
| ENSG00000055813 | CCDC85A | Coiled-coil domain containing 85A | 0.019794 |
| ENSG00000106348 | IMPDH1 | Inosine monophosphate dehydrogenase 1 | 0.020452 |
| ENSG00000086848 | ALG9 | ALG9 alpha-1,2-mannosyltransferase | 0.020551 |
| ENSG00000169926 | KLF13 | Kruppel like factor 13 | 0.020703 |
| ENSG00000205426 | KRT81 | Keratin 81 | 0.020959 |
| ENSG00000162711 | NLRP3 | NLR family pyrin domain containing 3 | 0.020959 |
| ENSG00000183527 | PSMG1 | Proteasome assembly chaperone 1 | 0.020959 |
| ENSG00000118263 | KLF7 | Kruppel like factor 7 | 0.021223 |
| ENSG00000214706 | IFRD2 | Interferon related developmental regulator 2 | 0.021859 |
| ENSG00000150990 | DHX37 | DEAH-box helicase 37 | 0.022255 |
| ENSG00000074582 | BCS1L | BCS1 homolog, ubiquinol-cytochrome c reductase complex chaperone | 0.022255 |
| ENSG00000111641 | NOP2 | NOP2 nucleolar protein | 0.022255 |
| ENSG00000038427 | VCAN | Versican | 0.022425 |
| ENSG00000227120 | AC009238.1 | Pseudogene similar to part of Cdon homolog (mouse) (CDON) | 0.023124 |
| ENSG00000109576 | AADAT | Aminoadipate aminotransferase | 0.023985 |
| ENSG00000198478 | SH3BGRL2 | SH3 domain binding glutamate rich protein like 2 | 0.024079 |

| | | | |
|---|---|---|---|
| ENSG00000071242 | RPS6KA2 | Ribosomal protein S6 kinase A2 | 0.024148 |
| ENSG00000185304 | RGPD2 | RANBP2 like and GRIP domain containing 2 | 0.024677 |
| ENSG00000077348 | EXOSC5 | Exosome component 5 | 0.02474 |
| ENSG00000106546 | AHR | Aryl hydrocarbon receptor | 0.02474 |
| ENSG00000147955 | SIGMAR1 | Sigma non-opioid intracellular receptor 1 | 0.02491 |
| ENSG00000278540 | ACACA | Acetyl-coa carboxylase alpha | 0.024952 |
| ENSG00000175183 | CSRP2 | Cysteine and glycine rich protein 2 | 0.025037 |
| ENSG00000094804 | CDC6 | Cell division cycle 6 | 0.025037 |
| ENSG00000171490 | RSL1D1 | Ribosomal L1 domain containing 1 | 0.025131 |
| ENSG00000171067 | C11orf24 | Chromosome 11 open reading frame 24 | 0.025197 |
| ENSG00000116991 | SIPA1L2 | Signal induced proliferation associated 1 like 2 | 0.025231 |
| ENSG00000106263 | EIF3B | Eukaryotic translation initiation factor 3 subunit B | 0.025231 |
| ENSG00000111666 | CHPT1 | Choline phosphotransferase 1 | 0.025327 |
| ENSG00000126785 | RHOJ | Ras homolog family member J | 0.025399 |
| ENSG00000157680 | DGKI | Diacylglycerol kinase iota | 0.025399 |
| ENSG00000134490 | TMEM241 | Transmembrane protein 241 | 0.025878 |
| ENSG00000198142 | SOWAHC | Sosondowah ankyrin repeat domain family member C | 0.025919 |
| ENSG00000168209 | DDIT4 | DNA damage inducible transcript 4 | 0.025984 |
| ENSG00000168496 | FEN1 | Flap structure-specific endonuclease 1 | 0.025984 |
| ENSG00000122042 | UBL3 | Ubiquitin like 3 | 0.026224 |
| ENSG00000071564 | TCF3 | Transcription factor 3 | 0.026266 |
| ENSG00000069122 | ADGRF5 | Adhesion G protein-coupled receptor F5 | 0.026343 |
| ENSG00000151835 | SACS | Sacsin molecular chaperone | 0.026379 |
| ENSG00000244274 | DBNDD2 | Dysbindin domain containing 2 | 0.026379 |
| ENSG00000138772 | ANXA3 | Annexin A3 | 0.026554 |
| ENSG00000128951 | DUT | Deoxyuridine triphosphatase | 0.02713 |
| ENSG00000186141 | POLR3C | RNA polymerase III subunit C | 0.027285 |
| ENSG00000251669 | FAM86EP | Family with sequence similarity 86 member E, pseudogene | 0.027302 |
| ENSG00000148926 | ADM | Adrenomedullin | 0.028611 |
| ENSG00000155438 | NIFK | Nucleolar protein interacting with the FHA domain of MKI67 | 0.028809 |
| ENSG00000102178 | UBL4A | Ubiquitin like 4A | 0.028971 |
| ENSG00000105185 | PDCD5 | Programmed cell death 5 | 0.029192 |
| ENSG00000270276 | H4C15 | H4 clustered histone 15 | 0.029418 |
| ENSG00000157827 | FMNL2 | Formin like 2 | 0.029504 |
| ENSG00000111799 | COL12A1 | Collagen type XII alpha 1 chain | 0.029767 |
| ENSG00000149591 | TAGLN | Transgelin | 0.030118 |
| ENSG00000185640 | KRT79 | Keratin 79 | 0.030242 |
| ENSG00000133597 | ADCK2 | Aarf domain containing kinase 2 | 0.030507 |
| ENSG00000069998 | HDHD5 | Haloacid dehalogenase like hydrolase domain containing 5 | 0.030535 |
| ENSG00000119599 | DCAF4 | DDB1 and CUL4 associated factor 4 | 0.030564 |
| ENSG00000188549 | CCDC9B | Coiled-coil domain containing 9B | 0.030614 |
| ENSG00000140937 | CDH11 | Cadherin 11 | 0.030614 |
| ENSG00000122644 | ARL4A | ADP ribosylation factor like gtpase 4A | 0.030614 |
| ENSG00000284753 | EEF1AKMT4 | EEF1A lysine methyltransferase 4 | 0.030729 |
| ENSG00000066583 | ISOC1 | Isochorismatase domain containing 1 | 0.030878 |
| ENSG00000131378 | RFTN1 | Raftlin, lipid raft linker 1 | 0.030923 |
| ENSG00000288031 | AC093838.2 | Novel pseudogene | 0.031492 |
| ENSG00000159199 | ATP5MC1 | ATP synthase membrane subunit c locus 1 | 0.031728 |

| | | | |
|---|---|---|---|
| ENSG00000143476 | DTL | Denticleless E3 ubiquitin protein ligase homolog | 0.031757 |
| ENSG00000186918 | ZNF395 | Zinc finger protein 395 | 0.031907 |
| ENSG00000154122 | ANKH | ANKH inorganic pyrophosphate transport regulator | 0.031907 |
| ENSG00000256667 | KLRA1P | Killer cell lectin like receptor A1, pseudogene | 0.032072 |
| ENSG00000085840 | ORC1 | Origin recognition complex subunit 1 | 0.032072 |
| ENSG00000124766 | SOX4 | SRY-box transcription factor 4 | 0.032844 |
| ENSG00000160214 | RRP1 | Ribosomal RNA processing 1 | 0.032855 |
| ENSG00000065485 | PDIA5 | Protein disulfide isomerase family A member 5 | 0.033075 |
| ENSG00000065183 | WDR3 | WD repeat domain 3 | 0.033075 |
| ENSG00000134917 | ADAMTS8 | ADAM metallopeptidase with thrombospondin type 1 motif 8 | 0.033107 |
| ENSG00000011677 | GABRA3 | Gamma-aminobutyric acid type A receptor alpha3 subunit | 0.033107 |
| ENSG00000165724 | ZMYND19 | Zinc finger MYND-type containing 19 | 0.033107 |
| ENSG00000156697 | UTP14A | UTP14A small subunit processome component | 0.033107 |
| ENSG00000154319 | FAM167A | Family with sequence similarity 167 member A | 0.033469 |
| ENSG00000138347 | MYPN | Myopalladin | 0.033855 |
| ENSG00000213585 | VDAC1 | Voltage dependent anion channel 1 | 0.033855 |
| ENSG00000177425 | PAWR | Pro-apoptotic WT1 regulator | 0.033855 |
| ENSG00000103888 | CEMIP | Cell migration inducing hyaluronidase 1 | 0.034194 |
| ENSG00000106511 | MEOX2 | Mesenchyme homeobox 2 | 0.034267 |
| ENSG00000205221 | VIT | Vitrin | 0.034281 |
| ENSG00000170802 | FOXN2 | Forkhead box N2 | 0.034281 |
| ENSG00000268089 | GABRQ | Gamma-aminobutyric acid type A receptor theta subunit | 0.034835 |
| ENSG00000154736 | ADAMTS5 | ADAM metallopeptidase with thrombospondin type 1 motif 5 | 0.035587 |
| ENSG00000114850 | SSR3 | Signal sequence receptor subunit 3 | 0.036362 |
| ENSG00000136877 | FPGS | Folylpolyglutamate synthase | 0.036362 |
| ENSG00000122641 | INHBA | Inhibin subunit beta A | 0.036563 |
| ENSG00000100664 | EIF5 | Eukaryotic translation initiation factor 5 | 0.036563 |
| ENSG00000041982 | TNC | Tenascin C | 0.036947 |
| ENSG00000132359 | AC002316.1 | RAP1 gtpase activating protein 2 | 0.037175 |
| ENSG00000148120 | AOPEP | Aminopeptidase O (putative) | 0.037191 |
| ENSG00000109452 | INPP4B | Inositol polyphosphate-4-phosphatase type II B | 0.037506 |
| ENSG00000114251 | WNT5A | Wnt family member 5A | 0.037551 |
| ENSG00000136813 | ECPAS | Ecm29 proteasome adaptor and scaffold | 0.037611 |
| ENSG00000144233 | AMMECR1L | AMMECR1 like | 0.037686 |
| ENSG00000220201 | ZGLP1 | Zinc finger GATA like protein 1 | 0.037722 |
| ENSG00000145685 | LHFPL2 | LHFPL tetraspan subfamily member 2 | 0.038198 |
| ENSG00000105976 | MET | MET proto-oncogene, receptor tyrosine kinase | 0.038662 |
| ENSG00000165617 | DACT1 | Dishevelled binding antagonist of beta catenin 1 | 0.039097 |
| ENSG00000264230 | ANXA8L1 | Annexin A8 like 1 | 0.039791 |
| ENSG00000187123 | LYPD6 | LY6/PLAUR domain containing 6 | 0.040303 |
| ENSG00000136379 | ABHD17C | Abhydrolase domain containing 17C | 0.040533 |
| ENSG00000130826 | DKC1 | Dyskerin pseudouridine synthase 1 | 0.040533 |
| ENSG00000130204 | TOMM40 | Translocase of outer mitochondrial membrane 40 | 0.040533 |
| ENSG00000198912 | C1orf174 | Chromosome 1 open reading frame 174 | 0.040542 |
| ENSG00000183098 | GPC6 | Glypican 6 | 0.040569 |
| ENSG00000114423 | CBLB | Cbl proto-oncogene B | 0.040686 |
| ENSG00000175305 | CCNE2 | Cyclin E2 | 0.040735 |
| ENSG00000004660 | CAMKK1 | Calcium/calmodulin dependent protein kinase kinase 1 | 0.04092 |

| | | | |
|---|---|---|---|
| ENSG00000079150 | FKBP7 | FKBP prolyl isomerase 7 | 0.040951 |
| ENSG00000138758 | SEPTIN11 | Septin 11 | 0.041077 |
| ENSG00000162746 | FCRLB | Fc receptor like B | 0.041766 |
| ENSG00000124370 | MCEE | Methylmalonyl-coa epimerase | 0.042041 |
| ENSG00000115414 | FN1 | Fibronectin 1 | 0.042709 |
| ENSG00000116761 | CTH | Cystathionine gamma-lyase | 0.042711 |
| ENSG00000187957 | DNER | Delta/notch like EGF repeat containing | 0.042711 |
| ENSG00000117143 | UAP1 | UDP-N-acetylglucosamine pyrophosphorylase 1 | 0.042711 |
| ENSG00000114450 | GNB4 | G protein subunit beta 4 | 0.043143 |
| ENSG00000181788 | SIAH2 | Siah E3 ubiquitin protein ligase 2 | 0.043916 |
| ENSG00000173926 | MARCHF3 | Membrane associated ring-CH-type finger 3 | 0.044693 |
| ENSG00000171084 | FAM86JP | Family with sequence similarity 86 member J, pseudogene | 0.044974 |
| ENSG00000173638 | SLC19A1 | Solute carrier family 19 member 1 | 0.045964 |
| ENSG00000173898 | SPTBN2 | Spectrin beta, non-erythrocytic 2 | 0.046283 |
| ENSG00000113460 | BRIX1 | Biogenesis of ribosomes BRX1 | 0.046283 |
| ENSG00000166250 | CLMP | CXADR like membrane protein | 0.046309 |
| ENSG00000185875 | THNSL1 | Threonine synthase like 1 | 0.046309 |
| ENSG00000186104 | CYP2R1 | Cytochrome P450 family 2 subfamily R member 1 | 0.046329 |
| ENSG00000156502 | SUPV3L1 | Suv3 like RNA helicase | 0.046342 |
| ENSG00000011052 | NME1-NME2 | NME1-NME2 readthrough | 0.046348 |
| ENSG00000119900 | OGFRL1 | Opioid growth factor receptor like 1 | 0.047101 |
| ENSG00000164122 | ASB5 | Ankyrin repeat and SOCS box containing 5 | 0.047107 |
| ENSG00000176022 | B3GALT6 | Beta-1,3-galactosyltransferase 6 | 0.04733 |
| ENSG00000035687 | ADSS2 | Adenylosuccinate synthase 2 | 0.047481 |
| ENSG00000066468 | FGFR2 | Fibroblast growth factor receptor 2 | 0.047866 |
| ENSG00000273213 | H3-2 | H3.2 histone (putative) | 0.047866 |
| ENSG00000160752 | FDPS | Farnesyl diphosphate synthase | 0.047866 |
| ENSG00000119950 | MXI1 | MAX interactor 1, dimerization protein | 0.048368 |
| ENSG00000170468 | RIOX1 | Ribosomal oxygenase 1 | 0.048644 |
| ENSG00000197498 | RPF2 | Ribosome production factor 2 homolog | 0.049042 |
| ENSG00000105825 | TFPI2 | Tissue factor pathway inhibitor 2 | 0.049042 |
| ENSG00000121440 | PDZRN3 | PDZ domain containing ring finger 3 | 0.049401 |
| ENSG00000116106 | EPHA4 | EPH receptor A4 | 0.049401 |
| ENSG00000170456 | DENND5B | DENN domain containing 5B | 0.049401 |
| ENSG00000111364 | DDX55 | DEAD-box helicase 55 | 0.049401 |
| ENSG00000185730 | ZNF696 | Zinc finger protein 696 | 0.049457 |
| ENSG00000171793 | CTPS1 | CTP synthase 1 | 0.049457 |
| ENSG00000182985 | CADM1 | Cell adhesion molecule 1 | 0.049717 |
| ENSG00000168952 | STXBP6 | Syntaxin binding protein 6 | 0.049821 |

**Supplementary Table S4.** Statistically significant DEGs derived from a meta-analysis comparing DESeq2 outputs from irradiated and control samples of three datasets [PRJNA421022, PRJNA436999, PRJNA494581].

| Ensembl ID | Gene Symbol | Gene Description | Adj. *p*-Value |
|---|---|---|---|
| **Up-Regulated Genes↑** | | | |
| ENSG00000124762 | CDKN1A | Cyclin dependent kinase inhibitor 1A | 1.24E-108 |
| ENSG00000130513 | GDF15 | Growth differentiation factor 15 | 1.07E-38 |
| ENSG00000161513 | FDXR | Ferredoxin reductase | 7.72E-33 |
| ENSG00000164938 | TP53INP1 | Tumor protein p53 inducible nuclear protein 1 | 5.26E-29 |
| ENSG00000120889 | TNFRSF10B | TNF receptor superfamily member 10b | 1.18E-27 |
| ENSG00000163071 | SPATA18 | Spermatogenesis associated 18 | 1.45E-25 |
| ENSG00000055163 | CYFIP2 | Cytoplasmic FMR1 interacting protein 2 | 1.79E-24 |
| ENSG00000256664 | AC025423.2 | Ribosomal L24 domain containing 1 (RSL24D1) pseudogene | 5.27E-22 |
| ENSG00000135679 | MDM2 | MDM2 proto-oncogene | 1.49E-21 |
| ENSG00000109321 | AREG | Amphiregulin | 1.86E-21 |
| ENSG00000026103 | FAS | Fas cell surface death receptor | 1.97E-17 |
| ENSG00000105327 | BBC3 | BCL2 binding component 3 | 1.22E-15 |
| ENSG00000173535 | TNFRSF10C | TNF receptor superfamily member 10c | 5.43E-15 |
| ENSG00000159388 | BTG2 | BTG anti-proliferation factor 2 | 6.41E-15 |
| ENSG00000132563 | REEP2 | Receptor accessory protein 2 | 6.87E-15 |
| ENSG00000174307 | PHLDA3 | Pleckstrin homology like domain family A member 3 | 1.47E-14 |
| ENSG00000080546 | SESN1 | Sestrin 1 | 2.06E-13 |
| ENSG00000110900 | TSPAN11 | Tetraspanin 11 | 2.86E-13 |
| ENSG00000181026 | AEN | Apoptosis enhancing nuclease | 2.08E-12 |
| ENSG00000177076 | ACER2 | Alkaline ceramidase 2 | 1.54E-11 |
| ENSG00000134574 | DDB2 | Damage specific DNA binding protein 2 | 3.93E-11 |
| ENSG00000087088 | BAX | BCL2 associated X, apoptosis regulator | 4.17E-11 |
| ENSG00000244694 | PTCHD4 | Patched domain containing 4 | 4.57E-11 |
| ENSG00000143217 | NECTIN4 | Nectin cell adhesion molecule 4 | 7.59E-11 |
| ENSG00000177106 | EPS8L2 | EPS8 like 2 | 1.69E-10 |
| ENSG00000154767 | XPC | XPC complex subunit, DNA damage recognition and repair factor | 9.08E-10 |
| ENSG00000168918 | INPP5D | Inositol polyphosphate-5-phosphatase D | 1.43E-09 |
| ENSG00000130517 | PGPEP1 | Pyroglutamyl-peptidase I | 2.23E-09 |
| ENSG00000131080 | EDA2R | Ectodysplasin A2 receptor | 1.96E-08 |
| ENSG00000166592 | RRAD | RRAD, Ras related glycolysis inhibitor and calcium channel regulator | 2.30E-08 |
| ENSG00000172667 | ZMAT3 | Zinc finger matrin-type 3 | 3.68E-08 |
| ENSG00000162490 | DRAXIN | Dorsal inhibitory axon guidance protein | 3.69E-08 |
| ENSG00000162643 | WDR63 | WD repeat domain 63 | 4.68E-08 |
| ENSG00000141232 | TOB1 | Transducer of ERBB2, 1 | 4.96E-08 |
| ENSG00000177459 | ERICH5 | Glutamate rich 5 | 5.51E-08 |
| ENSG00000196562 | SULF2 | Sulfatase 2 | 6.51E-08 |
| ENSG00000104870 | FCGRT | Fc fragment of igg receptor and transporter | 6.70E-08 |
| ENSG00000135423 | GLS2 | Glutaminase 2 | 1.10E-07 |
| ENSG00000112249 | ASCC3 | Activating signal cointegrator 1 complex subunit 3 | 1.22E-07 |
| ENSG00000123933 | MXD4 | MAX dimerization protein 4 | 1.25E-07 |
| ENSG00000173530 | TNFRSF10D | TNF receptor superfamily member 10d | 1.78E-07 |
| ENSG00000167107 | ACSF2 | Acyl-coa synthetase family member 2 | 1.84E-07 |

| | | | |
|---|---|---|---|
| ENSG00000164463 | CREBRF | CREB3 regulatory factor | 9.14E-07 |
| ENSG00000068745 | IP6K2 | Inositol hexakisphosphate kinase 2 | 1.06E-06 |
| ENSG00000105650 | PDE4C | Phosphodiesterase 4C | 1.35E-06 |
| ENSG00000175938 | ORAI3 | ORAI calcium release-activated calcium modulator 3 | 2.07E-06 |
| ENSG00000137975 | CLCA2 | Chloride channel accessory 2 | 3.38E-06 |
| ENSG00000104689 | TNFRSF10A | TNF receptor superfamily member 10a | 4.22E-06 |
| ENSG00000173230 | GOLGB1 | Golgin B1 | 4.41E-06 |
| ENSG00000125746 | EML2 | EMAP like 2 | 4.83E-06 |
| ENSG00000139112 | GABARAPL1 | GABA type A receptor associated protein like 1 | 7.55E-06 |
| ENSG00000164237 | CMBL | Carboxymethylenebutenolidase homolog | 1.85E-05 |
| ENSG00000135452 | TSPAN31 | Tetraspanin 31 | 2.16E-05 |
| ENSG00000173846 | PLK3 | Polo like kinase 3 | 3.09E-05 |
| ENSG00000132824 | SERINC3 | Serine incorporator 3 | 3.59E-05 |
| ENSG00000185088 | RPS27L | Ribosomal protein S27 like | 3.88E-05 |
| ENSG00000204219 | TCEA3 | Transcription elongation factor A3 | 3.90E-05 |
| ENSG00000042445 | RETSAT | Retinol saturase | 3.90E-05 |
| ENSG00000001461 | NIPAL3 | NIPA like domain containing 3 | 4.99E-05 |
| ENSG00000196975 | ANXA4 | Annexin A4 | 5.76E-05 |
| ENSG00000198919 | DZIP3 | DAZ interacting zinc finger protein 3 | 7.37E-05 |
| ENSG00000130703 | OSBPL2 | Oxysterol binding protein like 2 | 7.75E-05 |
| ENSG00000196814 | MVB12B | Multivesicular body subunit 12B | 9.89E-05 |
| ENSG00000010030 | ETV7 | ETS variant transcription factor 7 | 0.000112 |
| ENSG00000158055 | GRHL3 | Grainyhead like transcription factor 3 | 0.000113 |
| ENSG00000105290 | APLP1 | Amyloid beta precursor like protein 1 | 0.00012 |
| ENSG00000132793 | LPIN3 | Lipin 3 | 0.000134 |
| ENSG00000138166 | DUSP5 | Dual specificity phosphatase 5 | 0.000151 |
| ENSG00000244509 | APOBEC3C | Apolipoprotein B mrna editing enzyme catalytic subunit 3C | 0.000165 |
| ENSG00000153029 | MR1 | Major histocompatibility complex, class I-related | 0.000166 |
| ENSG00000239713 | APOBEC3G | Apolipoprotein B mrna editing enzyme catalytic subunit 3G | 0.00017 |
| ENSG00000172831 | CES2 | Carboxylesterase 2 | 0.000172 |
| ENSG00000161896 | IP6K3 | Inositol hexakisphosphate kinase 3 | 0.000172 |
| ENSG00000119630 | PGF | Placental growth factor | 0.000179 |
| ENSG00000188554 | NBR1 | NBR1 autophagy cargo receptor | 0.000189 |
| ENSG00000117461 | PIK3R3 | Phosphoinositide-3-kinase regulatory subunit 3 | 0.00022 |
| ENSG00000148660 | CAMK2G | Calcium/calmodulin dependent protein kinase II gamma | 0.000221 |
| ENSG00000130477 | UNC13A | Unc-13 homolog A | 0.000226 |
| ENSG00000132274 | TRIM22 | Tripartite motif containing 22 | 0.00023 |
| ENSG00000161835 | GRASP | General receptor for phosphoinositides 1 associated scaffold protein | 0.000236 |
| ENSG00000169429 | CXCL8 | C-X-C motif chemokine ligand 8 | 0.000238 |
| ENSG00000173156 | RHOD | Ras homolog family member D | 0.000258 |
| ENSG00000047346 | FAM214A | Family with sequence similarity 214 member A | 0.000265 |
| ENSG00000153208 | MERTK | MER proto-oncogene, tyrosine kinase | 0.00028 |
| ENSG00000116299 | KIAA1324 | Kiaa1324 | 0.000295 |
| ENSG00000163346 | PBXIP1 | PBX homeobox interacting protein 1 | 0.000296 |
| ENSG00000149809 | TM7SF2 | Transmembrane 7 superfamily member 2 | 0.000299 |
| ENSG00000012822 | CALCOCO1 | Calcium binding and coiled-coil domain 1 | 0.000304 |
| ENSG00000169760 | NLGN1 | Neuroligin 1 | 0.000322 |
| ENSG00000167861 | HID1 | HID1 domain containing | 0.000352 |

| | | | |
|---|---|---|---|
| ENSG00000188002 | AC026412.1 | Programmed cell death 6 (PDCD6) pseudogene | 0.000444 |
| ENSG00000166887 | VPS39 | VPS39 subunit of HOPS complex | 0.000459 |
| ENSG00000136378 | ADAMTS7 | ADAM metallopeptidase with thrombospondin type 1 motif 7 | 0.000484 |
| ENSG00000060656 | PTPRU | Protein tyrosine phosphatase receptor type U | 0.00051 |
| ENSG00000175155 | YPEL2 | Yippee like 2 | 0.000521 |
| ENSG00000181513 | ACBD4 | Acyl-coa binding domain containing 4 | 0.000543 |
| ENSG00000130766 | SESN2 | Sestrin 2 | 0.000573 |
| ENSG00000197093 | GAL3ST4 | Galactose-3-O-sulfotransferase 4 | 0.0006 |
| ENSG00000131015 | ULBP2 | UL16 binding protein 2 | 0.000695 |
| ENSG00000137414 | FAM8A1 | Family with sequence similarity 8 member A1 | 0.000705 |
| ENSG00000138271 | GPR87 | G protein-coupled receptor 87 | 0.000705 |
| ENSG00000132906 | CASP9 | Caspase 9 | 0.000746 |
| ENSG00000189195 | BTBD8 | BTB domain containing 8 | 0.000798 |
| ENSG00000043143 | JADE2 | Jade family PHD finger 2 | 0.000828 |
| ENSG00000185215 | TNFAIP2 | TNF alpha induced protein 2 | 0.000839 |
| ENSG00000146411 | SLC2A12 | Solute carrier family 2 member 12 | 0.000899 |
| ENSG00000198753 | PLXNB3 | Plexin B3 | 0.000995 |
| ENSG00000232871 | SEC1P | Secretory blood group 1, pseudogene | 0.000995 |
| ENSG00000167196 | FBXO22 | F-box protein 22 | 0.000997 |
| ENSG00000176907 | TCIM | Transcriptional and immune response regulator | 0.001035 |
| ENSG00000135506 | OS9 | OS9 endoplasmic reticulum lectin | 0.001174 |
| ENSG00000134070 | IRAK2 | Interleukin 1 receptor associated kinase 2 | 0.001241 |
| ENSG00000144452 | ABCA12 | ATP binding cassette subfamily A member 12 | 0.001241 |
| ENSG00000160469 | BRSK1 | BR serine/threonine kinase 1 | 0.001282 |
| ENSG00000085998 | POMGNT1 | Protein O-linked mannose N-acetylglucosaminyltransferase 1 (beta 1,2-) | 0.001319 |
| ENSG00000156671 | SAMD8 | Sterile alpha motif domain containing 8 | 0.001324 |
| ENSG00000174684 | B4GAT1 | Beta-1,4-glucuronyltransferase 1 | 0.001414 |
| ENSG00000163683 | SMIM14 | Small integral membrane protein 14 | 0.00145 |
| ENSG00000142627 | EPHA2 | EPH receptor A2 | 0.001464 |
| ENSG00000048392 | RRM2B | Ribonucleotide reductase regulatory TP53 inducible subunit M2B | 0.001513 |
| ENSG00000184205 | TSPYL2 | TSPY like 2 | 0.001563 |
| ENSG00000132879 | FBXO44 | F-box protein 44 | 0.001681 |
| ENSG00000196187 | TMEM63A | Transmembrane protein 63A | 0.001702 |
| ENSG00000185669 | SNAI3 | Snail family transcriptional repressor 3 | 0.001744 |
| ENSG00000179163 | FUCA1 | Alpha-L-fucosidase 1 | 0.001923 |
| ENSG00000166233 | ARIH1 | Ariadne RBR E3 ubiquitin protein ligase 1 | 0.002131 |
| ENSG00000178184 | PARD6G | Par-6 family cell polarity regulator gamma | 0.002273 |
| ENSG00000197496 | SLC2A10 | Solute carrier family 2 member 10 | 0.002423 |
| ENSG00000166311 | SMPD1 | Sphingomyelin phosphodiesterase 1 | 0.002441 |
| ENSG00000176531 | PHLDB3 | Pleckstrin homology like domain family B member 3 | 0.002467 |
| ENSG00000109323 | MANBA | Mannosidase beta | 0.002517 |
| ENSG00000125538 | IL1B | Interleukin 1 beta | 0.002517 |
| ENSG00000115008 | IL1A | Interleukin 1 alpha | 0.002599 |
| ENSG00000151117 | TMEM86A | Transmembrane protein 86A | 0.002599 |
| ENSG00000101298 | SNPH | Syntaphilin | 0.002627 |
| ENSG00000170271 | FAXDC2 | Fatty acid hydroxylase domain containing 2 | 0.002631 |
| ENSG00000157150 | TIMP4 | TIMP metallopeptidase inhibitor 4 | 0.002757 |
| ENSG00000165861 | ZFYVE1 | Zinc finger FYVE-type containing 1 | 0.002785 |

| | | | |
|---|---|---|---|
| ENSG00000021826 | CPS1 | Carbamoyl-phosphate synthase 1 | 0.002804 |
| ENSG00000188766 | SPRED3 | Sprouty related EVH1 domain containing 3 | 0.002817 |
| ENSG00000059122 | FLYWCH1 | FLYWCH-type zinc finger 1 | 0.002831 |
| ENSG00000138606 | SHF | Src homology 2 domain containing F | 0.002851 |
| ENSG00000135828 | RNASEL | Ribonuclease L | 0.002908 |
| ENSG00000124098 | FAM210B | Family with sequence similarity 210 member B | 0.002966 |
| ENSG00000148344 | PTGES | Prostaglandin E synthase | 0.003043 |
| ENSG00000172738 | TMEM217 | Transmembrane protein 217 | 0.003065 |
| ENSG00000164331 | ANKRA2 | Ankyrin repeat family A member 2 | 0.003113 |
| ENSG00000142459 | EVI5L | Ecotropic viral integration site 5 like | 0.003197 |
| ENSG00000137207 | YIPF3 | Yip1 domain family member 3 | 0.003408 |
| ENSG00000163703 | CRELD1 | Cysteine rich with EGF like domains 1 | 0.003548 |
| ENSG00000064687 | ABCA7 | ATP binding cassette subfamily A member 7 | 0.003625 |
| ENSG00000160460 | AC020929.1 | Spectrin beta, non-erythrocytic 4 | 0.003738 |
| ENSG00000067369 | TP53BP1 | Tumor protein p53 binding protein 1 | 0.003938 |
| ENSG00000135709 | KIAA0513 | Kiaa0513 | 0.003938 |
| ENSG00000198722 | UNC13B | Unc-13 homolog B | 0.004119 |
| ENSG00000084764 | MAPRE3 | Microtubule associated protein RP/EB family member 3 | 0.004163 |
| ENSG00000163947 | ARHGEF3 | Rho guanine nucleotide exchange factor 3 | 0.004163 |
| ENSG00000142687 | KIAA0319L | KIAA0319 like | 0.004222 |
| ENSG00000146278 | PNRC1 | Proline rich nuclear receptor coactivator 1 | 0.004421 |
| ENSG00000103742 | IGDCC4 | Immunoglobulin superfamily DCC subclass member 4 | 0.004506 |
| ENSG00000056558 | TRAF1 | TNF receptor associated factor 1 | 0.004527 |
| ENSG00000133460 | SLC2A11 | Solute carrier family 2 member 11 | 0.004527 |
| ENSG00000196152 | ZNF79 | Zinc finger protein 79 | 0.004664 |
| ENSG00000063854 | HAGH | Hydroxyacylglutathione hydrolase | 0.005015 |
| ENSG00000086544 | ITPKC | Inositol-trisphosphate 3-kinase C | 0.005288 |
| ENSG00000078237 | TIGAR | TP53 induced glycolysis regulatory phosphatase | 0.005419 |
| ENSG00000139508 | SLC46A3 | Solute carrier family 46 member 3 | 0.005919 |
| ENSG00000170734 | POLH | DNA polymerase eta | 0.005974 |
| ENSG00000180155 | LYNX1 | Ly6/neurotoxin 1 | 0.006029 |
| ENSG00000105204 | DYRK1B | Dual specificity tyrosine phosphorylation regulated kinase 1B | 0.006123 |
| ENSG00000173214 | MFSD4B | Major facilitator superfamily domain containing 4B | 0.006123 |
| ENSG00000118898 | PPL | Periplakin | 0.006231 |
| ENSG00000179277 | MEIS3P1 | Meis homeobox 3 pseudogene 1 | 0.006423 |
| ENSG00000197429 | IPP | Intracisternal A particle-promoted polypeptide | 0.006446 |
| ENSG00000127914 | AKAP9 | A-kinase anchoring protein 9 | 0.006666 |
| ENSG00000029534 | ANK1 | Ankyrin 1 | 0.006967 |
| ENSG00000186866 | POFUT2 | Protein O-fucosyltransferase 2 | 0.007011 |
| ENSG00000260300 | | | 0.007032 |
| ENSG00000137880 | GCHFR | GTP cyclohydrolase I feedback regulator | 0.007257 |
| ENSG00000156804 | FBXO32 | F-box protein 32 | 0.007462 |
| ENSG00000182580 | EPHB3 | EPH receptor B3 | 0.007504 |
| ENSG00000099875 | MKNK2 | MAPK interacting serine/threonine kinase 2 | 0.008133 |
| ENSG00000158769 | F11R | F11 receptor | 0.008233 |
| ENSG00000054148 | PHPT1 | Phosphohistidine phosphatase 1 | 0.008371 |
| ENSG00000141504 | SAT2 | Spermidine/spermine N1-acetyltransferase family member 2 | 0.00841 |
| ENSG00000143390 | RFX5 | Regulatory factor X5 | 0.00841 |
| ENSG00000139266 | MARCHF9 | Membrane associated ring-CH-type finger 9 | 0.008567 |

| | | | |
|---|---|---|---|
| ENSG00000181045 | SLC26A11 | Solute carrier family 26 member 11 | 0.008812 |
| ENSG00000181754 | AMIGO1 | Adhesion molecule with Ig like domain 1 | 0.008831 |
| ENSG00000186591 | UBE2H | Ubiquitin conjugating enzyme E2 H | 0.008957 |
| ENSG00000255112 | CHMP1B | Charged multivesicular body protein 1B | 0.009272 |
| ENSG00000277639 | AC007906.2 | Novel protein | 0.009577 |
| ENSG00000185339 | TCN2 | Transcobalamin 2 | 0.009642 |
| ENSG00000274180 | NATD1 | N-acetyltransferase domain containing 1 | 0.01023 |
| ENSG00000088340 | FER1L4 | Fer-1 like family member 4 (pseudogene) | 0.010493 |
| ENSG00000034713 | GABARAPL2 | GABA type A receptor associated protein like 2 | 0.010759 |
| ENSG00000158467 | AHCYL2 | Adenosylhomocysteinase like 2 | 0.010975 |
| ENSG00000169902 | TPST1 | Tyrosylprotein sulfotransferase 1 | 0.010991 |
| ENSG00000136003 | ISCU | Iron-sulfur cluster assembly enzyme | 0.01108 |
| ENSG00000128394 | APOBEC3F | Apolipoprotein B mrna editing enzyme catalytic subunit 3F | 0.011326 |
| ENSG00000111269 | CREBL2 | Camp responsive element binding protein like 2 | 0.011329 |
| ENSG00000113319 | RASGRF2 | Ras protein specific guanine nucleotide releasing factor 2 | 0.011676 |
| ENSG00000139182 | CLSTN3 | Calsyntenin 3 | 0.011719 |
| ENSG00000187688 | TRPV2 | Transient receptor potential cation channel subfamily V member 2 | 0.012032 |
| ENSG00000168216 | LMBRD1 | LMBR1 domain containing 1 | 0.012324 |
| ENSG00000012171 | SEMA3B | Semaphorin 3B | 0.012916 |
| ENSG00000140463 | BBS4 | Bardet-Biedl syndrome 4 | 0.012929 |
| ENSG00000076641 | PAG1 | Phosphoprotein membrane anchor with glycosphingolipid microdomains 1 | 0.01297 |
| ENSG00000117533 | VAMP4 | Vesicle associated membrane protein 4 | 0.013104 |
| ENSG00000160539 | PLPP7 | Phospholipid phosphatase 7 (inactive) | 0.013293 |
| ENSG00000237094 | AL732372.2 | Pseudogene similar to part of septin 14 SEPT14 | 0.013765 |
| ENSG00000175854 | SWI5 | SWI5 homologous recombination repair protein | 0.013831 |
| ENSG00000112343 | TRIM38 | Tripartite motif containing 38 | 0.013936 |
| ENSG00000173193 | PARP14 | Poly(ADP-ribose) polymerase family member 14 | 0.013936 |
| ENSG00000157954 | WIPI2 | WD repeat domain, phosphoinositide interacting 2 | 0.014002 |
| ENSG00000100100 | PIK3IP1 | Phosphoinositide-3-kinase interacting protein 1 | 0.014107 |
| ENSG00000166816 | LDHD | Lactate dehydrogenase D | 0.014434 |
| ENSG00000136874 | STX17 | Syntaxin 17 | 0.014483 |
| ENSG00000119508 | NR4A3 | Nuclear receptor subfamily 4 group A member 3 | 0.014559 |
| ENSG00000011243 | AKAP8L | A-kinase anchoring protein 8 like | 0.014597 |
| ENSG00000197892 | KIF13B | Kinesin family member 13B | 0.014938 |
| ENSG00000163956 | LRPAP1 | LDL receptor related protein associated protein 1 | 0.015172 |
| ENSG00000184588 | PDE4B | Phosphodiesterase 4B | 0.015719 |
| ENSG00000105649 | RAB3A | RAB3A, member RAS oncogene family | 0.015913 |
| ENSG00000136928 | GABBR2 | Gamma-aminobutyric acid type B receptor subunit 2 | 0.015976 |
| ENSG00000087085 | ACHE | Acetylcholinesterase (Cartwright blood group) | 0.016047 |
| ENSG00000103021 | CCDC113 | Coiled-coil domain containing 113 | 0.016057 |
| ENSG00000132256 | TRIM5 | Tripartite motif containing 5 | 0.01666 |
| ENSG00000119899 | SLC17A5 | Solute carrier family 17 member 5 | 0.01685 |
| ENSG00000137103 | TMEM8B | Transmembrane protein 8B | 0.017031 |
| ENSG00000065357 | DGKA | Diacylglycerol kinase alpha | 0.017314 |
| ENSG00000186642 | PDE2A | Phosphodiesterase 2A | 0.017314 |
| ENSG00000213463 | SYNJ2BP | Synaptojanin 2 binding protein | 0.017466 |
| ENSG00000283050 | GTF2IP12 | General transcription factor iii pseudogene 12 | 0.018207 |
| ENSG00000100577 | GSTZ1 | Glutathione S-transferase zeta 1 | 0.018272 |

| | | | |
|---|---|---|---|
| ENSG00000121310 | ECHDC2 | Enoyl-coa hydratase domain containing 2 | 0.018318 |
| ENSG00000149577 | SIDT2 | SID1 transmembrane family member 2 | 0.018318 |
| ENSG00000148541 | FAM13C | Family with sequence similarity 13 member C | 0.01862 |
| ENSG00000175793 | SFN | Stratifin | 0.019005 |
| ENSG00000170855 | TRIAP1 | TP53 regulated inhibitor of apoptosis 1 | 0.019174 |
| ENSG00000173992 | CCS | Copper chaperone for superoxide dismutase | 0.019205 |
| ENSG00000161544 | CYGB | Cytoglobin | 0.019473 |
| ENSG00000168300 | PCMTD1 | Protein-L-isoaspartate (D-aspartate) O-methyltransferase domain containing 1 | 0.019636 |
| ENSG00000178607 | ERN1 | Endoplasmic reticulum to nucleus signaling 1 | 0.019691 |
| ENSG00000166145 | SPINT1 | Serine peptidase inhibitor, Kunitz type 1 | 0.020192 |
| ENSG00000243749 | TMEM35B | Transmembrane protein 35B | 0.020192 |
| ENSG00000129472 | RAB2B | RAB2B, member RAS oncogene family | 0.020472 |
| ENSG00000121743 | GJA3 | Gap junction protein alpha 3 | 0.021079 |
| ENSG00000124067 | SLC12A4 | Solute carrier family 12 member 4 | 0.021132 |
| ENSG00000197302 | ZNF720 | Zinc finger protein 720 | 0.021656 |
| ENSG00000115129 | TP53I3 | Tumor protein p53 inducible protein 3 | 0.021687 |
| ENSG00000116209 | TMEM59 | Transmembrane protein 59 | 0.022305 |
| ENSG00000089847 | ANKRD24 | Ankyrin repeat domain 24 | 0.022555 |
| ENSG00000131386 | GALNT15 | Polypeptide N-acetylgalactosaminyltransferase 15 | 0.02256 |
| ENSG00000025434 | NR1H3 | Nuclear receptor subfamily 1 group H member 3 | 0.022759 |
| ENSG00000076344 | RGS11 | Regulator of G protein signaling 11 | 0.022809 |
| ENSG00000213930 | GALT | Galactose-1-phosphate uridylyltransferase | 0.023225 |
| ENSG00000137145 | DENND4C | DENN domain containing 4C | 0.023499 |
| ENSG00000100647 | SUSD6 | Sushi domain containing 6 | 0.024199 |
| ENSG00000135823 | STX6 | Syntaxin 6 | 0.024199 |
| ENSG00000143409 | MINDY1 | MINDY lysine 48 deubiquitinase 1 | 0.024199 |
| ENSG00000109103 | UNC119 | Unc-119 lipid binding chaperone | 0.024368 |
| ENSG00000138641 | HERC3 | HECT and RLD domain containing E3 ubiquitin protein ligase 3 | 0.025289 |
| ENSG00000172508 | CARNS1 | Carnosine synthase 1 | 0.025289 |
| ENSG00000168890 | TMEM150A | Transmembrane protein 150A | 0.025662 |
| ENSG00000124406 | ATP8A1 | Atpase phospholipid transporting 8A1 | 0.025871 |
| ENSG00000170581 | STAT2 | Signal transducer and activator of transcription 2 | 0.026216 |
| ENSG00000173221 | GLRX | Glutaredoxin | 0.026216 |
| ENSG00000165475 | CRYL1 | Crystallin lambda 1 | 0.026375 |
| ENSG00000142961 | MOB3C | MOB kinase activator 3C | 0.026764 |
| ENSG00000114626 | ABTB1 | Ankyrin repeat and BTB domain containing 1 | 0.027893 |
| ENSG00000136859 | ANGPTL2 | Angiopoietin like 2 | 0.027893 |
| ENSG00000169242 | EFNA1 | Ephrin A1 | 0.027893 |
| ENSG00000121671 | CRY2 | Cryptochrome circadian regulator 2 | 0.028045 |
| ENSG00000166313 | APBB1 | Amyloid beta precursor protein binding family B member 1 | 0.028045 |
| ENSG00000171219 | CDC42BPG | CDC42 binding protein kinase gamma | 0.028222 |
| ENSG00000137216 | TMEM63B | Transmembrane protein 63B | 0.028398 |
| ENSG00000169621 | APLF | Aprataxin and PNKP like factor | 0.028923 |
| ENSG00000285188 | AC008397.2 | Camp-specific 3',5'-cyclic phosphodiesterase 4C | 0.028923 |
| ENSG00000233369 | GTF2IP4 | General transcription factor iii pseudogene 4 | 0.029097 |
| ENSG00000105967 | TFEC | Transcription factor EC | 0.029244 |
| ENSG00000178038 | ALS2CL | ALS2 C-terminal like | 0.02939 |
| ENSG00000180104 | EXOC3 | Exocyst complex component 3 | 0.029588 |

| | | | |
|---|---|---|---|
| ENSG00000275066 | SYNRG | Synergin gamma | 0.029759 |
| ENSG00000004799 | PDK4 | Pyruvate dehydrogenase kinase 4 | 0.030153 |
| ENSG00000114796 | KLHL24 | Kelch like family member 24 | 0.030153 |
| ENSG00000159214 | CCDC24 | Coiled-coil domain containing 24 | 0.030349 |
| ENSG00000170836 | PPM1D | Protein phosphatase, Mg2+/Mn2+ dependent 1D | 0.030545 |
| ENSG00000095970 | TREM2 | Triggering receptor expressed on myeloid cells 2 | 0.030687 |
| ENSG00000120709 | FAM53C | Family with sequence similarity 53 member C | 0.031243 |
| ENSG00000166046 | TCP11L2 | T-complex 11 like 2 | 0.031243 |
| ENSG00000006282 | SPATA20 | Spermatogenesis associated 20 | 0.031382 |
| ENSG00000132510 | KDM6B | Lysine demethylase 6B | 0.031382 |
| ENSG00000077150 | NFKB2 | Nuclear factor kappa B subunit 2 | 0.031713 |
| ENSG00000103485 | QPRT | Quinolinate phosphoribosyltransferase | 0.031822 |
| ENSG00000165272 | AQP3 | Aquaporin 3 (Gill blood group) | 0.031822 |
| ENSG00000250770 | AC005865.1 | Tetraspanin 11 (TSPAN11) pseudogene | 0.031822 |
| ENSG00000189241 | TSPYL1 | TSPY like 1 | 0.032148 |
| ENSG00000146232 | NFKBIE | NFKB inhibitor epsilon | 0.032473 |
| ENSG00000070540 | WIPI1 | WD repeat domain, phosphoinositide interacting 1 | 0.03288 |
| ENSG00000167703 | SLC43A2 | Solute carrier family 43 member 2 | 0.033852 |
| ENSG00000132623 | ANKEF1 | Ankyrin repeat and EF-hand domain containing 1 | 0.03404 |
| ENSG00000113328 | CCNG1 | Cyclin G1 | 0.034444 |
| ENSG00000231551 | AC245100.4 | Phosphodiesterase 4D interacting protein (PDE4DIP) pseudogene | 0.034817 |
| ENSG00000135525 | MAP7 | Microtubule associated protein 7 | 0.035159 |
| ENSG00000240771 | ARHGEF25 | Rho guanine nucleotide exchange factor 25 | 0.035159 |
| ENSG00000206561 | COLQ | Collagen like tail subunit of asymmetric acetylcholinesterase | 0.035684 |
| ENSG00000068912 | ERLEC1 | Endoplasmic reticulum lectin 1 | 0.036298 |
| ENSG00000136114 | THSD1 | Thrombospondin type 1 domain containing 1 | 0.036754 |
| ENSG00000130363 | RSPH3 | Radial spoke head 3 | 0.036873 |
| ENSG00000249624 | AP000295.1 | Novel protein | 0.036873 |
| ENSG00000117305 | HMGCL | 3-hydroxy-3-methylglutaryl-coa lyase | 0.037472 |
| ENSG00000027869 | SH2D2A | SH2 domain containing 2A | 0.037495 |
| ENSG00000265808 | SEC22B | SEC22 homolog B, vesicle trafficking protein (gene/pseudogene) | 0.037495 |
| ENSG00000164088 | PPM1M | Protein phosphatase, Mg2+/Mn2+ dependent 1M | 0.037611 |
| ENSG00000163449 | TMEM169 | Transmembrane protein 169 | 0.037789 |
| ENSG00000006283 | CACNA1G | Calcium voltage-gated channel subunit alpha1 G | 0.037905 |
| ENSG00000106992 | AK1 | Adenylate kinase 1 | 0.037905 |
| ENSG00000102981 | PARD6A | Par-6 family cell polarity regulator alpha | 0.038019 |
| ENSG00000111540 | RAB5B | RAB5B, member RAS oncogene family | 0.038019 |
| ENSG00000105514 | RAB3D | RAB3D, member RAS oncogene family | 0.038133 |
| ENSG00000179941 | BBS10 | Bardet-Biedl syndrome 10 | 0.038133 |
| ENSG00000138036 | DYNC2LI1 | Dynein cytoplasmic 2 light intermediate chain 1 | 0.038215 |
| ENSG00000141295 | SCRN2 | Secernin 2 | 0.038215 |
| ENSG00000095066 | HOOK2 | Hook microtubule tethering protein 2 | 0.038853 |
| ENSG00000163659 | TIPARP | TCDD inducible poly(ADP-ribose) polymerase | 0.038853 |
| ENSG00000167971 | CASKIN1 | CASK interacting protein 1 | 0.038996 |
| ENSG00000155957 | TMBIM4 | Transmembrane BAX inhibitor motif containing 4 | 0.03917 |
| ENSG00000161509 | GRIN2C | Glutamate ionotropic receptor NMDA type subunit 2C | 0.039518 |
| ENSG00000186814 | ZSCAN30 | Zinc finger and SCAN domain containing 30 | 0.039518 |
| ENSG00000158604 | TMED4 | Transmembrane p24 trafficking protein 4 | 0.039659 |

| | | | |
|---|---|---|---|
| ENSG00000128604 | IRF5 | Interferon regulatory factor 5 | 0.040004 |
| ENSG00000111275 | ALDH2 | Aldehyde dehydrogenase 2 family member | 0.040455 |
| ENSG00000128268 | MGAT3 | Beta-1,4-mannosyl-glycoprotein 4-beta-N-acetylglucosaminyltransferase | 0.040455 |
| ENSG00000132471 | WBP2 | WW domain binding protein 2 | 0.041276 |
| ENSG00000132849 | PATJ | PATJ crumbs cell polarity complex component | 0.041276 |
| ENSG00000092758 | COL9A3 | Collagen type IX alpha 3 chain | 0.041615 |
| ENSG00000285976 | AL135905.2 | Novel protein | 0.041751 |
| ENSG00000171174 | RBKS | Ribokinase | 0.041852 |
| ENSG00000130244 | FAM98C | Family with sequence similarity 98 member C | 0.041987 |
| ENSG00000138642 | HERC6 | HECT and RLD domain containing E3 ubiquitin protein ligase family member 6 | 0.041987 |
| ENSG00000135414 | GDF11 | Growth differentiation factor 11 | 0.042155 |
| ENSG00000090238 | YPEL3 | Yippee like 3 | 0.042589 |
| ENSG00000126709 | IFI6 | Interferon alpha inducible protein 6 | 0.042589 |
| ENSG00000213918 | DNASE1 | Deoxyribonuclease 1 | 0.044011 |
| ENSG00000173918 | C1QTNF1 | C1q and TNF related 1 | 0.044175 |
| ENSG00000171236 | LRG1 | Leucine rich alpha-2-glycoprotein 1 | 0.044503 |
| ENSG00000198208 | RPS6KL1 | Ribosomal protein S6 kinase like 1 | 0.044666 |
| ENSG00000167642 | SPINT2 | Serine peptidase inhibitor, Kunitz type 2 | 0.044957 |
| ENSG00000171408 | PDE7B | Phosphodiesterase 7B | 0.046018 |
| ENSG00000167986 | DDB1 | Damage specific DNA binding protein 1 | 0.04639 |
| ENSG00000185133 | INPP5J | Inositol polyphosphate-5-phosphatase J | 0.04639 |
| ENSG00000225511 | LINC00475 | Long intergenic non-protein coding RNA 475 | 0.04639 |
| ENSG00000182473 | EXOC7 | Exocyst complex component 7 | 0.046477 |
| ENSG00000026950 | BTN3A1 | Butyrophilin subfamily 3 member A1 | 0.0466 |
| ENSG00000127125 | PPCS | Phosphopantothenoylcysteine synthetase | 0.046918 |
| ENSG00000100731 | PCNX1 | Pecanex 1 | 0.047392 |
| ENSG00000107537 | PHYH | Phytanoyl-coa 2-hydroxylase | 0.04767 |
| ENSG00000141376 | BCAS3 | BCAS3 microtubule associated cell migration factor | 0.04767 |
| ENSG00000197872 | FAM49A | Family with sequence similarity 49 member A | 0.047827 |
| ENSG00000108306 | FBXL20 | F-box and leucine rich repeat protein 20 | 0.048222 |
| ENSG00000186994 | KANK3 | KN motif and ankyrin repeat domains 3 | 0.048222 |
| ENSG00000088726 | TMEM40 | Transmembrane protein 40 | 0.048341 |
| ENSG00000116183 | PAPPA2 | Pappalysin 2 | 0.048341 |
| ENSG00000170634 | ACYP2 | Acylphosphatase 2 | 0.048652 |
| ENSG00000239382 | ALKBH6 | Alkb homolog 6 | 0.048807 |
| ENSG00000116017 | ARID3A | AT-rich interaction domain 3A | 0.049079 |
| ENSG00000089486 | CDIP1 | Cell death inducing p53 target 1 | 0.049312 |
| ENSG00000167676 | PLIN4 | Perilipin 4 | 0.049312 |
| ENSG00000184117 | NIPSNAP1 | Nipsnap homolog 1 | 0.049312 |
| ENSG00000250510 | GPR162 | G protein-coupled receptor 162 | 0.049772 |
| **Down-Regulated Genes↓** | | | |
| ENSG00000171848 | RRM2 | Ribonucleotide reductase regulatory subunit M2 | 2.30E-94 |
| ENSG00000148773 | MKI67 | Marker of proliferation Ki-67 | 7.25E-89 |
| ENSG00000189057 | FAM111B | Family with sequence similarity 111 member B | 2.04E-78 |
| ENSG00000145386 | CCNA2 | Cyclin A2 | 7.75E-75 |
| ENSG00000101057 | MYBL2 | MYB proto-oncogene like 2 | 2.95E-69 |
| ENSG00000203811 | H3C14 | H3 clustered histone 14 | 6.26E-67 |
| ENSG00000174371 | EXO1 | Exonuclease 1 | 6.70E-65 |
| ENSG00000166851 | PLK1 | Polo like kinase 1 | 1.01E-64 |

| | | | |
|---|---|---|---|
| ENSG00000094804 | CDC6 | Cell division cycle 6 | 3.12E-61 |
| ENSG00000286522 | H3C2 | H3 clustered histone 2 | 1.17E-60 |
| ENSG00000170312 | CDK1 | Cyclin dependent kinase 1 | 1.94E-60 |
| ENSG00000143476 | DTL | Denticleless E3 ubiquitin protein ligase homolog | 7.63E-54 |
| ENSG00000134690 | CDCA8 | Cell division cycle associated 8 | 2.75E-53 |
| ENSG00000184357 | H1-5 | H1.5 linker histone, cluster member | 2.63E-52 |
| ENSG00000088325 | TPX2 | TPX2 microtubule nucleation factor | 5.98E-51 |
| ENSG00000121152 | NCAPH | Non-SMC condensin I complex subunit H | 2.44E-50 |
| ENSG00000146670 | CDCA5 | Cell division cycle associated 5 | 2.67E-49 |
| ENSG00000109805 | NCAPG | Non-SMC condensin I complex subunit G | 7.17E-49 |
| ENSG00000105011 | ASF1B | Anti-silencing function 1B histone chaperone | 1.12E-48 |
| ENSG00000183856 | IQGAP3 | IQ motif containing gtpase activating protein 3 | 1.93E-47 |
| ENSG00000123485 | HJURP | Holliday junction recognition protein | 4.30E-47 |
| ENSG00000101447 | FAM83D | Family with sequence similarity 83 member D | 1.08E-45 |
| ENSG00000126787 | DLGAP5 | DLG associated protein 5 | 1.41E-45 |
| ENSG00000111665 | CDCA3 | Cell division cycle associated 3 | 1.98E-45 |
| ENSG00000175305 | CCNE2 | Cyclin E2 | 1.75E-43 |
| ENSG00000093009 | CDC45 | Cell division cycle 45 | 6.26E-43 |
| ENSG00000168078 | PBK | PDZ binding kinase | 6.94E-43 |
| ENSG00000138180 | CEP55 | Centrosomal protein 55 | 7.27E-43 |
| ENSG00000065328 | MCM10 | Minichromosome maintenance 10 replication initiation factor | 2.68E-42 |
| ENSG00000178999 | AURKB | Aurora kinase B | 1.54E-40 |
| ENSG00000198901 | PRC1 | Protein regulator of cytokinesis 1 | 7.13E-39 |
| ENSG00000122952 | ZWINT | ZW10 interacting kinetochore protein | 9.99E-39 |
| ENSG00000186185 | KIF18B | Kinesin family member 18B | 1.68E-38 |
| ENSG00000169607 | CKAP2L | Cytoskeleton associated protein 2 like | 2.20E-38 |
| ENSG00000168496 | FEN1 | Flap structure-specific endonuclease 1 | 4.87E-38 |
| ENSG00000276043 | UHRF1 | Ubiquitin like with PHD and ring finger domains 1 | 6.49E-38 |
| ENSG00000111206 | FOXM1 | Forkhead box M1 | 1.52E-37 |
| ENSG00000165304 | MELK | Maternal embryonic leucine zipper kinase | 2.45E-37 |
| ENSG00000167900 | TK1 | Thymidine kinase 1 | 5.48E-37 |
| ENSG00000197153 | H3C12 | H3 clustered histone 12 | 9.95E-37 |
| ENSG00000092853 | CLSPN | Claspin | 1.51E-36 |
| ENSG00000165480 | SKA3 | Spindle and kinetochore associated complex subunit 3 | 3.55E-36 |
| ENSG00000112742 | TTK | TTK protein kinase | 3.72E-36 |
| ENSG00000127564 | PKMYT1 | Protein kinase, membrane associated tyrosine/threonine 1 | 6.84E-36 |
| ENSG00000188486 | H2AX | H2A.X variant histone | 1.31E-35 |
| ENSG00000100297 | MCM5 | Minichromosome maintenance complex component 5 | 1.60E-35 |
| ENSG00000137804 | NUSAP1 | Nucleolar and spindle associated protein 1 | 5.45E-35 |
| ENSG00000167513 | CDT1 | Chromatin licensing and DNA replication factor 1 | 8.21E-35 |
| ENSG00000131153 | GINS2 | GINS complex subunit 2 | 1.20E-34 |
| ENSG00000085840 | ORC1 | Origin recognition complex subunit 1 | 2.05E-34 |
| ENSG00000111247 | RAD51AP1 | RAD51 associated protein 1 | 3.36E-34 |
| ENSG00000071539 | TRIP13 | Thyroid hormone receptor interactor 13 | 3.41E-34 |
| ENSG00000161888 | SPC24 | SPC24 component of NDC80 kinetochore complex | 9.15E-34 |
| ENSG00000075218 | GTSE1 | G2 and S-phase expressed 1 | 5.62E-33 |
| ENSG00000166803 | PCLAF | PCNA clamp associated factor | 6.45E-33 |
| ENSG00000161800 | RACGAP1 | Rac gtpase activating protein 1 | 6.99E-33 |
| ENSG00000274997 | H2AC12 | H2A clustered histone 12 | 8.31E-33 |
| ENSG00000144354 | CDCA7 | Cell division cycle associated 7 | 9.53E-33 |

| | | | |
|---|---|---|---|
| ENSG00000112029 | FBXO5 | F-box protein 5 | 2.47E-32 |
| ENSG00000164104 | HMGB2 | High mobility group box 2 | 2.97E-32 |
| ENSG00000198826 | ARHGAP11A | Rho gtpase activating protein 11A | 7.04E-32 |
| ENSG00000138160 | KIF11 | Kinesin family member 11 | 8.98E-32 |
| ENSG00000011426 | ANLN | Anillin actin binding protein | 1.66E-31 |
| ENSG00000092470 | WDR76 | WD repeat domain 76 | 1.98E-31 |
| ENSG00000073111 | MCM2 | Minichromosome maintenance complex component 2 | 2.34E-31 |
| ENSG00000024526 | DEPDC1 | DEP domain containing 1 | 7.22E-31 |
| ENSG00000113368 | LMNB1 | Lamin B1 | 7.23E-31 |
| ENSG00000186871 | ERCC6L | ERCC excision repair 6 like, spindle assembly checkpoint helicase | 1.41E-30 |
| ENSG00000007968 | E2F2 | E2F transcription factor 2 | 1.76E-30 |
| ENSG00000142945 | KIF2C | Kinesin family member 2C | 1.88E-30 |
| ENSG00000196747 | H2AC13 | H2A clustered histone 13 | 5.84E-30 |
| ENSG00000278463 | H2AC4 | H2A clustered histone 4 | 5.84E-30 |
| ENSG00000112984 | KIF20A | Kinesin family member 20A | 1.24E-29 |
| ENSG00000276903 | H2AC16 | H2A clustered histone 16 | 2.43E-29 |
| ENSG00000175063 | UBE2C | Ubiquitin conjugating enzyme E2 C | 2.45E-29 |
| ENSG00000276368 | H2AC14 | H2A clustered histone 14 | 1.07E-28 |
| ENSG00000277075 | H2AC8 | H2A clustered histone 8 | 2.14E-28 |
| ENSG00000119969 | HELLS | Helicase, lymphoid specific | 4.73E-28 |
| ENSG00000122966 | CIT | Citron rho-interacting serine/threonine kinase | 5.85E-28 |
| ENSG00000274641 | H2BC17 | H2B clustered histone 17 | 6.07E-28 |
| ENSG00000171241 | SHCBP1 | SHC binding and spindle associated 1 | 6.07E-28 |
| ENSG00000131747 | TOP2A | DNA topoisomerase II alpha | 8.14E-28 |
| ENSG00000143228 | NUF2 | NUF2 component of NDC80 kinetochore complex | 3.49E-27 |
| ENSG00000166508 | MCM7 | Minichromosome maintenance complex component 7 | 4.07E-27 |
| ENSG00000278677 | H2AC17 | H2A clustered histone 17 | 4.45E-27 |
| ENSG00000101412 | E2F1 | E2F transcription factor 1 | 2.24E-26 |
| ENSG00000196787 | H2AC11 | H2A clustered histone 11 | 3.45E-26 |
| ENSG00000137807 | KIF23 | Kinesin family member 23 | 3.84E-26 |
| ENSG00000138778 | CENPE | Centromere protein E | 5.78E-26 |
| ENSG00000013810 | TACC3 | Transforming acidic coiled-coil containing protein 3 | 1.41E-25 |
| ENSG00000068489 | PRR11 | Proline rich 11 | 1.68E-25 |
| ENSG00000183598 | H3C13 | H3 clustered histone 13 | 3.24E-25 |
| ENSG00000101003 | GINS1 | GINS complex subunit 1 | 3.66E-25 |
| ENSG00000282988 | AL031777.2 | Novel protein | 5.24E-25 |
| ENSG00000156970 | BUB1B | BUB1 mitotic checkpoint serine/threonine kinase B | 1.05E-24 |
| ENSG00000151725 | CENPU | Centromere protein U | 1.55E-24 |
| ENSG00000171320 | ESCO2 | Establishment of sister chromatid cohesion N-acetyltransferase 2 | 2.27E-24 |
| ENSG00000162073 | PAQR4 | Progestin and adipoq receptor family member 4 | 2.80E-24 |
| ENSG00000165490 | DDIAS | DNA damage induced apoptosis suppressor | 2.99E-24 |
| ENSG00000169679 | BUB1 | BUB1 mitotic checkpoint serine/threonine kinase | 3.74E-24 |
| ENSG00000144554 | FANCD2 | FA complementation group D2 | 5.20E-24 |
| ENSG00000186193 | SAPCD2 | Suppressor APC domain containing 2 | 6.28E-24 |
| ENSG00000117650 | NEK2 | NIMA related kinase 2 | 1.26E-23 |
| ENSG00000120802 | TMPO | Thymopoietin | 1.64E-23 |
| ENSG00000164109 | MAD2L1 | Mitotic arrest deficient 2 like 1 | 2.63E-23 |
| ENSG00000167670 | CHAF1A | Chromatin assembly factor 1 subunit A | 5.68E-23 |
| ENSG00000112118 | MCM3 | Minichromosome maintenance complex component 3 | 6.03E-23 |

| | | | |
|---|---|---|---|
| ENSG00000051180 | RAD51 | RAD51 recombinase | 2.39E-22 |
| ENSG00000159259 | CHAF1B | Chromatin assembly factor 1 subunit B | 2.60E-22 |
| ENSG00000134057 | CCNB1 | Cyclin B1 | 2.75E-22 |
| ENSG00000077152 | UBE2T | Ubiquitin conjugating enzyme E2 T | 4.31E-22 |
| ENSG00000129195 | PIMREG | PICALM interacting mitotic regulator | 8.03E-22 |
| ENSG00000168298 | H1-4 | H1.4 linker histone, cluster member | 8.99E-22 |
| ENSG00000133119 | RFC3 | Replication factor C subunit 3 | 1.12E-21 |
| ENSG00000090889 | KIF4A | Kinesin family member 4A | 1.58E-21 |
| ENSG00000165244 | ZNF367 | Zinc finger protein 367 | 3.38E-21 |
| ENSG00000100162 | CENPM | Centromere protein M | 3.45E-21 |
| ENSG00000187741 | FANCA | FA complementation group A | 3.48E-21 |
| ENSG00000117399 | CDC20 | Cell division cycle 20 | 3.77E-21 |
| ENSG00000160957 | RECQL4 | Recq like helicase 4 | 3.89E-21 |
| ENSG00000066279 | ASPM | Abnormal spindle microtubule assembly | 4.69E-21 |
| ENSG00000162063 | CCNF | Cyclin F | 4.71E-21 |
| ENSG00000076003 | MCM6 | Minichromosome maintenance complex component 6 | 7.70E-21 |
| ENSG00000142731 | PLK4 | Polo like kinase 4 | 1.03E-20 |
| ENSG00000185130 | H2BC13 | H2B clustered histone 13 | 1.15E-20 |
| ENSG00000136982 | DSCC1 | DNA replication and sister chromatid cohesion 1 | 1.50E-20 |
| ENSG00000101868 | POLA1 | DNA polymerase alpha 1, catalytic subunit | 1.54E-20 |
| ENSG00000146918 | NCAPG2 | Non-SMC condensin II complex subunit G2 | 2.66E-20 |
| ENSG00000117724 | CENPF | Centromere protein F | 2.84E-20 |
| ENSG00000135451 | TROAP | Trophinin associated protein | 3.97E-20 |
| ENSG00000014138 | POLA2 | DNA polymerase alpha 2, accessory subunit | 9.02E-20 |
| ENSG00000124635 | H2BC11 | H2B clustered histone 11 | 1.12E-19 |
| ENSG00000170779 | CDCA4 | Cell division cycle associated 4 | 1.23E-19 |
| ENSG00000164087 | POC1A | POC1 centriolar protein A | 1.54E-19 |
| ENSG00000177602 | HASPIN | Histone H3 associated protein kinase | 2.14E-19 |
| ENSG00000123219 | CENPK | Centromere protein K | 3.12E-19 |
| ENSG00000277157 | H4C4 | H4 clustered histone 4 | 6.95E-19 |
| ENSG00000140534 | TICRR | TOPBP1 interacting checkpoint and replication regulator | 7.84E-19 |
| ENSG00000183688 | RFLNB | Refilin B | 8.48E-19 |
| ENSG00000124575 | H1-3 | H1.3 linker histone, cluster member | 1.25E-18 |
| ENSG00000213347 | MXD3 | MAX dimerization protein 3 | 1.28E-18 |
| ENSG00000080986 | NDC80 | NDC80 kinetochore complex component | 1.35E-18 |
| ENSG00000270882 | H4C14 | H4 clustered histone 14 | 1.91E-18 |
| ENSG00000164611 | PTTG1 | PTTG1 regulator of sister chromatid separation, securin | 2.39E-18 |
| ENSG00000107562 | CXCL12 | C-X-C motif chemokine ligand 12 | 3.10E-18 |
| ENSG00000187837 | H1-2 | H1.2 linker histone, cluster member | 3.36E-18 |
| ENSG00000135476 | ESPL1 | Extra spindle pole bodies like 1, separase | 4.99E-18 |
| ENSG00000089685 | BIRC5 | Baculoviral IAP repeat containing 5 | 5.82E-18 |
| ENSG00000131470 | PSMC3IP | PSMC3 interacting protein | 7.30E-18 |
| ENSG00000228716 | DHFR | Dihydrofolate reductase | 8.64E-18 |
| ENSG00000157456 | CCNB2 | Cyclin B2 | 9.65E-18 |
| ENSG00000129810 | SGO1 | Shugoshin 1 | 1.06E-17 |
| ENSG00000167325 | RRM1 | Ribonucleotide reductase catalytic subunit M1 | 1.59E-17 |
| ENSG00000176890 | TYMS | Thymidylate synthetase | 1.72E-17 |
| ENSG00000121211 | MND1 | Meiotic nuclear divisions 1 | 1.92E-17 |
| ENSG00000273983 | H3C8 | H3 clustered histone 8 | 2.20E-17 |
| ENSG00000111602 | TIMELESS | Timeless circadian regulator | 2.28E-17 |
| ENSG00000137812 | KNL1 | Kinetochore scaffold 1 | 2.46E-17 |

| | | | |
|---|---|---|---|
| ENSG00000184270 | H2AC21 | H2A clustered histone 21 | 2.53E-17 |
| ENSG00000275714 | H3C1 | H3 clustered histone 1 | 7.46E-17 |
| ENSG00000139618 | BRCA2 | BRCA2 DNA repair associated | 8.24E-17 |
| ENSG00000109674 | NEIL3 | Nei like DNA glycosylase 3 | 1.13E-16 |
| ENSG00000136492 | BRIP1 | BRCA1 interacting protein C-terminal helicase 1 | 1.15E-16 |
| ENSG00000164045 | CDC25A | Cell division cycle 25A | 1.28E-16 |
| ENSG00000163918 | RFC4 | Replication factor C subunit 4 | 2.26E-16 |
| ENSG00000076248 | UNG | Uracil DNA glycosylase | 3.57E-16 |
| ENSG00000149636 | DSN1 | DSN1 component of MIS12 kinetochore complex | 5.60E-16 |
| ENSG00000153044 | CENPH | Centromere protein H | 6.65E-16 |
| ENSG00000273802 | H2BC8 | H2B clustered histone 8 | 1.09E-15 |
| ENSG00000277224 | H2BC7 | H2B clustered histone 7 | 1.09E-15 |
| ENSG00000035499 | DEPDC1B | DEP domain containing 1B | 1.15E-15 |
| ENSG00000198056 | PRIM1 | DNA primase subunit 1 | 1.70E-15 |
| ENSG00000275379 | H3C11 | H3 clustered histone 11 | 1.97E-15 |
| ENSG00000158402 | CDC25C | Cell division cycle 25C | 2.38E-15 |
| ENSG00000237649 | KIFC1 | Kinesin family member C1 | 3.18E-15 |
| ENSG00000106462 | EZH2 | Enhancer of zeste 2 polycomb repressive complex 2 subunit | 5.05E-15 |
| ENSG00000138092 | CENPO | Centromere protein O | 5.43E-15 |
| ENSG00000198554 | WDHD1 | WD repeat and HMG-box DNA binding protein 1 | 5.67E-15 |
| ENSG00000272196 | H2AC19 | H2A clustered histone 19 | 5.90E-15 |
| ENSG00000104147 | OIP5 | Opa interacting protein 5 | 5.95E-15 |
| ENSG00000137310 | TCF19 | Transcription factor 19 | 6.65E-15 |
| ENSG00000197299 | BLM | BLM recq like helicase | 8.18E-15 |
| ENSG00000113810 | SMC4 | Structural maintenance of chromosomes 4 | 9.62E-15 |
| ENSG00000180198 | RCC1 | Regulator of chromosome condensation 1 | 1.04E-14 |
| ENSG00000123080 | CDKN2C | Cyclin dependent kinase inhibitor 2C | 1.33E-14 |
| ENSG00000277775 | H3C7 | H3 clustered histone 7 | 1.50E-14 |
| ENSG00000076382 | SPAG5 | Sperm associated antigen 5 | 1.53E-14 |
| ENSG00000118193 | KIF14 | Kinesin family member 14 | 1.89E-14 |
| ENSG00000163808 | KIF15 | Kinesin family member 15 | 1.89E-14 |
| ENSG00000164032 | H2AZ1 | H2A.Z variant histone 1 | 1.89E-14 |
| ENSG00000166845 | C18orf54 | Chromosome 18 open reading frame 54 | 2.02E-14 |
| ENSG00000184260 | H2AC20 | H2A clustered histone 20 | 3.18E-14 |
| ENSG00000156802 | ATAD2 | Atpase family AAA domain containing 2 | 5.01E-14 |
| ENSG00000140525 | FANCI | FA complementation group I | 6.04E-14 |
| ENSG00000221829 | FANCG | FA complementation group G | 8.53E-14 |
| ENSG00000175643 | RMI2 | Recq mediated genome instability 2 | 9.02E-14 |
| ENSG00000182010 | RTKN2 | Rhotekin 2 | 1.31E-13 |
| ENSG00000182481 | KPNA2 | Karyopherin subunit alpha 2 | 1.72E-13 |
| ENSG00000196866 | H2AC7 | H2A clustered histone 7 | 2.23E-13 |
| ENSG00000125885 | MCM8 | Minichromosome maintenance 8 homologous recombination repair factor | 2.38E-13 |
| ENSG00000117632 | STMN1 | Stathmin 1 | 2.42E-13 |
| ENSG00000278588 | H2BC10 | H2B clustered histone 10 | 2.86E-13 |
| ENSG00000097046 | CDC7 | Cell division cycle 7 | 2.97E-13 |
| ENSG00000180596 | H2BC4 | H2B clustered histone 4 | 3.02E-13 |
| ENSG00000102384 | CENPI | Centromere protein I | 4.38E-13 |
| ENSG00000278828 | H3C10 | H3 clustered histone 10 | 7.44E-13 |
| ENSG00000158164 | TMSB15A | Thymosin beta 15a | 7.54E-13 |

| | | | |
|---|---|---|---|
| ENSG00000101945 | SUV39H1 | Suppressor of variegation 3-9 homolog 1 | 1.33E-12 |
| ENSG00000112312 | GMNN | Geminin DNA replication inhibitor | 1.43E-12 |
| ENSG00000138182 | KIF20B | Kinesin family member 20B | 1.65E-12 |
| ENSG00000213551 | DNAJC9 | Dnaj heat shock protein family (Hsp40) member C9 | 2.02E-12 |
| ENSG00000145604 | SKP2 | S-phase kinase associated protein 2 | 3.11E-12 |
| ENSG00000273703 | H2BC14 | H2B clustered histone 14 | 3.32E-12 |
| ENSG00000127423 | AUNIP | Aurora kinase A and ninein interacting protein | 3.37E-12 |
| ENSG00000126215 | XRCC3 | X-ray repair cross complementing 3 | 3.75E-12 |
| ENSG00000115163 | CENPA | Centromere protein A | 5.97E-12 |
| ENSG00000174442 | ZWILCH | Zwilch kinetochore protein | 5.97E-12 |
| ENSG00000167747 | C19orf48 | Chromosome 19 open reading frame 48 | 7.20E-12 |
| ENSG00000149554 | CHEK1 | Checkpoint kinase 1 | 1.25E-11 |
| ENSG00000168393 | DTYMK | Deoxythymidylate kinase | 1.47E-11 |
| ENSG00000108106 | UBE2S | Ubiquitin conjugating enzyme E2 S | 1.47E-11 |
| ENSG00000173207 | CKS1B | CDC28 protein kinase regulatory subunit 1B | 1.47E-11 |
| ENSG00000123473 | STIL | STIL centriolar assembly protein | 1.56E-11 |
| ENSG00000160447 | PKN3 | Protein kinase N3 | 1.68E-11 |
| ENSG00000171208 | NETO2 | Neuropilin and tolloid like 2 | 2.13E-11 |
| ENSG00000118655 | DCLRE1B | DNA cross-link repair 1B | 2.26E-11 |
| ENSG00000149503 | INCENP | Inner centromere protein | 2.33E-11 |
| ENSG00000162607 | USP1 | Ubiquitin specific peptidase 1 | 3.63E-11 |
| ENSG00000186638 | KIF24 | Kinesin family member 24 | 3.85E-11 |
| ENSG00000128245 | YWHAH | Tyrosine 3-monooxygenase/tryptophan 5-monooxygenase activation protein eta | 6.10E-11 |
| ENSG00000136122 | BORA | BORA aurora kinase A activator | 7.18E-11 |
| ENSG00000185480 | PARPBP | PARP1 binding protein | 7.52E-11 |
| ENSG00000197238 | H4C11 | H4 clustered histone 11 | 8.01E-11 |
| ENSG00000179750 | APOBEC3B | Apolipoprotein B mrna editing enzyme catalytic subunit 3B | 8.52E-11 |
| ENSG00000163507 | CIP2A | Cell proliferation regulating inhibitor of protein phosphatase 2A | 9.26E-11 |
| ENSG00000196584 | XRCC2 | X-ray repair cross complementing 2 | 9.26E-11 |
| ENSG00000160949 | TONSL | Tonsoku like, DNA repair protein | 9.37E-11 |
| ENSG00000121621 | KIF18A | Kinesin family member 18A | 9.38E-11 |
| ENSG00000197061 | H4C3 | H4 clustered histone 3 | 9.76E-11 |
| ENSG00000051341 | POLQ | DNA polymerase theta | 1.04E-10 |
| ENSG00000197903 | H2BC12 | H2B clustered histone 12 | 1.05E-10 |
| ENSG00000111445 | RFC5 | Replication factor C subunit 5 | 1.15E-10 |
| ENSG00000134222 | PSRC1 | Proline and serine rich coiled-coil 1 | 1.16E-10 |
| ENSG00000104738 | MCM4 | Minichromosome maintenance complex component 4 | 1.47E-10 |
| ENSG00000128973 | CLN6 | CLN6 transmembrane ER protein | 1.49E-10 |
| ENSG00000129173 | E2F8 | E2F transcription factor 8 | 1.58E-10 |
| ENSG00000158373 | H2BC5 | H2B clustered histone 5 | 1.67E-10 |
| ENSG00000121957 | GPSM2 | G protein signaling modulator 2 | 1.68E-10 |
| ENSG00000161547 | SRSF2 | Serine and arginine rich splicing factor 2 | 1.80E-10 |
| ENSG00000120539 | MASTL | Microtubule associated serine/threonine kinase like | 2.28E-10 |
| ENSG00000159055 | MIS18A | MIS18 kinetochore protein A | 2.38E-10 |
| ENSG00000104889 | RNASEH2A | Ribonuclease H2 subunit A | 2.45E-10 |
| ENSG00000128951 | DUT | Deoxyuridine triphosphatase | 3.76E-10 |
| ENSG00000198176 | TFDP1 | Transcription factor Dp-1 | 4.37E-10 |
| ENSG00000278637 | H4C1 | H4 clustered histone 1 | 4.53E-10 |

| | | | |
|---|---|---|---|
| ENSG00000079616 | KIF22 | Kinesin family member 22 | 4.55E-10 |
| ENSG00000109084 | TMEM97 | Transmembrane protein 97 | 4.78E-10 |
| ENSG00000105486 | LIG1 | DNA ligase 1 | 4.81E-10 |
| ENSG00000203814 | H2BC18 | H2B clustered histone 18 | 5.42E-10 |
| ENSG00000285920 | AC087721.2 | Novel protein | 5.58E-10 |
| ENSG00000101224 | CDC25B | Cell division cycle 25B | 5.63E-10 |
| ENSG00000099901 | RANBP1 | RAN binding protein 1 | 6.39E-10 |
| ENSG00000203760 | CENPW | Centromere protein W | 6.76E-10 |
| ENSG00000184445 | KNTC1 | Kinetochore associated 1 | 7.70E-10 |
| ENSG00000100479 | POLE2 | DNA polymerase epsilon 2, accessory subunit | 8.85E-10 |
| ENSG00000148019 | CEP78 | Centrosomal protein 78 | 9.22E-10 |
| ENSG00000013573 | DDX11 | DEAD/H-box helicase 11 | 1.06E-09 |
| ENSG00000100629 | CEP128 | Centrosomal protein 128 | 1.23E-09 |
| ENSG00000146674 | IGFBP3 | Insulin like growth factor binding protein 3 | 1.23E-09 |
| ENSG00000234289 | H2BS1 | H2B.S histone 1 | 1.28E-09 |
| ENSG00000154678 | PDE1C | Phosphodiesterase 1C | 1.44E-09 |
| ENSG00000176619 | LMNB2 | Lamin B2 | 1.45E-09 |
| ENSG00000164649 | CDCA7L | Cell division cycle associated 7 like | 2.01E-09 |
| ENSG00000147536 | GINS4 | GINS complex subunit 4 | 2.02E-09 |
| ENSG00000100714 | MTHFD1 | Methylenetetrahydrofolate dehydrogenase, cyclohydrolase and formyltetrahydrofolate synthetase 1 | 2.27E-09 |
| ENSG00000275591 | XKR5 | XK related 5 | 2.70E-09 |
| ENSG00000119333 | WDR34 | WD repeat domain 34 | 3.51E-09 |
| ENSG00000146263 | MMS22L | MMS22 like, DNA repair protein | 3.83E-09 |
| ENSG00000146410 | MTFR2 | Mitochondrial fission regulator 2 | 4.03E-09 |
| ENSG00000077514 | POLD3 | DNA polymerase delta 3, accessory subunit | 6.49E-09 |
| ENSG00000101361 | NOP56 | NOP56 ribonucleoprotein | 7.16E-09 |
| ENSG00000107796 | ACTA2 | Actin alpha 2, smooth muscle | 7.16E-09 |
| ENSG00000124795 | DEK | DEK proto-oncogene | 8.42E-09 |
| ENSG00000284946 | AC068831.8 | Novel protein | 1.23E-08 |
| ENSG00000115687 | PASK | PAS domain containing serine/threonine kinase | 1.34E-08 |
| ENSG00000138376 | BARD1 | BRCA1 associated RING domain 1 | 1.43E-08 |
| ENSG00000123737 | EXOSC9 | Exosome component 9 | 1.49E-08 |
| ENSG00000172244 | C5orf34 | Chromosome 5 open reading frame 34 | 1.57E-08 |
| ENSG00000092969 | TGFB2 | Transforming growth factor beta 2 | 1.70E-08 |
| ENSG00000160298 | C21orf58 | Chromosome 21 open reading frame 58 | 1.70E-08 |
| ENSG00000049541 | RFC2 | Replication factor C subunit 2 | 1.91E-08 |
| ENSG00000176974 | SHMT1 | Serine hydroxymethyltransferase 1 | 2.07E-08 |
| ENSG00000139354 | GAS2L3 | Growth arrest specific 2 like 3 | 2.10E-08 |
| ENSG00000062822 | POLD1 | DNA polymerase delta 1, catalytic subunit | 2.74E-08 |
| ENSG00000132436 | FIGNL1 | Fidgetin like 1 | 3.69E-08 |
| ENSG00000100749 | VRK1 | VRK serine/threonine kinase 1 | 4.01E-08 |
| ENSG00000187123 | LYPD6 | LY6/PLAUR domain containing 6 | 4.25E-08 |
| ENSG00000275713 | H2BC9 | H2B clustered histone 9 | 4.49E-08 |
| ENSG00000186767 | SPIN4 | Spindlin family member 4 | 4.97E-08 |
| ENSG00000151849 | CENPJ | Centromere protein J | 5.97E-08 |
| ENSG00000177084 | POLE | DNA polymerase epsilon, catalytic subunit | 6.10E-08 |
| ENSG00000091651 | ORC6 | Origin recognition complex subunit 6 | 6.47E-08 |
| ENSG00000154146 | NRGN | Neurogranin | 7.63E-08 |
| ENSG00000278705 | H4C2 | H4 clustered histone 2 | 7.75E-08 |
| ENSG00000189403 | HMGB1 | High mobility group box 1 | 7.95E-08 |

| | | | |
|---|---|---|---|
| ENSG00000137563 | GGH | Gamma-glutamyl hydrolase | 8.73E-08 |
| ENSG00000135763 | URB2 | URB2 ribosome biogenesis homolog | 8.85E-08 |
| ENSG00000158406 | H4C8 | H4 clustered histone 8 | 9.41E-08 |
| ENSG00000187514 | PTMA | Prothymosin alpha | 9.89E-08 |
| ENSG00000214826 | DDX12P | DEAD/H-box helicase 12, pseudogene | 1.18E-07 |
| ENSG00000274750 | H3C6 | H3 clustered histone 6 | 1.38E-07 |
| ENSG00000274290 | H2BC6 | H2B clustered histone 6 | 1.39E-07 |
| ENSG00000117877 | CD3EAP | CD3e molecule associated protein | 1.41E-07 |
| ENSG00000131351 | HAUS8 | HAUS augmin like complex subunit 8 | 1.46E-07 |
| ENSG00000154920 | EME1 | Essential meiotic structure-specific endonuclease 1 | 1.48E-07 |
| ENSG00000178409 | BEND3 | BEN domain containing 3 | 1.61E-07 |
| ENSG00000179431 | FJX1 | Four-jointed box kinase 1 | 1.71E-07 |
| ENSG00000137135 | ARHGEF39 | Rho guanine nucleotide exchange factor 39 | 1.93E-07 |
| ENSG00000162062 | TEDC2 | Tubulin epsilon and delta complex 2 | 2.23E-07 |
| ENSG00000109861 | CTSC | Cathepsin C | 2.43E-07 |
| ENSG00000101773 | RBBP8 | RB binding protein 8, endonuclease | 2.45E-07 |
| ENSG00000124610 | H1-1 | H1.1 linker histone, cluster member | 2.57E-07 |
| ENSG00000123374 | CDK2 | Cyclin dependent kinase 2 | 2.68E-07 |
| ENSG00000259316 | AC087632.2 | Novel protein | 3.26E-07 |
| ENSG00000166451 | CENPN | Centromere protein N | 3.70E-07 |
| ENSG00000168411 | RFWD3 | Ring finger and WD repeat domain 3 | 3.78E-07 |
| ENSG00000137124 | ALDH1B1 | Aldehyde dehydrogenase 1 family member B1 | 3.92E-07 |
| ENSG00000156398 | SFXN2 | Sideroflexin 2 | 4.08E-07 |
| ENSG00000178966 | RMI1 | Recq mediated genome instability 1 | 4.34E-07 |
| ENSG00000101938 | CHRDL1 | Chordin like 1 | 4.68E-07 |
| ENSG00000123213 | NLN | Neurolysin | 4.94E-07 |
| ENSG00000085999 | RAD54L | RAD54 like | 6.27E-07 |
| ENSG00000213186 | TRIM59 | Tripartite motif containing 59 | 7.59E-07 |
| ENSG00000159147 | DONSON | Downstream neighbor of SON | 7.83E-07 |
| ENSG00000233822 | H2BC15 | H2B clustered histone 15 | 8.01E-07 |
| ENSG00000181588 | MEX3D | Mex-3 RNA binding family member D | 8.26E-07 |
| ENSG00000123416 | TUBA1B | Tubulin alpha 1b | 8.37E-07 |
| ENSG00000006634 | DBF4 | DBF4 zinc finger | 8.41E-07 |
| ENSG00000163950 | SLBP | Stem-loop binding protein | 8.46E-07 |
| ENSG00000166801 | FAM111A | Family with sequence similarity 111 member A | 9.14E-07 |
| ENSG00000275221 | H2AC15 | H2A clustered histone 15 | 9.77E-07 |
| ENSG00000205208 | C4orf46 | Chromosome 4 open reading frame 46 | 9.94E-07 |
| ENSG00000259781 | HMGB1P6 | High mobility group box 1 pseudogene 6 | 1.11E-06 |
| ENSG00000128050 | PAICS | Phosphoribosylaminoimidazole carboxylase and phosphoribosylaminoimidazolesuccinocarboxamide synthase | 1.19E-06 |
| ENSG00000095002 | MSH2 | Muts homolog 2 | 1.19E-06 |
| ENSG00000172197 | MBOAT1 | Membrane bound O-acyltransferase domain containing 1 | 1.29E-06 |
| ENSG00000180914 | OXTR | Oxytocin receptor | 1.29E-06 |
| ENSG00000156136 | DCK | Deoxycytidine kinase | 1.32E-06 |
| ENSG00000127586 | CHTF18 | Chromosome transmission fidelity factor 18 | 1.44E-06 |
| ENSG00000005189 | REXO5 | RNA exonuclease 5 | 1.52E-06 |
| ENSG00000120334 | CENPL | Centromere protein L | 1.58E-06 |
| ENSG00000125450 | NUP85 | Nucleoporin 85 | 1.79E-06 |
| ENSG00000171793 | CTPS1 | CTP synthase 1 | 1.80E-06 |
| ENSG00000171791 | BCL2 | BCL2 apoptosis regulator | 2.08E-06 |

| | | | |
|---|---|---|---|
| ENSG00000213853 | EMP2 | Epithelial membrane protein 2 | 2.15E-06 |
| ENSG00000105974 | CAV1 | Caveolin 1 | 2.16E-06 |
| ENSG00000183287 | CCBE1 | Collagen and calcium binding EGF domains 1 | 2.34E-06 |
| ENSG00000129534 | MIS18BP1 | MIS18 binding protein 1 | 2.36E-06 |
| ENSG00000103995 | CEP152 | Centrosomal protein 152 | 2.41E-06 |
| ENSG00000129038 | LOXL1 | Lysyl oxidase like 1 | 2.49E-06 |
| ENSG00000127863 | TNFRSF19 | TNF receptor superfamily member 19 | 2.72E-06 |
| ENSG00000149929 | HIRIP3 | HIRA interacting protein 3 | 2.72E-06 |
| ENSG00000137054 | POLR1E | RNA polymerase I subunit E | 2.94E-06 |
| ENSG00000025770 | NCAPH2 | Non-SMC condensin II complex subunit H2 | 2.98E-06 |
| ENSG00000275126 | H4C13 | H4 clustered histone 13 | 3.15E-06 |
| ENSG00000172687 | ZNF738 | Zinc finger protein 738 | 3.41E-06 |
| ENSG00000134986 | NREP | Neuronal regeneration related protein | 3.42E-06 |
| ENSG00000145861 | C1QTNF2 | C1q and TNF related 2 | 4.42E-06 |
| ENSG00000197275 | RAD54B | RAD54 homolog B | 4.72E-06 |
| ENSG00000100504 | PYGL | Glycogen phosphorylase L | 4.74E-06 |
| ENSG00000115875 | SRSF7 | Serine and arginine rich splicing factor 7 | 4.90E-06 |
| ENSG00000197771 | MCMBP | Minichromosome maintenance complex binding protein | 5.79E-06 |
| ENSG00000146143 | PRIM2 | DNA primase subunit 2 | 5.84E-06 |
| ENSG00000080839 | RBL1 | RB transcriptional corepressor like 1 | 6.04E-06 |
| ENSG00000165501 | LRR1 | Leucine rich repeat protein 1 | 6.04E-06 |
| ENSG00000113569 | NUP155 | Nucleoporin 155 | 6.15E-06 |
| ENSG00000165891 | E2F7 | E2F transcription factor 7 | 7.59E-06 |
| ENSG00000111581 | NUP107 | Nucleoporin 107 | 7.86E-06 |
| ENSG00000125485 | DDX31 | DEAD-box helicase 31 | 8.25E-06 |
| ENSG00000000460 | C1orf112 | Chromosome 1 open reading frame 112 | 1.05E-05 |
| ENSG00000154839 | SKA1 | Spindle and kinetochore associated complex subunit 1 | 1.07E-05 |
| ENSG00000115159 | GPD2 | Glycerol-3-phosphate dehydrogenase 2 | 1.10E-05 |
| ENSG00000106628 | POLD2 | DNA polymerase delta 2, accessory subunit | 1.11E-05 |
| ENSG00000197472 | ZNF695 | Zinc finger protein 695 | 1.14E-05 |
| ENSG00000070950 | RAD18 | RAD18 E3 ubiquitin protein ligase | 1.14E-05 |
| ENSG00000164687 | FABP5 | Fatty acid binding protein 5 | 1.19E-05 |
| ENSG00000168405 | CMAHP | Cytidine monophospho-N-acetylneuraminic acid hydroxylase, pseudogene | 1.20E-05 |
| ENSG00000166881 | NEMP1 | Nuclear envelope integral membrane protein 1 | 1.20E-05 |
| ENSG00000284491 | THSD8 | Thrombospondin type 1 domain containing 8 | 1.22E-05 |
| ENSG00000120437 | ACAT2 | Acetyl-coa acetyltransferase 2 | 1.31E-05 |
| ENSG00000213024 | NUP62 | Nucleoporin 62 | 1.31E-05 |
| ENSG00000123136 | DDX39A | Dexd-box helicase 39A | 1.34E-05 |
| ENSG00000148229 | POLE3 | DNA polymerase epsilon 3, accessory subunit | 1.36E-05 |
| ENSG00000163781 | TOPBP1 | DNA topoisomerase II binding protein 1 | 1.56E-05 |
| ENSG00000136824 | SMC2 | Structural maintenance of chromosomes 2 | 1.66E-05 |
| ENSG00000170017 | ALCAM | Activated leukocyte cell adhesion molecule | 1.95E-05 |
| ENSG00000173894 | CBX2 | Chromobox 2 | 1.95E-05 |
| ENSG00000099194 | SCD | Stearoyl-coa desaturase | 1.95E-05 |
| ENSG00000132341 | RAN | RAN, member RAS oncogene family | 1.95E-05 |
| ENSG00000181751 | C5orf30 | Chromosome 5 open reading frame 30 | 1.95E-05 |
| ENSG00000040275 | SPDL1 | Spindle apparatus coiled-coil protein 1 | 1.96E-05 |
| ENSG00000134775 | FHOD3 | Formin homology 2 domain containing 3 | 1.97E-05 |
| ENSG00000123975 | CKS2 | CDC28 protein kinase regulatory subunit 2 | 2.26E-05 |
| ENSG00000031691 | CENPQ | Centromere protein Q | 2.27E-05 |

| | | | |
|---|---|---|---|
| ENSG00000132646 | PCNA | Proliferating cell nuclear antigen | 2.40E-05 |
| ENSG00000149136 | SSRP1 | Structure specific recognition protein 1 | 2.40E-05 |
| ENSG00000197451 | HNRNPAB | Heterogeneous nuclear ribonucleoprotein A/B | 2.40E-05 |
| ENSG00000175183 | CSRP2 | Cysteine and glycine rich protein 2 | 2.48E-05 |
| ENSG00000132661 | NXT1 | Nuclear transport factor 2 like export factor 1 | 2.94E-05 |
| ENSG00000183496 | MEX3B | Mex-3 RNA binding family member B | 3.01E-05 |
| ENSG00000104356 | POP1 | POP1 homolog, ribonuclease P/MRP subunit | 3.05E-05 |
| ENSG00000116774 | OLFML3 | Olfactomedin like 3 | 3.18E-05 |
| ENSG00000184162 | NR2C2AP | Nuclear receptor 2C2 associated protein | 3.29E-05 |
| ENSG00000143179 | UCK2 | Uridine-cytidine kinase 2 | 3.74E-05 |
| ENSG00000135549 | PKIB | Camp-dependent protein kinase inhibitor beta | 3.79E-05 |
| ENSG00000135045 | C9orf40 | Chromosome 9 open reading frame 40 | 3.81E-05 |
| ENSG00000136518 | ACTL6A | Actin like 6A | 3.95E-05 |
| ENSG00000119285 | HEATR1 | HEAT repeat containing 1 | 4.09E-05 |
| ENSG00000112081 | SRSF3 | Serine and arginine rich splicing factor 3 | 4.67E-05 |
| ENSG00000072571 | HMMR | Hyaluronan mediated motility receptor | 4.71E-05 |
| ENSG00000074800 | ENO1 | Enolase 1 | 4.82E-05 |
| ENSG00000115648 | MLPH | Melanophilin | 5.09E-05 |
| ENSG00000103495 | MAZ | MYC associated zinc finger protein | 5.27E-05 |
| ENSG00000164125 | GASK1B | Golgi associated kinase 1B | 5.68E-05 |
| ENSG00000160352 | ZNF714 | Zinc finger protein 714 | 5.72E-05 |
| ENSG00000124207 | CSE1L | Chromosome segregation 1 like | 5.80E-05 |
| ENSG00000120158 | RCL1 | RNA terminal phosphate cyclase like 1 | 5.94E-05 |
| ENSG00000116679 | IVNS1ABP | Influenza virus NS1A binding protein | 6.04E-05 |
| ENSG00000184897 | H1-10 | H1.10 linker histone | 6.18E-05 |
| ENSG00000176208 | ATAD5 | Atpase family AAA domain containing 5 | 6.32E-05 |
| ENSG00000162909 | CAPN2 | Calpain 2 | 6.46E-05 |
| ENSG00000076770 | MBNL3 | Muscleblind like splicing regulator 3 | 7.56E-05 |
| ENSG00000127337 | YEATS4 | YEATS domain containing 4 | 8.03E-05 |
| ENSG00000101911 | PRPS2 | Phosphoribosyl pyrophosphate synthetase 2 | 8.10E-05 |
| ENSG00000158427 | TMSB15B | Thymosin beta 15B | 8.10E-05 |
| ENSG00000143815 | LBR | Lamin B receptor | 8.19E-05 |
| ENSG00000168672 | LRATD2 | LRAT domain containing 2 | 8.58E-05 |
| ENSG00000170540 | ARL6IP1 | ADP ribosylation factor like gtpase 6 interacting protein 1 | 9.16E-05 |
| ENSG00000249115 | HAUS5 | HAUS augmin like complex subunit 5 | 9.19E-05 |
| ENSG00000134291 | TMEM106C | Transmembrane protein 106C | 9.68E-05 |
| ENSG00000164105 | SAP30 | Sin3A associated protein 30 | 9.76E-05 |
| ENSG00000058804 | NDC1 | NDC1 transmembrane nucleoporin | 9.97E-05 |
| ENSG00000156876 | SASS6 | SAS-6 centriolar assembly protein | 1.00E-04 |
| ENSG00000276966 | H4C5 | H4 clustered histone 5 | 0.000109 |
| ENSG00000125319 | HROB | Homologous recombination factor with OB-fold | 0.000119 |
| ENSG00000185760 | KCNQ5 | Potassium voltage-gated channel subfamily Q member 5 | 0.00012 |
| ENSG00000029993 | HMGB3 | High mobility group box 3 | 0.000128 |
| ENSG00000122870 | BICC1 | Bicc family RNA binding protein 1 | 0.000143 |
| ENSG00000125384 | PTGER2 | Prostaglandin E receptor 2 | 0.00015 |
| ENSG00000053372 | MRTO4 | MRT4 homolog, ribosome maturation factor | 0.000162 |
| ENSG00000120658 | ENOX1 | Ecto-NOX disulfide-thiol exchanger 1 | 0.000179 |
| ENSG00000079462 | PAFAH1B3 | Platelet activating factor acetylhydrolase 1b catalytic subunit 3 | 0.000181 |
| ENSG00000117519 | CNN3 | Calponin 3 | 0.000191 |
| ENSG00000114999 | TTL | Tubulin tyrosine ligase | 0.000191 |

| | | | |
|---|---|---|---|
| ENSG00000115380 | EFEMP1 | EGF containing fibulin extracellular matrix protein 1 | 0.000193 |
| ENSG00000072041 | SLC6A15 | Solute carrier family 6 member 15 | 0.000196 |
| ENSG00000061337 | LZTS1 | Leucine zipper tumor suppressor 1 | 0.000196 |
| ENSG00000126453 | BCL2L12 | BCL2 like 12 | 0.000197 |
| ENSG00000012963 | UBR7 | Ubiquitin protein ligase E3 component n-recognin 7 (putative) | 0.000201 |
| ENSG00000100526 | CDKN3 | Cyclin dependent kinase inhibitor 3 | 0.000204 |
| ENSG00000169258 | GPRIN1 | G protein regulated inducer of neurite outgrowth 1 | 0.000204 |
| ENSG00000147202 | DIAPH2 | Diaphanous related formin 2 | 0.00021 |
| ENSG00000184635 | ZNF93 | Zinc finger protein 93 | 0.000221 |
| ENSG00000196230 | TUBB | Tubulin beta class I | 0.000234 |
| ENSG00000170264 | FAM161A | FAM161 centrosomal protein A | 0.000257 |
| ENSG00000137814 | HAUS2 | HAUS augmin like complex subunit 2 | 0.00026 |
| ENSG00000130816 | DNMT1 | DNA methyltransferase 1 | 0.00027 |
| ENSG00000155858 | LSM11 | LSM11, U7 small nuclear RNA associated | 0.000283 |
| ENSG00000136527 | TRA2B | Transformer 2 beta homolog | 0.000285 |
| ENSG00000071575 | TRIB2 | Tribbles pseudokinase 2 | 0.000285 |
| ENSG00000198830 | HMGN2 | High mobility group nucleosomal binding domain 2 | 0.000288 |
| ENSG00000146281 | PM20D2 | Peptidase M20 domain containing 2 | 0.000291 |
| ENSG00000176894 | PXMP2 | Peroxisomal membrane protein 2 | 0.000299 |
| ENSG00000136450 | SRSF1 | Serine and arginine rich splicing factor 1 | 0.000303 |
| ENSG00000151376 | ME3 | Malic enzyme 3 | 0.000304 |
| ENSG00000151287 | TEX30 | Testis expressed 30 | 0.000309 |
| ENSG00000242114 | MTFP1 | Mitochondrial fission process 1 | 0.000313 |
| ENSG00000120217 | CD274 | CD274 molecule | 0.00032 |
| ENSG00000135446 | CDK4 | Cyclin dependent kinase 4 | 0.000324 |
| ENSG00000154760 | SLFN13 | Schlafen family member 13 | 0.000337 |
| ENSG00000170515 | PA2G4 | Proliferation-associated 2G4 | 0.00036 |
| ENSG00000159131 | GART | Phosphoribosylglycinamide formyltransferase, phosphoribosylglycinamide synthetase, phosphoribosylaminoimidazole synthetase | 0.000381 |
| ENSG00000177917 | ARL6IP6 | ADP ribosylation factor like gtpase 6 interacting protein 6 | 0.000387 |
| ENSG00000141052 | MYOCD | Myocardin | 0.000387 |
| ENSG00000181544 | FANCB | FA complementation group B | 0.000418 |
| ENSG00000149485 | FADS1 | Fatty acid desaturase 1 | 0.00043 |
| ENSG00000116830 | TTF2 | Transcription termination factor 2 | 0.000436 |
| ENSG00000180730 | SHISA2 | Shisa family member 2 | 0.000456 |
| ENSG00000196363 | WDR5 | WD repeat domain 5 | 0.000456 |
| ENSG00000073536 | NLE1 | Notchless homolog 1 | 0.000463 |
| ENSG00000183527 | PSMG1 | Proteasome assembly chaperone 1 | 0.0005 |
| ENSG00000164985 | PSIP1 | PC4 and SFRS1 interacting protein 1 | 0.000503 |
| ENSG00000139278 | GLIPR1 | GLI pathogenesis related 1 | 0.000507 |
| ENSG00000163535 | SGO2 | Shugoshin 2 | 0.000521 |
| ENSG00000104626 | ERI1 | Exoribonuclease 1 | 0.000524 |
| ENSG00000114346 | ECT2 | Epithelial cell transforming 2 | 0.000524 |
| ENSG00000065150 | IPO5 | Importin 5 | 0.000527 |
| ENSG00000196456 | ZNF775 | Zinc finger protein 775 | 0.000547 |
| ENSG00000213160 | KLHL23 | Kelch like family member 23 | 0.000558 |
| ENSG00000156466 | GDF6 | Growth differentiation factor 6 | 0.00057 |
| ENSG00000241697 | TMEFF1 | Transmembrane protein with EGF like and two follistatin like domains 1 | 0.000589 |

| | | | |
|---|---|---|---|
| ENSG00000011304 | PTBP1 | Polypyrimidine tract binding protein 1 | 0.0006 |
| ENSG00000244306 | AL589743.1 | Double homeobox A pseudogene 10 | 0.000628 |
| ENSG00000071564 | TCF3 | Transcription factor 3 | 0.000661 |
| ENSG00000243444 | | | 0.000668 |
| ENSG00000155561 | NUP205 | Nucleoporin 205 | 0.000671 |
| ENSG00000012048 | BRCA1 | BRCA1 DNA repair associated | 0.000727 |
| ENSG00000184661 | CDCA2 | Cell division cycle associated 2 | 0.000727 |
| ENSG00000029153 | ARNTL2 | Aryl hydrocarbon receptor nuclear translocator like 2 | 0.000746 |
| ENSG00000162419 | GMEB1 | Glucocorticoid modulatory element binding protein 1 | 0.000801 |
| ENSG00000128059 | PPAT | Phosphoribosyl pyrophosphate amidotransferase | 0.000862 |
| ENSG00000138346 | DNA2 | DNA replication helicase/nuclease 2 | 0.000869 |
| ENSG00000184009 | ACTG1 | Actin gamma 1 | 0.000872 |
| ENSG00000077684 | JADE1 | Jade family PHD finger 1 | 0.000926 |
| ENSG00000151503 | NCAPD3 | Non-SMC condensin II complex subunit D3 | 0.000928 |
| ENSG00000150687 | PRSS23 | Serine protease 23 | 0.000939 |
| ENSG00000106268 | NUDT1 | Nudix hydrolase 1 | 0.000995 |
| ENSG00000132780 | NASP | Nuclear autoantigenic sperm protein | 0.001004 |
| ENSG00000183763 | TRAIP | TRAF interacting protein | 0.001006 |
| ENSG00000116062 | MSH6 | Muts homolog 6 | 0.001032 |
| ENSG00000159176 | CSRP1 | Cysteine and glycine rich protein 1 | 0.001037 |
| ENSG00000143493 | INTS7 | Integrator complex subunit 7 | 0.001083 |
| ENSG00000044524 | EPHA3 | EPH receptor A3 | 0.001098 |
| ENSG00000169813 | HNRNPF | Heterogeneous nuclear ribonucleoprotein F | 0.001139 |
| ENSG00000118513 | MYB | MYB proto-oncogene, transcription factor | 0.001146 |
| ENSG00000145681 | HAPLN1 | Hyaluronan and proteoglycan link protein 1 | 0.001152 |
| ENSG00000075624 | ACTB | Actin beta | 0.001168 |
| ENSG00000147955 | SIGMAR1 | Sigma non-opioid intracellular receptor 1 | 0.001168 |
| ENSG00000165724 | ZMYND19 | Zinc finger MYND-type containing 19 | 0.001176 |
| ENSG00000130826 | DKC1 | Dyskerin pseudouridine synthase 1 | 0.001241 |
| ENSG00000119326 | CTNNAL1 | Catenin alpha like 1 | 0.001243 |
| ENSG00000181201 | H2BU2P | H2B.U histone 2, pseudogene | 0.001268 |
| ENSG00000265107 | GJA5 | Gap junction protein alpha 5 | 0.00127 |
| ENSG00000135245 | HILPDA | Hypoxia inducible lipid droplet associated | 0.001276 |
| ENSG00000085415 | SEH1L | SEH1 like nucleoporin | 0.001282 |
| ENSG00000162849 | KIF26B | Kinesin family member 26B | 0.001282 |
| ENSG00000111450 | STX2 | Syntaxin 2 | 0.001361 |
| ENSG00000198885 | ITPRIPL1 | ITPRIP like 1 | 0.001386 |
| ENSG00000120699 | EXOSC8 | Exosome component 8 | 0.001492 |
| ENSG00000128408 | RIBC2 | RIB43A domain with coiled-coils 2 | 0.001537 |
| ENSG00000186777 | ZNF732 | Zinc finger protein 732 | 0.001561 |
| ENSG00000083720 | OXCT1 | 3-oxoacid coa-transferase 1 | 0.001587 |
| ENSG00000154734 | ADAMTS1 | ADAM metallopeptidase with thrombospondin type 1 motif 1 | 0.0016 |
| ENSG00000170468 | RIOX1 | Ribosomal oxygenase 1 | 0.001616 |
| ENSG00000187840 | EIF4EBP1 | Eukaryotic translation initiation factor 4E binding protein 1 | 0.001625 |
| ENSG00000147274 | RBMX | RNA binding motif protein X-linked | 0.001676 |
| ENSG00000183684 | ALYREF | Aly/REF export factor | 0.001693 |
| ENSG00000140416 | TPM1 | Tropomyosin 1 | 0.001764 |
| ENSG00000215784 | FAM72D | Family with sequence similarity 72 member D | 0.00178 |
| ENSG00000136699 | SMPD4 | Sphingomyelin phosphodiesterase 4 | 0.001815 |

| | | | |
|---|---|---|---|
| ENSG00000139734 | DIAPH3 | Diaphanous related formin 3 | 0.001838 |
| ENSG00000155755 | TMEM237 | Transmembrane protein 237 | 0.001847 |
| ENSG00000075702 | WDR62 | WD repeat domain 62 | 0.001889 |
| ENSG00000152240 | HAUS1 | HAUS augmin like complex subunit 1 | 0.001901 |
| ENSG00000118596 | SLC16A7 | Solute carrier family 16 member 7 | 0.001906 |
| ENSG00000178202 | POGLUT3 | Protein O-glucosyltransferase 3 | 0.001923 |
| ENSG00000185347 | TEDC1 | Tubulin epsilon and delta complex 1 | 0.001923 |
| ENSG00000144681 | STAC | SH3 and cysteine rich domain | 0.001961 |
| ENSG00000108055 | SMC3 | Structural maintenance of chromosomes 3 | 0.002061 |
| ENSG00000160752 | FDPS | Farnesyl diphosphate synthase | 0.002073 |
| ENSG00000145545 | SRD5A1 | Steroid 5 alpha-reductase 1 | 0.002081 |
| ENSG00000283559 | AC139491.6 | Centrosomal protein 192kda (CEP192) pseudogene | 0.002103 |
| ENSG00000188312 | CENPP | Centromere protein P | 0.002104 |
| ENSG00000085276 | MECOM | MDS1 and EVI1 complex locus | 0.002104 |
| ENSG00000187193 | MT1X | Metallothionein 1X | 0.002138 |
| ENSG00000196890 | H2BU1 | H2B.U histone 1 | 0.002277 |
| ENSG00000145675 | PIK3R1 | Phosphoinositide-3-kinase regulatory subunit 1 | 0.002405 |
| ENSG00000116120 | FARSB | Phenylalanyl-trna synthetase subunit beta | 0.00243 |
| ENSG00000102098 | SCML2 | Scm polycomb group protein like 2 | 0.002441 |
| ENSG00000154646 | TMPRSS15 | Transmembrane serine protease 15 | 0.002478 |
| ENSG00000138741 | TRPC3 | Transient receptor potential cation channel subfamily C member 3 | 0.002539 |
| ENSG00000179409 | GEMIN4 | Gem nuclear organelle associated protein 4 | 0.002549 |
| ENSG00000120800 | UTP20 | UTP20 small subunit processome component | 0.002564 |
| ENSG00000144283 | PKP4 | Plakophilin 4 | 0.002606 |
| ENSG00000164002 | EXO5 | Exonuclease 5 | 0.002673 |
| ENSG00000136875 | PRPF4 | Pre-mrna processing factor 4 | 0.00274 |
| ENSG00000172336 | POP7 | POP7 homolog, ribonuclease P/MRP subunit | 0.002743 |
| ENSG00000108179 | PPIF | Peptidylprolyl isomerase F | 0.002788 |
| ENSG00000204899 | MZT1 | Mitotic spindle organizing protein 1 | 0.002791 |
| ENSG00000102172 | SMS | Spermine synthase | 0.002791 |
| ENSG00000160193 | WDR4 | WD repeat domain 4 | 0.002804 |
| ENSG00000099256 | PRTFDC1 | Phosphoribosyl transferase domain containing 1 | 0.002851 |
| ENSG00000117597 | UTP25 | UTP25 small subunit processor component | 0.002851 |
| ENSG00000277443 | MARCKS | Myristoylated alanine rich protein kinase C substrate | 0.002864 |
| ENSG00000101546 | RBFA | Ribosome binding factor A | 0.002874 |
| ENSG00000172927 | MYEOV | Myeloma overexpressed | 0.002986 |
| ENSG00000100304 | TTLL12 | Tubulin tyrosine ligase like 12 | 0.002989 |
| ENSG00000149548 | CCDC15 | Coiled-coil domain containing 15 | 0.003006 |
| ENSG00000261236 | BOP1 | BOP1 ribosomal biogenesis factor | 0.003065 |
| ENSG00000103540 | CCP110 | Centriolar coiled-coil protein 110 | 0.003113 |
| ENSG00000112658 | SRF | Serum response factor | 0.003113 |
| ENSG00000243955 | GSTA1 | Glutathione S-transferase alpha 1 | 0.003113 |
| ENSG00000178695 | KCTD12 | Potassium channel tetramerization domain containing 12 | 0.003123 |
| ENSG00000115232 | ITGA4 | Integrin subunit alpha 4 | 0.003142 |
| ENSG00000248710 | AC079594.2 | TRIM59 and ift80 readthrough | 0.003189 |
| ENSG00000119403 | PHF19 | PHD finger protein 19 | 0.003195 |
| ENSG00000096060 | FKBP5 | FKBP prolyl isomerase 5 | 0.003342 |
| ENSG00000203668 | CHML | CHM like Rab escort protein | 0.003471 |
| ENSG00000111057 | KRT18 | Keratin 18 | 0.0035 |
| ENSG00000134758 | RNF138 | Ring finger protein 138 | 0.003524 |

| | | | |
|---|---|---|---|
| ENSG00000141668 | CBLN2 | Cerebellin 2 precursor | 0.003524 |
| ENSG00000134824 | FADS2 | Fatty acid desaturase 2 | 0.003548 |
| ENSG00000107104 | KANK1 | KN motif and ankyrin repeat domains 1 | 0.003577 |
| ENSG00000135362 | PRR5L | Proline rich 5 like | 0.003625 |
| ENSG00000143157 | POGK | Pogo transposable element derived with KRAB domain | 0.003625 |
| ENSG00000189212 | DPY19L2P1 | DPY19L2 pseudogene 1 | 0.003625 |
| ENSG00000132669 | RIN2 | Ras and Rab interactor 2 | 0.003653 |
| ENSG00000005059 | MCUB | Mitochondrial calcium uniporter dominant negative beta subunit | 0.00371 |
| ENSG00000092201 | SUPT16H | SPT16 homolog, facilitates chromatin remodeling subunit | 0.00371 |
| ENSG00000256229 | ZNF486 | Zinc finger protein 486 | 0.003799 |
| ENSG00000154473 | BUB3 | BUB3 mitotic checkpoint protein | 0.00386 |
| ENSG00000082516 | GEMIN5 | Gem nuclear organelle associated protein 5 | 0.003888 |
| ENSG00000092964 | DPYSL2 | Dihydropyrimidinase like 2 | 0.003938 |
| ENSG00000217555 | CKLF | Chemokine like factor | 0.003966 |
| ENSG00000082512 | TRAF5 | TNF receptor associated factor 5 | 0.004092 |
| ENSG00000106144 | CASP2 | Caspase 2 | 0.004163 |
| ENSG00000136261 | BZW2 | Basic leucine zipper and W2 domains 2 | 0.004163 |
| ENSG00000101407 | TTI1 | TELO2 interacting protein 1 | 0.004374 |
| ENSG00000115461 | IGFBP5 | Insulin like growth factor binding protein 5 | 0.004374 |
| ENSG00000170153 | RNF150 | Ring finger protein 150 | 0.004395 |
| ENSG00000188807 | TMEM201 | Transmembrane protein 201 | 0.004395 |
| ENSG00000164284 | GRPEL2 | Grpe like 2, mitochondrial | 0.004448 |
| ENSG00000075131 | TIPIN | TIMELESS interacting protein | 0.004618 |
| ENSG00000130935 | NOL11 | Nucleolar protein 11 | 0.004644 |
| ENSG00000112039 | FANCE | FA complementation group E | 0.004664 |
| ENSG00000107566 | ERLIN1 | ER lipid raft associated 1 | 0.004817 |
| ENSG00000113460 | BRIX1 | Biogenesis of ribosomes BRX1 | 0.004964 |
| ENSG00000122644 | ARL4A | ADP ribosylation factor like gtpase 4A | 0.004964 |
| ENSG00000116237 | ICMT | Isoprenylcysteine carboxyl methyltransferase | 0.00499 |
| ENSG00000161647 | MPP3 | Membrane palmitoylated protein 3 | 0.005072 |
| ENSG00000198912 | C1orf174 | Chromosome 1 open reading frame 174 | 0.005313 |
| ENSG00000091527 | CDV3 | CDV3 homolog | 0.005338 |
| ENSG00000186522 | SEPTIN10 | Septin 10 | 0.005419 |
| ENSG00000161996 | WDR90 | WD repeat domain 90 | 0.005475 |
| ENSG00000134285 | FKBP11 | FKBP prolyl isomerase 11 | 0.005555 |
| ENSG00000138587 | MNS1 | Meiosis specific nuclear structural 1 | 0.005555 |
| ENSG00000204291 | COL15A1 | Collagen type XV alpha 1 chain | 0.00558 |
| ENSG00000231205 | ZNF826P | Zinc finger protein 826, pseudogene | 0.005636 |
| ENSG00000163029 | SMC6 | Structural maintenance of chromosomes 6 | 0.00584 |
| ENSG00000255561 | FDXACB1 | Ferredoxin-fold anticodon binding domain containing 1 | 0.00584 |
| ENSG00000105185 | PDCD5 | Programmed cell death 5 | 0.005919 |
| ENSG00000171492 | LRRC8D | Leucine rich repeat containing 8 VRAC subunit D | 0.006052 |
| ENSG00000150556 | LYPD6B | LY6/PLAUR domain containing 6B | 0.006107 |
| ENSG00000093217 | XYLB | Xylulokinase | 0.006208 |
| ENSG00000167088 | SNRPD1 | Small nuclear ribonucleoprotein D1 polypeptide | 0.006254 |
| ENSG00000184216 | IRAK1 | Interleukin 1 receptor associated kinase 1 | 0.006278 |
| ENSG00000105202 | FBL | Fibrillarin | 0.006293 |
| ENSG00000175175 | PPM1E | Protein phosphatase, Mg2+/Mn2+ dependent 1E | 0.006293 |
| ENSG00000167553 | TUBA1C | Tubulin alpha 1c | 0.006377 |
| ENSG00000151892 | GFRA1 | GDNF family receptor alpha 1 | 0.0064 |

| | | | |
|---|---|---|---|
| ENSG00000105894 | PTN | Pleiotrophin | 0.00656 |
| ENSG00000146477 | SLC22A3 | Solute carrier family 22 member 3 | 0.006666 |
| ENSG00000091986 | CCDC80 | Coiled-coil domain containing 80 | 0.006688 |
| ENSG00000156471 | PTDSS1 | Phosphatidylserine synthase 1 | 0.006771 |
| ENSG00000258555 | SPECC1L-ADORA2A | SPECC1L-ADORA2A readthrough (NMD candidate) | 0.006793 |
| ENSG00000054277 | OPN3 | Opsin 3 | 0.006989 |
| ENSG00000133107 | TRPC4 | Transient receptor potential cation channel subfamily C member 4 | 0.007235 |
| ENSG00000168077 | SCARA3 | Scavenger receptor class A member 3 | 0.007338 |
| ENSG00000109576 | AADAT | Aminoadipate aminotransferase | 0.007351 |
| ENSG00000121057 | AKAP1 | A-kinase anchoring protein 1 | 0.007351 |
| ENSG00000173457 | PPP1R14B | Protein phosphatase 1 regulatory inhibitor subunit 14B | 0.007504 |
| ENSG00000116161 | CACYBP | Calcyclin binding protein | 0.007674 |
| ENSG00000183048 | SLC25A10 | Solute carrier family 25 member 10 | 0.007754 |
| ENSG00000179104 | TMTC2 | Transmembrane O-mannosyltransferase targeting cadherins 2 | 0.007894 |
| ENSG00000163923 | RPL39L | Ribosomal protein L39 like | 0.008054 |
| ENSG00000254726 | MEX3A | Mex-3 RNA binding family member A | 0.008054 |
| ENSG00000150753 | CCT5 | Chaperonin containing TCP1 subunit 5 | 0.008233 |
| ENSG00000143401 | ANP32E | Acidic nuclear phosphoprotein 32 family member E | 0.008518 |
| ENSG00000080200 | CRYBG3 | Crystallin beta-gamma domain containing 3 | 0.008587 |
| ENSG00000102007 | PLP2 | Proteolipid protein 2 | 0.00885 |
| ENSG00000113657 | DPYSL3 | Dihydropyrimidinase like 3 | 0.008975 |
| ENSG00000094916 | CBX5 | Chromobox 5 | 0.009082 |
| ENSG00000143942 | CHAC2 | Chac cation transport regulator homolog 2 | 0.009159 |
| ENSG00000182963 | GJC1 | Gap junction protein gamma 1 | 0.009177 |
| ENSG00000109881 | CCDC34 | Coiled-coil domain containing 34 | 0.009225 |
| ENSG00000141560 | FN3KRP | Fructosamine 3 kinase related protein | 0.009407 |
| ENSG00000206190 | ATP10A | Atpase phospholipid transporting 10A (putative) | 0.009454 |
| ENSG00000141401 | IMPA2 | Inositol monophosphatase 2 | 0.009472 |
| ENSG00000134802 | SLC43A3 | Solute carrier family 43 member 3 | 0.009642 |
| ENSG00000160229 | ZNF66 | Zinc finger protein 66 | 0.009775 |
| ENSG00000111186 | WNT5B | Wnt family member 5B | 0.009908 |
| ENSG00000089280 | FUS | FUS RNA binding protein | 0.010213 |
| ENSG00000141569 | TRIM65 | Tripartite motif containing 65 | 0.010304 |
| ENSG00000151576 | QTRT2 | Queuine trna-ribosyltransferase accessory subunit 2 | 0.010682 |
| ENSG00000152455 | SUV39H2 | Suppressor of variegation 3-9 homolog 2 | 0.010727 |
| ENSG00000079156 | OSBPL6 | Oxysterol binding protein like 6 | 0.010743 |
| ENSG00000132773 | TOE1 | Target of EGR1, exonuclease | 0.011007 |
| ENSG00000113140 | SPARC | Secreted protein acidic and cysteine rich | 0.011153 |
| ENSG00000011260 | UTP18 | UTP18 small subunit processome component | 0.011169 |
| ENSG00000110108 | TMEM109 | Transmembrane protein 109 | 0.011329 |
| ENSG00000152503 | TRIM36 | Tripartite motif containing 36 | 0.011373 |
| ENSG00000145416 | MARCHF1 | Membrane associated ring-CH-type finger 1 | 0.011388 |
| ENSG00000133019 | CHRM3 | Cholinergic receptor muscarinic 3 | 0.011544 |
| ENSG00000145990 | GFOD1 | Glucose-fructose oxidoreductase domain containing 1 | 0.011559 |
| ENSG00000137337 | MDC1 | Mediator of DNA damage checkpoint 1 | 0.011674 |
| ENSG00000163002 | NUP35 | Nucleoporin 35 | 0.011674 |
| ENSG00000160208 | RRP1B | Ribosomal RNA processing 1B | 0.011676 |
| ENSG00000068028 | RASSF1 | Ras association domain family member 1 | 0.011833 |

| | | | |
|---|---|---|---|
| ENSG00000109099 | PMP22 | Peripheral myelin protein 22 | 0.011833 |
| ENSG00000127418 | FGFRL1 | Fibroblast growth factor receptor like 1 | 0.011862 |
| ENSG00000136111 | TBC1D4 | TBC1 domain family member 4 | 0.011862 |
| ENSG00000163006 | CCDC138 | Coiled-coil domain containing 138 | 0.01196 |
| ENSG00000104064 | GABPB1 | GA binding protein transcription factor subunit beta 1 | 0.011962 |
| ENSG00000214706 | IFRD2 | Interferon related developmental regulator 2 | 0.011962 |
| ENSG00000145934 | TENM2 | Teneurin transmembrane protein 2 | 0.012074 |
| ENSG00000196950 | SLC39A10 | Solute carrier family 39 member 10 | 0.012116 |
| ENSG00000145220 | LYAR | Ly1 antibody reactive | 0.01242 |
| ENSG00000123505 | AMD1 | Adenosylmethionine decarboxylase 1 | 0.0126 |
| ENSG00000170876 | TMEM43 | Transmembrane protein 43 | 0.012834 |
| ENSG00000035928 | RFC1 | Replication factor C subunit 1 | 0.012847 |
| ENSG00000157240 | FZD1 | Frizzled class receptor 1 | 0.012983 |
| ENSG00000188610 | FAM72B | Family with sequence similarity 72 member B | 0.013051 |
| ENSG00000135312 | HTR1B | 5-hydroxytryptamine receptor 1B | 0.013064 |
| ENSG00000144891 | AGTR1 | Angiotensin II receptor type 1 | 0.013091 |
| ENSG00000166333 | ILK | Integrin linked kinase | 0.013091 |
| ENSG00000184992 | BRI3BP | BRI3 binding protein | 0.013144 |
| ENSG00000173848 | NET1 | Neuroepithelial cell transforming 1 | 0.013441 |
| ENSG00000197646 | PDCD1LG2 | Programmed cell death 1 ligand 2 | 0.01359 |
| ENSG00000079387 | SENP1 | SUMO specific peptidase 1 | 0.014068 |
| ENSG00000180611 | MB21D2 | Mab-21 domain containing 2 | 0.014119 |
| ENSG00000144824 | PHLDB2 | Pleckstrin homology like domain family B member 2 | 0.014158 |
| ENSG00000120256 | LRP11 | LDL receptor related protein 11 | 0.014384 |
| ENSG00000162433 | AK4 | Adenylate kinase 4 | 0.014434 |
| ENSG00000135480 | KRT7 | Keratin 7 | 0.014483 |
| ENSG00000254093 | PINX1 | PIN2 (TERF1) interacting telomerase inhibitor 1 | 0.014548 |
| ENSG00000129484 | PARP2 | Poly(ADP-ribose) polymerase 2 | 0.014768 |
| ENSG00000188641 | DPYD | Dihydropyrimidine dehydrogenase | 0.014976 |
| ENSG00000185298 | CCDC137 | Coiled-coil domain containing 137 | 0.015262 |
| ENSG00000187231 | SESTD1 | SEC14 and spectrin domain containing 1 | 0.015309 |
| ENSG00000198478 | SH3BGRL2 | SH3 domain binding glutamate rich protein like 2 | 0.015309 |
| ENSG00000099783 | HNRNPM | Heterogeneous nuclear ribonucleoprotein M | 0.015399 |
| ENSG00000205659 | LIN52 | Lin-52 DREAM muvb core complex component | 0.015436 |
| ENSG00000119900 | OGFRL1 | Opioid growth factor receptor like 1 | 0.015657 |
| ENSG00000153993 | SEMA3D | Semaphorin 3D | 0.016038 |
| ENSG00000130204 | TOMM40 | Translocase of outer mitochondrial membrane 40 | 0.016145 |
| ENSG00000206053 | JPT2 | Jupiter microtubule associated homolog 2 | 0.016337 |
| ENSG00000117394 | SLC2A1 | Solute carrier family 2 member 1 | 0.016616 |
| ENSG00000188529 | SRSF10 | Serine and arginine rich splicing factor 10 | 0.016616 |
| ENSG00000183722 | LHFPL6 | LHFPL tetraspan subfamily member 6 | 0.016625 |
| ENSG00000175768 | TOMM5 | Translocase of outer mitochondrial membrane 5 | 0.016747 |
| ENSG00000111331 | OAS3 | 2'-5'-oligoadenylate synthetase 3 | 0.016755 |
| ENSG00000121775 | TMEM39B | Transmembrane protein 39B | 0.016764 |
| ENSG00000153976 | HS3ST3A1 | Heparan sulfate-glucosamine 3-sulfotransferase 3A1 | 0.017014 |
| ENSG00000171649 | ZIK1 | Zinc finger protein interacting with K protein 1 | 0.017023 |
| ENSG00000006625 | GGCT | Gamma-glutamylcyclotransferase | 0.017143 |
| ENSG00000010292 | NCAPD2 | Non-SMC condensin I complex subunit D2 | 0.017373 |
| ENSG00000168476 | REEP4 | Receptor accessory protein 4 | 0.017433 |
| ENSG00000124784 | RIOK1 | RIO kinase 1 | 0.017833 |
| ENSG00000156265 | MAP3K7CL | MAP3K7 C-terminal like | 0.0182 |

| | | | |
|---|---|---|---|
| ENSG00000133863 | TEX15 | Testis expressed 15, meiosis and synapsis associated | 0.018214 |
| ENSG00000119537 | KDSR | 3-ketodihydrosphingosine reductase | 0.018318 |
| ENSG00000136938 | ANP32B | Acidic nuclear phosphoprotein 32 family member B | 0.018434 |
| ENSG00000169689 | CENPX | Centromere protein X | 0.018434 |
| ENSG00000004777 | ARHGAP33 | Rho gtpase activating protein 33 | 0.018441 |
| ENSG00000188985 | DHFRP1 | Dihydrofolate reductase pseudogene 1 | 0.018448 |
| ENSG00000186918 | ZNF395 | Zinc finger protein 395 | 0.01848 |
| ENSG00000174600 | CMKLR1 | Chemerin chemokine-like receptor 1 | 0.01862 |
| ENSG00000196368 | NUDT11 | Nudix hydrolase 11 | 0.018652 |
| ENSG00000150990 | DHX37 | DEAH-box helicase 37 | 0.018734 |
| ENSG00000185697 | MYBL1 | MYB proto-oncogene like 1 | 0.018917 |
| ENSG00000244067 | GSTA2 | Glutathione S-transferase alpha 2 | 0.018923 |
| ENSG00000100664 | EIF5 | Eukaryotic translation initiation factor 5 | 0.019011 |
| ENSG00000198959 | TGM2 | Transglutaminase 2 | 0.019042 |
| ENSG00000118620 | ZNF430 | Zinc finger protein 430 | 0.019286 |
| ENSG00000197785 | ATAD3A | Atpase family AAA domain containing 3A | 0.019473 |
| ENSG00000180801 | ARSJ | Arylsulfatase family member J | 0.019503 |
| ENSG00000102967 | DHODH | Dihydroorotate dehydrogenase (quinone) | 0.019559 |
| ENSG00000134987 | WDR36 | WD repeat domain 36 | 0.019636 |
| ENSG00000148459 | PDSS1 | Decaprenyl diphosphate synthase subunit 1 | 0.019636 |
| ENSG00000125863 | MKKS | Mckusick-Kaufman syndrome | 0.019636 |
| ENSG00000170802 | FOXN2 | Forkhead box N2 | 0.019636 |
| ENSG00000256663 | AC112777.1 | Ubiquitin-like with PHD and ring finger domains 1 (UHRF1) pseudogene | 0.019746 |
| ENSG00000136159 | NUDT15 | Nudix hydrolase 15 | 0.019801 |
| ENSG00000213397 | HAUS7 | HAUS augmin like complex subunit 7 | 0.019831 |
| ENSG00000145293 | ENOPH1 | Enolase-phosphatase 1 | 0.020034 |
| ENSG00000106348 | IMPDH1 | Inosine monophosphate dehydrogenase 1 | 0.020344 |
| ENSG00000166582 | CENPV | Centromere protein V | 0.020673 |
| ENSG00000075142 | SRI | Sorcin | 0.020801 |
| ENSG00000112365 | ZBTB24 | Zinc finger and BTB domain containing 24 | 0.020977 |
| ENSG00000173085 | COQ2 | Coenzyme Q2, polyprenyltransferase | 0.021454 |
| ENSG00000103404 | USP31 | Ubiquitin specific peptidase 31 | 0.021506 |
| ENSG00000187790 | FANCM | FA complementation group M | 0.021687 |
| ENSG00000172167 | MTBP | MDM2 binding protein | 0.022129 |
| ENSG00000111788 | AC009533.1 | DEAD/H (Asp-Glu-Ala-Asp/His) box polypeptide like pseudogene | 0.022278 |
| ENSG00000164619 | BMPER | BMP binding endothelial regulator | 0.022308 |
| ENSG00000116667 | C1orf21 | Chromosome 1 open reading frame 21 | 0.022407 |
| ENSG00000153048 | CARHSP1 | Calcium regulated heat stable protein 1 | 0.02256 |
| ENSG00000167460 | TPM4 | Tropomyosin 4 | 0.022708 |
| ENSG00000144395 | CCDC150 | Coiled-coil domain containing 150 | 0.022932 |
| ENSG00000103121 | CMC2 | C-X9-C motif containing 2 | 0.023199 |
| ENSG00000131462 | TUBG1 | Tubulin gamma 1 | 0.023226 |
| ENSG00000145375 | SPATA5 | Spermatogenesis associated 5 | 0.023396 |
| ENSG00000152402 | GUCY1A2 | Guanylate cyclase 1 soluble subunit alpha 2 | 0.023398 |
| ENSG00000119335 | SET | SET nuclear proto-oncogene | 0.0234 |
| ENSG00000253250 | C8orf88 | Chromosome 8 open reading frame 88 | 0.023473 |
| ENSG00000095319 | NUP188 | Nucleoporin 188 | 0.023811 |
| ENSG00000120253 | NUP43 | Nucleoporin 43 | 0.02403 |
| ENSG00000142149 | HUNK | Hormonally up-regulated Neu-associated kinase | 0.02403 |

| | | | |
|---|---|---|---|
| ENSG00000157483 | MYO1E | Myosin IE | 0.02403 |
| ENSG00000184900 | SUMO3 | Small ubiquitin like modifier 3 | 0.02403 |
| ENSG00000100522 | GNPNAT1 | Glucosamine-phosphate N-acetyltransferase 1 | 0.024368 |
| ENSG00000174136 | RGMB | Repulsive guidance molecule BMP co-receptor b | 0.024368 |
| ENSG00000122565 | CBX3 | Chromobox 3 | 0.024558 |
| ENSG00000165197 | VEGFD | Vascular endothelial growth factor D | 0.024558 |
| ENSG00000176887 | SOX11 | SRY-box transcription factor 11 | 0.024771 |
| ENSG00000092621 | PHGDH | Phosphoglycerate dehydrogenase | 0.02496 |
| ENSG00000131652 | THOC6 | THO complex 6 | 0.02496 |
| ENSG00000105968 | H2AZ2 | H2A.Z variant histone 2 | 0.025102 |
| ENSG00000136108 | CKAP2 | Cytoskeleton associated protein 2 | 0.025102 |
| ENSG00000148120 | AOPEP | Aminopeptidase O (putative) | 0.025102 |
| ENSG00000171067 | C11orf24 | Chromosome 11 open reading frame 24 | 0.025102 |
| ENSG00000140451 | PIF1 | PIF1 5'-to-3' DNA helicase | 0.025452 |
| ENSG00000174827 | PDZK1 | PDZ domain containing 1 | 0.025452 |
| ENSG00000179958 | DCTPP1 | Dctp pyrophosphatase 1 | 0.025452 |
| ENSG00000139350 | NEDD1 | NEDD1 gamma-tubulin ring complex targeting factor | 0.026216 |
| ENSG00000149596 | JPH2 | Junctophilin 2 | 0.026216 |
| ENSG00000167775 | CD320 | CD320 molecule | 0.026216 |
| ENSG00000130402 | ACTN4 | Actinin alpha 4 | 0.026375 |
| ENSG00000154127 | UBASH3B | Ubiquitin associated and SH3 domain containing B | 0.026375 |
| ENSG00000137547 | MRPL15 | Mitochondrial ribosomal protein L15 | 0.026558 |
| ENSG00000178531 | CTXN1 | Cortexin 1 | 0.026558 |
| ENSG00000131019 | ULBP3 | UL16 binding protein 3 | 0.02697 |
| ENSG00000139211 | AMIGO2 | Adhesion molecule with Ig like domain 2 | 0.027176 |
| ENSG00000134779 | TPGS2 | Tubulin polyglutamylase complex subunit 2 | 0.027356 |
| ENSG00000158716 | DUSP23 | Dual specificity phosphatase 23 | 0.027356 |
| ENSG00000051596 | THOC3 | THO complex 3 | 0.027561 |
| ENSG00000011332 | DPF1 | Double PHD fingers 1 | 0.027765 |
| ENSG00000148926 | ADM | Adrenomedullin | 0.027893 |
| ENSG00000142230 | SAE1 | SUMO1 activating enzyme subunit 1 | 0.028045 |
| ENSG00000121210 | TMEM131L | Transmembrane 131 like | 0.028222 |
| ENSG00000182054 | IDH2 | Isocitrate dehydrogenase (NADP(+)) 2 | 0.028398 |
| ENSG00000188229 | TUBB4B | Tubulin beta 4B class ivb | 0.0288 |
| ENSG00000242265 | PEG10 | Paternally expressed 10 | 0.0288 |
| ENSG00000166002 | SMCO4 | Single-pass membrane protein with coiled-coil domains 4 | 0.028923 |
| ENSG00000196730 | DAPK1 | Death associated protein kinase 1 | 0.028923 |
| ENSG00000160214 | RRP1 | Ribosomal RNA processing 1 | 0.029097 |
| ENSG00000102271 | KLHL4 | Kelch like family member 4 | 0.029244 |
| ENSG00000135801 | TAF5L | TATA-box binding protein associated factor 5 like | 0.029244 |
| ENSG00000055044 | NOP58 | NOP58 ribonucleoprotein | 0.02939 |
| ENSG00000198142 | SOWAHC | Sosondowah ankyrin repeat domain family member C | 0.02939 |
| ENSG00000139263 | LRIG3 | Leucine rich repeats and immunoglobulin like domains 3 | 0.029759 |
| ENSG00000108468 | CBX1 | Chromobox 1 | 0.030687 |
| ENSG00000156509 | FBXO43 | F-box protein 43 | 0.030687 |
| ENSG00000153885 | KCTD15 | Potassium channel tetramerization domain containing 15 | 0.030882 |
| ENSG00000198924 | DCLRE1A | DNA cross-link repair 1A | 0.031077 |
| ENSG00000227120 | AC009238.1 | Pseudogene similar to part of Cdon homolog (mouse) (CDON) | 0.031382 |
| ENSG00000124787 | RPP40 | Ribonuclease P/MRP subunit p40 | 0.03152 |
| ENSG00000147224 | PRPS1 | Phosphoribosyl pyrophosphate synthetase 1 | 0.03152 |

| | | | |
|---|---|---|---|
| ENSG00000172009 | THOP1 | Thimet oligopeptidase 1 | 0.03152 |
| ENSG00000128641 | MYO1B | Myosin IB | 0.031822 |
| ENSG00000112276 | BVES | Blood vessel epicardial substance | 0.031985 |
| ENSG00000173638 | SLC19A1 | Solute carrier family 19 member 1 | 0.031985 |
| ENSG00000126216 | TUBGCP3 | Tubulin gamma complex associated protein 3 | 0.032148 |
| ENSG00000075461 | CACNG4 | Calcium voltage-gated channel auxiliary subunit gamma 4 | 0.032283 |
| ENSG00000107371 | EXOSC3 | Exosome component 3 | 0.032283 |
| ENSG00000174013 | FBXO45 | F-box protein 45 | 0.032283 |
| ENSG00000182173 | TSEN54 | Trna splicing endonuclease subunit 54 | 0.033287 |
| ENSG00000105855 | ITGB8 | Integrin subunit beta 8 | 0.033476 |
| ENSG00000172732 | MUS81 | MUS81 structure-specific endonuclease subunit | 0.033664 |
| ENSG00000127334 | DYRK2 | Dual specificity tyrosine phosphorylation regulated kinase 2 | 0.03463 |
| ENSG00000131773 | KHDRBS3 | KH RNA binding domain containing, signal transduction associated 3 | 0.035003 |
| ENSG00000151388 | ADAMTS12 | ADAM metallopeptidase with thrombospondin type 1 motif 12 | 0.035314 |
| ENSG00000171960 | PPIH | Peptidylprolyl isomerase H | 0.035314 |
| ENSG00000172059 | KLF11 | Kruppel like factor 11 | 0.035684 |
| ENSG00000122861 | PLAU | Plasminogen activator, urokinase | 0.036481 |
| ENSG00000178776 | C5orf46 | Chromosome 5 open reading frame 46 | 0.036664 |
| ENSG00000114686 | MRPL3 | Mitochondrial ribosomal protein L3 | 0.036754 |
| ENSG00000117152 | RGS4 | Regulator of G protein signaling 4 | 0.036754 |
| ENSG00000125148 | MT2A | Metallothionein 2A | 0.036754 |
| ENSG00000101574 | METTL4 | Methyltransferase like 4 | 0.036873 |
| ENSG00000137801 | THBS1 | Thrombospondin 1 | 0.036993 |
| ENSG00000164167 | LSM6 | LSM6 homolog, U6 small nuclear RNA and mrna degradation associated | 0.036993 |
| ENSG00000198890 | PRMT6 | Protein arginine methyltransferase 6 | 0.036993 |
| ENSG00000234571 | H2BP2 | H2B histone pseudogene 2 | 0.037386 |
| ENSG00000101220 | C20orf27 | Chromosome 20 open reading frame 27 | 0.037472 |
| ENSG00000130520 | LSM4 | LSM4 homolog, U6 small nuclear RNA and mrna degradation associated | 0.037472 |
| ENSG00000136243 | NUP42 | Nucleoporin 42 | 0.037472 |
| ENSG00000118894 | EEF2KMT | Eukaryotic elongation factor 2 lysine methyltransferase | 0.037495 |
| ENSG00000138356 | AOX1 | Aldehyde oxidase 1 | 0.037495 |
| ENSG00000165175 | MID1IP1 | MID1 interacting protein 1 | 0.037495 |
| ENSG00000178031 | ADAMTSL1 | ADAMTS like 1 | 0.037495 |
| ENSG00000064666 | CNN2 | Calponin 2 | 0.037611 |
| ENSG00000070404 | FSTL3 | Follistatin like 3 | 0.037611 |
| ENSG00000114450 | GNB4 | G protein subunit beta 4 | 0.037905 |
| ENSG00000155189 | AGPAT5 | 1-acylglycerol-3-phosphate O-acyltransferase 5 | 0.038019 |
| ENSG00000128944 | KNSTRN | Kinetochore localized astrin (SPAG5) binding protein | 0.038133 |
| ENSG00000125458 | NT5C | 5', 3'-nucleotidase, cytosolic | 0.038215 |
| ENSG00000125753 | VASP | Vasodilator stimulated phosphoprotein | 0.038215 |
| ENSG00000117411 | B4GALT2 | Beta-1,4-galactosyltransferase 2 | 0.038359 |
| ENSG00000178860 | MSC | Musculin | 0.038359 |
| ENSG00000117228 | GBP1 | Guanylate binding protein 1 | 0.038535 |
| ENSG00000129474 | AJUBA | Ajuba LIM protein | 0.038853 |
| ENSG00000163655 | GMPS | Guanine monophosphate synthase | 0.038996 |

| | | | |
|---|---|---|---|
| ENSG00000130921 | C12orf65 | Chromosome 12 open reading frame 65 | 0.039659 |
| ENSG00000198168 | SVIP | Small VCP interacting protein | 0.040004 |
| ENSG00000148484 | RSU1 | Ras suppressor protein 1 | 0.040382 |
| ENSG00000115241 | PPM1G | Protein phosphatase, Mg2+/Mn2+ dependent 1G | 0.040455 |
| ENSG00000166483 | WEE1 | WEE1 G2 checkpoint kinase | 0.040455 |
| ENSG00000173218 | VANGL1 | VANGL planar cell polarity protein 1 | 0.040626 |
| ENSG00000155368 | DBI | Diazepam binding inhibitor, acyl-coa binding protein | 0.040797 |
| ENSG00000121690 | DEPDC7 | DEP domain containing 7 | 0.041276 |
| ENSG00000123843 | C4BPB | Complement component 4 binding protein beta | 0.041276 |
| ENSG00000284989 | AL451062.4 | Novel transcript | 0.041445 |
| ENSG00000197837 | H4-16 | H4 histone 16 | 0.041751 |
| ENSG00000111885 | MAN1A1 | Mannosidase alpha class 1A member 1 | 0.041852 |
| ENSG00000273213 | H3-2 | H3.2 histone (putative) | 0.041852 |
| ENSG00000125630 | POLR1B | RNA polymerase I subunit B | 0.042323 |
| ENSG00000091436 | MAP3K20 | Mitogen-activated protein kinase kinase kinase 20 | 0.042589 |
| ENSG00000106366 | SERPINE1 | Serpin family E member 1 | 0.042589 |
| ENSG00000106399 | RPA3 | Replication protein A3 | 0.042756 |
| ENSG00000105676 | ARMC6 | Armadillo repeat containing 6 | 0.043089 |
| ENSG00000170854 | RIOX2 | Ribosomal oxygenase 2 | 0.043089 |
| ENSG00000071282 | LMCD1 | LIM and cysteine rich domains 1 | 0.043221 |
| ENSG00000167552 | TUBA1A | Tubulin alpha 1a | 0.043221 |
| ENSG00000177192 | PUS1 | Pseudouridine synthase 1 | 0.043517 |
| ENSG00000181938 | GINS3 | GINS complex subunit 3 | 0.043517 |
| ENSG00000184207 | PGP | Phosphoglycolate phosphatase | 0.043517 |
| ENSG00000169683 | LRRC45 | Leucine rich repeat containing 45 | 0.043682 |
| ENSG00000285130 | AL358113.1 | Novel protein | 0.044011 |
| ENSG00000213390 | ARHGAP19 | Rho gtpase activating protein 19 | 0.044339 |
| ENSG00000125266 | EFNB2 | Ephrin B2 | 0.04483 |
| ENSG00000120708 | TGFBI | Transforming growth factor beta induced | 0.044957 |
| ENSG00000084774 | CAD | Carbamoyl-phosphate synthetase 2, aspartate transcarbamylase, and dihydroorotase | 0.045246 |
| ENSG00000086848 | ALG9 | ALG9 alpha-1,2-mannosyltransferase | 0.045246 |
| ENSG00000128510 | CPA4 | Carboxypeptidase A4 | 0.045246 |
| ENSG00000147050 | KDM6A | Lysine demethylase 6A | 0.045373 |
| ENSG00000151835 | SACS | Sacsin molecular chaperone | 0.045373 |
| ENSG00000141556 | TBCD | Tubulin folding cofactor D | 0.045499 |
| ENSG00000169684 | CHRNA5 | Cholinergic receptor nicotinic alpha 5 subunit | 0.045499 |
| ENSG00000168003 | SLC3A2 | Solute carrier family 3 member 2 | 0.045857 |
| ENSG00000119599 | DCAF4 | DDB1 and CUL4 associated factor 4 | 0.046142 |
| ENSG00000143554 | SLC27A3 | Solute carrier family 27 member 3 | 0.046142 |
| ENSG00000138772 | ANXA3 | Annexin A3 | 0.046302 |
| ENSG00000019549 | SNAI2 | Snail family transcriptional repressor 2 | 0.046477 |
| ENSG00000182197 | EXT1 | Exostosin glycosyltransferase 1 | 0.046477 |
| ENSG00000137656 | BUD13 | BUD13 homolog | 0.0466 |
| ENSG00000130810 | PPAN | Peter pan homolog | 0.046759 |
| ENSG00000143314 | MRPL24 | Mitochondrial ribosomal protein L24 | 0.047076 |
| ENSG00000182240 | BACE2 | Beta-secretase 2 | 0.047392 |
| ENSG00000163110 | PDLIM5 | PDZ and LIM domain 5 | 0.047549 |
| ENSG00000141858 | SAMD1 | Sterile alpha motif domain containing 1 | 0.048222 |
| ENSG00000186603 | HPDL | 4-hydroxyphenylpyruvate dioxygenase like | 0.048222 |
| ENSG00000204856 | FAM216A | Family with sequence similarity 216 member A | 0.048222 |

| | | | |
|---|---|---|---|
| ENSG00000050344 | NFE2L3 | Nuclear factor, erythroid 2 like 3 | 0.048497 |
| ENSG00000198796 | ALPK2 | Alpha kinase 2 | 0.048962 |
| ENSG00000038295 | TLL1 | Tolloid like 1 | 0.049079 |
| ENSG00000117395 | EBNA1BP2 | EBNA1 binding protein 2 | 0.049233 |
| ENSG00000101464 | PIGU | Phosphatidylinositol glycan anchor biosynthesis class U | 0.049772 |
| ENSG00000247077 | PGAM5 | PGAM family member 5, mitochondrial serine/threonine protein phosphatase | 0.049772 |
| ENSG00000138668 | HNRNPD | Heterogeneous nuclear ribonucleoprotein D | 0.049887 |
| ENSG00000197977 | ELOVL2 | ELOVL fatty acid elongase 2 | 0.049887 |
